# A Suite of Models to Support the Quantitative Assessment of Spread in Pest Risk Analysis

**DOI:** 10.1371/journal.pone.0043366

**Published:** 2012-10-09

**Authors:** Christelle Robinet, Hella Kehlenbeck, Darren J. Kriticos, Richard H. A. Baker, Andrea Battisti, Sarah Brunel, Maxime Dupin, Dominic Eyre, Massimo Faccoli, Zhenya Ilieva, Marc Kenis, Jon Knight, Philippe Reynaud, Annie Yart, Wopke van der Werf

**Affiliations:** 1 INRA, UR 633 Zoologie Forestière, Orléans, France; 2 Julius Kühn-Institute, Institute for National and International Plant Health, Kleinmachnow, Germany; 3 CSIRO Ecosystem Sciences, Canberra, Australia and Cooperative Research Centre for National Plant Biosecurity, Bruce, Australia; 4 Food and Environment Research Agency, York, United Kingdom; 5 Department of Agronomy, Food, Natural Resources, Animals and Environment (DAFNAE), University of Padova, Legnaro, Italy; 6 European and Mediterranean Plant Protection Organization, Paris, France; 7 Plant Protection Institute, Kostinbrod, Bulgaria; 8 CABI Europe-Switzerland, Delémont, Switzerland; 9 Imperial College London, Ascot, United Kingdom; 10 Horticultural Development Company (HDC), Agriculture and Horticulture Development Board, Kenilworth, United Kingdom; 11 Anses Laboratoire de la Santé des Végétaux, Montferrier-sur-Lez, France; 12 Centre for Crop Systems Analysis, Wageningen University, Wageningen, The Netherlands; National University of Singapore, Singapore

## Abstract

Pest Risk Analyses (PRAs) are conducted worldwide to decide whether and how exotic plant pests should be regulated to prevent invasion. There is an increasing demand for science-based risk mapping in PRA. Spread plays a key role in determining the potential distribution of pests, but there is no suitable spread modelling tool available for pest risk analysts. Existing models are species specific, biologically and technically complex, and data hungry. Here we present a set of four simple and generic spread models that can be parameterised with limited data. Simulations with these models generate maps of the potential expansion of an invasive species at continental scale. The models have one to three biological parameters. They differ in whether they treat spatial processes implicitly or explicitly, and in whether they consider pest density or pest presence/absence only. The four models represent four complementary perspectives on the process of invasion and, because they have different initial conditions, they can be considered as alternative scenarios. All models take into account habitat distribution and climate. We present an application of each of the four models to the western corn rootworm, *Diabrotica virgifera virgifera*, using historic data on its spread in Europe. Further tests as proof of concept were conducted with a broad range of taxa (insects, nematodes, plants, and plant pathogens). Pest risk analysts, the intended model users, found the model outputs to be generally credible and useful. The estimation of parameters from data requires insights into population dynamics theory, and this requires guidance. If used appropriately, these generic spread models provide a transparent and objective tool for evaluating the potential spread of pests in PRAs. Further work is needed to validate models, build familiarity in the user community and create a database of species parameters to help realize their potential in PRA practice.

## Introduction

Due to the intensification of world trade and the increase of travel and human activities throughout the world, more and more species are transported from their native area to new territories [1], [2]. Although only a small proportion of species are capable of establishing and spreading [3], it has been suggested that this proportion may be increasing due to global warming [4]. The number of non-indigenous terrestrial invertebrates and pathogens established outside their native area has been increasing dramatically in Europe in the last century [5], [6], causing serious concern for the European economy and environment [5], [7], [8]. Effective phytosanitary measures are required to revert or slow down this trend [9]–[11].

To assess the risk caused by invasive alien plant pests, Pest Risk Analyses (PRAs) are conducted on pest species to evaluate the probability of entry, establishment, spread and their potential impact in the PRA area. The conclusions of a PRA are used to decide whether risk management measures are required, and to determine which measures are the most appropriate [12]. The potential impacts associated with a pest invasion influence how much effort may be justified for prevention or management [13]. Potentially vulnerable assets can be identified using bioclimatic modelling tools, however, economic theory holds that for two pests that have similar vulnerable assets, the faster-spreading one is more costly, because its impacts will accrue more rapidly [13], [14].

Evaluating the potential spread of a pest in the PRA area is challenging because both spatial and temporal processes are involved. Besides, the area of potential establishment, the presence of natural dispersal barriers, the dispersal capability of individuals, the potential vectors of the pest, the potential movements with commodities and transport, but also predators, mutualisms and many other biotic and abiotic factors can be involved [15], [16]. Formal PRA schemes (e.g. [17]) typically require qualitative assessments of potential spread, through answers to questions such as: “How does the pest spread?”, “How far can the pest spread within a given time?”, “How fast can the pest invade the area of potential establishment?” and levels of uncertainty are often requested. Such qualitative assessments of the potential spread rely on expert judgment. Extrapolation can be made from other situations: the spread of the pest in another region or the spread of a closely related species (see PRAs made by the European and Mediterranean Plant Protection Organization, EPPO, [18]). In some cases, the qualitative assessment of potential spread is insufficient, and quantitative estimation is desirable, especially when the cost-effectiveness of phytosanitary measures is at stake [19].

**Figure 1:**
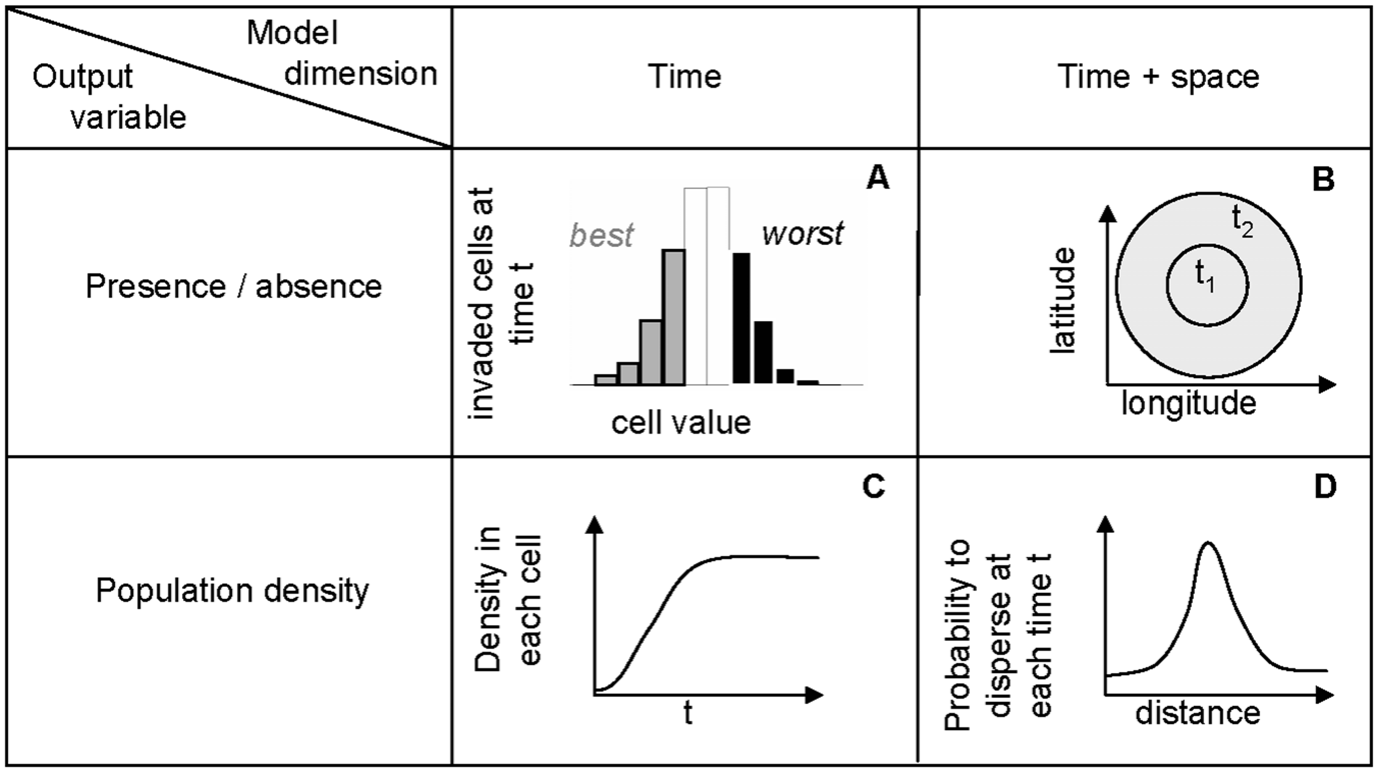
Classification of the models used for calculating scenarios of pest spread. Models A and B are models for occupancy of cells (presence/absence) on the PRA area. Models C and D are models for pest density. They calculate pest abundance within cells at given times *t*. Within each class, one model considers the process of spread only in the time dimension (A, C) while the other model considers processes in both time and space (B, D). The four models are further described in the text.

Theoretical models have been developed to quantify spread based on reaction-diffusion models (e.g. [20]–[23]). When these models do not fit the observed spread pattern because of long distance dispersal, stratified dispersal models that combine long distance jumps with local spread can be used [24], [25]. Some specific spread models have been developed to simulate the potential spread of a species taking into account human-assisted dispersal (e.g., [26]–[30]). These models address details of the life cycle and dispersal mechanisms, and they take considerable time and effort to develop, parameterise and test. It is not realistic to request the development of species-specific complex models in real world PRAs because risk assessors are generally not modellers, and they lack the time, resources and training to do it. Instead, there is a need for generic modelling tools in PRAs that can be used by risk assessors to capture the main processes driving the invasion process of alien species. While developments towards generalization and more unified application of complex modelling platforms in spread modelling for PRA are underway (e.g. [25], [31], [32]), there is as yet no modelling toolbox that risk assessors may use to conduct rapid appraisals of pest spread in the context of a PRA.

**Figure 2:**
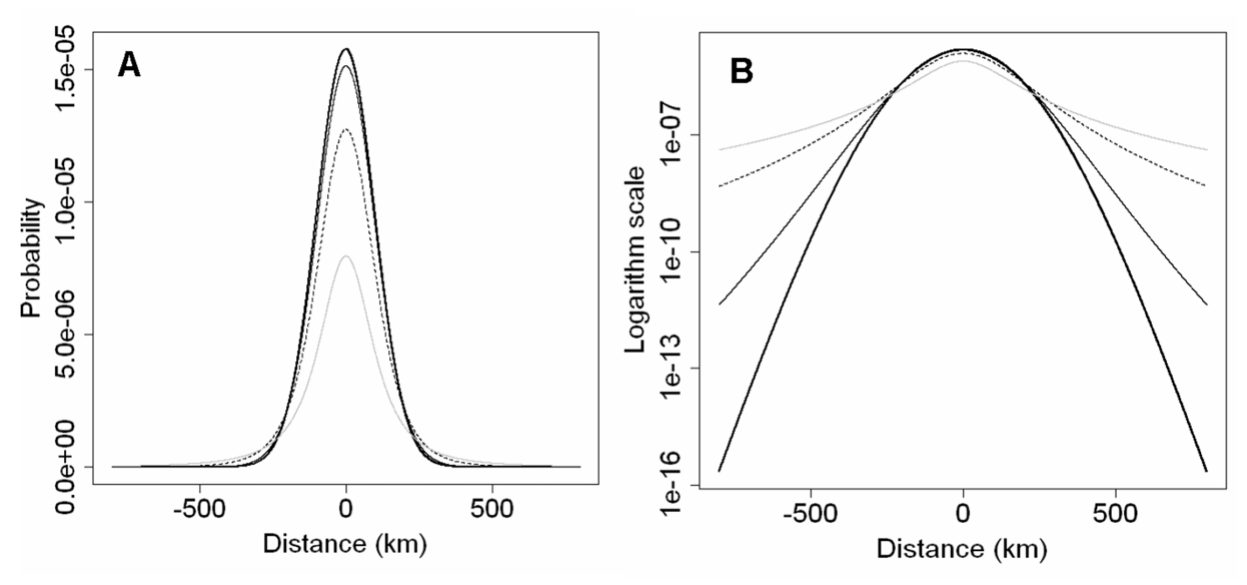
Four cross sections through a rotated t distribution for *u* = 100 km, and *ν* = 2 (grey line), *ν* = 5 (black dashed line), *ν* = 20 (thin black line), and *ν* = 100 (thick black line). In A, the kernels are shown with linear y-scale, whereas in B the same kernels are shown with logarithmic y-scale to bring out the differences in the fatness of the tails of the distributions.

The European Union 7^th^ Framework project PRATIQUE [33] aimed to deliver new tools to assist the risk assessor in the PRA. In this project, we developed a prototype for a generic spread modelling toolbox that risk assessors may use in PRAs in the future. The prototype consists of a suite of parsimonious ecological models for population growth and dispersal processes, with linkages to fundamental niche maps, based on climate suitability and presence of hosts or non-climatic habitat factors. While most of the model components are well established in the ecological literature, they have never before been brought together in an overarching integrated framework meant for future use (after appropriate testing and familiarization) in PRA. We conducted several case studies in collaboration with practical pest risk analysts with specific species knowledge to test the tools and develop a proof of concept. We present one case study in detail for illustration, including a sensitivity analysis. Finally, we report on the first expert feedback collected from these case studies. A detailed tutorial on the generic spread models is provided in Materials S1. Case studies are detailed in Materials S2. The spread module package which includes the R code and the files used for case studies can be downloaded at a permanent repository of the Royal Dutch Academy of Arts and Sciences: http://www.persistent-identifier.nl/?identifier=urn:nbn:nl:ui:13-jz0d-d5.

**Figure 3:**
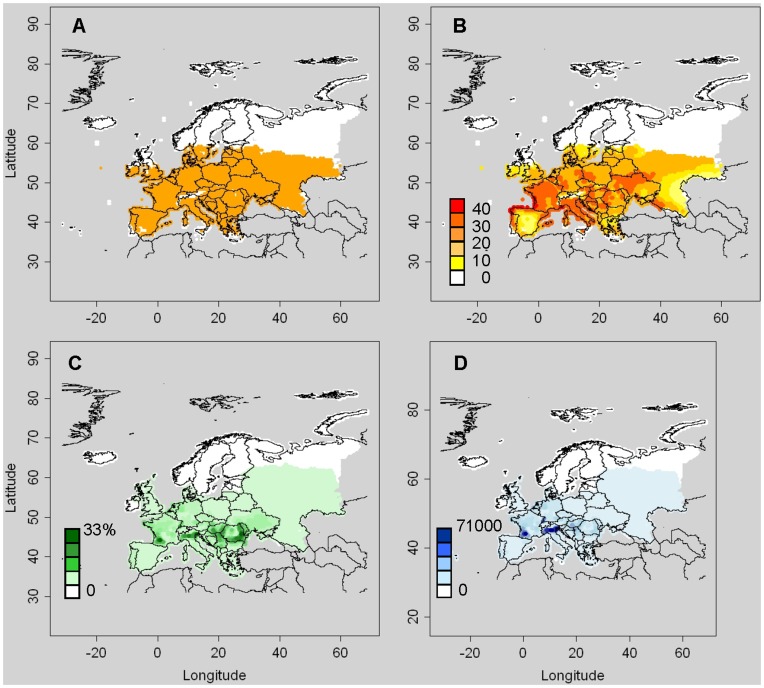
Suitable areas for *Diabrotica virgifera virgifera* and value of assets. A: Area where the ecoclimatic index (EI) is above zero, B: Growth index (GI) (source for A and B: Kriticos et al. 2012), C: Percentage of area covered by grain and forage maize, and D: Value of grain and forage maize in euros per km^2^ (source for C and D: McGill University 2011).

## Materials and Methods

### Definition of the Area of Potential Establishment

To apply the spread models for an invasive species, it is firstly necessary to define the area of potential establishment (the fundamental niche) of the alien species. This area is defined by favourable climatic conditions for long-term survival, the availability of host plants for plant pests and pathogens, suitable habitats and other abiotic factors, e.g. soil pH for plants and soil-dwelling organisms such as nematodes. Assessing the establishment potential of a pest in a PRA area is part of all PRA procedures and there is a large variety of methods and information sources to inform such assessments (e.g. [17], [34]–[36]).

**Table 1:** Parameter estimates for four spread models in seven case studies*.

			Parameters			
Species	Group	CLIMEX model	Model A	Model B	Model C	Model D
***Diabrotica virgifera virgifera***	Insect	Kriticos et al. 2012 [54]	*r* = 0.33 yr^−1^	*c* = 80 km/yr	*P*_max_ = 6.3×10^10^λ _max_ = 40	*P*_max_ = 6.3×10^10^λ _max_ = 40 *u* = 80 ν = 5
***Anoplophora chinensis***	Insect	D. Eyre, based on DeBoer(2004) [78]	Not applied	*c* = 1–2 km/yr	*P*_max_ = 1.9–19×10^7^λ _max_ = 6	*P*_max_ = 1.9–19×10^7^λ _max_ = 6 *u* = 2 ν = 10–50
***Anoplophora glabripennis***	Insect	D. Eyre, based on DeBoer(2004) [78]	Not applied	*c* = 1.5–3 km/yr	*P*_max_ = 7.58–15×10^6^λ _max_ = 5	*P*_max_ = 7.58–15×10^6^λ _max_ = 5*u* = 1.5–3 ν = 30–50
***Eichhornia crassipes***	Plant	EPPO & D. Kriticos [79]	Not applied	*c* = 30–100 km/yr	*P*_max_ = 7×10^7^ tons (of the plant) per grid cellλ _max_ = 30	*P*_max_ = 7×10^7^ tons (of the plant) per grid cellλ _max_ = 30*u* = 30–70 ν = 10–50
***Meloidogyne enterolobii***	Nematode	Z. Ilieva, unpublished	Not applied	*c* = 10–30 km/yr	*P*_max_ = 6.2×10^14^λ _max_ = 9.7	*P*_max_ = = 6.2×10^14^λ _max_ = 9.7*u* = 10–30 ν = 10–50
***Bursaphelenchus xylophilus/Monochamus***	Nematode/Vector beetle	C. Robinet unpublished, derived from Mediterranean template*	*r* = 0.27 yr^−1^	*c* = 6–35 km/yr	*P*_max_ = 14.69 infested trees per grid cellλ _max_ = 8.76	*P*_max_ = 14.69 infested trees per grid cellλ _max_ = 8.76*u* = 35 ν = 5
***Gibberella circinata***	Pathogen	Ganley et al. (2009) [80]	Not applied	Not applied	*P*_max_ = 5.21×10^8^λ _max_ = 2.72	*P*_max_ = 5.21×10^8^λ _max_ = 2.72*u* = 1 ν = 2

* Details are given in Materials S2.

Here we used the outputs of a CLIMEX model run on gridded climatic data [37]–[39] to define the area of potential establishment. We used two CLIMEX outputs in the spread modelling. These are the Ecoclimatic Index (EI, from 0 to 100) and the annual Growth Index (GI_A_, from 0 to 100; from here on written as GI), where the EI characterizes the suitability of the climate for long term survival and the GI indicates the potential for population growth during favourable seasons [38]. The CLIMEX model was run on a 0.5×0.5 degree grid [40]. As the dimensions of grid cells are defined in degrees, the size of grid cells varies with Latitude (Materials S1). To ensure consistency between map layers, the spread model implementation uses the grid on which the climatic and host inputs are defined. In the applications in this paper, the simulation grid for spread modelling is therefore a 0.5×0.5 degree grid. We define a “suitable cell” as a cell located within the area of potential establishment, based on climate and host or habitat, and an “invaded cell” as a cell that is not only suitable but also occupied by the study species. Unsuitable cells cannot be occupied.

**Figure 4:**
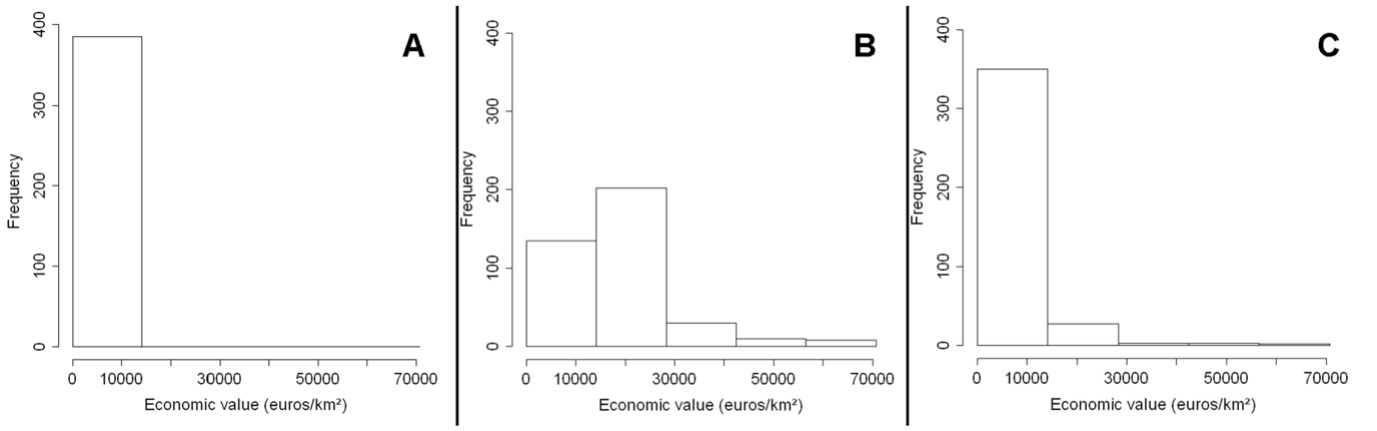
Frequency distribution of the potential economic impact of pest invasion in three scenarios of model A in a case study based on *Diabrotica virgifera virgifera*. The potential economic impact is quantified by accumulating the asset value in invaded cells in 2010. These three figures correspond to (A) best case scenario, (B) worst case scenario (C) random case scenario. Spread model A is based on logistic increase (*r* = 0.33 yr^−1^) in the number of invaded cells on the map.

**Figure 5:**
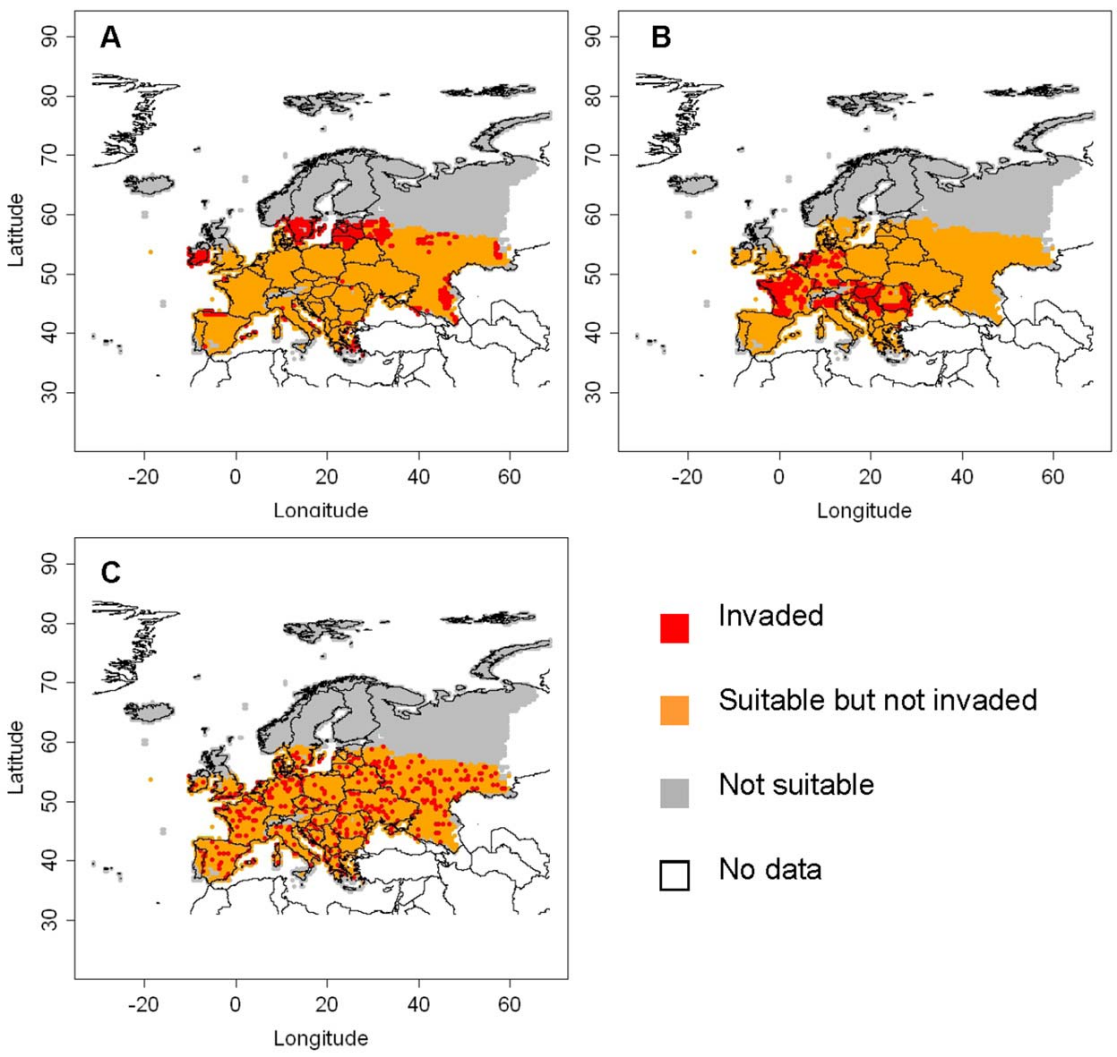
Spread simulation of *Diabrotica virgifera virgifera* for the year 2010 with model A. The invaded area (in red), non invaded area within the area of potential establishment (in orange), and area outside the area of potential establishment (in grey) for each of the three scenarios are shown (A: best case scenario, B: worst case scenario, C: random case scenario). The area coloured in white is outside the study area (no data).

### Description of the Spread Models

The four models developed here can be uniquely classified by considering two criteria (Fig. 1):

– whether the output variable is the occupancy of the pest (presence/absence – models A and B) or the pest density (percentage of the carrying capacity – models C and D) in each grid cell,
– whether the model ignores the geographical distance between the cells (spatially implicit – models A and C) or describes both temporal and spatial processes taking into account dispersal distance (spatially explicit – models B and D).

**Figure 6:**
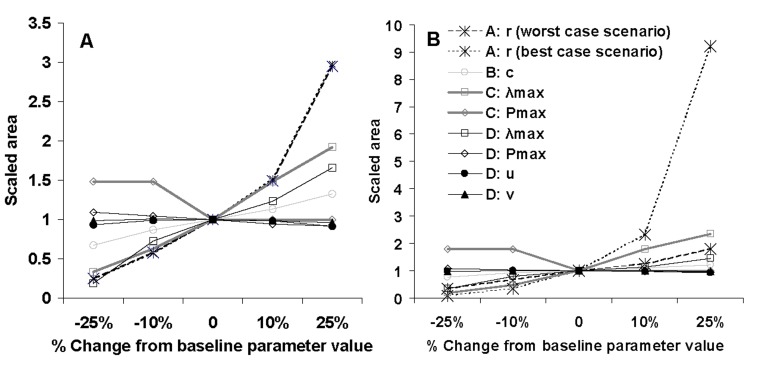
Sensitivity analysis: (A) total invaded area (km^2^) (for the baseline values in model A: 691,400–751,523 km^2^, model B (*t* = 8 yrs): 3,578,880 km^2^, model C: 59,790 km^2^, and model D: 2,010,382 km^2^), (B) total invaded maize acreage (for the baseline values in model A: 1,868–97,385 km^2^, model B: 174,706 km^2^, model C: 2,508 km^2^, and model D: 122,274 km^2^).

**Figure 7:**
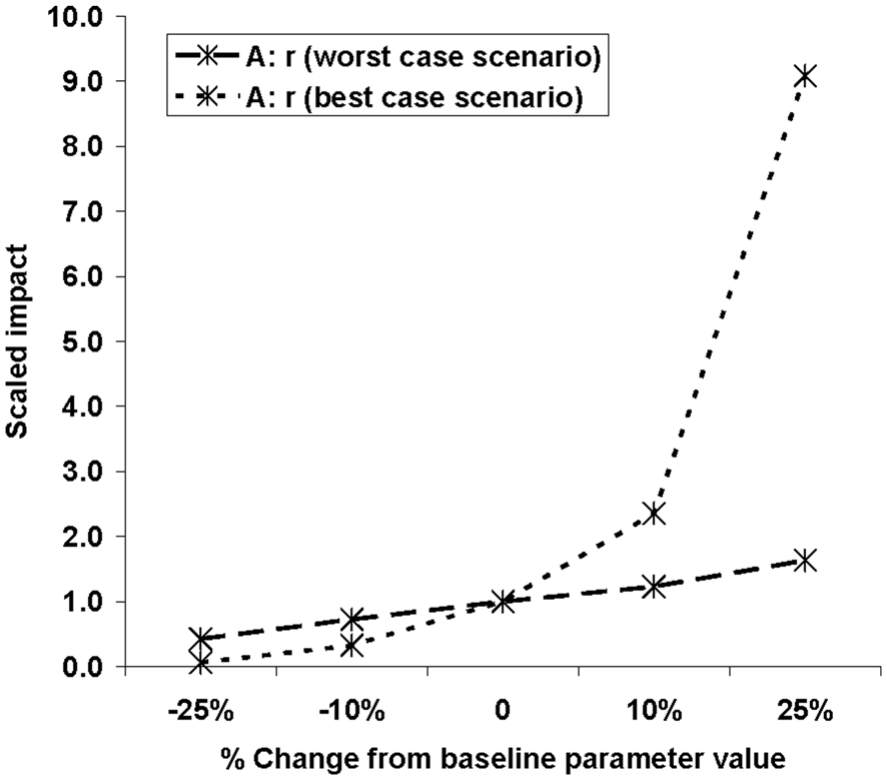
Sensitivity analysis: effects of parameter changes in model A on total impact. The impact (y-axis) is given as a multiple of the impact in the base line scenario (15 billion euros in the worst case scenario, and 150 million euros in the best case scenario).

Models A and C are not true spread models in a strict sense because they primarily focus on population growth over time which influences, but does not directly predict, the potential for population spread in space. However, these simple demographic models describe an important component of the invasion process and provide part of the theoretical basis for some true invasion models. They partly explain the spread rate (see [41]). Furthermore, these models have potential usefulness for PRA. We therefore consider all of these four models and call them “spread models” for brevity. Hereafter these models are described in more detail.

**Figure 8:**
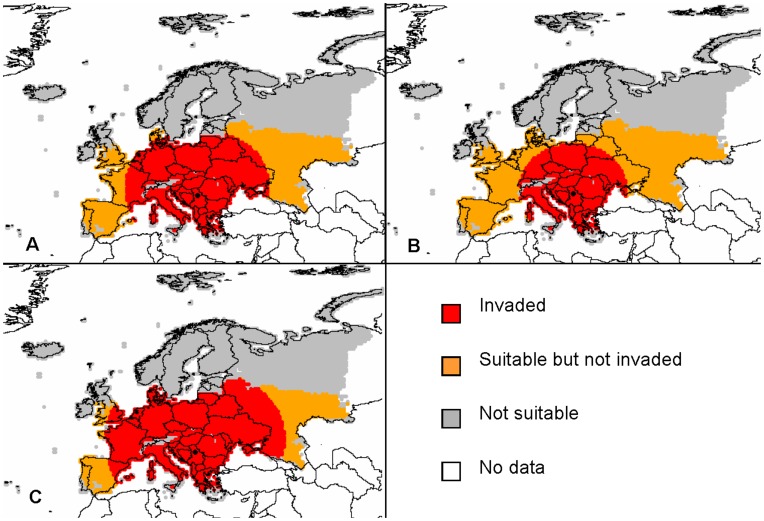
Spread simulation of *Diabrotica virgifera virgifera* for the year 2010 using model B for (A) the baseline value of *c* (80 km/yr), (B) the best case (−25%), and (C) the worst case (+25%).

### Model A: Output Variable is Occupancy and Model Ignores the Geographical Distance

The first model describes the logistic increase of the invaded area over time [42], [43]. The invaded area is determined by the number of cells and these cells are then allocated to positions within the area of potential establishment depending on their asset value, according to three scenarios: best, worst, and random. This occupancy model considers the increase of the number of invaded cells over time, starting from one or more invaded cells at the beginning of the simulation. The final state of the model is that all cells that are in the area of potential establishment are occupied. The logistic growth model is implemented as a difference equation at a one year time step:
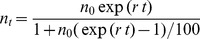
(1)where *n_t_* is the number of invaded cells as a percentage of the number of cells within the area of potential establishment at time *t*, and *r* is the relative rate of increase of the invaded area (yr^−1^). The user needs to identify one initial value, the percentage of cells initially invaded (*n*_0_, %) and estimate a single parameter, *r,* which is the only parameter in the model. In addition, the user needs to provide data on the economic values of assets (affected hosts) across the area of potential establishment because the model takes into account values at risk in each cell and assigns the new invasions preferentially to the most valuable cells in the worst case scenario, or to the least valuable cells in the best case scenario. There is also a random dispersal scenario in which invaded cells are assigned irrespective of their asset value. The economic impact is calculated directly in each of these scenarios by adding the asset value over all invaded cells at a given time *t*. The resulting number estimates the total asset value at risk in invaded cells. A damage function that calculates the actual yield loss in the invaded cells is not included here, but would be straightforward to add. The results can be summarized in a bar chart representing the frequency of invaded cells according to their economic value. Results can also be presented on a map, but the user should be aware that the location of invaded cells at time *t* is generated by a spatially implicit model that does not explicitly consider spatial processes.

**Figure 9:**
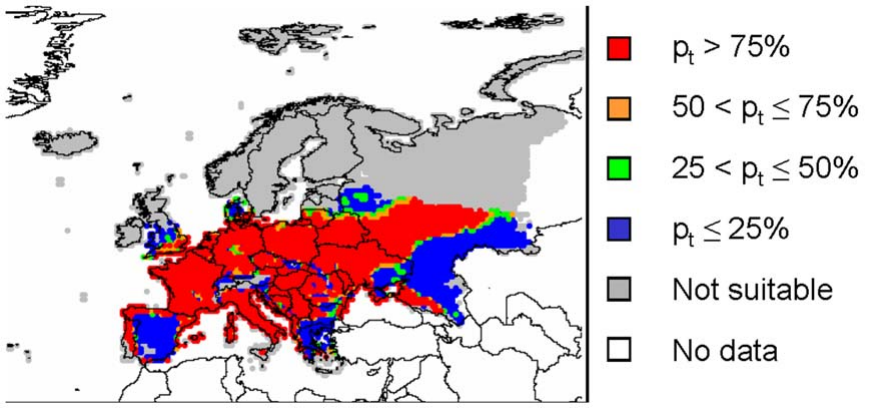
Spread simulation of *Diabrotica virgifera virgifera* for the year 2010 using model C with the baseline parameter values. There was virtually no difference between scenarios, therefore only the baselines scenario is given.

**Figure 10:**
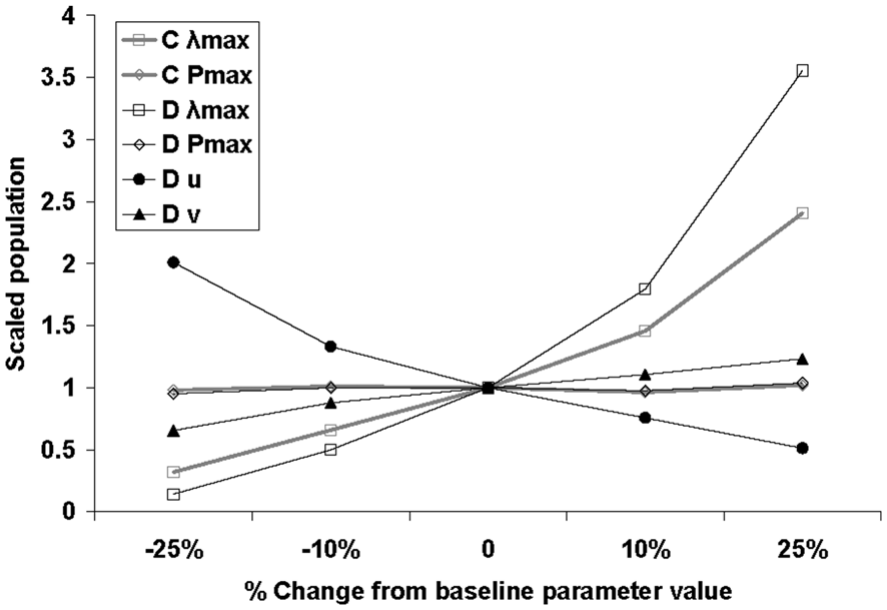
Sensitivity analysis: effect of parameter changes in models C and D on the total population. The population size (y-axis) is given as a multiple of the population size in the base line scenario (3.2×10^10^ in model C and 1.6×10^12^ in model D). The total population represents the total number of insects in the area of potential establishment.

### Model B: Output Variable is Occupancy and Model Considers the Geographical Distance

The second model is also an occupancy model. It simulates radial range expansion at a constant rate *c* (km/yr). Since the outcome is similar to reaction diffusion models (which generate an invasion wave with a constant asymptotic speed [44]), model B can be regarded as a simplified version of reaction diffusion models and the expansion rate *c* as an integrated estimate of both mean distance of dispersal and population growth. The user should provide the initial point(s) of entry, and the model will generate circles around this for different times *t*. The distance between circles describing the invaded range in subsequent years is defined by the radial rate of range expansion *c*. These circles represent the border of the maximum range and all suitable cells located in this range are considered invaded. In sum, this model has one parameter, *c*, and needs one or more entry points of the pest as initialization.

**Figure 11:**
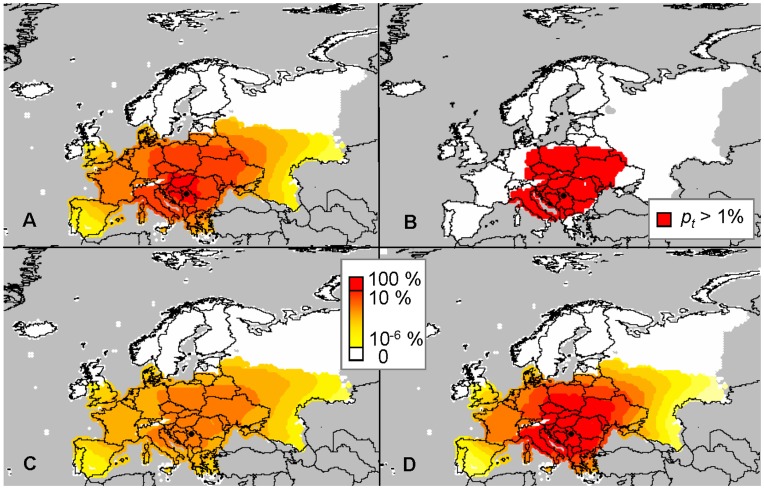
Distribution of *Diabrotica virgifera virgifera* in the year 2010 simulated with model D according to three scenarios: (A) the intermediate scenario with λ_max_ = 40, *u* = 80 km, ν = 5, and *P_max_* = 6.3×10^10^; (B) the intermediate scenario giving the area of potential presence (red indicates *p_t_* >1%), (C) best case scenario with λ_max_ = 30, *u* = 100 km, ν = 3.75, and *P_max_* = 7.9×10^10^; and (D) worst case scenario with λ_max_ = 50, *u* = 60 km, ν = 6.25, and *P_max_* = 4.7×10^10^. Grey indicates no data (outside of the study area).

### Model C: Output Variable is Density and Model Ignores the Geographical Distance

The third model is a density-based population dynamics model that describes logistic growth of the pest population independently within each cell. In this model, all suitable cells are initially infested at a relative density *p*_0_, expressed as a percentage of a carrying capacity *P*_max_. The initial relative density *p*_0_ is specified by the risk assessor. Population density at time *t*, *p_t_*, is expressed on a scale of 0–100 where 0 represents absence of the population and 100 represents the carrying capacity, *P*_max_. This carrying capacity is defined as the maximum population abundance in a cell with an average size and a given proportion of host cover. The proportion of host cover can be made cell-specific if sufficient data are available, but it is assumed to be spatially homogeneous in the case studies presented here. The population density within each suitable cell is calculated using a logistic growth function with a yearly time step and a yearly multiplication factor λ:
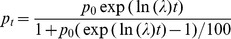
(2)


The yearly multiplication factor λ varies across the PRA area in accordance with the spatial variability of the climate, using the annual growth index (GI) calculated in CLIMEX:
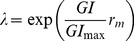
(3)where *r_m_* is the maximum intrinsic growth rate over the PRA area (realized where the conditions are best), GI is interpreted as a scaled form of the intrinsic growth rate, consistent with Sutherst et al. [35], and GI_max_ is the maximum value of GI over the PRA area, where λ = exp(*r*_m_). The term in brackets 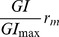 is the realized intrinsic growth rate in each grid cell. In practice, it may be easier to estimate the maximum multiplication factor within the PRA area, λ_max_, and estimate *r*_m_ as *r*_m_ = ln(λ_max_). In our model implementation, λ_max_ is the highest value of the maximum yearly multiplication factor reached within the PRA area, or in other words, it is the value of λ where GI has its highest value within the PRA area. This GI_max_ depends on the PRA area considered and is automatically generated by the model implementation (Materials S1). Therefore, λ_max_ should reflect the maximum multiplication rate of the pest realized within the PRA area, irrespective of whether a higher rate could be obtained elsewhere (i.e. outside the PRA area).

**Table 2:** Perturbation of invaded area with time for each parameter.

Model	Parameters		t = 8	t = 18	t = 28
Model A“worst”	Relative rate of increase	r	0.50	0.91	0.36
Model A“best”	Relative rate of increase	r	0.46	0.94	0.39
Model B	Spread rate	c	0.35	0.26	0.12
Model C	Yearly multiplication factor	λmax	0.85	0	0.02
	Carrying capacity	Pmax	−0.48	0	0
Model D	Yearly multiplication factor	λmax	ND (*)	0.50	0 (¤)
	Carrying capacity	Pmax	ND (*)	−0.10	0 (¤)
	Scale parameter	u	ND (*)	−0.01	0 (¤)
	Shape parameter	ν	ND (*)	−0.02	0 (¤)

A value of zero means that the scaled area does not change with a change of the parameter +/−10%. (*) ND means that the value is not defined because the invaded area for the baseline value is 0. (¤) These values are all below 4×10^−3^.

**Table 3:** Perturbation of invaded area with time for each model.

Model	t = 8	t = 18	t = 28
Model A worst	0.50	0.91	0.36
Model A best	0.46	0.94	0.39
Model B	0.35	0.26	0.12
Model C	1.29	0	0.02
Model D	ND (*)	0.70	0 (¤)

A value of zero means that the scaled area does not change with a combination of parameters +/−10%. (*) ND means that the value is not defined because the invaded area for the baseline value is 0. (¤) Since a change in the parameter values of model D has negligible effects on the invaded area at time t  = 18 yrs (see Table 2), it was not possible to define worst and best cases associated with this variable, and the sensitivity was set to 0.

This model has only one parameter affecting the dynamics (λ_max_) and one initial condition, *p*_0_ where the latter is estimated from information on the initial founder population and the maximum pest population in an average grid cell (*P*_max_). *P*_max_ is a scaling factor that relates relative population density *p_t_* (−) and absolute population density *P_t_* (number/grid cell). It is not a parameter in the true sense, but it must be estimated in order to calculate the initial condition *p*_0_. Over time, population densities in different cells will deviate as a result of differences in GI, i.e. climate suitability for population growth as assessed by a fundamental niche model such as CLIMEX. Although the results of this model are presented on a map, the user should be aware that patterns over the map represent spatial variation in climate suitability for the pest and the effect of this variability on population growth rate, and do not represent spatial dispersal processes. The added value of this model is that it visualizes the time that the pest needs to grow from an inconspicuous founder population in each 0.5×0.5 degree grid cell to widespread presence within such a cell.

**Table 4:** Experts’ assessment regarding the level of difficulty of parameter estimation in their case study (numbers indicate how often a score was given).

Model	Parameters		Easy	Somewhat difficult	Difficult	Impossible	*n*
Model A	Relative rate of increase	*r*	–	2	–	1	3
Model B	Spread rate	*c*	3	2	2	1	8
Model C	Yearly multiplication factor	λ_max_	–	2	6	–	8
	Carrying capacity	*P*_max_	1	3	4	–	8
Model D	Shape parameter	ν	–	–	8	–	8
	Scale parameter	*u*	4	1	3	–	8

**Table 5:** Experts’ assessment of the uncertainty of parameter estimates in their case study (numbers indicate how often a score was given).

Model	Parameters		Low uncertainty	High uncertainty	*n*
Model A	Relative rate of increase	*r*	1	1	2
Model B	Radial rate of range expansion	*c*	4	3	7
Model C/D	Carrying capacity	*P*_max_	4	4	8
	Yearly multiplication factor	λ_max_	2	6	8
Model D	Shape parameter	ν	-	8	8
	Scale parameter	*u*	4	4	8

**Table 6:** Experts’ assessment of the level difficulty to obtain data for model parameterisation in their case study (numbers indicate how often a score was given).

Model	Easy	Feasible	Difficult	Verydifficult	Model notapplicable^1^	*n*
Model A	–	1	1	–	6	8
Model B	4	3	–	–	1	8
Model C	–	4	4	–	–	8
Model D	–	3	3	2	–	8

1 Model A was deemed not applicable in 6 out of 8 cases, mostly because of the effort involved in obtaining spatially explicit data on the value of assets at risk. The spread model component of model A is relatively simple to apply, but was not tested separately.

**Table 7:** Experts’ feedback on the suitability of four models in practical pest risk assessment based on their experience on specific case studies (numbers indicate how often a score was given).

Model	Suitable for PRAsand should become acommon tool in PRAs	Suitable for PRAs andI may use it in the future	Suitable for PRAsbut needs improvement(s)	Not suitable forPRAs	*n*
Model A	–	1	–	1	2
Model B	3	3	–	1	7
Model C	–	6	2	–	8
Model D	2	2	3	1	8

### Model D: Output Variable is Density and Model Considers the Geographical Distance

This model considers population density in space and time based on the combination of local population growth with dispersal. The population growth process is logistic, as in model C. The dispersal process is modelled with an integro-difference equation [45]:
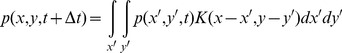
(4)where *x’* and *y’* are potential source locations, *x* and *y* are target locations, *K* is a spatial probability density (kernel) that specifies where individuals are moving in a time step of one year and *p* is the population density expressed as a percentage of the carrying capacity, *P*_max_, same as in model C. As a dispersal model, we use a t-distribution with two parameters, a length scale *u* (km) and a shape parameter *ν*:
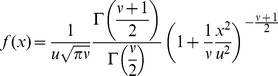
(5)where Γ is the gamma function, and *x* is distance from the source point. The standard deviation of this distribution is 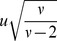. To use the kernel in 2-D, it is rotated, and the integration constant is adjusted to guarantee the probability mass equals 1:
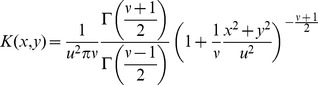
(6)([46]; Materials S1). Apart from details of parameterization, this distribution is identical to the 2Dt-distribution derived by Clark et al. (1999) [46], Eq. 8. The 2Dt-distribution has the advantage of a biologically plausible shape for the distribution of dispersal distances, due to the concavity (downward curvature; second derivative <0) of the peak at the origin (x = 0), a trait not shared by some frequently used distribution models from the exponential family (e.g. the Laplace and square root exponential distributions), and power laws [46]–[48]. The t-distribution approaches the fat-tailed Cauchy distribution for 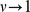, and the thinly tailed normal distribution for 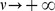. The Cauchy has been often used in spread studies (e.g. [24], [49], [50]). Because of its versatility, the rotated t-distribution is very suitable for dispersal modelling in studies on large scale spread [46]. It can be easily adjusted to reflect smaller or larger dispersal distances (by changing u) and larger or smaller frequency of long distance dispersal (by changing ν). The width of the distribution is regulated by the length scale u (km). The majority of the probability mass of the kernel is within 2u from the source (Fig. 2). The fatness of the tails, which reflects the likelihood of long-distance dispersal events, is determined by the parameterν. Small values of ν result in fat tails, while large values of ν result in thin tails. Fat tails are known to generate accelerating waves, i.e. a rate of range expansion that increase with time as the population front is “pulled” progressively by the satellite foci that are generated in the far tail of the dispersal distribution [47]. A kernel with fat tails may be used to represent a situation in which trade, for example, can be responsible for occasional spread events over much longer distances than are attained by biological spread mechanisms [23], [24]. The fatness of the tail is very difficult to discern on a linear scale (Fig. 2A), and is almost impossible to estimate from dispersal data. Therefore ν must be estimated by calibrating the simulated spread to the observed spread for the same organism in different areas, or for similar organisms in the same area. The rotated t-distribution with scale parameter *u* approaches the 2-D normal kernel with standard deviation *u* when the shape parameter *ν* of the t-distribution becomes very large. It approaches a rotated Cauchy distribution for *ν* → 1. However, the parameter *ν* must strictly be greater than 1, otherwise the distribution is undefined. In sum, model D has three parameters that the user needs to estimate: λmax, *u* and *ν*. Furthermore, the user needs to provide as initial condition the spatial location of one or more points of entry, and the corresponding sizes of the founder populations as expressed as a percentage of carrying capacity in an average cell, Pmax.

In the case studies, after fixing λ_max_ we use a heuristic calibration process to estimate the kernel parameters. Initial values are set for the length scale *u* (km) and the shape parameter ν (−) after which simulations were run to refine these estimates by calibration to known patterns of spread, taking into account biological information and expert judgement on dispersal mechanisms. We used the rule of thumb *u* = *c*, where *c* is the rate of radial range expansion in model B, to obtain an initial estimate of the length scale, *u*. An initial value for the shape parameter ν was set taking into account the analyst’s assessment of the propensity for long distance dispersal. A small value of ν was chosen (between 1 and 5) if long distance dispersal was judged to be frequent and the dispersal kernel was likely to have fat tails as a result. A large value of the shape parameter was chosen (e.g. ν = 100) if long distance dispersal events were considered very unlikely, and all dispersal would result from random walks, resulting in a normal distribution of dispersal distances [21]–[23], [42]. Here below we illustrate the estimation procedure using a case study.

### Detailed Case Study: the Western Corn Rootworm

#### Description of the case study and datasets

To show an example of applying the generic spread models, we chose an important maize pest species that is of great concern to Europe and the USA, and whose invasion process is well documented, the western corn rootworm, *Diabrotica virgifera virgifera*. This species is considered to be native to Mexico, and was first detected in Europe in 1992 (in Belgrade, Serbia) [51], [52]. It has since spread rapidly throughout central and south-eastern Europe [53].

For the climatic layer, we used a recently published CLIMEX model for the western corn rootworm [54] in combination with 1961–1990 mean monthly climate interpolated over Europe at 0.5° by 0.5° spatial resolution by the Climate Research Unit of the University of East Anglia (Norwich, UK) [55]. The area of potential establishment was defined as the collection of cells where EI>0 and maize was present. Grain and forage maize distribution (for host distribution) and yield (for economic data on host value – needed in model A) were retrieved from McGill University [56]. Maps of EI, GI and maize presence indicate that a large part of Europe is suitable for establishment while the areas with the highest value of assets are located in northern Italy and south-western France (Figure 3).

Initial conditions in the spread models were those in 1992 (time *t* = 0 in the models) and we modelled the situation in 2010 for comparison to reality (corresponding to *t* = 18, see 2010 distribution in [53]). The entry point taken in models B and D was Belgrade (N 44.82°; E 20.30°) [52].

#### Parameterisation of model A for *D. virgifera virgifera*

Model A uses a logistic equation for the number of invaded cells. This equation is linearized (Eq. 7) to estimate *r* from invasion data:
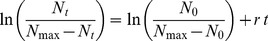
(7)where *N_t_* is the number of invaded cells at time *t* and *N*_max_ the number of suitable cells in the PRA area. We assume that only one cell was initially infested in 1992, therefore we have *N*_0_ = 1. In 2010, *N*_18_ = 358 cells were invaded, based on the observed distribution map in 2010 [53]. The number of cells within the area of potential establishment was *N*_max_ = 3104 cells, calculated from CLIMEX outputs (EI) and maize distribution (Figure 3). We find *r* = 0.33 yr^−1^.

#### Parameterisation of model B for *D. virgifera virgifera*

In Europe, the observed spread of *D. virgifera virgifera* is not spatially homogenous [53] because maize is not uniformly distributed and the role played by environmental factors and control measures varies in different areas. In a PRA context, we are primarily interested in potential spread independent of human intervention so that it is then possible to estimate the costs and benefits under control scenarios. Without containment measures, the spread of the *D. virgifera virgifera* ranged from 60 to 100 km per year assuming maize is continuously distributed [57]–[60]. Since the average natural spread rate of *D. virgifera virgifera* in Europe is approximately 80 km/year [60], we took *c* = 80 km/year.

#### Parameterisation of model C for *D. virgifera virgifera*

According to Hemerik et al. [61], the maximum yearly multiplication factor, λ_max_, for *D. virgifera virgifera* is approximately 40 in the Balkans, but lower in northern regions. For instance, Kruegener et al. [62] calculated a multiplication factor of 7.5 for German conditions. Since λ_max_ represents the maximum multiplication factor over the PRA area, we chose the value of 40. In other areas, the multiplication factor decreases with decreasing GI, down to 0 where GI = 0.

The initial population density (*p*_0_, %) is defined by: 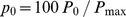 where *P*_0_ is the initial founder population, e.g. 100 beetles, in each suitable cell, and *P_max_* is the carrying capacity, the maximum number of individuals in a cell. We calculated *P*_max_ from observations in Serbia and Hungary, where beetle densities of 20–50 beetles per plant were counted [63]–[65]. Assuming a maize density of 55 000 plants ha^−1^ was common in Serbia in the 1990s [66], the abundance of adult beetles reached 1.1×10^6^ ha^−1^ of maize. In Italy, the maximum was estimated at 2.75×10^6^ ha^−1^ of maize. Based on these data, the maximum number of beetles was assumed to be 200 m^−^^2^ of maize (as a mean value of the calculated data), equivalent to 2×10^8^ beetles per km^2^ of maize. The maximum population size in a grid cell (*P*_max_) is the product of the area of the cell (km^2^), the proportion of the cell covered by the host (we assume 20% of the area of a grid cell where maize is present is grown with maize), and the maximum population density of the pest per unit host area (km-^2^). The mean area of the cell is determined by the grid resolution. The CLIMEX model for *D. virgifera virgifera* has a grid resolution of 0.5° longitude by 0.5° latitude [54]. The size of the cells varies with the cosine of the latitude and decreases from South to North. The average cell size in the simulated area of Europe is 1 578 km^2^ (this value is calculated by the implemented model code). Thus, we have *P_max_* = 6.3×10^10^ beetles per cell and *p*_0_ = 1.6×10^−7%^.

#### Parameterisation of model D for *D. virgifera virgifera*

The population dynamics parameter λ_max_ and the estimates of carrying capacity *P*_max_ and initial population *p*_0_ (%) are the same as in model C. In addition we need a length scale *u* (km) and a shape parameter ν (−), characterizing the dispersal kernel. These parameters can be estimated from dispersal data if these are available, but usually dispersal data on the pertinent scale (the continental) are lacking. Another option is to estimate the dispersal parameters from data on invasion. As the rate of population expansion results from a complex interaction between the three parameters λ_max_, *u* and ν [47], the parameters are estimated from invasion data using a calibration approach.

In the case of *D. virgifera virgifera*, since we suspect that a large proportion of individuals disperse over long distances within Europe [30], [67], we used ν = 5, giving a fat-tailed kernel. For the scale parameter, we took *u* = 80 km [60]. These initial guesses provided satisfactory simulations and no further adjustments were made.

#### Sensitivity analysis

We tested the sensitivity of model behaviour to parameter changes in each of the four spread models for *Diabrotica virgifera virgifera*. When we tested the sensitivity to more than one parameter for a given model, we first made a one-at-a-time analysis and took the baseline estimate(s) for the other parameter(s). For comparison, we considered three response variables in 2010: (1) the total area invaded (for models A, B, C, D), (2) the total pest population (for models C and D), and (3) the total economic damage (for model A only) over the entire PRA area. The total area invaded was calculated in two ways: (i) as the total area of invaded cells in km^2^, (ii) as the total maize area invaded. For models C and D, we defined a threshold above which a species was considered to be present and readily detectable. As a threshold, we took an arbitrary value of 1% of the carrying capacity *P*_max_. To estimate the total pest population, we considered the population level in each cell based on the carrying capacity *P*_max_ adjusted for the maize area in the cell [56], and then we summed this quantity over the PRA area. To estimate the potential total economic impact, we calculated the value of assets at risk in the invaded cells, again using data from McGill University [56]. When plotting the results of sensitivity analysis, we scaled the response variable to the output in the default parameterization. To obtain the non scaled values, the scaled values should be multiplied by the baseline result.

#### One-at-a-time analyses: perturbation of response variables for t = 18 years

In the sensitivity analysis of model A, we took *r* = 0.25, 0.30, 0.36, and 0.41 yr^−1^ (−25%, −10%, +10%, +25% as compared to the base line value of 0.33 yr^−1^). In the sensitivity analysis of Model B, we used *c* = 60, 72, 88, and 100 km/year (−25%, −10%, +10%, +25% as compared to the base line value of 80 km/yr). In the sensitivity analysis of model C, we used λ_max_ = 30, 36, 44, and 50 (−25%, −10%, +10%, +25% as compared to the base line value of 40). Furthermore, in model C, we conducted a sensitivity analysis for the carrying capacity in each cell: *P*_max_ = 4.7×10^10^, 5.7×10^10^, 6.9×10^10^ and 7.9×10^10^ (−25%, −10%, +10%, +25% as compared to the base line value of 6.3×10^10^). Changing *P*_max_ affects the initial value for *p*_0_, the relative population density (as compared to *P*_max_). In the sensitivity analysis of model D, we included the simulations for different λ_max_ and *P*_max_ as reported for model C. Furthermore, we studied the sensitivity to changes in the kernel length scale *u* and the shape factor ν. We used *u* = 60, 72, 88 and 100 km, and ν* = *3.75, 4.5, 5.5 and 6.25 (−25%, −10%, +10%, +25% as compared to the base line values of *u* = 80 km and ν = 5). Although the chosen values for the parameter ν are not integers, the volume underneath the rotated t distribution remains equal to one and can therefore be used as a dispersal kernel.

#### One-at-a-time analyses: perturbation of the invaded area with time

To summarize and compare the sensitivity of the models to each parameter, we calculated the difference between the scaled areas when the parameter values are increased and decreased by 10%. The scaled area is the ratio of the total area of invaded cells calculated with a change of the parameter value by this area with the baseline parameter value. The area is the only response variable that could be used to compare all the models. We considered *t* = 8, 18 and 28 years to explore the change in the area invaded with time.

#### Multi-parameter changes: perturbation of potential spread for t = 18 years

Furthermore, we generated for each model three maps corresponding to the best, most likely and worst cases based on changes in multiple parameters to their “best case”, “worst case” and “most likely” settings. Here, best case and worst case represent minimum and maximum expected spread, respectively, while “most likely” refers to the combination of parameter values considered most plausible by the species experts.

#### Multi-parameter changes: perturbation of invaded area with time

To summarize and compare the overall sensitivity of the models to their parameters, we calculated the difference between the scaled invaded areas simulated with the “worst case” and the “best case”. These cases were defined by the combination of the parameter values (+/−10%) which gave respectively the largest and the smallest invaded areas. We chose *t* = 8, 18 and 28 years to explore the perturbation of invaded area with time.

### Experts’ Feedback

The generic spread model was applied to other species representing a wide variety of groups: insects, nematodes, pathogens and plants. The objective was to assess how well the spread modelling concepts could be applied to a broad range of invasive species and determine whether these generic spread models can fulfil experts’ requirements in the context of PRA. A total of six species were tested in addition to *D. virgifera virgifera*: the citrus longhorn beetle, *Anoplophora chinensis* and the Asian longhorn beetle, *Anoplophora glabripennis*; the root-knot nematode, *Meloidogyne enterolobii*, the insect-vectored pine wood nematode, *Bursaphelenchus xylophylus*; water hyacinth, *Eichhornia crassipes*, and the fungus *Gibberella circinata*, causal agent of pitch canker disease. The parameters were estimated in collaboration between PRA experts and modellers (Table 1, details in Materials S2). We collected feedback about how difficult the experts found the parameterisation, how uncertain the estimations were, how difficult it was to obtain enough information to apply the models, and whether the experts considered the spread models potentially useful in PRA. A total of eight experts gave feedback: two experts for *A. chinensis* and one for each of the six other case studies.

## Results

### Application to the Western Corn Rootworm

#### Simulations with model A

This model calculates a logistic increase in the number of invaded cells across the map, assigning invasions according to the value of the assets in the cells. In the worst case scenario, which assigns new invasions preferentially to cells with high asset value (Fig. 4), a large part of France, Germany, northern Italy, and central Europe were invaded (Fig. 5), representing an asset value at risk in the invaded area of 15.2 billion euros. In the best case scenario, which assigns new invasions preferentially to cells with a low asset value (Fig. 4), peripheral areas in the East and South were invaded (Fig. 5), representing an asset value at risk in the invaded area of 150 million euros. The random case was intermediate as it assigns invasions randomly across Europe (Fig. 4, 5). The *number* of cells invaded in the three economic scenarios was the same, but their location was different, in accordance with the asset value.

A change in the value of the relative rate of increase of the number of invaded cells (*r*) resulted in large changes in the invaded area (Fig. 6A), and the effect was the same across the economic scenarios. Only three percent of the cells in the area of potential establishment were invaded if *r* was reduced by 25%, whereas 34% were invaded if *r* was increased with 25%, versus 12% invaded cells in the baseline scenario. The invaded maize area differed between the economic scenarios because cells differ in the area of maize within them. While cells without maize are not eligible for assigning new invasions, the model can preferentially assign pest invasion to cells with high or low areas and value of maize. In the best case scenario, very little maize acreage (0.059%) was invaded if *r* was decreased by 25% whereas 7% of the total maize growing acreage was invaded if *r* was increased by 25% (Fig. 6B), versus 0.8% for the baseline value of *r*. In the worst case scenario, 15% of the maize growing acreage was invaded if *r* was decreased by 25% whereas 75% of the maize growing acreage was invaded if *r* was increased by 25% (Fig. 6B) versus 42% for the baseline value of *r*. In terms of economic impact, this change also had a major effect: 6.5 to 24.8 billion euros in the worst case scenario and 9 to 1,360 million euros in the best case scenario (Fig. 7). Model A was thus sensitive to changes in its growth parameter, especially in the best case scenario (Figs. 6, 7).

#### Simulations with model B

This model calculates invasion according to spatial expansion of the invaded area at a constant radial rate of range expansion. Simulations with model B indicated that *D. virgifera virgifera* had spread over a large part of Europe by 2010 (Fig. 8). More than half of the area of potential establishment was invaded within 18 years if no containment measures were applied. A change in the radial expansion rate *c* in the sensitivity analysis greatly affected the total invaded area in 2010, from 37 to 75% of the cells invaded, and from 57 to 90% of the maize area invaded if the radial rate of expansion was either decreased or increased by 25% compared to its baseline value (Figs. 6, 8). Model B was therefore moderately sensitive to changes in the radial rate of range expansion.

#### Simulations with model C

This model simulates local population growth in each cell, based on an initial presence of the pest at low density throughout the area of potential establishment. Results indicate that large parts of Europe (especially Central Europe, Poland, Germany, France, and Italy) are favourable for the population growth resulting in high densities (Fig. 9) as soon as 2005–2007, i.e. 13–15 years after entry. It should be noted, however, that actual entry was at one location, whereas this model assumes that entry has occurred in every cell. A change in the parameters had limited effect on the results in 2010 because most of the area of potential establishment was invaded (data not shown). The model was more sensitive in 2000 (*t* = 8 years after entry). The simulated total population in 2000, integrated over the PRA area, varied from 3.1 to 3.3×10^10^ beetles when changing *P*_max_ but the abundance varied a little more, from 1.0 to 7.7×10^10^ beetles when changing λ_max_ (Fig. 10). The proportion of invaded cells (where *p_t_* >1%) varied from 0.3 to 1.7% and the proportion of invaded maize growing area varied from 0.2 to 2.5% when changing λ_max_ and there were only marginal effects when changing *P_max_* (Fig. 6). On the whole this model was moderately sensitive to changes in the parameters.

#### Simulations resulting from model D

According to this model, a large part of Central Europe is invaded by 2010 (Fig. 11). The population spread far from the source point in Belgrade and it had – in the simulation - invaded (albeit at low density) nearly the whole area of potential establishment by 2004 (map not shown here). When considering presence only where the model simulated a density above 1% of the carrying capacity, the potential range of *D. virgifera virgifera* in 2010 was consistent with the known spatial extent of the population in this year (Fig. 11B, [53]). In terms of population density, none of the parameters had a very strong effect. The range of population size over the PRA area was 0.2 to 5.7×10^12^ with the largest variation for λ_max_, moderate variation for *u* and very low variation within this range for *P_max_* and ν (Fig. 10). In terms of total invaded area, λ_max_ had the strongest effect (Fig. 6), with the proportion of invaded cells ranging from 6 to 53% and the invaded maize acreage ranging from 17 to 76%. Since the total population increased with λ_max_, we used the highest value (+25%) for the worst case and the lowest value (−25%) for the best case. For *P_max_*, since there was no clear effect on the total population but the invaded area decreased when increasing *P*_max_, we considered the lowest value (−25%) for the worst case and the highest value (+25%) for the best case. Changing parameter *u* produced no clear effect on the invaded area but the total population decreased when increasing *u*, due to spillover of individuals outside the area of potential establishment. We considered the lowest value (−25%) for the worst case and the highest value (+25%) for the best case. Changing ν had no clear effect on the invaded area but the total population increased when increasing ν, again due to less spillover as a result of thinner tails, so we considered the highest value (+25%) for the worst case and the lowest value (−25%) for the best case. In the best case scenario, the population density was lower everywhere in the PRA area (Fig. 11) and in the worst case, the population density was higher than in the most likely scenario but the overall pattern was similar.

### Comparison of Models’ Sensitivity: Perturbation of the Invaded Area with Time

The sensitivity varied greatly between the models, the parameters and the three time periods (Tables 2, 3). Model A was globally the most sensitive, especially when time *t* = 18 years. Model B was moderately sensitive to its parameter and its sensitivity decreased with time. Model C was highly sensitive to both parameters at time *t* = 8 years but caused little change to the invaded area (Table 2). We obtained the same result when simultaneously changing the parameters (Table 3). In this model, all the area of potential establishment is invaded by a low pest density at time  = 0, the population density increases rapidly until the threshold for establishment is reached. As a result, for a large time *t*, changes to the values of the parameters have little effect on the invaded area. Model D was not very sensitive to parameter change, except for the yearly multiplication factor at time *t* = 18 years (Table 2). Simultaneously changing the values of the parameters accentuated its sensitivity (Table 3). For earlier times (*t* = 8), the population density was too low and remained below the threshold for establishment. Since the invaded area for the baseline values was 0,it was not possible to calculate the scaled area. For later times (*t* = 28), the population had time to spread nearly everywhere in the area of potential establishment and the model was thus insensitive to small variations in its parameters. Consequently, the choice of time *t* to evaluate the sensitivity of the models was very important overall.

### Experts’ Feedback

The application of the spread models to other case study species showed that the models could be applied for a broad range of plant pests and invasive plants (Table 1; details in Materials S2). Data availability and difficulty of parameter estimation were important issues. Model A requires data on host or habitat value and was applied on only two out of the seven test species because economic data on assets at risk were difficult to obtain. This model was applied without too much difficulty for *Diabrotica virgifera virgifera*, for which the economic data were readily available [56]. The model was also applied – but with some difficulty – to pine wood nematode, but not in the other case studies. The easiest parameter to estimate was the radial rate of range expansion, *c*, in Model B, because it can be directly derived from range expansion data. The most difficult parameter to estimate was the measure for the fatness of the tail of the dispersal kernel, ν, in model D (Table 4), not surprisingly, because there is no direct method and applicable data with which to estimate it. The multiplication factor λ_max_ was also found relatively difficult to estimate, whereas the scale parameter *u* of the dispersal kernel was deemed easier to estimate. The shape parameter (ν) of the dispersal kernel and the annual multiplication factor (λ_max_) were rated as having high uncertainty (Table 5). The models A–D differed in the ease with which data can be obtained to inform the parameter estimation (Table 6). Model A was considered not applicable due to lack of data in five of the seven case studies. Of the other three models, model B was relatively easy or feasible to apply whilst models C and D were feasible to difficult. Although model B was generally the easiest to apply, it could not be applied to *G. circinata* (Tables 1, 4–6) because this pathogen mostly disperses between nurseries and a radial expansion process was deemed inconsistent with its dispersal mode by the risk assessor. Table 7 summarizes experts’ feedback on the usefulness of the models in PRA. Model A was rated by two experts, with different results. Experts considered models B, C and D suitable for PRA, but risk assessors asked for more guidance in parameter estimation and examples to help them parameterizing and using models C and D.

## Discussion

The generic spread models presented here, give a set of different scenarios that can help risk assessors to determine the potential temporal or spatial expansion of pests in a pest risk assessment. The spatially implicit model for logistic growth of the amount of occupied area (Model A) provides a direct assessment of the assets at risk, but it requires spatially explicit data on their economic value. The difficulty of obtaining such economic data was the main reason why risk assessors found this model difficult to use in their case study, despite the simplicity of the underlying logistic spread model. The range expansion model B provides an easy way to map the potential geographic spread of the pest through time, but it suffers from lack of biological realism, especially if the pest enters an area that is marginally suitable for survival and growth and where the rate of range expansion would be lower than that predicted by this model. The spatially implicit model for logistic density increase (Model C) can be used to identify areas where the pest can grow rapidly to damaging levels. The spatially explicit model for population growth and dispersal (Model D) simulates both spatial and temporal dynamics more realistically using only three biological parameters: λ_max_, *u* and ν. Out of all these simple models, model D can be considered to be the most sophisticated. The increase of model complexity provides more flexibility and the ability to describe a wider range of processes. Thus, complex models can potentially give better descriptions of pest spread. For instance, model D can simulate both short and long distance dispersal. These two dispersal mechanisms cannot be simulated by the other models. However, increasing model complexity means that more parameters are needed, more data are required for their estimation and the overall uncertainty in the parameter values can play an important role. To assist the PRA process, spread models should be applicable to a wide variety of species for which more or less amount of information are available. Consequently, these models should stay as simple as possible. In this study, these models complement each other by using different assumptions and initial conditions to provide predictions of population growth and spatial expansion of pests over time, thus providing a more comprehensive assessment of potential spread. Together these models allow a more profound and better informed interpretation of spread and invasion risks than a single model could do, leading to a more robust basis for exploring invasion scenarios within a PRA.

The key difficulty in using these models in PRA is estimating the parameters. There is no universal method for estimation since this depends on the available data. As shown for *D. virgifera virgifera*, values of parameters are not directly published in the literature, and a good understanding of the parameters’ meaning in the context of the model is needed to extract the required values from data. It would be of great help to risk assessors if a database were developed listing parameter values for example species that might then guide parameterization for species with similar population dynamics and dispersal traits. The shortlist of seven case study species in the current paper provides a beginning for such a list.

The sensitivity analysis shows that is extremely important to obtain correct estimates of the parameters. The relative rate of increase (*r*; model A) is the most sensitive parameter (Tables 2, 3) and needs very careful estimation. Although model D has the largest number of parameters, its global sensitivity was not considerably higher (Table 3). Parameters associated with long distance dispersal are known to affect the asymptotic invasion wave speed and have a great impact on the potential spread [68]–[69]. However, in model D, the shape parameter ν, governing the proportion of long distance dispersers, does not have the strongest effect on the invaded area (Table 2). Because of the tight interaction between growth and dispersal processes, the parameters associated with dispersal (*u* and ν) mostly affected the overall population density (at least for the range of values tested in this study).

A key requirement for using models for prediction is that their validity for this purpose should be demonstrated. This validation can be divided into predictive validity and structural validity. The key challenge for predictive validity is the availability of data. Validation requires that a model is first parameterized with data and then tested with independent data (e.g. [70]). Due to the scarcity of usable data, especially for predictive validation in the context of PRAs, we have not attempted such a validation in this study. Validation therefore mainly results from structural validity: all the models rely on or derive from well-established models with a strong theoretical basis (such as logistic growth, diffusion model or dispersal kernel). Comparing the model outputs also give an indirect indication of their validity. This detailed comparison of the models was not undertaken in this study, except in the user evaluation, which can be considered to be a “soft” and non-quantitative comparison. In ecological modelling, different models can be proposed for a process, and the best model for the data selected. A sophisticated and satisfying way for model selection is the use of likelihood to assess model fit and penalize models for the number of parameters, e.g. using Akaike’s Information Criterion [71], [72]. Unfortunately, the application of such a formal model selection framework is problematic for comparing the four models proposed in this study because the models have different outputs (two simulate occupancy and two simulate density), and they have different initial conditions: one spatially random (model A); one spatially implicit (model C), and two initialized at a point (models B and D). These differences make a formal comparison of model outputs meaningless. The key issue is whether the model is “fit for purpose” and this depends critically, not only on model prediction quality, but as much on the requirements of the end user. We therefore conducted an expert elicitation to assess user opinion as to the question of whether models are potentially useful in PRA practice. Caution is needed to interpret the low number of assessments (only 8), but, overall, the assessment is a positive one. It was frequently stated that PRA analysts would need greater guidance in using the models. Further model testing and building a database of case studies could be very useful for providing guidance. The usability of these models in the PRA context is shown not only by the experts’ testing and feedback but also by the availability of a decision support scheme (DSS) on quantitative spread modelling integrated in a generic PRA DSS (see [73]).

Application of the spread models should take account of technical issues that relate to the spatial resolution of the grid cells. The size of the cells should be small enough to avoid calculation errors but large enough to avoid unreasonably long time durations of simulations (especially for model D). The models were implemented in the statistical language R [74] and two versions of the code have been written: a decimal degree version (which simulates the spread over a regular grid in decimal degrees) and a metric version (which simulates the spread over a regular 10 km grid in the European system projection LAEA). An advantage of the first version is that it is broadly applicable to any part of the world while an advantage of the second version is that it generates maps at the European scale that can be more easily combined with other risk maps [36].

The models presented currently use the outputs of a CLIMEX model to define the area of potential establishment and the population growth. Adapting the spread models to another bioclimatic model such as those available in NAPPFAST [75] is straightforward in principle as long as the bioclimatic model can provide similar information, in particular indices with interpretations similar to the growth and eco-climatic indices of CLIMEX, where the growth index characterizes the potential for population growth during the favourable season(s) while the eco-climatic index integrates this potential for population growth with stresses during unfavourable seasons [38].

The four spread models presented here represent a selection from the potential range of techniques that could be applied. It is possible to explore other modelling techniques. For example, the difficulties the experts had in using model A were confounded by the need for spatially explicit data on the value of assets at risk. In addressing this issue, it is possible to abandon the need for spatially explicit data for the value of assets at risk, and simply combine the spread function in model A (simplicity in parameterisation) with a total asset value figure as a means of estimating the intermediate scenario costs through time. Similarly, it is possible to apply the other spatially implicit model (C) to ordinated assets to assess the best and worst case scenarios. Ultimately, the choice of modelling method should depend on the PRA question at hand (spread or impact?), the availability of data, and the preference of the risk assessor for more or less detail in the modelling approach.

While several biologically based models have been developed to study the invasion processes of exotic pests, few are simple and general enough to be applicable in the context of PRA. We should however be wary of employing techniques that are unnecessarily complex, especially when there are difficulties in selecting reliable values for sensitive parameters (Table 5), and when there have been so few examples of spread models validated with independent data [31]–[32].

Carrasco [31] developed a modelling framework for pest invasion that includes a phenological model as well as a dispersal kernel. This model was applied to two insect species and one bacterium. The model has 14 parameters [76]. This large number of parameters is likely to render the parameterization in a PRA context too laborious and challenging except for very well-known pests.

Pitt [15] developed a GIS based modelling environment for assessing pest invasions, named Modular Dispersal in GIS (MDiG - http://fruitionnz.com/mdig/). The system combines four modules: (1) a growth module which describes birth, death and density dependent processes, (2) a local contiguous dispersal model for a specified proportion of individuals, (3) a Poisson distribution to generate dispersal events and dispersal kernels to determine the distance travelled, and (4) a survival probability based on suitability or survival maps. MDiG has been applied to spread of the Argentine ant, *Linepithema humile* (Mayr) [50] and to the spread of butterfly bush, *Buddleja davidii* [32]. MDiG was not designed to assist a risk assessor in a PRA.

Waage et al. [25] combined spread and economic models to simulate the potential impact of pests and estimate the benefits and costs of government action. The spread model consists of a reaction-diffusion model combined with a model that generates satellite populations. The spread pattern described by this model is spatially implicit, but nonetheless has a strong resemblance to the most sophisticated model in the suite of models described in the current paper, model D. As is the case for our model D, the framework of Waage et al. [25] is challenging to apply in many instances, due to lack of data. Nevertheless, these authors reported spread patterns for a wide variety of taxa, including terrestrial invertebrates and plants, plant diseases, vertebrates, animal diseases and aquatic species. In actuality, however, for many terrestrial invertebrates and plants reported in this study, only the observed radial expansion rate is known and there is no information available about other parameters of this model such as the population’s intrinsic growth rate, the diffusion coefficient, the total area occupied, or the rate of satellite generation. In such a case, it may be advisable to use a simpler model, such as one of the three models A, B, or C, proposed in this paper [71]. The small number of parameters and the conceptual simplicity of these models may assist in making the parameterisation and the results more robust and credible [77]. Further work is needed to test these ideas, and conduct the work that is required to collect the data and validate models. We believe that after sufficient testing and development of expertise, some of these models may be used in the future not only for making PRAs more quantitative, transparent and reliable but also to help target surveillance and management measures more accurately to areas at greatest risk of invasion.

## Supporting Information

Materials S1 **Tutorial on the generic spread models.**
(DOC)

Materials S2 **Case studies.**
(DOC)
