# Supplementary material for: A Suite of Models to Support the Quantitative Assessment of Spread in Pest Risk Analysis
Source: PLoS One. 2012 Oct 9;7(10):e43366. doi: 10.1371/journal.pone.0043366 (PMC3467266; doi:10.1371/journal.pone.0043366)
Supplement: Materials S1 — Tutorial on the generic spread models. (DOC) [file pone.0043366.s001.doc]

**S1 Tutorial on the generic spread models**

This document contains supplementary material to the paper "A suite of models to support the quantitative assessment of spread in pest risk analysis" by Robinet C, Kehlenbeck H, Kriticos DJ, Baker RHA, Battisti A, Brunel S, Dupin M, Eyre D, Faccoli M, Ilieva Z, Kenis M, Knight J, Reynaud P, Yart A & van der Werf W.

Note on font color: The main text is in black font. Red font color indicates R code. Blue font color is used for technical comments about the code or the result returned by R.

**Table of contents**

1. Introduction Page 2
2. First steps in the spread module Page 2
3. Description of the models (Decimal Degree version) Page 12
4. Supplement for the metric version Page 37
5. Supplement for the world version Page 46
6. References Page 47

## 1 Introduction

This document is a tutorial for the spread module (a toolbox composed by four generic spread models) accompanying the article “A suite of simple models to support quantitative assessment of spread and impact in pest risk”. The purpose of this supplementary material is to describe in detail how the spread module can be applied using R (<http://www.r-project.org/>; R Development Core Team 2009).

We focus on Europe because this work is part of the EU project PRATIQUE (<https://secure.fera.defra.gov.uk/pratique/index.cfm>; Baker et al. 2009). The metric version was developed to be able to combine these maps of spread to other risk maps (see Baker et al. 2012). Then, the spread module was extended to be applicable to any part of the world. Consequently, several versions are available: (1) decimal degree version for Europe, (2) metric version for Europe, and (3) decimal degree for any part of the world. Illustration in the article was done using the first version but all these versions are described here.

Note that the names of the parameters were initially different in the code. In this tutorial, we use the names of the parameters given in the code. To facilitate the understanding, the correspondence between the names is given in tables in section 3.

## 2 First steps in the spread module

This tutorial is associated with the R SpreadModule code version 21 (May 30th, 2011) for the decimal degree version (DD version), and version 8 (May 30th, 2011) for the metric version.

## 2.1 Required datasets

The spread module is part of a generic integrated model. It requires several inputs:

1. **Climatic suitability (obligatory) (“ClimexOutput.txt”)**

Two variables are needed:

- a variable indicating whether the species can potentially establish (values from 0 to 100, or 0 to1)
- a variable indicating how the populations can grow (values from 0 to 100).

In the spread models we have used two outputs from CLIMEX respectively: the ecoclimatic index (**EI**) and growth index (**GI**). The EI indicates the favourability of the climate for long-term survival of the species (from 0 if not favourable to 100 in case of ideal conditions) and GI indicates the overall potential for population growth (see Sutherst et al. 2007 for further details, <http://www.hearne.com.au/attachments/ClimexUserGuide3.pdf>).

Note that in the spread module:

- GI is rescaled so that the max(GI) over the area of interest = 100.
- it is possible to use other datasets than these CLIMEX indexes as long as the range of values and the meaning are the same.

1. **Habitat distribution (optional) (“habitat”)**
   A raster indicating the habitat distribution (based on hosts, soil or a combination of both) can be used (values from 0 to 100, or 0/1). In this case, the species cannot disperse in areas where its habitat is absent. In practice, we assume that EI=0 and GI=0 at these locations.
2. **Economic data (optional; just for one model) (“econ”)**
   A raster indicating the economic value (€ / km²) represented by the host can be used. This dataset is required for only one model (model A). If the units are different, e.g., € /ha, it is possible to convert them by directly applying a multiplicative factor (*mult*) (see the following chapter for more details). These data should already take into account the proportion of land covered by the host. If not, you can assume that a given proportion of the area within the host distribution is actually covered by the host. In this case, you should also use the multiplicative factor to ensure that the data represent the economic value of the host per km^2^.

Note that:

- - The **spatial resolution** of the spread module is the resolution of the climatic suitability dataset (except for the metric version, see section 4). This dataset should be composed of locations (defined by their latitude and longitude in **decimal degrees**) and values for EI and GI at these points. In this guide, we will refer either to the **locations** or to the **cells** (rectangles centred at these points), with the ensemble of all the cells covering the whole study area.
  - **Raster files** can be easily exported from GIS software. Be sure that the coordinate system is the same as in CLIMEX (decimal degrees, WGS 1984). In ArcGIS, for instance, select the required layer, then click right on the mouse, select “Data”, then “Export data” and choose the GRID format. Two folders are generated: one folder with the name you entered (the folder you need) and another one called “info” (not needed).

🡪 If you want to include habitat distribution in the models, you should:

- export this dataset as previously described and call this exported file **“habitat”**
- copy and paste the raster for habitat distribution (only the folder “habitat”) into the spread module folder

🡪 If you want to apply the model associated with economic values, you should:

- export this dataset as previously described and call this exported file **“econ”**
- copy and paste the raster for economic values (only the folder “econ”) into the spread module folder

Note that, in the DD version, habitat and economic can be in any raster format (GRID but also .tif or .asc for instance).

## 2.2 Procedure to get the required files

## 2.2.1 CLIMEX file

The spread module needs outputs from CLIMEX (the ecoclimatic index EI and the growth index GI).

- If no CLIMEX model is available, then it is not possible to apply the spread module (unless another bioclimatic model can provide similar information).
- If a specific CLIMEX model is available, then consider the following instructions.

Use CLIMEX[[1]](#footnote-2) compare locations (1 species) with **grid data**, for the area of interest (e.g., Europe). **Do not use the CLIMEX grid data for the world** because GI will be rescaled over the world instead of rescaled over the PRA area, inducing some errors, and, due to the amount of points, the code may crash for some models. Then, export the CLIMEX file:

- click on the “Run” button
- click on the “Save to file” button, then select “new”
- select the model variables: Latitude, Longitude, EI, GI (Comma-delimited), and “Save” (Fig. 1a)
- find the exported file in the folder “models” of CLIMEX, copy this .csv file and paste it in the spread module folder of your study species

Then you have two options:

Option1: you directly use this file. In this case:

- rename this csv file: “**ClimexOutput.csv**”

Option 2: you want to use a reworked file, providing only the coordinates, EI and GI. This option is useful when you want to work on these data before applying the spread module. In this case:

- open this file with Excel (all the data are in the first column) (Fig. 1b)
- select the whole first column (A) and go to “Data”, “Convert”, then select “Delimited”, and “Comma” for the separator. *The preview already shows you the result. Check that all the data appear correctly*. *Decimals should be written with dots and not commas*.
- delete the 4 first columns (Continent, Country, State, Location) and keep only **Latitude, Longitude, EI and GI** columns. ***You must always keep them in this order****,* ***otherwise significant errors will occur. If your columns are not in this order, change them.***
- delete the first empty lines and the header line (Fig. 1c)
- **save as a .txt file, but use either MS-DOS (.txt) or ANSI (.txt).**
- call this file: “**ClimexOutput.txt”** (you must respect capital letters) (Fig. 1d). This is the .txt file which will be imported in R (these points will define the grid cells).

(a)
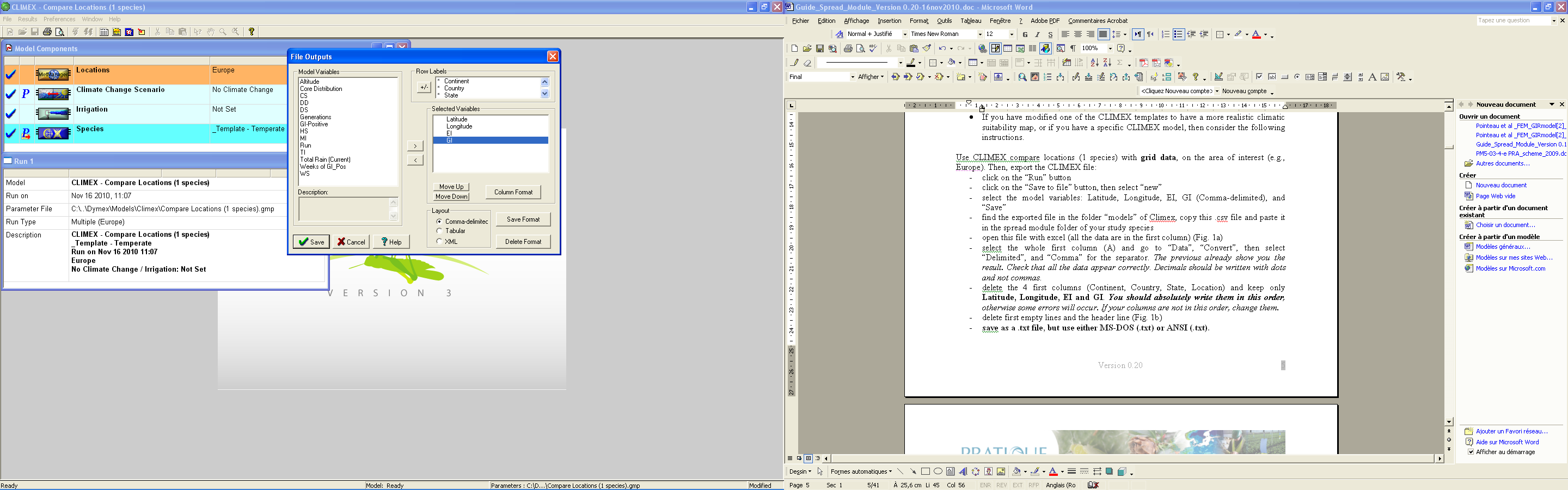


(b)
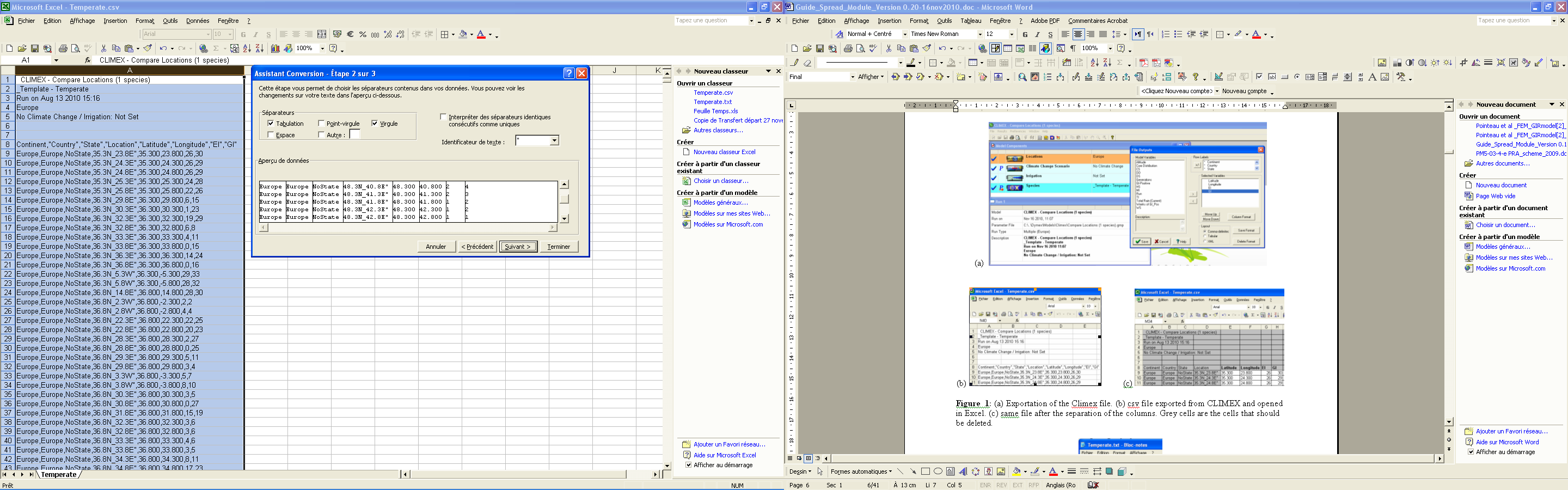


(c)
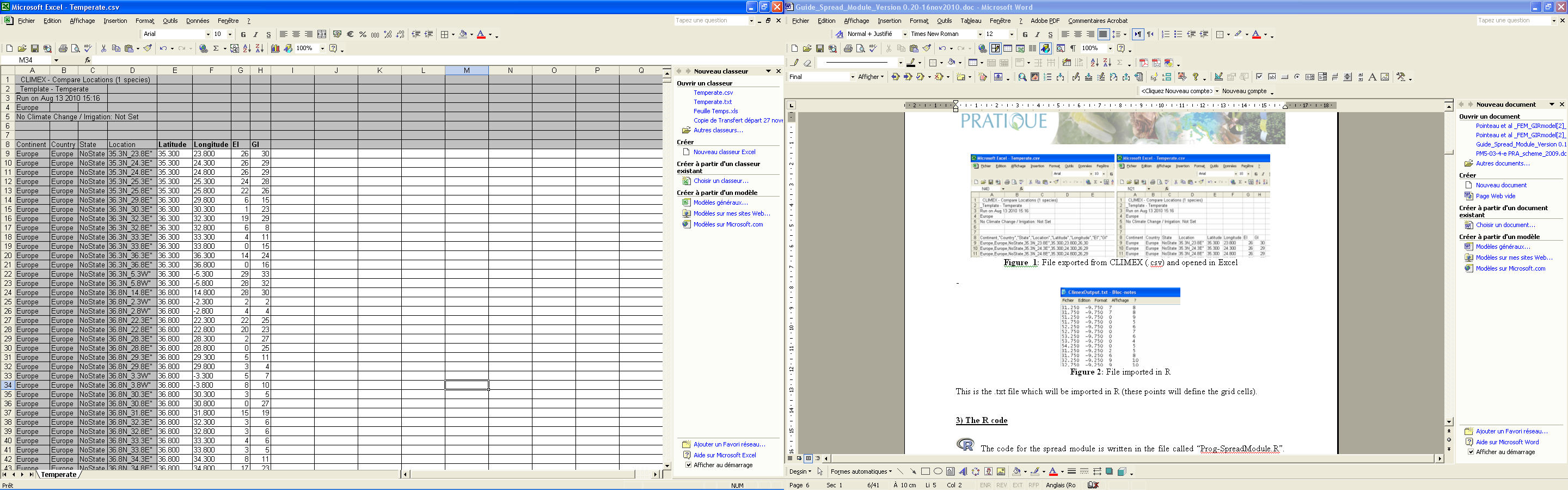
 (d)
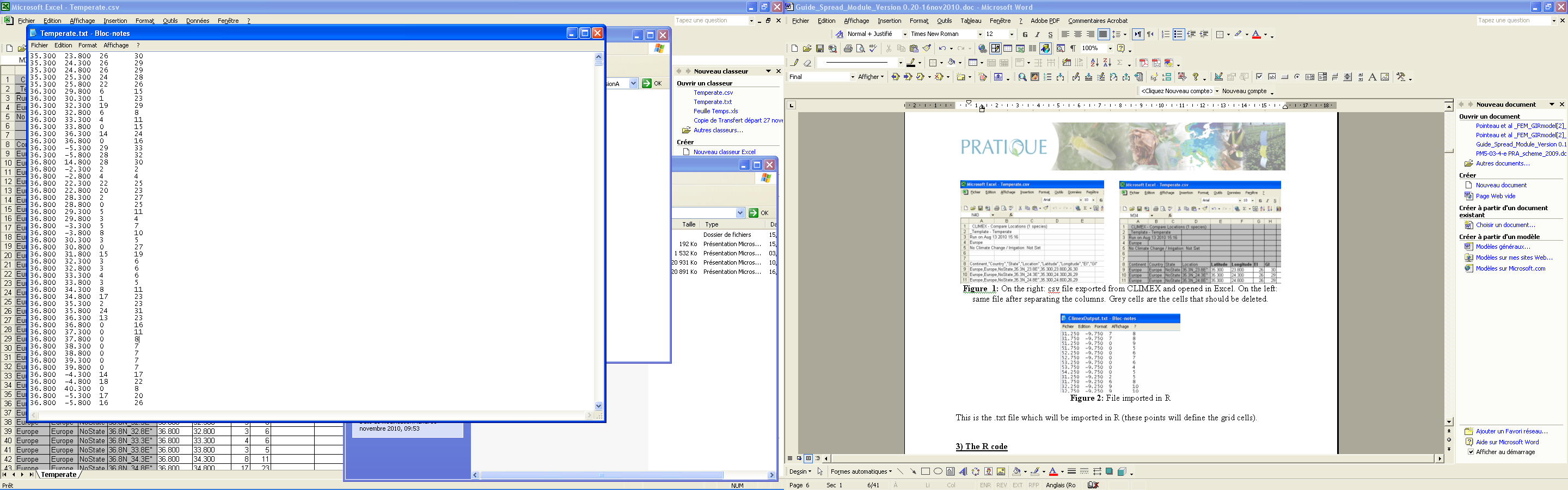


**Figure 2-1:** (a) Exporting the CLIMEX file. (b) csv file exported from CLIMEX and opened in Excel and the procedure to separate the columns. (c) the same file after the separation of the columns. Grey cells are the cells that should be deleted. (d) txt file that should be imported into R.

## 2.2.2 Habitat and economic files

- If habitat and economic files are already used for mapping endangered areas, then these maps can be exported from MCAS (see Baker et al. 2012).
  - In the metric version of the spread module, these maps can be used directly. Just rename them correctly (“habitat” and “econ”).
  - In the decimal degree version, open R and write:

library(sp)

library(raster)

library(rgdal)

projWGS84="+proj=longlat +ellps=WGS84 +datum=WGS84 +no_defs +towgs84=0,0,0"

projETRS89="+proj=laea +lat_0=52 +lon_0=10 +x_0=4321000 +y_0=3210000 +ellps=GRS80 +units=m +no_defs"

prj=as.character(expression(PROJCS["ETRS_1989_LAEA",GEOGCS["GCS_ETRS_1989",DATUM["D_ETRS_1989",SPHEROID["GRS_1980",6378137.0,298.257222101]],PRIMEM["Greenwich",0.0],UNIT["Degree",0.0174532925199433]],PROJECTION["Lambert_Azimuthal_Equal_Area"],PARAMETER["False_Easting",4321000.0],PARAMETER["False_Northing",3210000.0],PARAMETER["Central_Meridian",10.0],PARAMETER["Latitude_Of_Origin",52.0],UNIT["Meter",1.0]]))

r <- raster(choose.files())
# find your .asc file

projection(r)=projETRS89

plot(r)

mask=raster(xmn=-20, xmx=70, ymn=30, ymx=80, crs=projWGS84)

# here you can adjust the spatial extent you need

res(mask)=0.5

rproj=projectRaster(r,mask)

plot(rproj)

writeRaster(rproj,"name_of_the_export_file.tif",format="GTiff")

# => in this last command, put a name for the output file, either habitat or econ

- If these maps are not available from risk mapping (MCAS), then the risk assessor can obtain host distribution data for instance from the McGill University (Canada) website:

<http://www.geog.mcgill.ca/landuse/pub/Data/175crops2000/ArcASCII-Zip/>
If the host species is found there, the risk assessor should download the corresponding file (either “harea”, the percentage of land covered by the species within the cell, or “yield”, the production in tons per ha). Then, unzip the file, open the .asc file with a text editor (notepad for instance), delete the first blanks at the beginning of the 7 first lines (before “ncols”, … and before the first value) and save the changes. Then open R and:

- in the metric version, write:

library(sp)

library(raster)

projWGS84="+proj=longlat +ellps=WGS84 +datum=WGS84 +no_defs +towgs84=0,0,0"

projETRS89="+proj=laea +lat_0=52 +lon_0=10 +x_0=4321000 +y_0=3210000 +ellps=GRS80 +units=m +no_defs"

prj=as.character(expression(PROJCS["ETRS_1989_LAEA",GEOGCS["GCS_ETRS_1989",DATUM["D_ETRS_1989",SPHEROID["GRS_1980",6378137.0,298.257222101]],PRIMEM["Greenwich",0.0],UNIT["Degree",0.0174532925199433]],PROJECTION["Lambert_Azimuthal_Equal_Area"],PARAMETER["False_Easting",4321000.0],PARAMETER["False_Northing",3210000.0],PARAMETER["Central_Meridian",10.0],PARAMETER["Latitude_Of_Origin",52.0],UNIT["Meter",1.0]]))

r = raster(choose.files())

# find your .asc file

mask=raster(nrows=500, ncols=450, xmn=2500000, xmx=7500000, ymn=1000000, ymx=5500000, crs=projETRS89)

res(mask)=10000

projection(r)=projWGS84

q = projectRaster(r,mask)

res(q) = 10000

ymin(q) = 1000000

ymax(q) = 5500000

xmin(q) = 2500000

xmax(q) = 7500000

plot(q)

writeRaster(q,"name_of_the_export_file.tif",format="GTiff")

# => in this last command, put a name for the output file, either habitat or econ

- in the decimal degree version, write:

library(sp)

library(raster)

r = raster(choose.files())

# find your .asc file

r2 = aggregate(r,fact=6,method="bilinear")

# to have a resolution of 0.5° (30 min = 6 * 5 min of the origin file)

# it usually takes a while for the calculation, be patient

res(r2) = 0.5

ymin(r2) = -90

ymax(r2) = 90

xmin(r2) = -180

xmax(r2) = 180

projWGS84="+proj=longlat +ellps=WGS84 +datum=WGS84 +no_defs +towgs84=0,0,0"

mask=raster(xmn=-20, xmx=70, ymn=30, ymx=80, crs=projWGS84)

# here you can adjust the spatial extent you need

res(mask)=0.5

q = crop(r2,mask)

plot(q)

writeRaster(q,"name_of_the_export_file.tif",format="GTiff")

# => in this last command, put a name for the output file, either habitat or econ

- If these maps are available neither from MCAS nor from McGill University, risk assessors could find them from other sources, but in this case, they need to be able to convert them into the correct format since we cannot provide a universal procedure here.

*Note for the economic file*: in the McGill university database, it is possible to derive an economic file. You should make the following calculation: 100 * yield [ton/ha] * area [% covered by the species] to obtain the number of tonnes per km² of land. Then, for model A, you can use this file and attribute a constant economic value for each ton (*mult*).

## 2.3 The R code

The code for the spread module is written in a file called “Prog-SpreadModule.R”. You can load this code with the statistical language software R. It is a free software program you can download at: [http://www.r-project.org](http://www.r-project.org/) (click on “CRAN” on the left column, select the closest location to you, then consider only the part at the top, and select for instance “Windows”, then “base”, and “Download R x.xx.x for Windows” – versions 2.10.1 and 2.11.1 were used for the testing).

### 2.3.1 Required files for the spread module

The following files are provided for the spread module:

| **File name** | **Description** |
| --- | --- |
| SpreadModule.RData | A blank R file where the simulations should be done |
| Prog-SpreadModule.R | The code you should load in R |
| europeL.txt | Countries’ border required for the maps |
| elev.asc | Elevation raster (meters above sea level) |
| raster_1.3-11.zip sp_0.9-66.zip rgdal_0.6-28.zip | Libraries needed by R, also available on the R project website. You should install them only once and then simply call them each time you open R. See section 3.2. |

The following files should be supplied by the end user:

| **File name** | **Description** |
| --- | --- |
| ClimexOutput.txt or.csv | Climatic suitability (derived from CLIMEX) |
| habitat | Raster file of the habitat distribution (host, soil, …) |
| econ | Raster file for the LG-Econ model only |
| presence.txt | Entry points, see section 3.3.3.3, dispersal kernel models |

The first file (ClimexOutput) is obligatory and the others optional.

CAUTION: All these files should be placed in the same folder. If you are assessing several species, you should create several folders and duplicate non-specific files in these folders.

### 2.3.2 To start with the spread module

1) Open the SpreadModule.RData file in the spread module folder of your study species (do not use the R icon on the desktop otherwise you will have to change the working directory where R works).

2) The R code used for the spread module calls some raster files, e.g. to take into account the maximum elevation limit for the species occurrence, host distribution, or economic value. Therefore, you should load some specific libraries. If they are already installed on your computer, go to step 3. If not, go to “Packages”, then “load the package file from a zip file”, and select “sp_0.9-66.zip”. Do the same thing for “raster_1.3-11.zip” and “rgdal_0.6-28.zip”.

3) write in the R console:

> library(sp)

> library(raster)

> library(rgdal)

4) say if you have a direct output from CLIMEX (.csv, option1):

> climexcsv = T

or if not (you should provide a re-worked .txt file as described previously, option 2)

> climexcsv = F

5) give the elevation limit (for instance 1000 m hereafter)

> elevmax = 1000

or, if you do not want to have an elevation limit, write:

> elevmax = F

6) Then, if you have a raster file for the habitat distribution and want to take it into account, write:

> habitatfile = T

If this is not the case, write:

> habitatfile = F

7) You should tell the raster format of the habitat file (if used). If it is a tif format, then you should write:

> habitatformat=”.tif”

If it is an asc format, then you should write:

> habitatformat=”.asc”

If it is a GRID format (arcGIS specific format), a folder with many files will be generated. In this case, you should write:

> habitatformat=NULL

8) **LOAD THE R CODE**: click on “File”, “open a R source code”, find the file Prog-SpreadModule.R, open. It takes a few seconds (be patient). Then the information concerning the spread module is given. If you want to display this information later, just type:

> printinfo()
The prompt > appears just after this and then you can type the commands to run the models (see next sections for the command lines that need to be typed to call the models).

**If the habitat distribution is provided, then the CLIMEX EI and GI values are considered to be 0 where the host is absent. If an elevation limit is provided, then, above the limit, EI and GI values are also considered to be 0.**

You can plot several maps: the risk area map (where EI>0 – area of potential establishment), the rescaled GI, and the habitat (if available) using the following commands respectively:

> plotRA()

> plotGI()

> plothabitat()

The map legend is given on the R console when calling the functions.

If you have the coordinates of infested locations and you want to know the number of infested cells, you should create a new text file and enter longitude (1st column) and latitude (2nd column) in decimal degrees, and save it as a text file in your working directory. The first line should not give the name of the columns but directly the values. Then, you can use the following function:

> pointtocell(“name_of_your_file.txt”)

to obtain the number of infested cells.

### 2.3.3 Some basic notes about the use of R

- You can save the workspace. In this case R will keep in memory all the functions and the values saved in variable names. If you open this R file later, you will just need to call the libraries and you can continue working as if R has not been closed.
- If you want to save the commands you have written in the R console, you should save the history (use the file menu). Then you can open this history file (with a text editor for instance) and find all the commands you typed in R.
- When you call a function (see section 3), you can change the order of the parameters. If you do not write a given parameter with its value, then R will take the default value (arbitrary but constant value) for this parameter.
- T means true, F means false, NA means ”Not a Number”, NULL means no value.
- R differentiates lower case and capital letters, therefore you should always include appropriate capital letters otherwise R will not recognise the names of variables or files.
- The decimal is a dot and not a comma.

## 2.4 Quick launch

If you want to test the models rapidly, follow the steps below, copy the commands and paste them into the R console. They are given as examples; you should change them according to the specific case you are interested in. This is a summary of the commands. For more details, read the following sections.

1- Obtain the CLIMEX dataset (or other climatic suitability datasets)
2- Obtain the habitat distribution if required

3- Obtain economic data if required (for one model only)

and place them in the spread module folder.

R commands:

| library(sp)  library(raster)  library(rgdal)  climexcsv = T  elevmax = F  habitatfile = T  habitatformat= ".tif"  ***# LOAD THE R CODE*** *(do not forget this important step)*  # model A:  res = lgecon(N0=0.1,r=2,t=5, econraster=F,mult=1,sim=10) # model B:  res = radial(RR=80,t=5,coord=c(-5,39))  # hybrid model:  res = radialrand(RR=200,t=4,N0=0.1,r=1,coord=c(30,47))  # model C:  res = slg(N0=0.1,lmax=10,t=5)  # model D:  res = dispk(N0=1, t=10, lmax=40, p=50, u=100, presencefile=F,nentry=1) |
| --- |

Note that you should type: zero and not the capital letter O when writing N0; little L and not the number 1 when writing lmax.

## 2.5 WARNING

These models should be considered as ***scenarios***. The outputs of the models are not predictions to be trusted blindly. These predictions depend closely on the validity of the assumptions and parameter estimates. **You**, the user, are responsible for these estimates and a prudent interpretation of the model results. The models developed for the spread module are tools for risk assessors to obtain a spatial representation of the potential spread of the study species. To some extent, they are designed to summarize on a map the basic biological knowledge available on spread and allow the user to test several values for the parameters to mimic uncertainty and see how this affects the potential spread. None of these model scenarios will give the same results because their assumptions are different. It is thus recommended to test several models to obtain a range of possible outcomes, and eventually to select the most suitable models according to the species spread behaviour, data availability and the degree of confidence that you place on the model assumptions and the parameter estimates.

# **3 Description of the models (Decimal Degree version)**

This tutorial is associated with the R SpreadModule code version 21 (May 30^th^, 2011) for this DD version.

This section describes the models and the parameters. Risk assessors should first collect data to estimate the value of the parameters. In this section, the models and the meaning of the parameters are described but no guidance on how to estimate the parameters is provided here because there is no universal method.

## 3.1 How to map uncertainty

It is generally difficult to find a single value for the parameters, because there is very often a range of possible values or a range of values resulting from this uncertainty. In this R code program, you can only enter a single value for each parameter each time you run the model, but it is advisable to test several values, one by one, within the possible range to explore the range of potential outcomes for a chosen model.

More precisely, ***to illustrate the uncertainty***, you should plot ***three maps***: the best case, the most likely case and the worst case, taking, in turn, the parameter values (within their potential range) corresponding to the slowest spread, the most likely spread and the most rapid spread simulations. If the spread module is applied by only one expert, then we recommend testing the most likely values - 10% and + 10% for all the parameters to obtain the best and worst cases. If there are large uncertainties in the estimates, you can use +/- 50% instead of 10%. If the spread module is applied by a group of experts, then each expert should provide his/her best estimates and the most likely case will then be provided by the mean, the best case by the minimum and the worst case by the maximum parameter value.

## 3.2 Temporal process models

In these models, we ignore the distance between the cells and the dispersal from one cell to another. There is no link between the cells. Here we make no assumption about the initial distribution (e.g. entry points).

3.2.1 Model C: Population dynamics model (SLG) *“Simple Logistic Growth model” or “Temporal spread within cells”*

We assume that the same initial population density *N_0_* (%) is introduced in all suitable cells and then we simulate the “spread” or more precisely the population growth within each cell according to a logistic function. The output map will show all the areas which are the most suitable for population growth within the area of potential establishment. This model provides a population density for the worst case (i.e. all suitable cells are invaded) and the output is particularly useful for indicating areas where the highest population densities are likely to occur, a key factor to take into account in economic impact assessments.

**Table 1a**: List of parameters for model C

| *Parameter* | *Unit* | *Range* | *Default value* | *Type of parameter* | *Description* |
| --- | --- | --- | --- | --- | --- |
| ***N_0_*** | % | 0-100 | 1 | Biological or user choice | Population abundance for all suitable cells at time t = 0 expressed as a percentage of the maximum abundance (carrying capacity K) |
| ***λ*_max_** |  | ≥1 | 7 | Biological | Maximum year to year multiplication factor (“finite growth rate”) that a population could achieve under optimal conditions assuming unlimited space |
| ***t*** | year | ≥1 | 1 | End user choice | The time horizon for the simulations (year) to calculate the spread |
| ***movie*** | Logical | T or F | F | Graphic | If *movie* = T (true), then you will see the map for each time from 1 to t, clicking on the figure each time. If F (false), then only the map at time t will be plotted |
| ***export*** | Logical | T or F | F | End user choice | If *export* = T (true), then the output will be exported in a tif file. |
| ***name*** | Character |  | SLG-DD-output | End user choice | If *export* = T (true), then the name given here will be the name of the exported tif file. |

Note that the parameter *λ*_max_ is written lmax in R.

We apply a logistic function to calculate the population abundance (%) in each suitable cell at time *t*:

$N_{t}=\frac{N_{0}\exp\left(r t \right)}{1+N_{0}(\exp\left(r t\right)-1)/100}$ (Eq 1)

with $r=GI*\ln(\lambda_{\max})/100$ (Eq 2)

The growth index (GI) provided by CLIMEX can potentially range from 0 to 100 and provides an indication of the potential growth based on climate. However, in many cases, GI does not reach 100. To adjust this parameter to the “real” population growth observed in the PRA area, this parameter was rescaled to range effectively from 0 to 100 and the specific intrinsic rate of natural increase *r* is equal to ln(λ_max_) where *GI*=100.

**Figure 3-1a**: Curve used in the simple logistic growth

model to calculate the population abundance in grid cells.

**
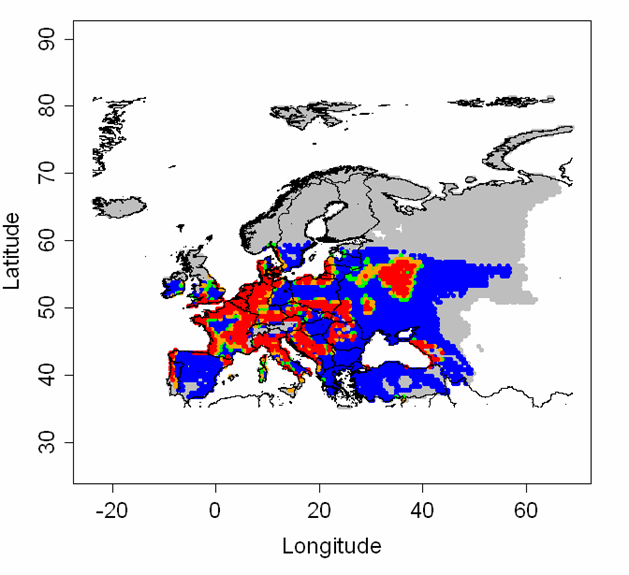
**

**Figure 3-1b:** Example of an output for the simple logistic growth model. Grey dots represent cells where EI = 0, blue dots where 0 < *N*_t_ ≤ 25, green dots where 25 < *N*_t_ ≤ 50, orange dots where 50 < *N*_t_ ≤ 75, and red dots where 75 < *N*_t_ ≤ 100. This example was generated for *t* = 20, *N*_0_ = 5*10^(-5), λ_max_=40.

In R, write:

res = slg()

and press enter to run this model with default values, or if you want to use other parameter values, you can write for instance:

res = slg(N0=0.1,lmax=10,t=5)

You will see the result on a figure. Grey dots represent cells where EI = 0, blue dots where 0 < *N*_t_ ≤ 25, green dots where 25 < *N*_t_ ≤ 50, orange dots where 50 < *N*_t_ ≤ 75, and red dots where 75 < *N*_t_ ≤ 100. The following commands allow you to produce several results:

res$slg the corresponding population abundance (%)

res$ntot the total number of cells considered in the model

res$nRA the number of cells included in the risk area (EI>0)

res$sum0 the number of cells where *N*_t_ = 0

res$sum25 the number of cells where 0< *N*_t_ ≤ 25

res$sum50 the number of cells where 25 < *N*_t_ ≤ 50

res$sum75 the number of cells where 50 < *N*_t_ ≤ 75

res$sum100 the number of cells where 75 < *N*_t_ ≤ 100

**To export the output:**

res = slg(N0=0.1,lmax=10,t=5,export=T,name=”SLGresult”)

In this case, the output will be exported in a tif file called SLGresult.tif in the working directory. The projection of this raster file is Lambert Azimuthal Equal Area (ETRS 1989 LAEA), a European metric projection used in risk mapping (see WP3 and the metric supplement, section 3.4). This file can be combined to other risk maps using MCAS (see WP3).

We do not recommend exporting the file at first because it takes a long time to create the raster file and export it, and problems arise if you export the file several times with the same name. The best approach is to play with the model first and then, when the results are satisfactory and you want to work on this output, you can export it (as mentioned above).

**Table 1b. Correspondence between the names of the parameters for model C**

| *Name in this tutorial and in the code* | *Name in the article* | *Description* |
| --- | --- | --- |
| ***N_0_*** | ***p_0_*** | Population abundance for all suitable cells at time t = 0 expressed as a percentage of the maximum abundance (carrying capacity) |
| ***K*** | ***P_max_*** | Carrying capacity |
| ***λ*_max_** | ***λ*_max_** | Maximum year to year multiplication factor |
| ***t*** | ***t*** | The time horizon for the simulations |

3.2.2 Model A: Temporal spread over cells integrated with impact (LG-Econ)  ***“****Logistic Growth model based on Economic values”*

In this model, we use a logistic function to calculate the percentage of the cells invaded at time *t*.

We assume that an initial population abundance *N*_0_ is introduced and then we simulate the invasion based on various scenarios.

- worst case scenario: cells with the highest economic value are invaded first
- best case scenario: cells with the lowest economic value are invaded first
- random case scenario: cells are invaded at random

This model, like the simple logistic growth model, uses logistic growth. However, it is conceptually a very different model. Whilst the logistic equation in the simple logistic growth model represents the S-shaped growth of a population *within each grid cell*, the Logistic growth model based on economic values represents the S-shaped increase in the *number of invaded grid cells* over time. It therefore operates over a much greater scale. This model has the same parameters as the simple logistic growth model, *r* and *N*_0_, however, its interpretation is different. In this case, *r* is the relative rate of increase in the number of invaded grid cells per unit of time. This model is useful to obtain a range of economic impact.

**Table 2a**: List of parameters for model A (LG-Econ)

| *Parameter* | *Unit* | *Range* | *Default value* | *Type of parameter* | *Description* |
| --- | --- | --- | --- | --- | --- |
| ***N_0_*** | % | 0-100 | 1 | Biological or End user choice | Initial percentage of the risk area invaded at time *t*=0 |
| ***r*** | year^-1^ | 0-100 | 1 | Biological | Relative rate of spatial increase per year |
| ***t*** | year | ≥1 | 1 | user choice | The time horizon for the simulations (year) to calculate the spread |
| ***econraster*** | Logical | T or F | F | Data availability | If T, then the code will use the raster “econ” provided by the end-user to obtain the values at required points. If F, then values are either derived from the host distribution if hostraster=T, or chosen at random between 1 and 100 if hostraster=F. |
| ***econformat*** | Character |  | NULL | Data availability | If econraster = T (true), then the format of the economic file should be given. If it is a GRID format, then it should be NULL. If it is another raster format, then write the extension of the file, e.g. “.tif”. |
| ***habitat raster*** | Logical | T or F | F | Data availability | If no economic raster is available, economic values are derived from habitat abundance. |
| ***valperhost*** | €/host abundance | > 0 | NULL | Economic variable | The value (€/host abundance per km²) should be provided if economic values are derived from host abundance. |
| ***mult*** |  | > 0 | 1 | Data availability | Multiplicative factor to convert the values given in the raster file into the required unit: €/ km² |
| ***sim*** |  | ≥1 | 1 | End user choice | Number of replicate simulations for the random case. |
| ***export*** | Logical | T or F | F | End user choice | If *export* = T (true), then the output will be exported in a tif file. |
| ***name*** | Character |  | LGECON-DD-output | End user choice | If *export* = T (true), then the name given here will be the name of the exported tif file. |

In R, write:

res = lgecon()

and press enter to run this model with default values, or if you want to use other parameter values, you can write for instance:

res = lgecon(N0=0.1,r=1,t=5,sim=10)

You will see the results of the 3 cases on a figure. Grey dots represent cells where EI=0, orange dots where EI>0, and red dots invaded cells (among those where EI>0). The following commands allow you to obtain several results:

res$ntot the total number of cells considered in the model

res$nRA the number of cells included in the risk area (EI>0)

res$ninv the number of cells invaded

res$pniche the percentage of cells invaded within the risk area

res$worst the accumulated economic values for the worst case

res$best the accumulated economic values for the best case

res$rand the accumulated economic values for random case
 (min, 1^st^ qu., median, mean, 3^rd^ qu. , and max)

res$econvalues economic values used by the model

**To export the output**:

res = lgecon(N0=0.1,r=1,t=5,sim=10,export=T, name=”LGECONresult”)

In this case, the output for each of the three scenarios will be exported in tif files in the working directory (files called LGECONresult_best, LGECONresult_rand and LGECONresult_worst respectively for the best, random and worst scenarios). The projection of this raster file is Lambert Azimuthal Equal Area (ETRS 1989 LAEA), a European metric projection used in risk mapping (see section 4). This file can be combined to other risk maps using MCAS (see Baker et al. 2012). The value 1 means that the cell is invaded, the value 0 means that the cell is within the area of potential establishment but not invaded, the value -1 means that the cell is not within the area of potential establishment and the value -9999 means that data is missing.

To know how many cells are covered by some points of pest presence, you can use the following function:

pointtocell(“name_of_your_file.txt”)

You should enter the name of the file containing the longitude (column 1) and latitude (column 2) in decimal degrees. This file should be located in the working directory. For each point, the function determines the cell where it is located within the area of potential establishment. If the point is located outside the area of potential establishment, then the corresponding cell is the closest one within this area.


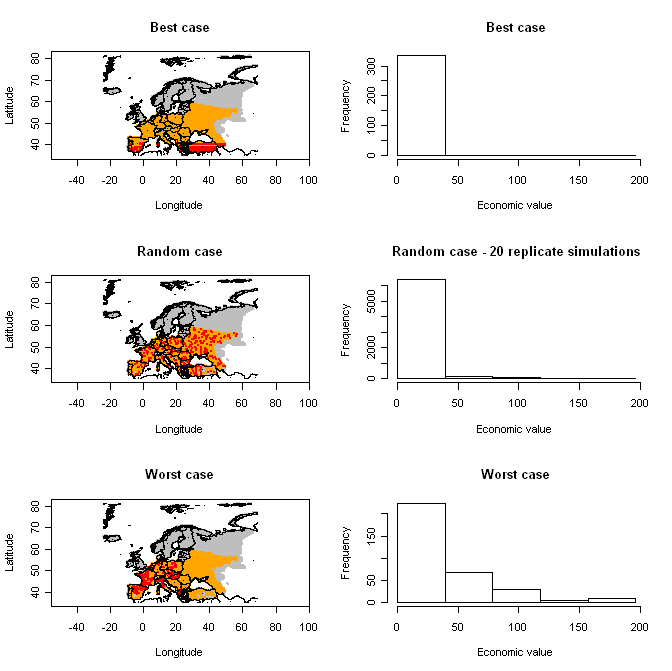
**Figure 3-2:** Example of an output for the logistic growth model based on economic values. Grey dots represent the cells where EI=0, orange dots where EI>0, and red dots the invaded cells. Economic values are expressed in monetary units per raster cell. These are defined by the user in the input file “econ”.

**Table 2b. Correspondence between the names of the parameters for model A**

| *Name in this tutorial and in the code* | *Name in the article* | *Description* |
| --- | --- | --- |
| ***N_0_*** | ***n_0_*** | Initial percentage of the risk area invaded at time *t*=0 |
| ***K*** | ***N_max_*** | Carrying capacity (number of cells within the area of potential establishment) |
| ***r*** | ***r*** | Relative rate of spatial increase per year |
| ***t*** | ***t*** | The time horizon for the simulations |

## 3.3 Spatial process models

In these models, we take into account the entry points and the distance between the cells to describe the dispersal capability of the species.

3.3.1 Model B : Radial range expansion model (RR)

This model determines the potential spread of a species introduced in the PRA area based on a parameter for the radial rate of expansion (see section 5.4 for the recommendations). The model output runs within the area of potential establishment based on climatic suitability (EI>0).

We used the following formula to calculate the distance d in km between two points (x1,y1) and (x2,y2) in decimal degrees:

$$d=\frac{20000}{\pi}\arccos\left(\sin\left(\frac{\pi}{180}y1\right)\sin\left(\frac{\pi}{180}y2\right)+\cos\left(\frac{\pi}{180}y1\right)\cos\left(\frac{\pi}{180}y2\right)\cos\left(\frac{\pi}{180}\left(x2-x1\right)\right)\right)$$

(Eq 3)

For the map projection in a metric system, we chose the origin of the x-axis to be x=20° East, and we used the following formulae:

$\begin{matrix} xkm=\frac{40000}{360}\left(x-longref\right)\cos\left(\frac{\pi}{180}y\right) \\ ykm=\frac{40000}{360}y \end{matrix}$ (Eq 4)

These complicated formulas are needed because the earth is not flat, but a sphere.

**Table 3a**: List of parameters for model B

| *Parameter* | *Unit* | *Range* | *Default value* | *Type of parameter* | *Description* |
| --- | --- | --- | --- | --- | --- |
| ***RR*** | km/yr | > 0 | 20 | Biological | Radial rate of range expansion per year |
| ***t*** | year | ≥1 | 1 | End user choice | The time horizon for the simulations (years) to calculate the spread |
| ***coord*** | Decimal degrees | Similar to the range of ClimexOutput | At random in the risk area | Place of introduction place or user choice (test) | The entry point(s) or a simulation of an introduction at this place. |
| ***figkm*** | Logical | T or F | T | Graphic | If T (true), then *figdd* should be F, and the figure will be in a metric system. |
| ***figdd*** | Logical | T or F | F | Graphic | If T (true), then *figkm* should be F, and the figure will be in a decimal degrees. |
| ***export*** | Logical | T or F | F | End user choice | If *export* = T (true), then the output will be exported in a tif file. |
| ***name*** | Character |  | Radial-DD-output | End user choice | If *export* = T (true), then the name given here will be the name of the exported tif file. |

Note that entry points should be chosen from within the area that is climatically suitable for establishment (EI>0). If the point is outside this area, the code will select the closest point in the suitable area.


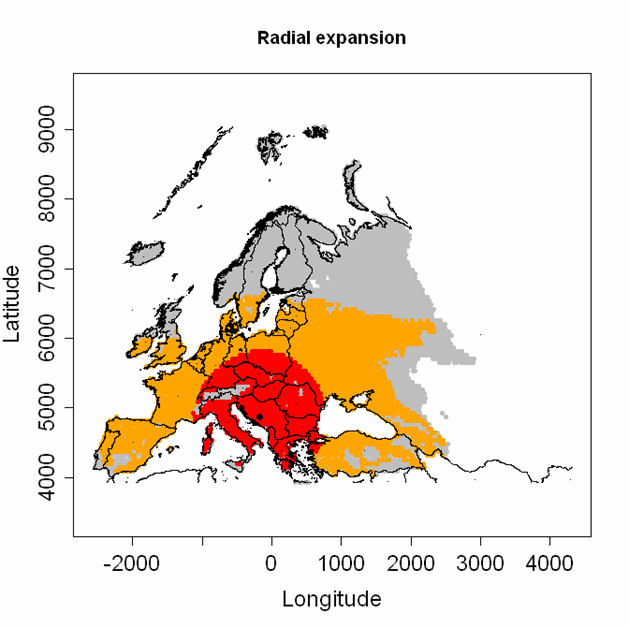


**Figure 3-3:** Radial expansion model for *t* = 16, *RR* = 60 km/year based on an entry point in former Yugoslavia (black dot). Grey dots represent the points where EI = 0, orange dots represent the suitable area (EI>0) not invaded and red dots the invaded cells based on the spread rate given by the user.

In R, write:

res = radial()

and press enter to run this model with default values, or if you want to use other parameter values (e.g. a radial rate of 80 km/year, and a simulation time of 5 years), you can write for instance:

res = radial(RR=80,t=5)

In the previous case, the entry point is selected at random within the area where EI>0. In case, you want to provide the coordinates of this entry point, write:

res = radial(RR=80,t=5,coord=c(-5,39))

The first value in coord is the longitude and the second one the latitude of the entry point in decimal degrees. Note that you can enter several entry points. In this case, write longitude(point1), latitude(point1), longitude(point2),latitude(point2),… as follows:

res = radial(RR=80,t=5,coord=c(-5,39,-6,42))

You will see the result on a figure. Grey dots represent the points where EI=0, orange dots represent suitable area (EI>0) not invaded and red dots invaded cells. The following commands allow you to have several results:

res$radial latitude (col1), longitude (col2), EI (col 3), GI (col 4)
of invaded points

res$ntot the total number of cells considered in the model
res$nRA the number of cells within the risk area (EI>0)

res$ninv the number of cells invaded

res$pniche the percentage of cells invaded within the risk area

**To export the output**:

res = radial(RR=80,t=5,coord=c(-5,39,-6,42),export=T, name=”Radialresult”)

In this case, the output will be exported in a tif file called Radialresult.tif in the working directory. The projection of this raster file is Lambert Azimuthal Equal Area (ETRS 1989 LAEA), a European metric projection used in risk mapping (see WP3 and the metric supplement, section 3.4). This file can be combined to other risk maps using MCAS (see WP3). The value 1 means that the cell is invaded, the value 0 means that the cell is within the area of potential establishment but not invaded, the value -1 means that the cell is not within the area of potential establishment and the value -9999 means that data is missing.

**Table 3b. Correspondence between the names of the parameters for model B**

| *Name in this tutorial and in the code* | *Name in the article* | *Description* |
| --- | --- | --- |
| ***RR*** | ***c*** | Initial percentage of the risk area invaded at time *t*=0 |
| ***t*** | ***t*** | The time horizon for the simulations |

## 3.3 Spatial process models

3.3.2 Hybrid model of logistic growth and radial rate expansion (model A and B)

This model combines a randomised version of the temporal spread over cells model (model A) with the radial expansion model (model B) to take into account the distance from the entry point(s). Since this model is based on the random selection of the cells (in-between the best and worst cases), it does not require economic data inputs. It is therefore an advantage in terms of data availability. Invaded cells are chosen at random within the area given by the radial expansion model which makes the spatial distribution more realistic and credible. The distance and the projection in a metric system are calculated using Eqs 3-4. This model is useful to take into account that the population may not spread over all the area defined by the radial expansion rate. It provides more information than model B.

**Table 4**: List of parameters for this hybrid model

| *Parameter* | *Type or unit* | *Range* | *Default value* | *Type of parameter* | *Description* |
| --- | --- | --- | --- | --- | --- |
| ***N_0_*** | % | 0-100 | 1 | Biological | Initial percentage of the risk area invaded at time *t*=0 |
| ***r*** | year^-1^ | 0-100 | 1 | Biological | Relative rate of spatial increase per year |
| ***RR*** | Km/yr | > 0 | 20 | Biological | Radial rate of range expansion per year |
| ***t*** | year | ≥1 | 1 | End user choice | The time horizon for the simulations (year) to calculate the spread |
| ***coord*** | Decimal degrees | Similar to the range of ClimexOutput | At random in the risk area | Introduction place or end user choice (test) | The entry point(s) or a simulation of an introduction at this place. |
| ***figkm*** | Logical | T or F | T | Graphic | If T (true), then *figdd* should be F, and the figure will be in a metric system. |
| ***figdd*** | Logical | T or F | F | Graphic | If T (true), then *figkm* should be F, and the figure will be in a decimal degrees. |
| ***export*** | Logical | T or F | F | End user choice | If *export* = T (true), then the output will be exported in a tif file. |
| ***name*** | Character |  | Radialrand-DD-output | End user choice | If *export* = T (true), then the name given here will be the name of the exported tif file. |

**
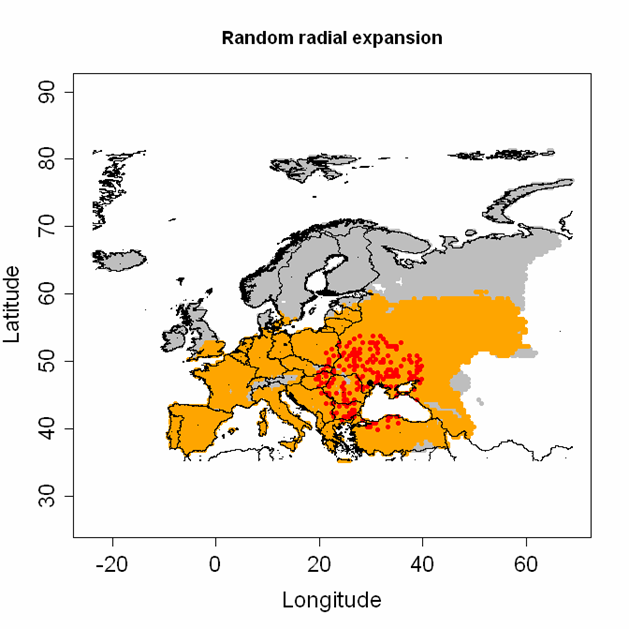
**

**Figure 3-4:** Random radial expansion model for *t* = 4, *RR* = 200, *N*_0_ = 0.1, *r* = 1, *coord* = c(30,47). Grey dots represent the points where EI = 0, orange dots represent suitable area (EI>0) not invaded and red dots invaded cells.

In R, write:

res = radialrand()

and press enter to run this model with default values, or if you want to use other parameter values, you can write for instance:

res = radialrand(RR=200,t=4,N0=0.1,r=1)

In the previous case, the entry point is selected at random within the area where EI>0. In case, you want to provide the coordinates of this entry point, write:

res = radialrand(RR=200,t=4,N0=0.1,r=1,coord=c(30,47))

The first value in coord is the longitude and the second one the latitude of the entry point in decimal degrees. You can enter several entry points. In this case, write longitude(point1), latitude(point1), longitude(point2),latitude(point2),… as follows:

res = radialrand(N0=0.1,r=0.5,RR=80,t=5, coord=c(0,37,5,50))

You will see the result on a figure. Grey dots represent the cells where EI=0, orange dots represent suitable area (EI>0) not invaded and red dots invaded cells. The following commands allow you to display several results:

res$radialrand latitude (col1), longitude (col2), EI (col 3), GI (col 4) of invaded cells

res$ntot the total number of cells considered in the model
res$nRA the number of cells within the risk area (EI>0)

res$ninv the number of cells invaded

res$pniche the percentage of cells invaded within the risk area

**To export the output**:

res = radialrand(N0=0.1,r=0.5,RR=80,t=5, coord=c(0,37,5,50),export=T, name=”Radialrandresult”)

In this case, the output will be exported in a tif file called Radialrandresult.tif in the working directory. The projection of this raster file is Lambert Azimuthal Equal Area (ETRS 1989 LAEA), a European metric projection used in risk mapping (see WP3 and the metric supplement, section 3.4). This file can be combined with other risk maps using MCAS (see WP3). The value 1 means that the cell is invaded, the value 0 means that the cell is within the area of potential establishment but not invaded, the value -1 means that the cell is not within the area of potential establishment and the value -9999 means that data is missing.

3.3.3 Model D: Dispersal kernel models (DK)

These types of model require: a population growth model, a dispersal kernel (2Dt in this case) and the proportion of population engaged in dispersal. Two versions have been developed: a deterministic version where the population spread relies on probabilities, and a stochastic version, where the dispersal distance is chosen at random in the probability distribution and several replicate simulations are required to obtain a representative population spread.

The dispersal kernel in 2 dimensions is given by:

$f\left(r\right)=\frac{1}{u^{2}p\pi}\frac{\Gamma\left(\frac{p+1}{2}\right)}{\Gamma\left(\frac{p-1}{2}\right)}\frac{1}{\left(1+\frac{1}{p}\frac{r^{2}}{u^{2}}\right)^{\frac{p+1}{2}}}$ (Eq 5)

where *r* is the distance between two points calculated with eq 3.

For *p* = 1, this kernel has a Cauchy distribution (thick tail; a large number of individuals disperses at long distance) and for *p*$\to+\infty$, it has a normal distribution (thin tail) (Clark et al. 1999).

To account for the shrinkage due to the earth curvature (the length of the ring at distance *r* is less than that of a ring in the plane at distance *r*), we included a correction and finally used the following formula to calculate the probability to disperse at a distance *r*:

$$k(r)=\frac{r}{20000/\pi}\frac{1}{\sin\left(\frac{r}{20000/\pi}\right)}f(r)$$

To see the shape of the kernel, write:
> plotkp(u=50,p=5,xmax=200,color=”blue”)

and eventually:

> plotkp(u=5,p=5,xmax=200, add=T,color=”red”)

**
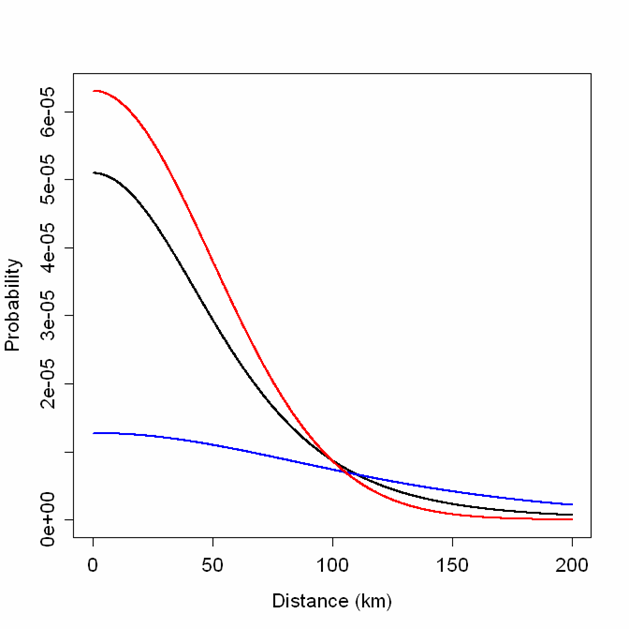
**

**Figure 3.3-5**: Dispersal kernel with
 *p* = 5 and *u* = 50 (black),
 *p* = 100 and *u* = 50 (red),
 *p* = 5 and *u* = 100 (blue).

Depending on the knowledge of the locations and population densities where the species is present in the PRA area the model operates in three ways:

1. the risk assessor knows the points where the species is present and the population density at a given time *t* = 0 (initial condition for the model): the corresponding file should be a text file with three columns. **Column 1 is longitude, column 2 latitude in decimal degrees, and column 3 the population density** (in % of the carrying capacity, between 0 and 100). Note that in this case no formula should be entered in this file. For instance if the population density is 1.7*10^(-7) at time *t* = 0, the risk assessor should enter 1.7e-7 in the file (3^rd^ column). This file should be named “**presence.txt**” and placed in the folder containing the R file “SpreadModule.Rdata”.


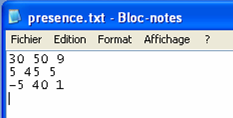


**Figure 3.3-6:** Presence file with three points and initial population densities (9, 5 and 1%). You should press enter at the end of the last line and save it.

1. The risk assessor knows only the points where the species is present and can provide a single arbitrary value *N*_0_ for the population density at these locations before calling the function: the corresponding file should be a text file with two columns. Column 1 is longitude and column 2 latitude in decimal degrees. This file should be named “**presence.txt**” and placed in the folder containing the R file “SpreadModule.Rdata”.
2. The risk assessor does not know the points where the species is present or the species is not present in the PRA area. In this case, no presence file should be provided and the model will randomly select some presence points (the risk assessor can choose the number of presence points with the parameter *nentry*). If the risk assessor wants to test entry at some precise points (e.g. introduction at some ports or airports), he can create a presence file and consider these points as in case (2).

This model cannot be used when the annual spread rate is smaller than the CLIMEX grid resolution.

### 3.3.3.1 Deterministic model

Description of the model:

- the presence file is first transformed into a vector associated with the grid points. This vector contains either false/true values to identify the points where the species is present (in cases 2 and 3) or the population density at each grid point (= 0 when there is no point where the species is present near the grid point, and the mean population density if there is more than one point near the grid point);

- for each time step between 1 and *t*, we select the grid points where EI>0 and the initial points where the species is present as potential source points (to limit the calculations). For each of these potential source points, we calculate the distance from this point to all the grid points, apply the dispersal kernel and integrate this kernel over space (multiplying these dispersal probabilities by the area of the cells). We finally multiply these probabilities by the density of the population engaged in dispersal and accumulate these values. Then, we add non-dispersing individuals. The distance and the projection in a metric system are calculated using Eqs 3-4.

- We assume that individuals die if they disperse into a cell where the EI=0.

- We apply the growth model (logistic model) to this new population distribution.

- This result provides the population expansion after one time step. Then we repeat these calculations until the time step *t*.

**Table 5a**: List of parameters for the deterministic version of model D

| *Parameter* | *Unit* | *Range* | *Default value* | *Type of parameter* | *Description* |
| --- | --- | --- | --- | --- | --- |
| ***N_0_*** | % | 0-100 | NULL | Biological | Population abundance at time t = 0 expressed as a percentage of the maximum population abundance (carrying capacity K). If NULL then the file presence.txt should be provided. |
| ***λ*_max_** |  | ≥1 | 7 | Biological | Maximum year to year multiplication factor (“finite growth rate”) that a population could achieve under optimal conditions assuming unlimited space |
| ***P*** |  | 0-1 | 1 | Biological | Proportion of the population engaged in dispersal |
| ***p*** | Degree of freedom | ≥2 | 50 | Biological | Shape parameter of the 2Dt dispersal kernel (number of degrees of freedom) |
| ***u*** | km | > grid resolution | 100 | Biological | Scale parameter of the 2Dt dispersal kernel |
| ***t*** | year | ≥1 | 1 | End user choice | The time horizon for the simulations (year) to calculate the spread |
| ***presencefile*** | Logical | T or F | T | End user choice | If T (true), a presence file should be provided. If F (false), then provide the number of entry points (*nentry*) to be selected at random in the suitable area and the population density *N_0_*. |
| ***nentry*** |  | ≥1 | NULL | End user choice | The number of entry points to select at random within the risk area (EI>0) if no presence file is provided |
| ***figkm*** | Logical | T or F | T | Graphic | If T (true), then *figdd* should be F, and the figure will be in a metric system |
| ***figdd*** | Logical | T or F | F | Graphic | If T (true), then *figkm* should be F, and the figure will be in a decimal degrees |

In this model, the parameter *P* represents the proportion of individuals engaged in dispersal. Although there is an option to alter the value of this parameter, it is advisable to keep *P*=1 (the default value).


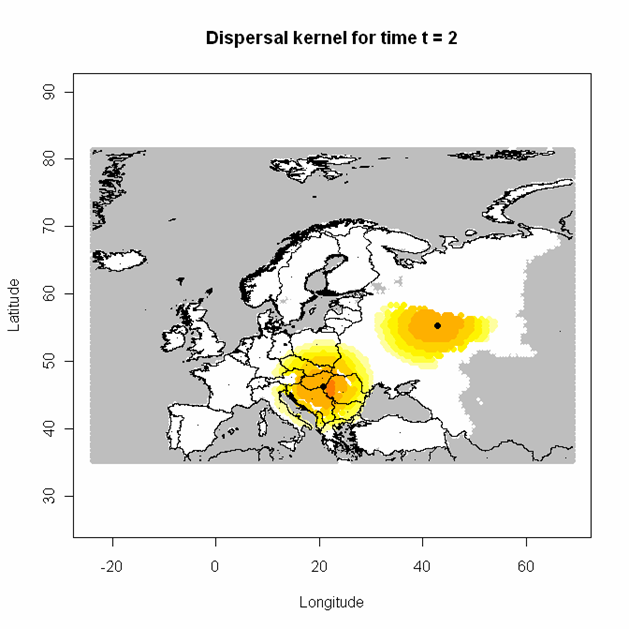


**Figure 3-7:** Output of the dispersal kernel model (*N*_0_=2, *lmax*=7, *t* = 2, *u* = 100, *p* = 50, *nentry*=2) in decimal degrees.

In R, write:

res = dispk()

and press enter to run this model with default values, or if you want to use other parameter values, you can write for instance:

res = dispk(t=10, lmax=40, p=50, u=100, figkm=T,figdd=F)

*N*_0_ and *nentry* are not required if you provide some entry points (if you provide a “presence.txt” file, these values are automatically used whatever values you enter here). If you want to have a figure in decimal degrees, write figdd=T (T means true), and if you want a figure in a metric system, write figkm=T (otherwise write = F, F means false). You will see the result on a figure. The colors are going from white for a population density < 10^(-6) to yellow, orange and red for a population density ≥ 10%. Grey represents missing values (no CLIMEX data). The following commands allow you to have several results:

res$dispk a vector indicating the population density on the grid
res$ntot the total number of cells considered in the model
res$nRA the number of cells within the risk area (EI>0)

res$ninv the number of cells invaded (where the density is above 1)

res$pniche the percentage of cells invaded within the risk area

This model is much more computation intensive than previous models. Therefore, the simulation time is much longer. Note that increasing the year *t* will increase the time for calculation. You will see the year for which the calculations are running on the screen at any time of the calculation (click on the R console window to update the value). The map on the R graphics screen will be refreshed for each time between 1 and *t*.

If you want to define a threshold for the population abundance (%) above which you consider that the area is invaded, and see the resulting map, you should type:

plotkernel(res$dispk,res$presence, figkm=F,figdd=T, legend=F,threshold=0.01)


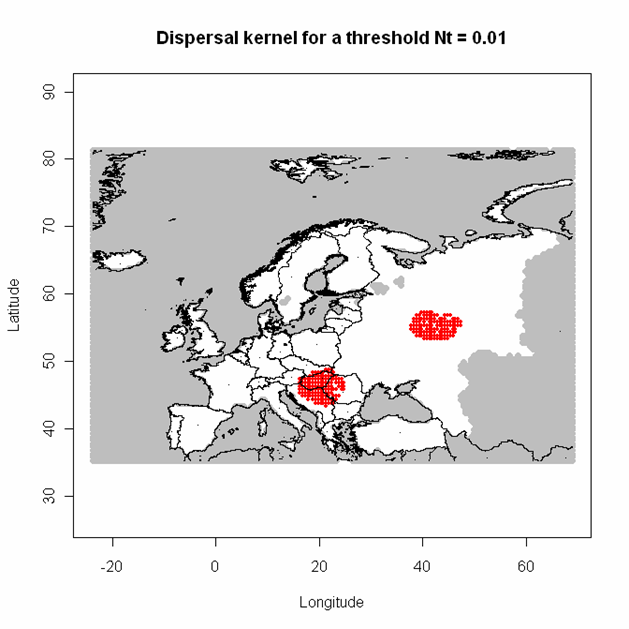


**Figure 3-8:** Figure 3.3-7 with a threshold = 0.01%. The population abundance (%) is above this threshold in red cells.

Note that you can also use this function to plot the map resulting from the dispersal kernel model. In this case, provide no value for threshold:

plotkernel(res$dispk, res$presence, t=10, legend=T, threshold=NULL, figkm=F,figdd=T)

**To export the output**:

Since it takes a long time to obtain the output for this model, the exportation procedure is slightly different from the other models. Here, the model should be called as above, and then, in a second step, the following function should be called:

exportkernel(res$dispk, name=”Kernelresult”)

In this case, the output will be exported in a tif file called Kernelresult.tif in the working directory. The projection of this raster file is Lambert Azimuthal Equal Area (ETRS 1989 LAEA), a European metric projection used in risk mapping (see WP3 and the metric supplement, section 3.4). This file can be combined to other risk maps using MCAS (see WP3). The values given by this file represent the population density expressed as a percentage of the carrying capacity at time *t* defined by the end user.

**Table 5b. Correspondence between the names of the parameters for model D**

| *Name in this tutorial and in the code* | *Name in the article* | *Description* |
| --- | --- | --- |
| ***N_0_*** | ***p_0_*** | Population abundance for all suitable cells at time t = 0 expressed as a percentage of the maximum abundance (carrying capacity) |
| ***K*** | ***P_max_*** | Carrying capacity |
| ***λ*_max_** | ***λ*_max_** | Maximum year to year multiplication factor |
| ***p*** | ***υ*** | Shape parameter |
| ***u*** | ***u*** | Scale parameter |
| ***t*** | ***t*** | The time horizon for the simulations |

### 3.3.3.2 Stochastic model

This model is similar to the previous one, except that we choose at random where the individuals disperse according to the dispersal probabilities provided by the 2Dt kernel. The number of random jumps from one cell is equal to the population density (rounded to the unit). Generally, several hundred replicate simulations should be done to provide a reasonable indication of the invasion probability.

The time needed for the simulations is relatively long. This time closely depends on the number of cells within the area of potential establishment and the number of replicate simulations. For instance, for the Colorado beetle and the corresponding CLIMEX model, the calculations take 2h25min for only 10 replicate simulations and *t* =15 years. Although this version is available, it has not been widely tested and, until now, it is recommended to use the deterministic version as a priority.

**Table 6**: List of parameters for the stochastic version of model 5(DK)

| *Parameter* | *Type or unit* | *Range* | *Default value* | *Type of parameter* | *Description* |
| --- | --- | --- | --- | --- | --- |
| ***N_0_*** | % | 0-100 | Given in the file presence.txt | Biological | Population abundance at time t = 0 expressed as a percentage of the maximum population abundance (carrying capacity K) |
| ***λ*_max_** |  | ≥1 | 7 | Biological | Maximum year to year multiplication factor (“finite growth rate”) that a population could achieve under optimal conditions assuming unlimited space |
| ***P*** |  | 0-1 | 1 | Biological | Proportion of the population engaged in dispersal |
| ***p*** | Degree of freedom | ≥2 | 50 | Biological | Shape parameter of the 2Dt dispersal kernel (number of degrees of freedom) |
| ***u*** | km | > grid resolution | 100 | Biological | Scale parameter of the 2Dt dispersal kernel |
| ***t*** | year | ≥1 | 1 | user choice | The time horizon for the simulations (year) to calculate the spread |
| ***sim*** |  | ≥1 | 200 | user choice | The number of replicate simulations. Since the model is stochastic, it is necessary to make several simulations and calculate the average result. |
| ***presencefile*** | Logical | T or F | T | user choice | If T (true), a presence file should be provided. If F (false), then provide the number of entry points (*nentry*) to be selected at random in the suitable area and the population density *N_0_*. |
| ***nentry*** | number | ≥1 | null | user choice | The number of entry points to select at random within the risk area (EI>0) if no presence file is provided |
| ***figkm*** | Logical | T or F | T | Graphic | If T (true), then *figdd* should be F, and the figure will be in a metric system |
| ***figdd*** | Logical | T or F | F | Graphic | If T (true), then *figkm* should be F, and the figure will be in a decimal degrees |


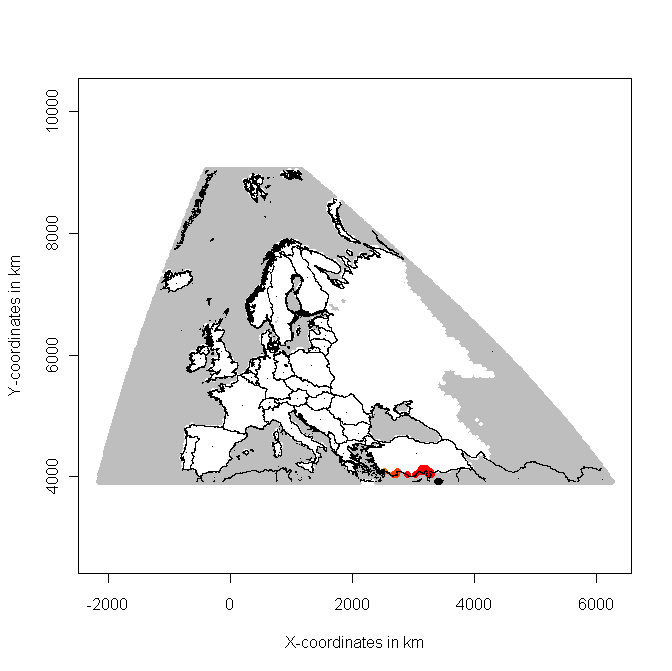

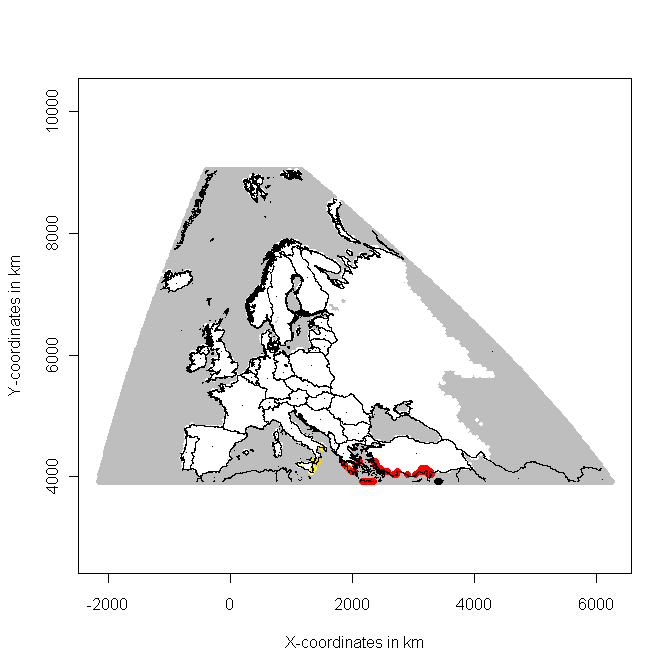


**Figure 3-9**: Output of the stochastic version (left) and deterministic version (right) for t = 3, u = 100, p =50, sim=200, and the CLIMEX model of the Queensland fruit fly.

In R, write:

res = dispksto()

and press enter to run this model with default values, or if you want to use other parameter values, you can write for instance:

res = dispksto(N0=1, t=1, p=50, u=100, nentry=1, figkm=T,figdd=F,sim=10)

*N*_0_ and *nentry* are not required if you provide some entry points (if you provide a “presence.txt” file, these values are automatically derived whatever values you enter here). If you want to have a figure in decimal degrees, write figdd=T (T means true), and if you want a figure in a metric system, write figkm=T (otherwise write = F, F means false). You will see the result on a figure. The colors are going from white for a population density < 10^(-6) to yellow, orange and red for a population density ≥ 10%. Grey represents missing values (no CLIMEX data). The following commands allow you to have several results:

res$dispksto a vector indicating the population density relative to the grid
res$ntot the total number of cells considered in the model
res$nRA the number of cells within the risk area (EI>0)

res$ninv the number of cells invaded (where the density is above 1)

res$pniche the percentage of assets invaded within the risk area

Note also that increasing the number of simulations will increase the time for calculation. You will see the number of simulations done on the screen at any time of the calculation (click on the R console window to update the value).

# **4 Supplement for the metric version**

## This tutorial is associated with the R SpreadModule code version 8 (May 30^th^, 2011) for the metric version.

The spread module was initially developed on a regular grid in latitude and longitude (0.5° x 0.5°), directly derived from the CLIMEX dataset. Since the generic integrated model developed by the PRATIQUE project aims to combine various types of GIS datasets (notably including host distribution), it was decided to choose a single coordinate system: the metric projection, Lambert Azimuthal Equal Area (ETRS 1989 LAEA) and a single spatial resolution, 10 km x 10 km, for all the maps.

Therefore, this section devoted to the metric version aims to guide the users using this version of the spread module and to describe the changes. The main advantage for the end-user is the possibility to work in the same projection as the projection used in risk maps (see Baker et al. 2012). As well as the DD version, it is possible to export the outputs in a tif file that can be used in MCAS and integrated in Decision Support Scheme for Mapping Endangered Areas (Baker et al. 2012).

Since the estimation of some parameters depends on the spatial resolution, it is necessary to carefully check the calculations and update the estimations when switching from the DD version to the metric version, and vice versa.

**The CLIMEX file:**

Similarly to the non metric version, you can also use the output of CLIMEX directly (csv file; climexcsv = T) or a modified file providing only latitude, longitude, EI and GI (in this order) (txt file; climexcsv = F).

**Be careful** to use the meteorological dataset: CRU_WRLD_V2_1.mm

- click on MetManager icon on the locations line, add/edit/remove sequence

- click on “new”

- select CRU_WRLD_V2_1.mm

- find Europe CRU HD V2_1, then click on OK

- click on OK

The spatial resolution of the meteorological dataset should be absolutely 0.5° everywhere (even around the longitude 0°). By using the meteorological dataset CRU_WRLD_V2_1.mm some problems can be avoided.

**Description of the metric version**

library(sp)

library(raster)

library(rgdal)

climexcsv = T

elevmax = F

habitatfile = T

habitatformat = ".tif "

*# load the R code*

plotRA()


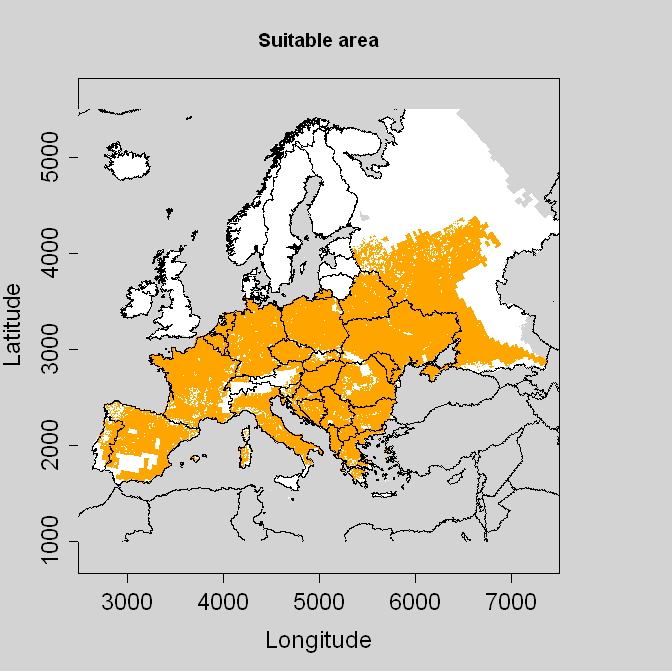


plotGI()


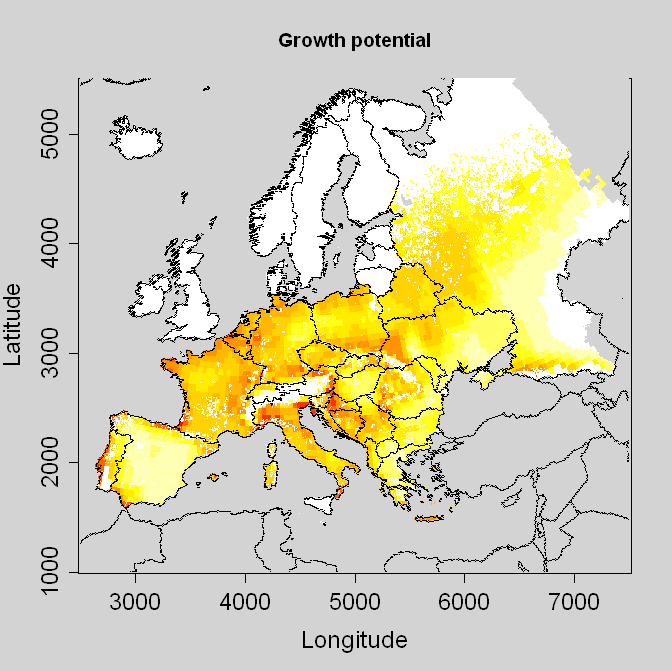


plothabitat()


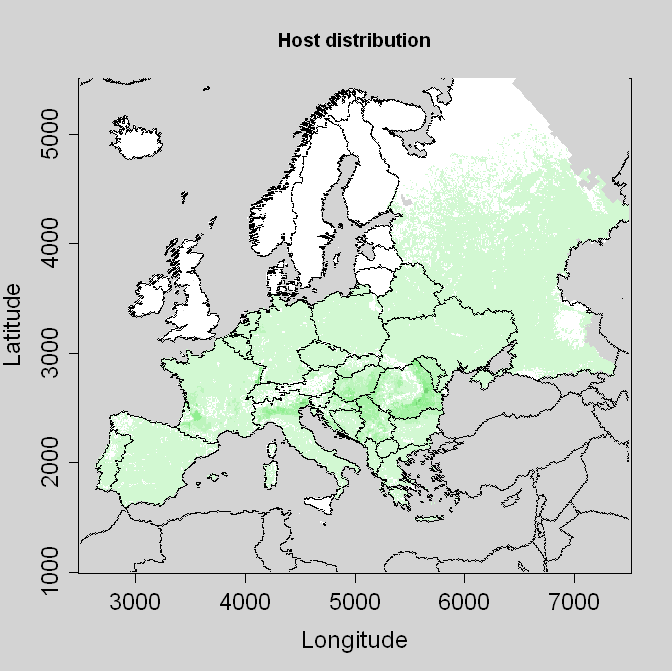


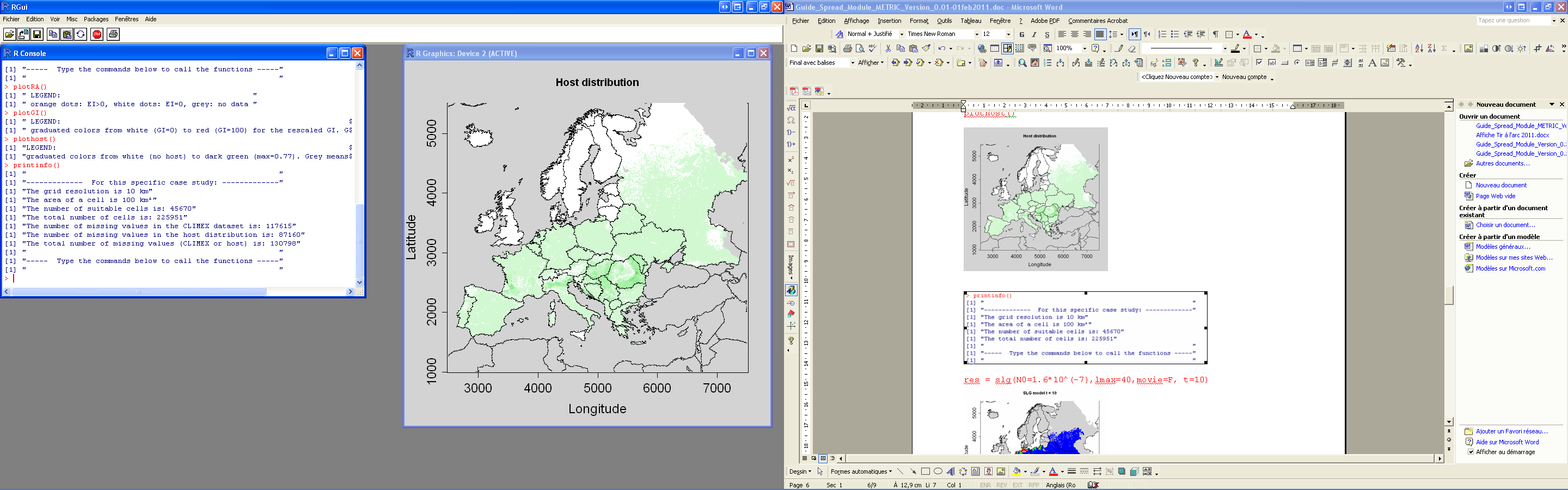


Note that in this version, you do not have the choice of viewing the map in decimal degrees or in a metric system (the variables figdd and figkm do not exist anymore in this version) because the map is always projected in the ETRS 1989 LAEA metric system.

## *4.1* Temporal process models

4.1.1 Model C: Population dynamics model *“Simple Logistic Growth model”*

In this metric version, nothing has changed to call the function:

res = slg(N0=1.6*10^(-7),lmax=40,movie=F, t=10)


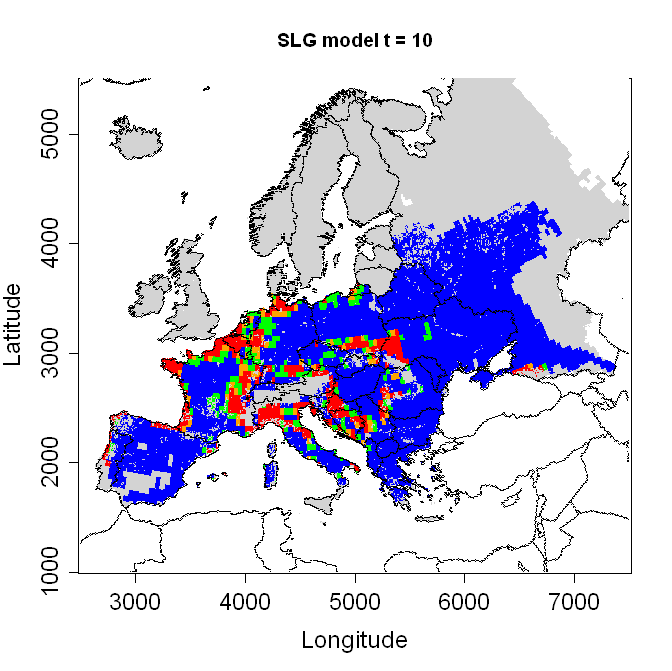


***Exporting the output***

res = slg(N0=1.6*10^(-7),lmax=40,movie=F, t=10, export=T, name= “SLGoutput”)

We do not recommend exporting the file at first because it takes a long time to create the raster file and export it, and problems arise if you export the file several times with the same name. The best approach is to play with the model first and then, when the results are satisfactory and you want to work on this output, you can export it (as mentioned above). Missing values (NA in the exported file) coming from both the CLIMEX dataset and the habitat distribution (if used). The data represent the percentage of the carrying capacity of the species (0-100).

***Warning: the parameter estimates should be updated***

$N_{0}=100*\frac{n_{0}}{K}$ with

K = area_cell (km²) * proportion_covered_by_host * max_population_density (/km²)

Therefore, we have:

K = (10*10) (cell area in km²) * 0.20 (host proportion)*200e6

n0 = 100

=> **N0 = 5e-8**

4.1.2 Model A: Temporal spread over cells integrated with impact  ***“****Logistic Growth model based on Economic values”*

In this metric version, there are also three cases for the economic data file. We can use:

1. a raster of economic values of the habitat in LAEA projection at the 10 km resolution. In this case, the value should be the value in euros of the crop for each cell. If this value is in another unit, you should use the multiplicative factor *mult* in the function to make conversion. For instance, if the value is euros per km², then you should simply put *mult* = 100 (to have per cell of 10 km * 10 km).
2. a raster of habitat density. In this case, the value, *valperhost,* should be given per km² assuming that habitat is everywhere.
3. random values

res = lgecon(N0=0.0388,r=0.45,econraster=T,econformat= ".tif ",t=16,mult=1, sim=1)

***Warning: the parameter estimates should be updated***

*N_0_* = 100 * *n*_0_ / *K*

with *n*_0_, the number of invaded cells at *t* = 0, and *K* the number of suitable cells.

We assume that only one cell is invaded at the beginning (only one infested location).

The number of suitable cells is found when calling the module’s information: printinfo(). We have 45670 suitable cells.

Thus, the initial density for the logistic function is: $N_{0}=100*\frac{1}{45670}$= 0.0022, i.e. one cell colonized out of the 45670 cells with EI>0.

=> **N0 = 0.0022**

We roughly estimated that the colonized area in 2008 covered approximately 1/3^rd^ of the risk area (=> 45670 / 3 = 15223 infested cells in 2008 over the 45670 grid cells (K)). $\begin{matrix} \ln\left(\frac{15223}{45670-15223}\right)=\ln\left(\frac{1}{45670-1}\right)+16r \\ r=\frac{1}{16}\left(\ln\left(\frac{15223}{30447}\right)-\ln\left(\frac{1}{45669}\right)\right)=0.62\text{ yr}^{\text{-1}} \end{matrix}$

=> **r = 0.62**


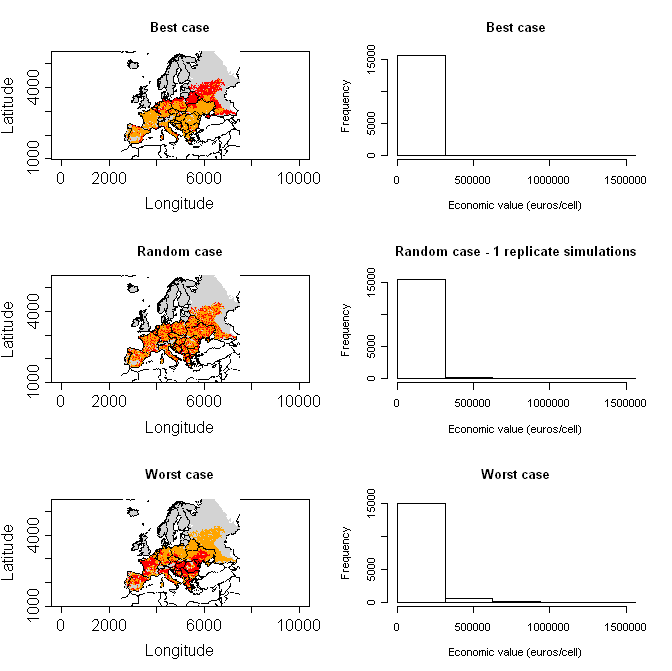


***Exporting the output***

res = lgecon(N0=0.0388,r=0.45,econraster = T, t=16, mult=1, export=T, name=“EconOutput”)

In this case, three files are exported:

- name_best: file for the best case scenario
- name_worst: file for the worst case scenario
- name_rand: file for the random case scenario (cumulating the number *sim* of replicate simulations)

In these files, the value 1 means that the cell is invaded, the value 0 means that the cell is within the area of potential establishment but not invaded, the value -1 means that the cell is not in the area of potential establishment and the value -9999 means that data is missing.

To know how many cells are covered by some points of pest presence, you can use the following function:

pointtocell(“name_of_your_file.txt”)

You should enter the name of the file containing the longitude (column 1) and latitude (column 2) in km (ETRS 1989 LAEA projection). This file should be located in the working directory. For each point, the function determines the cell where it is located within the area of potential establishment. If the point is located outside the area of potential establishment, then the corresponding cell is the closest one within this area.

## 4.2 Spatial process models

4.2.1 Model B: Radial range expansion model

***A function to transform the decimal degrees in LAEA projection system, in km***

The following function has been added to find the coordinates of a point in LAEA (km):

proj(20.3,44.82)

The first value is the longitude and the second one is the latitude in decimal degrees. This function converts the coordinates of the introduction point to the required projection coordinates.

If you need the inverse function, use:

projinv(5020,2090)

the first value should be the longitude in km and the second value the latitude in km (LAEA projection). The function returns the coordinates of this point in decimal degrees.

res = radial(RR=60,t=16,coord=c(5134.207, 2468.253))


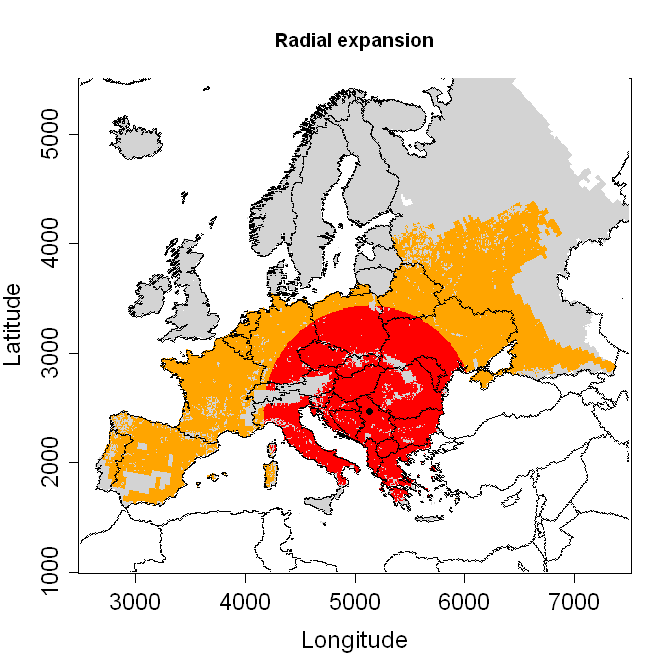


***Exporting the output***

res = radial(RR=60,t=16,coord=c(5134.207, 2468.253), export=T, name=“RadialOutput”)

In this case, the value 1 means that the cell is invaded, the value 0 means that the cell is within the area of potential establishment but not invaded, the value -1 means that the cell is not in the area of potential establishment and the value -9999 means that data is missing.

4.2.2 Hybrid model

***Exporting the output***

res=radialrand(N0=0.0388,r=0.45,t=16,RR=60,coord= c(5134.207, 2468.253),export=T,name=“HybridOutput”)


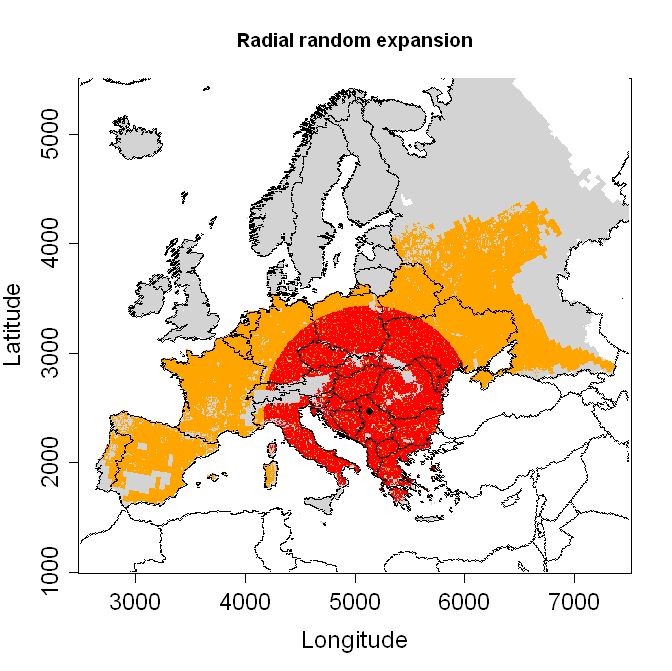


In these files, the value 1 means that the cell is invaded, the value 0 means that the cell is within the area of potential establishment but not invaded, the value -1 means that the cell is not in the area of potential establishment and the value -9999 means that data is missing.

***Warning: the parameter estimates should be updated***

=> **N0 = 0.0022**

=> **r = 0.62**

4.2.3 Model D: Dispersal kernel model

Note that the dispersal kernel can theoretically be used in the metric version of the spread module, but there is a technical problem that is impossible to solve until now: the calculation time. The number of cells is 13 times higher in the metric version than in the DD version (with a 0.5° resolution) for the dispersal kernel (17,298 in the DD version vs 225,951 cells in the metric version). For *Diabrotica virgifera virgifera*, for instance, the number of cells within the area of potential establishment is 16 times higher in the metric version than in the DD version (with a 0.5° resolution) for the dispersal kernel (3,326 in the DD version vs 54,792 cells in the metric version). Since the time increases exponentially with the number of cells within the area of potential establishment (potential source points), it is almost impossible to use this model in practice. Therefore, this model is described hereafter but it is recommended **not to apply the dispersal kernel model in the metric version**.

***Exporting the output***

Since this last model takes a long time for the simulations, a separate function has been coded to export the outputs.

You should first create the presence file (if you need) and call the function:


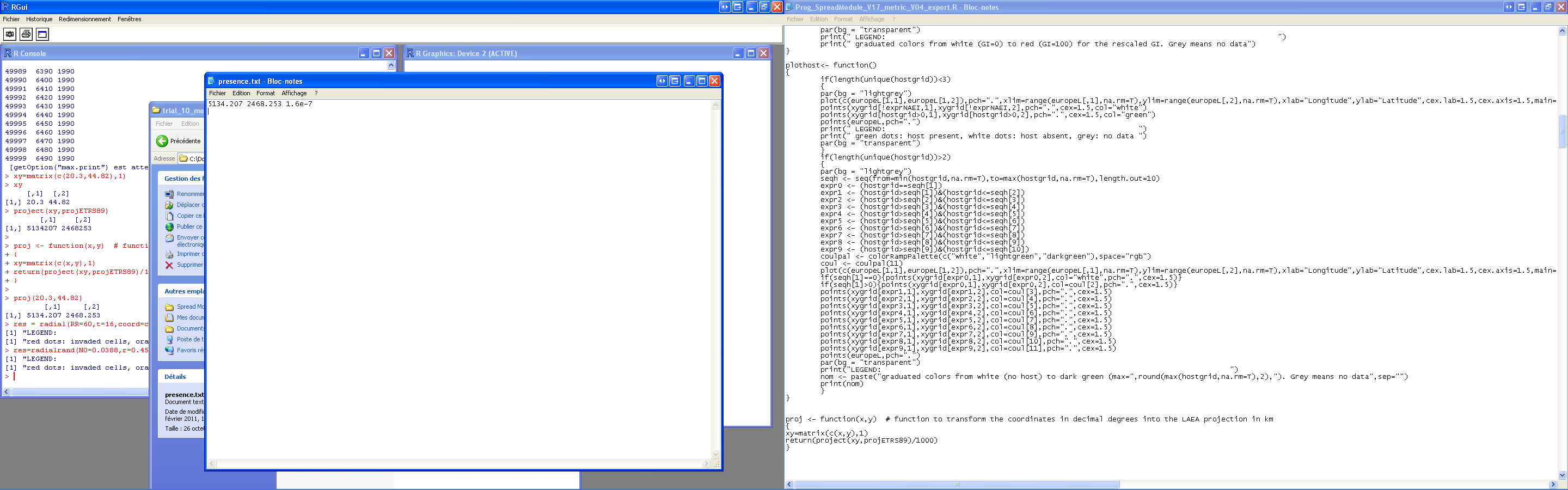


res = dispk(N0=NULL, t=1,lmax=40, p=5,u=60, presencefile=T, nentry=NULL)

Then call another one to export:
exportkernel(res$dispk,name=”KernelOutput”)

***Warning: the parameter estimates should be updated***

=> **N0 = 5e-8**

Note that the stochastic version is not available in the metric version because of the very long time required for the simulations.

# **5 Supplement for the world version**

## This tutorial is associated with the R SpreadModule code version 21 world. The objective is to describe the changes done to allow the user to apply the spread module anywhere in the world.

## In the previous spread module package, there were two versions: the decimal degree version (“DD version”) and a version in the European metric projection LAEA (“metric version”). To be applicable to any part of the world, the DD version has been adjusted and called “DD version world”. The maps of the study countries will be automatically displayed, following the spatial extent given by the CLIMEX model.

## Compared to the previous DD version, here are the following changes:

## (1) There is still the possibility to export the results of each spread model, but in this new version, it will be exported in decimal degree and not in a metric projection. Of course, you can then work on this output file using a GIS software such as ArcGIS and convert it in the projection you wish.

## (2) There is not the possibility to view directly the result of the spread models in a metric projection. The results will be displayed only in decimal degrees (the parameters figdd and figkm do not exist any more).

## Remember that before calling the code and the models, you should first write a few lines of code (see the “Quick launch” section).

# **6 REFERENCES**

Baker RHA, Battisti A, Bremmer J, Kenis M, Mumford J, Petter F, Schrader G, Bacher S, De Barro P, Hulme PE, Karadjova O, Lansink AO, Pruvost O, Pysek P, Roques A, Baranchikov Y & Sun JH (2009) PRATIQUE: a research project to enhance pest risk analysis techniques in the European Union. Bull. OEPP⁄EPPO Bulletin 39, 87-93.

Baker R, Benninga J, Bremmer J, Brunel S, Dupin M, Eyre D, Ilieva Z, Jarosik V, Kehlenbeck H, Kriticos D, Makowski D, Pergl J, Reynaud P, Robinet C, Soliman T, Van der Werf W, Worner S. (2012) A decision support scheme for mapping endangered areas in pest risk analysis. Bull. OEPP/EPPO Bull. (in press)

Clark JS, Silman M, Kern R, Macklin E & HilleRisLambers J (1999) Seed dispersal near and far: patterns across temperate and tropical forests. Ecology, 80, 1475-1494.

R Development Core Team (2009). R: A language and environment for statistical computing. R Foundation for Statistical Computing, Vienna, Austria. ISBN 3-900051-07-0, URL http://www.R-project.org.

1. These instructions are valid for CLIMEX version 3 [↑](#footnote-ref-2)
