# Supplementary material for: A Suite of Models to Support the Quantitative Assessment of Spread in Pest Risk Analysis
Source: PLoS One. 2012 Oct 9;7(10):e43366. doi: 10.1371/journal.pone.0043366 (PMC3467266; doi:10.1371/journal.pone.0043366)
Supplement: Materials S2 — Case studies. (DOC) [file pone.0043366.s002.doc]

**S2 Case studies**

This document contains supplementary material to the paper "A suite of models to support the quantitative assessment of spread in pest risk analysis" by Robinet C, Kehlenbeck H, Kriticos DJ, Baker RHA, Battisti A, Brunel S, Dupin M, Eyre D, Faccoli M, Ilieva Z, Kenis M, Knight J, Reynaud P, Yart A & van der Werf W.

Note on font color: The main text is in black font. Red font color indicates R code.

**Table of contents**

1 Introduction Page 2

2 Citrus Longhorn Beetle, *Anoplophora chinensis* Page 2

3 Asian Longhorn Beetle, *Anoplophora glabripennis* Page 16

4 Root-knot nematode, *Meloidogyne enterolobii* Page 32

5 Pine wood nematode, *Bursaphelenchus xylophilus,* and resulting
 wilt disease Page 48

6 Water hyacinth, *Eichhornia crassipes* Page 62

7 Pitch canker, *Gibberella circinata* Page 71

**1. Introduction**

The spread module has been tested on six further case study pests: the Citrus longhorn beetle (*Anoplophora chinensis*), the Asian longhorned beetle (*Anoplophora glabripennis*), the Root-knot nematode (*Meloidogyne enterolobii)*  the Pine wood nematode (*Bursaphelenchus xylophilus)* and resulting wilt disease, the Water hyacinth (*Eichhornia crassipes)* and pitch canker (*Gibberella circinata).* Modelling has been performed with the R-code Version V17. Each of the case study chapters first summarizes necessary information and data on the pest species and then explains how parameters were derived and shows the output of the models that were applied to the pest. Parameterisation was done based on our knowledge up until July 2011 and improvement is possible in the future if new data or information becomes available. The main purpose of this document is to show how the values of the parameters can be found and how the models can be applied.

**2. *Anoplophora* *chinensis* (Citrus longhorn beetle)**

Authors: Hella Kehlenbeck (JKI, Germany), Dominic Eyre (Fera, UK) and Marc Kenis (CABI, Switzerland)

**Information on *A. chinensis***

*A. chinensis* is a longhorn beetle whose larvae bore inside living hosts. It is a major wood boring pest of fruit trees, including *Citrus*, in China, Japan and Korea.

Information and data on *A. chinensis* are mainly based on a Pest Risk Assessment conducted in 2008 (Van der Gaag et al, 2008), on information provided on the EPPO website on *A. chinensis* (see references) and Van der Gaag et al. (2010).

Host range and host distribution

*A. chinensis* is polyphagous on many deciduous trees: amenity trees and many natural forests in northern and southern Member States are at risk. *A. chinensis* can attack plant species of more than 20 plant families. In Italy, plant species belonging to 22 genera are attacked. Host plants include *Acer*, *Betula Corylus, Fagus, Prunus, Citrus*, *Malus*, *Platanus, Populus, Pyrus, Rosa,* *Ulmus* and *Salix* and suitable habitats are widespread in the EU. For this case study, therefore, we used the percentage of land covered by broadleaf forest (from Päivinen et al., 2001) see figure 2-1).

**
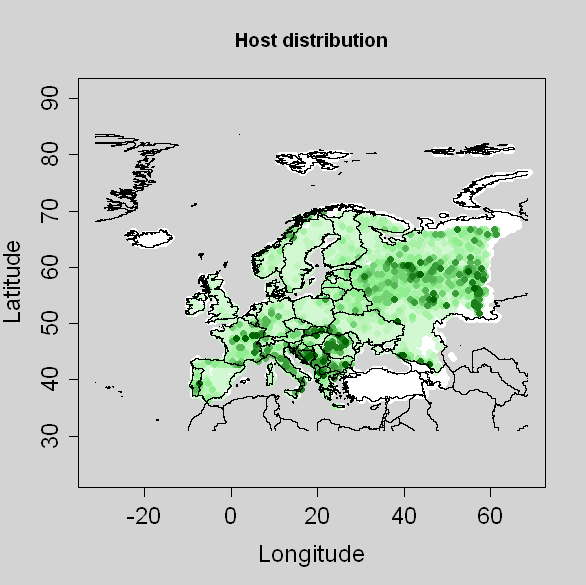
**

**Figure 2-1:** Host plant distribution for *Anoplophora chinensis* in Europe based on the percentage of area covered with broadleaf forest distribution according to Päivinen et al., 2001 (graduated colors from white (no host) to dark green (max=91%). Grey means no data).

Findings in Europe

A population of *A. chinensis,* first detected in 2000*,* is present in several areas of Lombardy in northern Italy (Provinces of Milano, Varese and Brescia). Although beetles have been found in thousands of trees and shrubs and the outbreak area now covers over 100 km², it is under official control (OEPP, 2011). Van der Gaag et. al (2010) summarized that outbreaks of the pest have been discovered in Soyons, France (2003), Westland, the Netherlands (2007), Croatia (2007), Rome, Italy (2008) and Boskoop, the Netherlands (2009). *A. chinensis* has also been intercepted in several European countries (UK, Netherlands, Germany, France, Switzerland).

Pathways

There is a history of *A. chinensis* being transported from Asia into the EU via plants for planting, especially with bonsai and young maple trees (*Acer* spp.). In the Netherlands, 1.6 to 2 million *Acer* spp. were imported per year during the period 2005 – 2007. Dutch importers and growers estimate the total value of these plants (wholesale price) at about € 3 – 6 million. They also estimate that 30 – 70 % of all *Acer* spp. imported into the EU from China, Japan and Korea are imported via the Netherlands. The total import of *Acer* spp. from these countries into the EU was assessed to be about 4 million plants with a total value of about € 6 –12 million. On one occasion, *A. chinensis* has also been intercepted on wood packing material although this pathway is considered to be insignificant compared to live plants (Haack et al. 2010).

Climatic suitability in Europe

A CLIMEX study conducted by the Netherlands in 2004 (De Boer, 2004) indicated that it was unlikely that *A. chinensis* could establish in North Western European countries. A UK PRA had concluded that it was unlikely that *A. chinensis* could establish in the UK based on a comparison of climate data from one of the warmest parts of the UK and areas where *A. chinensis* is known to be present and the presumption that a maximum of two years was needed to complete its life cycle (Baker & Eyre, 2006). However, the finding in the Netherlands with summer temperatures comparable to those in the warmest parts of the UK and a life cycle of three years shows that *A. chinensis* can establish in the Netherlands and probably also in the UK. Additionally, findings of breeding populations in Italy, France and the Netherlands, have shown that *A. chinensis* is able to establish in a range ofclimatic regions of the EU. Adults are not active at temperatures below about 10°C.

CLIMEX file

The output file of a new CLIMEX model for *A. chinensis* was provided by Dominic Eyre. This new model takes account of the findings of the beetle in the more Northern parts of Europe in the Netherlands, France and the UK. Figure 2-2 shows the environmental index and figure 2-3 the risk area and the growth potential for *A. chinensis* according to this new CLIMEX model.

**
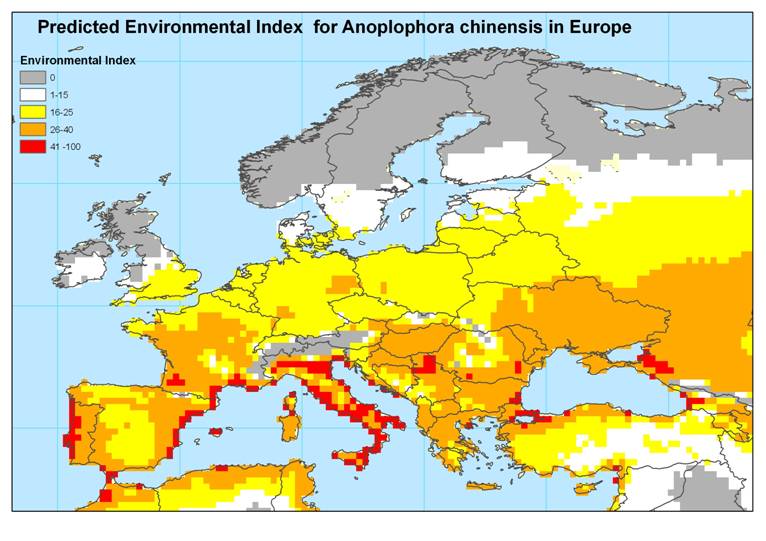
**

**Figure 2-2:** Environmental Index for *Anoplophora chinensis*in Europe based on a CLIMEX model by Dominic Eyre (October 2010).

The parameters of the Climex model are as follows:

| **Index** | **Parameter** | **Values** |
| --- | --- | --- |
| Tempertature | DV0= lower threshold | 7 |
|  | DV1 = lower optimum temperature | 15 |
|  | DV2 = upper optimum temperature | 28 |
|  | DV3= upper threshold | 29 |
| Moisture | SM0 = lower soil moisture | 0.05 |
|  | SM1= lower optimum soil moisture | 0.08 |
|  | SM2 = upper optimum soil moisture | 1.5 |
|  | SM3 = upper soil moisture threshold | 1.8 |
| Cold Stress | TTCS = temperature threshold | -8 |
|  | THCS = stress accumulation rate | -0.002 |
| Heat stress | TTHS = temperature threshold | 29 |
|  | THHS = stress accumulation rate | 0.00075 |
|  | DTHS = degree-day threshold | 150 |
|  | DTHS = degree-day stress | 1 |
| Dry stress | SMDS = soil moisture dry stress threshold | 0.01 |
|  | HDS = stress accumulation rate | -0.0025 |
| Wet stress | SMWS = soil moisture wet stress threshold | 1.8 |
|  | HWS = stress accumulation rate | 0.0015 |
| Annual heat sum | PPP= degree day threshold | 1550 |

Reproduction strategy

In Southern Europe, *A. chinensis* has a life cycle of 1-2 years and populations will build up much faster than in Northern European countries.

No information is available about the number of female and male beetles that is needed to start a new population. The presence of only one male and one female beetle at the same location and at the same time may be sufficient to start a new population. Findings of trees in France and the Netherlands with exit holes close to a nursery with trees from Eastern Asia suggest that only a few beetles are needed to infest new areas. However, this is very uncertain as information is lacking about the number of beetles that were actually present when eggs were deposited on these trees.


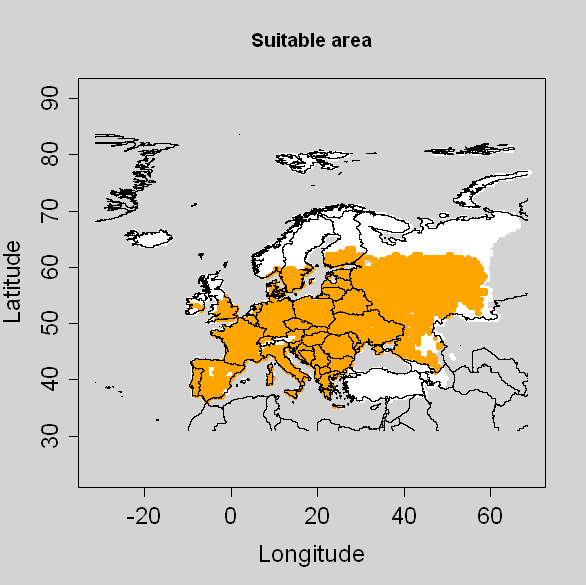

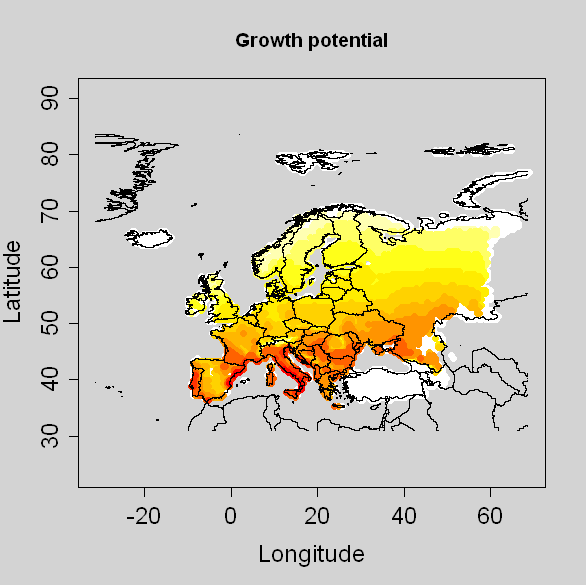

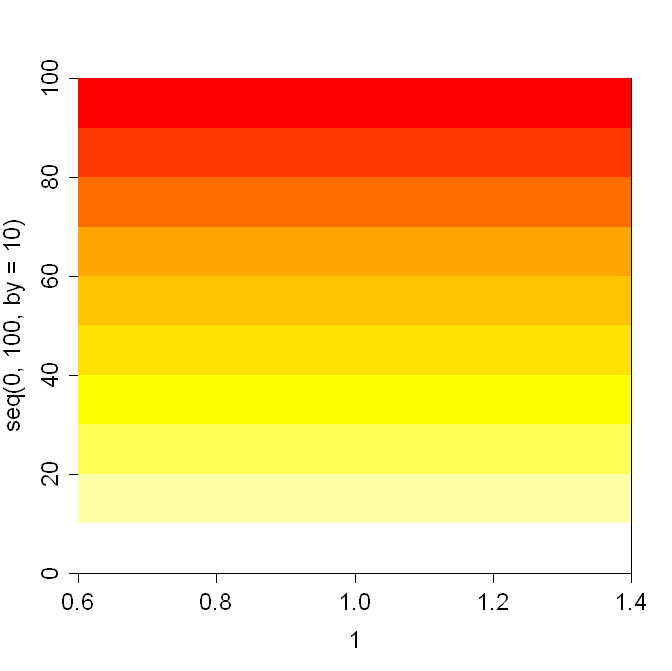


**Figure 2-3:** Suitable area (on the left; orange dots: EI>0 and host present, white dots: EI=0 or host absent, grey: no data) and growth potential (adjusted GI; on the right) for *A. chinensis* in Europe based on a CLIMEX model by D. Eyre (October 2010; EI = Ecoclimatic Index, indicates, how favourable the climate is for the long term survival of the species; GI=Growth Index indicates the overall potential for the population growth).

Natural spread:

Adult *A. chinensis* are considered to behave like the related species, *A. glabripennis,* in that they usually do not fly further than 400 m. Natural spread from the infested area in Italy (Lombardy) is therefore likely to proceed slowly. It is thought that *A. chinensis,* like *A. glabripennis,* will usually stay near the tree from which it emerged:

In France, two *Acer* trees were infested next to the nursery that had imported infested plants from eastern Asia.

In the Netherlands, *Acer* trees were infested within 30 m of the nursery that had imported infested plants. The infested trees were found during the winter of 2007/2008 while the pest had probably already been introduced in 2002.

Thus, *A. chinensis* will probably spread slowly by natural means. At high population densities *A. chinensis* may fly more than 2 km and may spread more rapidly. However, it is likely to take several years for populations to build up to high densities at new outbreak sites in the EU.

Human assisted spread:

*A. chinensis* could spread by human assistance in several ways

a) By trade in infested trees - *A. chinensis* has been introduced into new areas by movement of infested plants over large distances (from Eastern Asia to Europe and the USA). If areas become infested in which plants are grown for trade, *A. chinensis* can be moved over large distances within the PRA area.

b) As a contaminant on transport vehicles

c) By movement of infested wood – this is considered relatively low risk, because *A. chinensis* generally infests the lower part of trunks which are less likely to be used as firewood.

Topography or elevation limits

No information is available on the elevation limits for *A. chinensis*, therefore they were not considered.

Assumptions: for the purposes of Models B and D, it has been assumed that *A. chinensis* continues to spread at the rate it had apparently spread between the estimated time of introduction (1981-1991) until 2007. It should be considered that the outbreak is under eradication. The measures taken to eradicate and contain the pest have been substantially increased since 2008 which has significantly reduced the potential for expansion.

**Model C:** Population dynamics model

*For this model we assume that an initial population abundance p_0_ (%) is introduced in each suitable cell (cells where EI > 0) and then we simulate the “spread” or more precisely the growth within each cell according to a logistic function. The output map shows the areas which are the most suitable for population growth assuming introduction.*

- **Starting population *p_0_***

*p_0_  is the population abundance for all suitable cells at time t = 0 expressed as a percentage of the maximum abundance (carrying capacity P_max_).*

*Calculation:*

$p_{0}=100*\frac{P_{0}}{P_{\max}}$ *with P_0_ the number of introduced individuals, in each suitable cell, and P_max_ the carrying capacity (the maximum number of individuals in a cell).*

*P_max_ = area_cell (km²) * proportion_covered_by_host * max_population_density (/km²), or
P_max_ = area_cell (km²) * host_plant_density (/km²) * max_population_density (/host plant)*

Relevant information and data

Details of an outbreak in the Netherlands (from the PRA, 2008): The Netherlands observed 1 – 7 exit holes on infested Acer shrubs and trees (see table in the PRA). The Acer tree had 7 exit holes and 18 larvae of *A. chinensis* were found inside the trunk. If all these 18 larvae will complete their development, the number of beetles per tree would be 25. Therefore an estimated carrying capacity of 25 beetles per tree (and perhaps a lower number for shrubs) seems plausible. The number of exit holes was obtained from living trees, meaning that the beetles did not kill the trees or shrubs.

The CABI Crop Protection Compendium states that: ‘Across all regions, there was a mean of 3.8 holes per [citrus] tree although means between regions varied from 2.2 to 5.9 holes per tree’. These numbers are from orchards where some form of control may be applied.

We assume the maximum population density (max_population_density) to be 5- 25 beetles per host plant

Host trees and shrubs per ha:

50 - 100 trees or shrubs / ha, i.e about 50% hosts of *A. chinensis* = 25 – 50 host plants/ha

Calculation of the capacity P_max_:

P_max1_ = 5 beetles/shrub x 25 hosts/ha x 100 ha (transfer ha to km²) x 1579 km² (grid cell)

P_max1_ = 19.737.500 = 1.97x10^7^ beetles/grid cell

P_max2_ = 25 beetles/shrub x 50 hosts/ha x 100 ha (transfer ha to km²) x 1579 km² (grid cell)

P_max2_ = 197.375.000 = 1.9 x 10^8^ beetles/grid cell

Calculation of p_0_

Starting number of beetles (= P_0_) : can be very low, just 2 beetles (one female and one male) per grid cell could be sufficient

p_0_ = 100 *x starting number P_0_/P_max_ = %

p_0_ = 100 x (2 beetles per grid cell / 1,97x10^7^ beetles/grid cell) -
 100 x (2 beetles per grid cell /1.9 x10^8^  beetles per grid cell

p_0_ = 0,00001% (=1.0 x10^-5^) to 1.0 x 10^-6^

p_0_ = 1.0 x 10^-6^ % - 1.0 x 10^-5^ %

- **The multiplication factor *λ_max_***

*λ_max_ is the maximum year to year multiplication factor (“finite growth rate”) that a population could achieve under optimal conditions assuming unlimited space.*

Relevant information and data

In Europe A. chinensis finds very favorable conditions especially in Italy (besides some small parts of France, Spain and Portugal, see predicted growth index in Fig. 2-3), where a population has established in Lombardy. No data on the population size based on beetle abundances for different years were available from literature or expert knowledge. Therefore published data on the eradication of infested trees in the Lombardian area was used to calculate the population size from the number of infested trees and the number of beetles per infested tree. Over a period of 5 years (between 2002 and 2006) the number of infested and destroyed trees summed up to 2673 (http://www.eppo.org/QUARANTINE/anoplophora_chinensis/chinensis_IT_2007.htm). Given that two beetles (one male and one female) were the initial population, and assuming that there were 5 – 10 beetles/plant, the multiplication factor over these 5 years would have been 6683 – 13365. The multiplication factor for one year (*λ_max_)* can be calculated in the following way:

*λ_max_* = 6683^1/5^ – 13365^1/5^

*λ_max_* = 5.8 – 6.7

*λ_max_* = 6

This value reflects the conditions for population development in Italy. For the Netherlands e.g. the mulitplication factor may be lower. Van der Gaag et al. (2010) expect a low reproduction rate under Dutch conditions. For simulations which should more reflect these conditions a lower value for *λ_max_* may be recommended.

- **Time frame t**

t = 10 - 30 years

The results of the simulation with Model C in figure 2-4 show the area where the species can grow rapidly and therefore become a more important problem compared to other places.

**Results**:


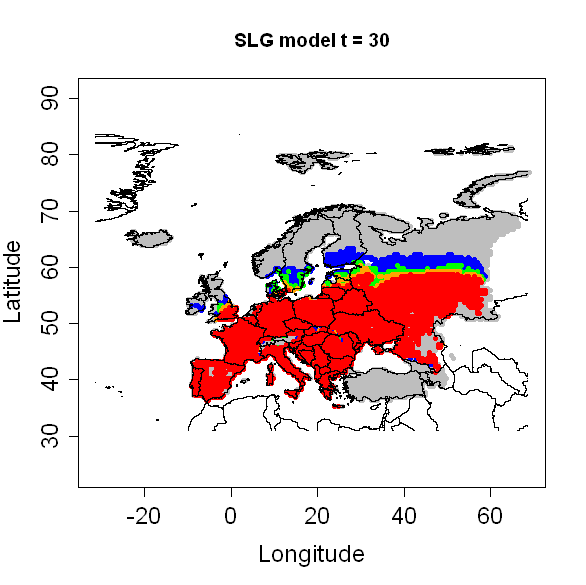

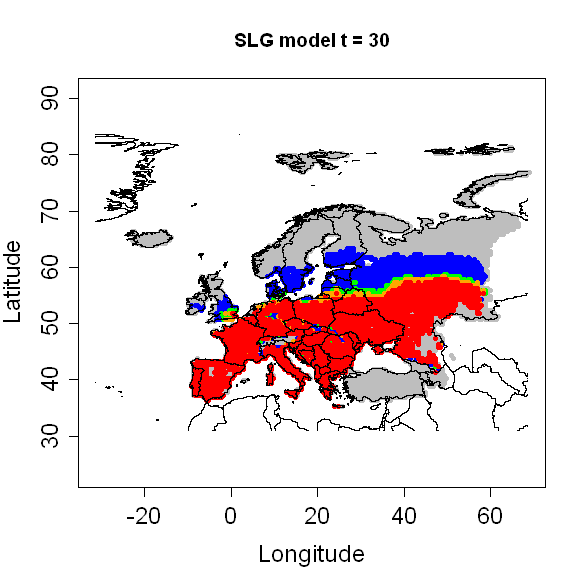


**N_0_=1.0 x10^-6^, *λ_max_*= 6**

**N_0_=1.0 x10^-5^, *λ_max_*= 6**


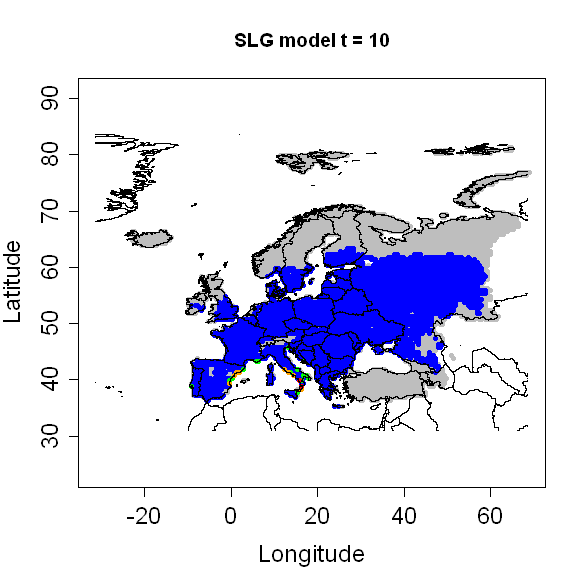

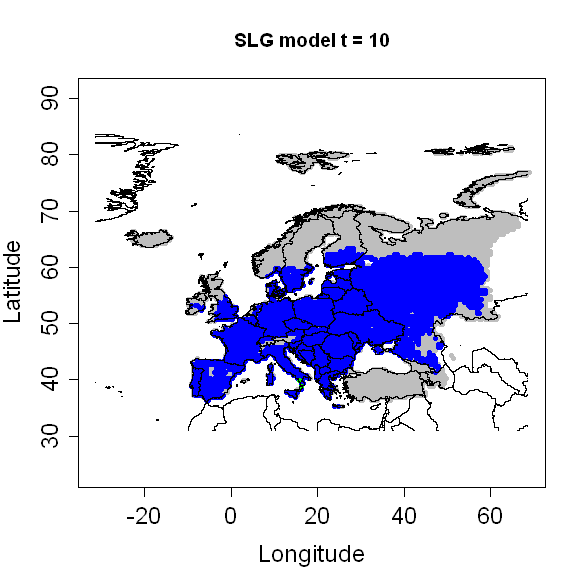


**p_0_=1.0 x10^-6^, *λ_max_*= 6**

**p_0_=1.0 x10^-5^, *λ_max_*= 6**


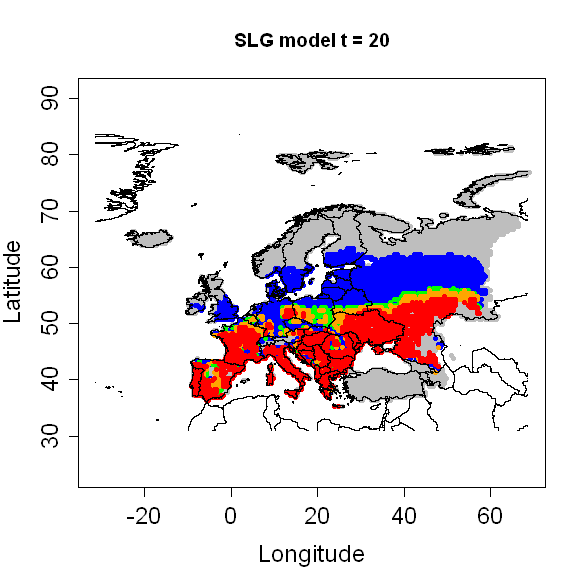

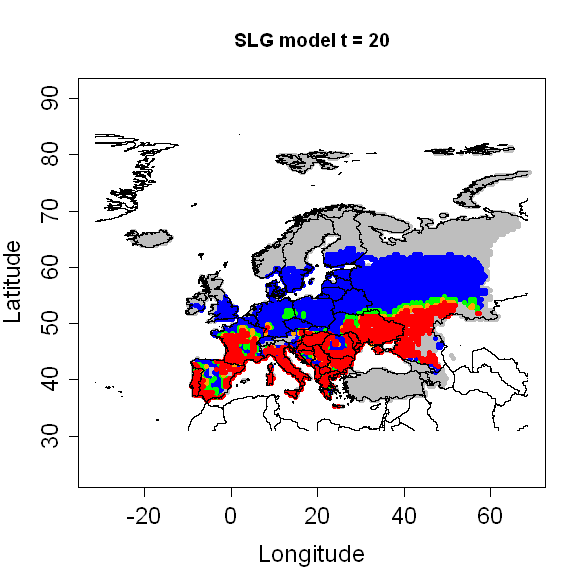


**p_0_=1.0 x10^-6^, *λ_max_*= 6**

**p_0_=1.0 x10^-5^, *λ_max_*= 6**

**Figure 2-4:** Output of Model C for *A. chinensis* for 10, 20 and 30 years, for two different estimates of p_0_, ( blue dots: 0 < p_t_ < = 25, green dots: 25 < p_t_ < = 50, orange dots: 50 < p_t_ < = 75, red dots: 75 < p_t_ < = 100, grey dots: p_t_=0, white: no data). Left pictures: p_0_=1.0x10^-5^; right pictures: p_0_=1.0 x10^-6^ .

**Model B: Radial range expansion model**

*This model aims to determine the potential spread of a species introduced in the PRA area based on the radial rate expansion parameter. The model output is overlapped with the niche map (EI>0).*

- **Radial rate of range expansion per year**

Relevant information and data

The initial introduction of A. chinensis to Italy (Lombardy at approximately N45°00; E09°00) may have happened 10-20 years before the pest was first discovered in 2001 and it is very likely that repeated introductions occurred

The furthest boundary of an infested municipality was approximately 28km from the site where the outbreak was first discovered in the spring of 2007 (see map below). This would be in accordance with the estimate of 1km/year (10-20 years before 2001 plus 6 years until 2007 -> 28km /16years or 28 km/26 years). However, An eradication programme in place shortly after the discovery of the pest in 2000 and it is not known how far the pest would have spread without this programme.

c was tested with 1 km/year and also with 2 km/year.

- **Time frame t**

t = 30 – 60 years

- **Entry point(s) or simulation of an introduction at a place (*coord*)**

Northern Italy: coord (9,45); France (0,50); Netherlands (8,53)

**
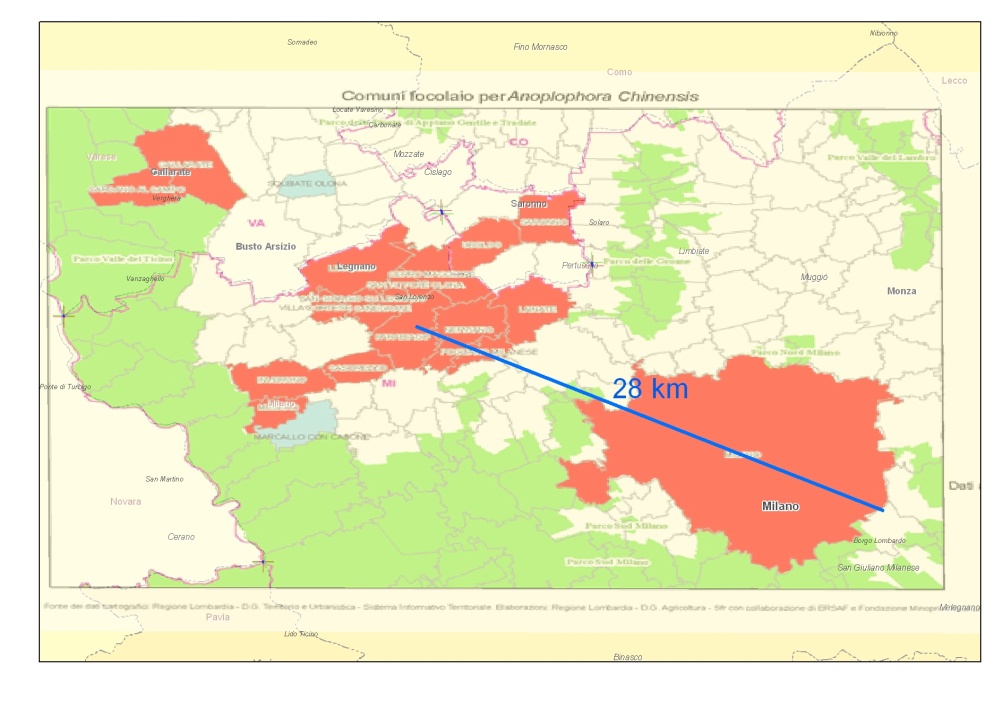
**

**Figure 2-5**: Infested area with *Anoplophora chinensis* in Lombardia Italy . Red Areas show the municipalities where the Citrus longhorn beetle is present; in blue marked areas only adult beetles were found. Infestation was first observed near Milano in 2000 and this map shows the presence of the pest in 2007 (source: http://www.eppo.org/QUARANTINE/anoplophora_chinensis/chinensis_IT_2007.htm) .

**Results**:

Radial range expansion after 30 years with one entry point in Northern Italy


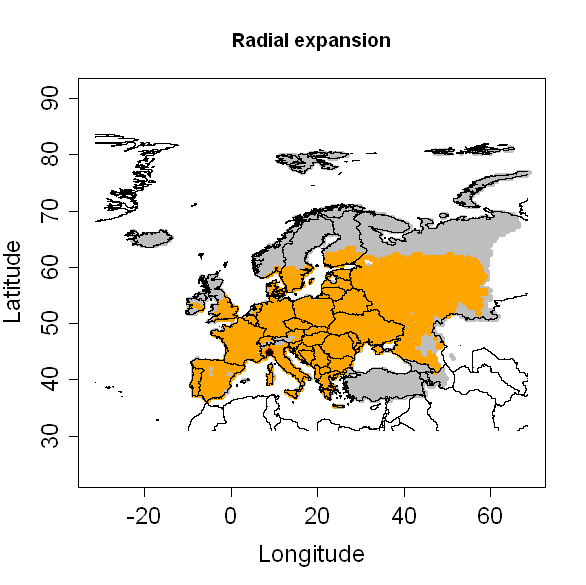


**c = 2 km, t = 30 years**


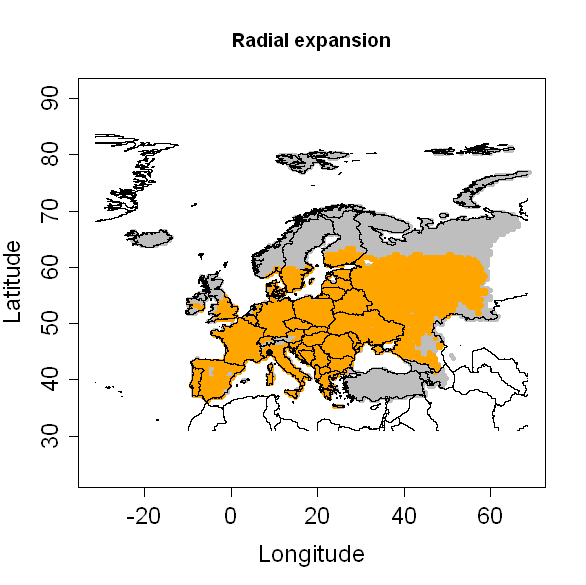


**c = 1 km, t = 30 years**

**Figure 2-6:** Radial range expansion of *A. chinensis* for t=30 years, an entry point in Northern Italy. Left picture: c = 1 km /year; right picture: c = 2 km/year; (red dots: invaded cells, orange dots: non invaded but suitable cells, grey dots: EI=0, white: no data).

Radial range expansion after 60 years with one entry point in Northern Italy


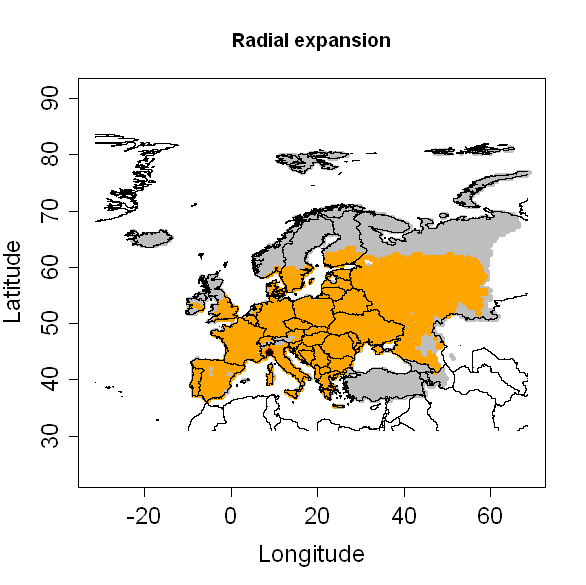


**c=1 km, t=60 years**


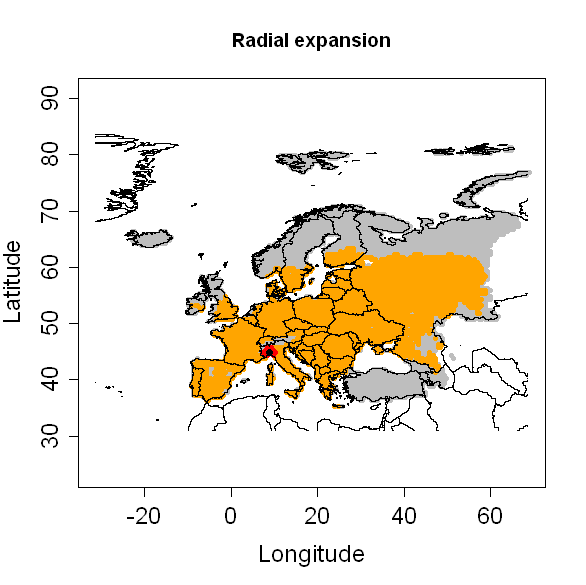


**c=2 km, t=60 years**

**Figure 2-7**: Radial range expansion of *A. chinensis* for t=60 years, an entry point in Northern Italy. Left picture: c = 1 km /year; right picture: c = 2 km/year; (red dots: invaded cells, orange dots: non invaded but suitable cells, grey dots: EI=0, white: no data).

Radial range expansion after 60 years with two and three entry points


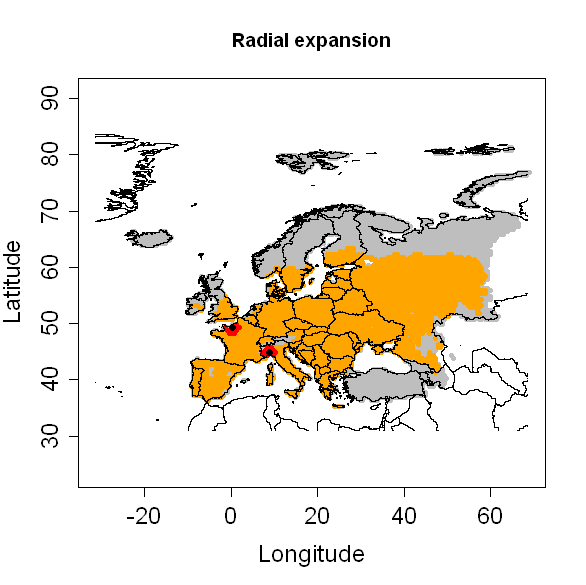


**c = 2 km, coord = (9,45,0,50), t = 60**


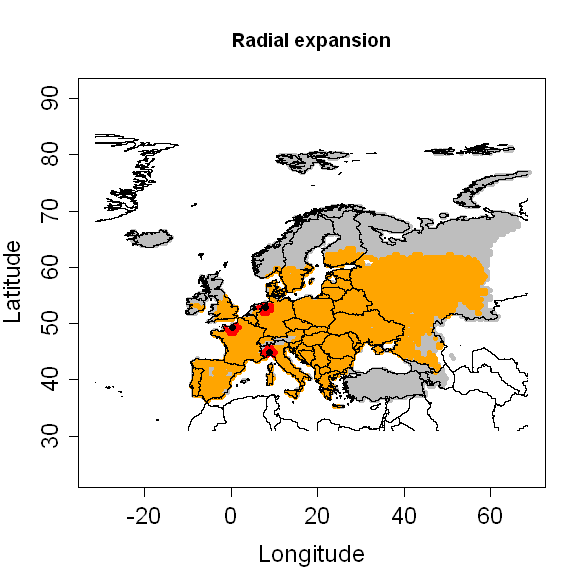


**c=2 km, coord = (9,45,0,50,8,53), t= 60**

**Figure 2-8**: Radial range expansion of *A. chinensis* for t=60 years, two entry points (left picture: Northern Italy and France) and three entry points (right picture: Northern Italy, France and Netherlands). c = 2 km/year; (red dots: invaded cells, orange dots: non invaded but suitable cells, grey dots: EI=0 or host absent, white: no data).

**Model D: Deterministic version of the dispersal kernel model**

*This type of model combines a population growth model and a dispersal kernel (2Dt) and it considers the proportion of the population that is engaged in dispersal.*

- **Starting population *p_0_***

*p_0_ is the population abundance for all suitable cells at time t = 0 expressed as a percentage of the maximum abundance (carrying capacity P_max_).*

p_0_ = 1.0 x 10^-6^ % - 1.0 x 10^-5^ %

- **Multiplication factor *λ_max_***

*λ_max_ is the maximum year to year multiplication factor (“finite growth rate”) that a population could achieve under optimal conditions assuming unlimited space*

λ_max_ = 6

- **Shape parameter (**υ**) of the 2Dt dispersal kernel**

*(number of degrees of freedom or the proportion of the population engaged in long or short distance dispersal)*

*For* υ *= 1, this kernel has a Cauchy distribution (thick tail; a large number of individuals disperses further than 3σ) and for* υ$\to+\infty$*, it has a normal distribution (thin tail; individuals disperse at short distance).*

*
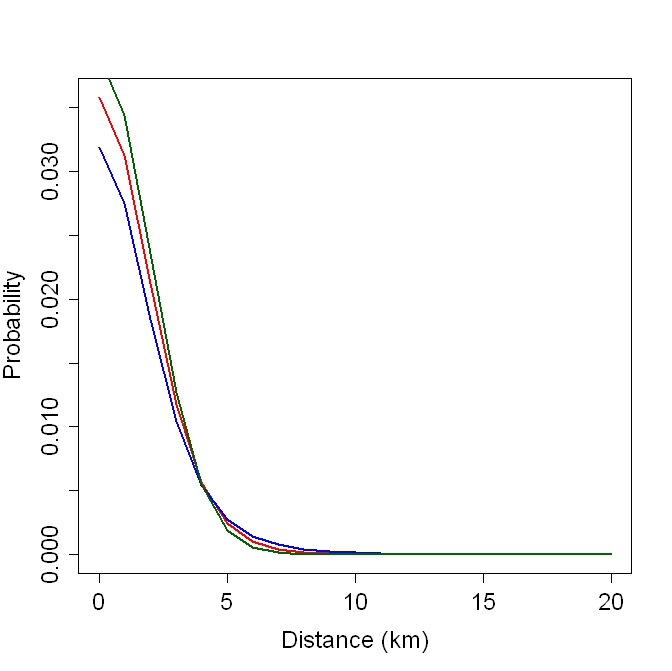
*

u = 2 km

υ = 50

υ = 10

υ = 5

**Figure 2- 9**: Dispersal kernel with υ = 50 (green graph, short distance spread), υ = 10 (red graph) and υ = 5 (blue graph, long distance spread) and u = 2 km.

Based on the information on *A. chinensis* we assume that only a small number of beetles will be engaged in long distance dispersal (see above under natural spread). We therefore do not use a “small” υ (e.g. 1 or 5) but test the Dispersal Kernel Model with a “medium” υ and compare υ=10 and υ=50.

- **Scale parameter (u) of the 2Dt dispersal kernel for the distance in km**

Here we use u = 2 km

**Presence file**

*The presence file contains the coordinates for the entry point and p_0_.*
In this case the average p_0_ = 5.5x10^-6^ was used.

**Results:**


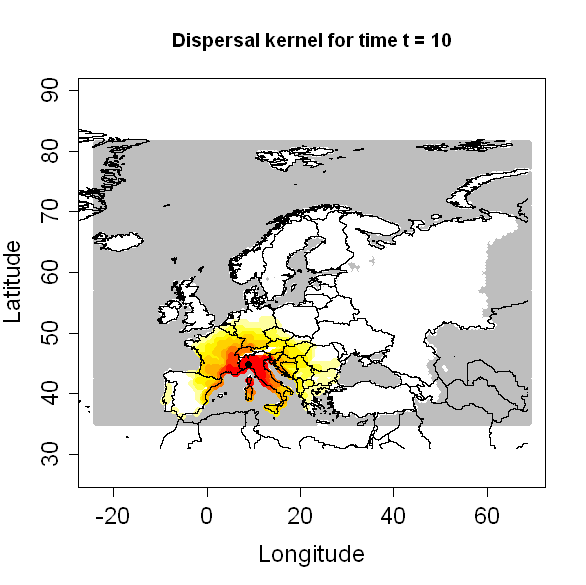
Results for υ=10

λ**max =6,** υ **=10, u =2, t =10**

**Figure 2-10**: Output of the dispersal kernel model for *A. chinensis.* p_0_ = 5.5*10^-6^; υ=10, u=2, t=10 years (pt: population abundance (%); graduated colors from white (p_t_ < 10^(-6) %) to yellow, orange and red (p_t_ > = 10%). Grey means no data)


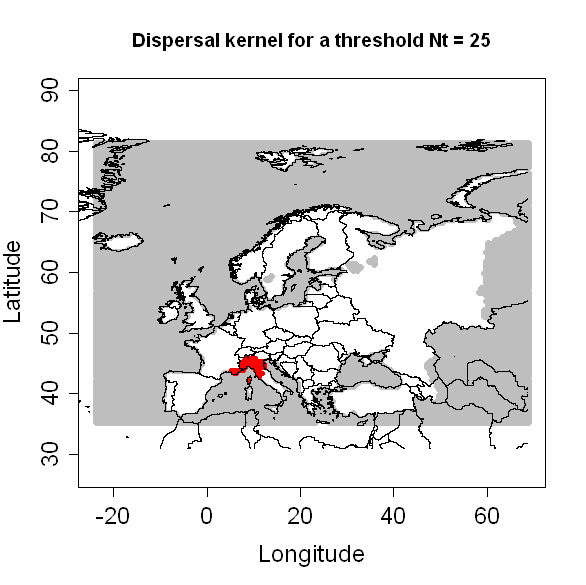


λ**max =6,** υ**p =10, u =2, t =10**

**Figure 2-11**: Output of the dispersal kernel model for *A. chinensis* (figure 2-10) with a threshold = 25%.
p_0_ = 5.5*10^-6^; υ=10, u=2, t=10 years. The population abundance (%) is above this threshold in red cells. (red dots: cells where p_t_ > = threshold, white: cells where p_t_ < threshold. Grey means no data)

Results for υ=50


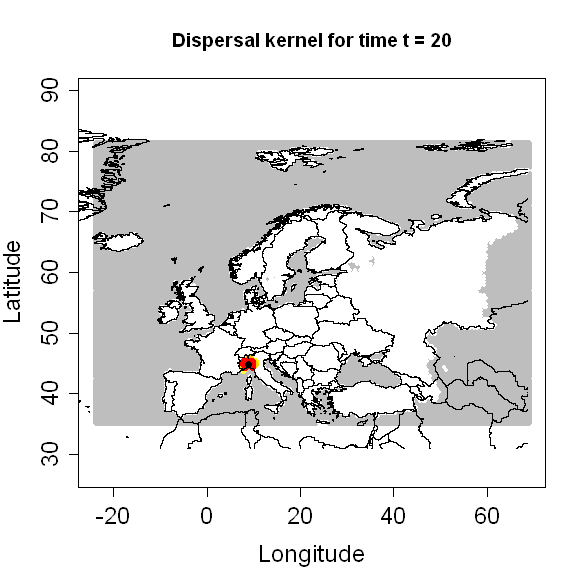


λ**max =6, υ =50, u =2, t =20**


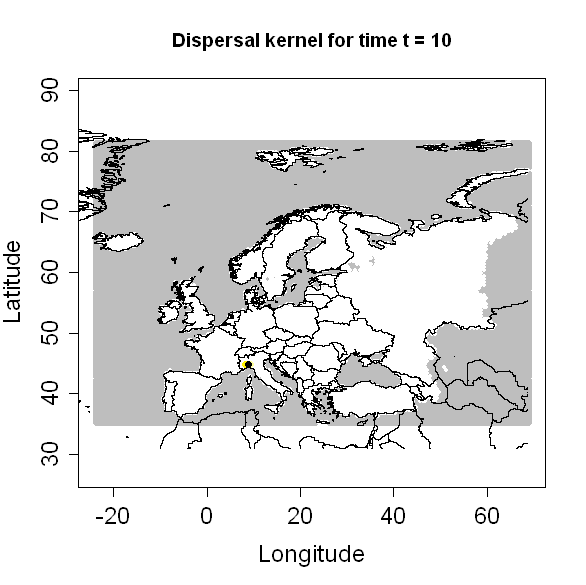


λ**max =6, υ =50, u =2, t =10**

**Figure 2-12**: Output of the dispersal kernel model for *A. chinensis*; p_0_ = 5.5*10^-6^; υ=50, u=2, t=10 years (left picture) and 20 years (right picture) ; p_t_: population abundance (%); graduated colors from white (p_t_ < 10^(-6) %) to yellow, orange and red (p_t_ > = 10%). Grey means no data.


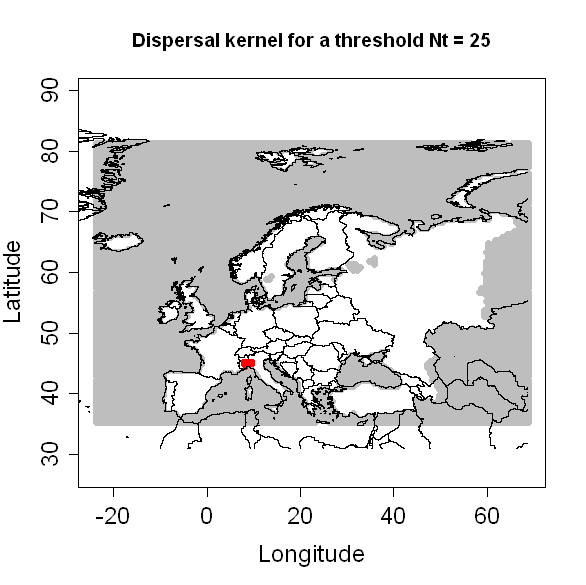


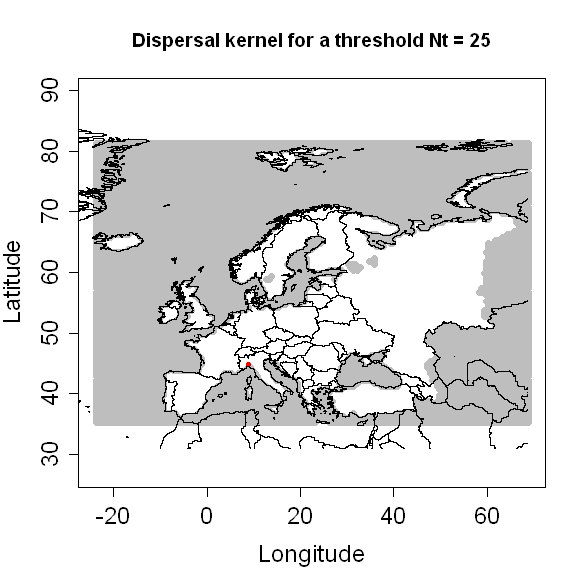


λ**max =6, υ =50, u =2, t =10**

λ**max =6,** υ **=50, u =2, t =20**

**Figure 2 -13**: Output of the dispersal kernel model for *A. chinensis* (figure 2-12) with a threshold = 25%. p_0_ = 5.5*10^-6^; υ=50, u=2, t=10 years (left picture) and 20 years (right picture) . The population abundance (%) is above this threshold in red cells (red dots: cells where p_t_ > = threshold, white: cells where p_t_ < threshold. Grey means no data).

**Summary**

The application of the generic spread models to the Citrus longhorn beetle showed that the spread of the beetle could be mapped with reasonable results by model B (the Radial Range Expansion Model) and model D (the Dispersal Kernel Model). According to literature and data from China and Italy the beetle disperses slowly by natural means and this was clearly displayed by the resulting maps, showing the areas at risk.

Additional information is provided by model C (the Simple Logistic Model). Due to the assumptions underlying this approach that every suitable grid cell is invaded at the same time the result shows the temporal spread within cells. It therefore shows clearly which areas are expected to have high beetle abundances after different time intervals providing useful information for identifying locations where the highest impacts are to be expected.

Model B (the Radial Range Expansion Model) requires few parameters (starting population, radial range expansion per year and starting point(s) for the infestation). For *A. chinensis*, these could be derived from the literature and outbreak data from Italy. The application of this model approach is straightforward and the assisting experts felt quite comfortable with its outcome.

Model D (the Dispersal Kernel Model) also leads to reasonable results, which are more visible by mapping the area above a defined threshold (p_t_) for the abundance after a specific time (t). However, in this case, the results depend on the choice of the shape parameter (υ) of the 2Dt kernel. The application therefore requires some testing and "playing" with the model" and in the future more guidance on the most appropriate values for the given species (and how to derive them) should be provided.

Feedback from experts after reviewing the case study

The Radial Range Expansion per year (Model B) was felt to result in more sensible results compared to the output of the Dispersal Kernel (Model D) which from the point of view of the experts results in a faster spread than they would have expected from their experience. The dependency on the chosen parameters is seen to be more critical for the Dispersal Kernel Model since this more sophisticated approach needs more testing to select the most appropriate parameters (e.g. the shape parameter υ).

**Summary of the commands for modelling the spread of** *Anoplophora chinensis*

library(sp)

library(raster)

library(rgdal)

elevmax = F

hostfile = T

*# load the R code*

plothost()

plotRA()

plotGI()

res = slg(N0=1.0*10^(-5),lmax=6,movie=F, t=20)

res = radial(RR=2,t=60,coord=c(9,45,0,50),figkm=T, figdd=F)

res = dispk(N0=NULL, t=10,lmax=6, p=50,u=2, presencefile=T, nentry=NULL, figkm=F,figdd=T)

plotkernel (res$dispk,threshold=25)

**References**

Baker, R. and D. Eyre (2006) Pest Risk Analysis for *Anoplophora chinensis*. CSL, York, UK. (unpublished, internal CSL document).

De Boer, D. (2004) Distribution potential of three *Anoplophora* species – A CLIMEX study. Plant Protection Service, Section Entomology, Wageningen, The Netherlands, 49 pp.

EPPO (2011) EPPO Reporting Service No. 9, September 2011.

EPPO website on *A. chinensis*. [Accessed on 12. January 2011]. (<http://www.eppo.org/QUARANTINE/anoplophora_chinensis/chinensis_IT_2007.htm>

Haack RA, Hérard F, Sun J, Turgeon, JJ (2010) Managing invasive populations of Asian longhorned beetle and citrus longhorned beetle: a worldwide perspective. Annual Review of Entomology. 55: 521-546.

Päivinen, R., M. Lehikoinen, A. Schuck, T. Häme, S. Väätäinen, P. Kennedy, P. & S. Folving (2001) Combining Earth Observation Data and Forest Statistics. EFI Research Report 14. European Forest Institute, Joint Research Centre - European Commission. EUR 19911 EN. 101p. [EFI Research Report 14](http://www.efi.int/portal/virtual_library/publications/research_reports/14/). Available online at: <http://www.efi.int/portal/virtual_library/publications/research_reports/14/> , accessed on 21st December 2011.

Van der Gaag, D-J., M. Ciampitti, B. Cavagna, M. Maspero and F. Hérard, 2008: Pest Risk Analysis for *Anoplophora chinensis*. Plant Protection Service, the Netherlands. http://edepot.wur.nl/117610 [Accessed on 21. December 2011].

Van der Gaag, D. J., G. Sinatra, P. F. Roversi, A. Loomans, F. Hérard and A. Vukadin, 2010: Evaluation of eradication measures against *Anoplophora chinensis* in early stage infestations in Europe. Bulletin OEPP/EPPO Bulletin 40, 176–187.

**3. Asian Longhorn Beetle, *Anoplophora glabripennis***

Authors: Hella Kehlenbeck (JKI, Germany), Massimo Faccoli and Andrea Battisti (both UPAD, Italy)

**Information on *A. glabripennis***

Natural spread potential

Adult beetles are capable of flying several hundred metres or more in a single flight to locate host trees. *A. glabripennis* tends to fly farther to find suitable host trees when no host trees are present in the surrounding area, whereas, when host trees are densely planted, little dis­persal by adult *A. glabripennis* occurs (Huang, 1991).

Mark-release-recapture experiments showed that adults of the Asian longhorn beetle can disperse 1 to 3 km during their life span, although most remain near the tree where they emerge (Bancroft & Smith, 2005; Smith et al. 2001, Smith et al., 2004). Smith et al. (2004) recaptured 98% of the marked beetles within 920 m from the release point.

The average annual population dispersal distance measured by Wen et al. (1998) was 106.3 m, and this dispersal was positively correlated with wind velocity and temperature. However, a mean dispersal distance of 266 m has been recorded using the mark–release–recapture method (Smith et al., 2001). Further mark–release–recapture studies demonstrated that, although 72% of beetles were recaptured within 300 m of release points, some beetles were recaptured up to 2600 m away (Smith et al., 2004). In these studies, beetle dispersal from release trees was positively associated with the abundance of beetles at the release tree and smaller female beetles moved greater distances and were more attracted to taller trees (Bancroft & Smith, 2005).

A study of the outbreak in New York / New Jersey suggests that a low density introduction of *A. glabripennis* occurred and remained localized and undetected for many years and spread slowly until the beetle density on hosts became unsustainable at which time adults dispersed hundreds of metres to over 1 km (Sawyer, 2007). Sawyer & Panagakos (2008) reported estimated dispersal distances of ALB at three infested areas in New York and New Jer­sey of 2.25 km in 7 years, 2.25 km in 5 years and 1.6 km in 4 years leading to a medium range of 0.4 km per year. If suitable host trees are abundant, a population of ALB may re­main highly localized for years, reproducing on few trees in a limited area (a few hundred metres in radius).

In *A. glabripennis* infested areas in Europe; all infested trees were found within an area with a ra­dius of 200 – 500 m at the end of 2004 (Hérard et al., 2005). The infestation size in Cornuda (Northern Italy) was detected in June 2009, but dendrochronological studies carried out on infested trees indicate that the infestation begun at least since summer 2004 (first exit holes date from summer 2005) (Faccoli, experience from Italy). In October 2009 the infestation area covered about 10.37 km^2^. Assuming a constant linear radial dispersal from the inoculation point, in the five years (2004-2009) before the application of the eradication protocol, the recorded infestation area was covered by an adult dispersal radius of about 300-350 m per year.

These observations indicate that beetles do not fly over long distances in these areas. A study of *A. glabripennis* infestations in New Jersey suggests that a low density introduction of these beetles remains localized for many years and spread is slow until, at some point, the local resource becomes over-exploited, at which time dispersal from hundreds of metres to over 1 km occurs (Sawyer, 2007). Beetles will possibly fly over longer distances at high population densities or low host plant densities. Mark–release–recapture studies demonstrated that, although 72% of beetles were recaptured within 300 m of release points, some beetles were recaptured up to 2600 m away (Smith *et al*., 2004).

*A. glabripennis* is likely to spread locally in the short term, especially where host trees are abundant. Development is slow; it may take one or more years to complete a generation. It would take many years (decades) for *A. glabripennis* to spread by natural means over much of Europe.

Human-mediated spread potential, which is the most important pathway for long distance dispersal of *A. glabripennis*, is not considered by the modeling approach used here.

Host range and host distribution

*Anoplophora glabripennis* can adapt to feed and develop on many tree species. In Asia, North America and Europe various species from many different families have been reported (Lingafelter & Hoebeke, 2002; Hérard *et al*., 2006; Sawyer 2008). The main hosts include species from *Acer*, *Populus*, *Salix* and *Ulmus* (Lingafelter & Hoebeke, 2002; Williams *et al.,* 2004; Haack, 2006).

The complete development of *A. glabripennis* in Europe has been recorded on *Acer, Aescu­lus, Alnus, Betula, Carpinus, Fagus, Fraxinus, Platanus, Populus, Prunus, Salix* and Sorbus with *Acer* being the most commonly infested genus followed by *Betula, Salix, Aesculus* and *Populus* (Hérard et al. 2006; 2009). In a risk analysis of 1997 MacLeod (1997) reported the following species as hosts: *Acer dasycarpum –* silver maple, *A. negundo* – box elder, *A. pla­tanoides* – Norway maple, *A. pseudoplatanus* – sycamore, *A. saccharinum* – silver maple, *A. saccharum* – sugar maple, *A. trunctatum* – a maple in N. China, *Aesculus hippocastanum* – horse-chestnut, *Morus alba* – white mulberry. Within Europe *Acer* species, especially *A. ne­gundo*, *A. platanoides* and *A. pseudoplatanus*, as well as some *Populus* ssp., *Salix* ssp. and *Aesculus hippocastanum* are widely spread.

*Anoplophora glabripennis* is highly polyphagous and its major hosts are present in many European countries in urban areas. Hosts also occur widely in the natural environment. Some hosts are grown across large areas for forestry or to a lesser extent within mixed and natural forests. According to Schröder et al. (2006) all broadleaved trees (including fruit trees) are potential hosts of *A. glabripennis*. We therefore used the percentage of land cov­ered by broadleaf forest for the spread modelling (see figures 3-1 and 3-2).


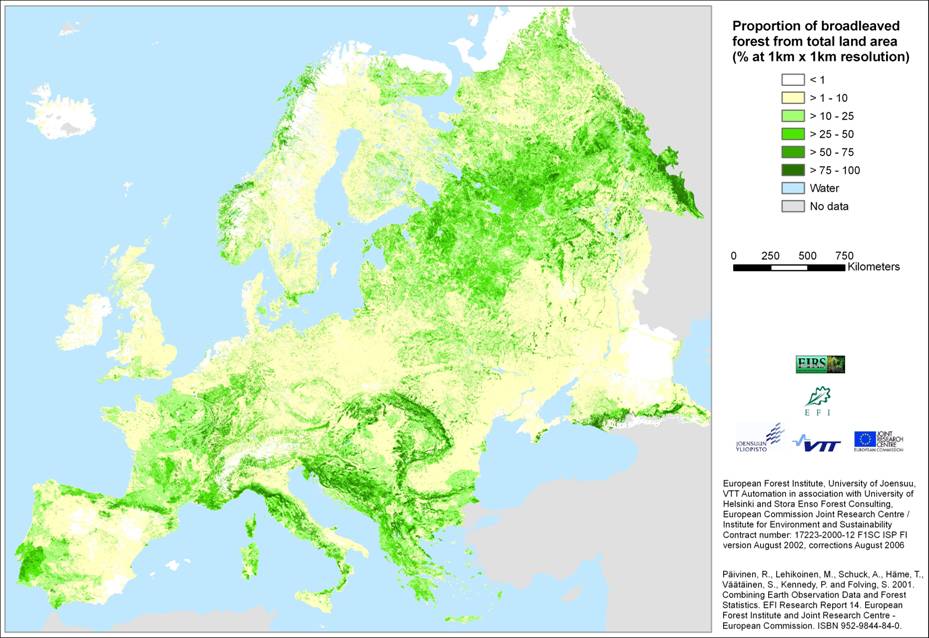


**Figure 3-1:** Proportion of broadleaved forest from total land area (% at 1 km by 1 km resolution; Päivinen et al. 2001).


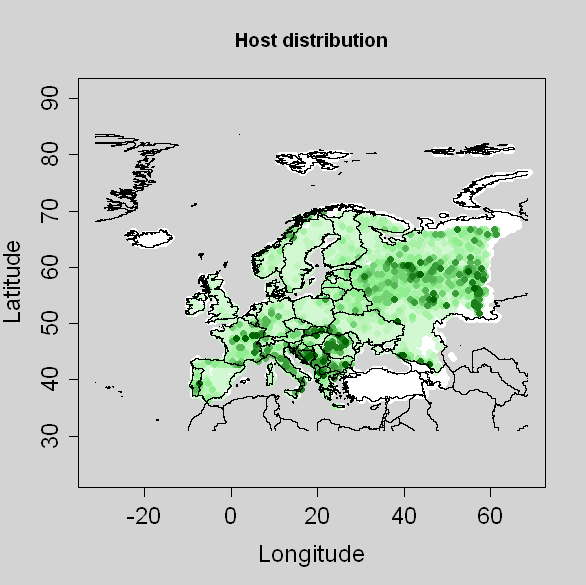


**Figure 3-2:** Host plant distribution for *Anoplophora glabripennis* in Europe based on the percentage of area covered with broadleaved forest according to Päivinen et al. 2001 (graduated colors from white (no host) to dark green (max=91%). Grey means no data).

Findings of *A. glabripennis* in Europe
(to be used as starting points for the modeling; source: EPPO reporting service, different years; coordinates for the locations were collected from the Internet/Google Earth)

Austria

- First finding in 2001 in Braunau am Inn (48°15'53''N,13°2'11''E (48.27,13.04))

France

- first finding in 2003 in Gien (Loiret) [47°41′N, 2°38′E](http://toolserver.org/~geohack/geohack.php?pagename=Gien_(Loiret)&language=de&params=47.688888888889_N_2.6294444444444_E_dim:20000_region:FR-45_type:city(15442)&title=Gien) (47.68,2.63))

- 2004 finding in Saint-Anne-sur-Brivet (Loire-Atlantique, Western France)
 47°27‘38‘‘N, 2°0‘8‘‘W (47.46,2.002)
- 2008 finding in Strasbourg (Hérard, et al. 2009)

Germany

- first finding in 2004 in Neukirchen am Inn (near Passau, Bavaria, Southern
 Germany), 48°31’N,13°22’E (48.51,13.37)

- 2005 finding in Bornheim (near Bonn, Northrhine-Westfalia, Western Germany)
 [50°46′N,7°0′E](http://toolserver.org/~geohack/geohack.php?pagename=Bornheim_(Rheinland)&language=de&params=50.759166666667_N_7.005_E_region:DE-NW_type:city(48544)) (50.77,7.0)

Italy
- first finding in 2007 in Corbetta (Lombardia region, near Milano) [45°28′0″N, 8°55′0″E](http://toolserver.org/~geohack/geohack.php?pagename=Corbetta_(Lombardei)&language=de&params=45.466666666667_N_8.9166666666667_E_dim:10000_region:IT-MI_type:city(16889))
- in 2009 in Cornuda (Veneto region; 45°50'N, 11°59'E (45.83,11.98))

Netherlands

- First finding in 2010 in the municipality of Almere, [52°23′N, 5°13′E](http://toolserver.org/~geohack/geohack.php?pagename=Almere&language=de&params=52.376388888889_N_5.2227777777778_E_region:NL-FL_type:adm2nd(188209)) (52.38,5.22)

Climatic suitability in Europe

MacLeod *et al.* (2002) reported that *A. glabripennis* would find the climate in many regions of Europe suitable for establishment, if biotic factors were appropriate. Detailed modelling using CLIMEX (by Dominic Eyre, 2010, see fiig. 3-3) also suggests that large areas of Europe provide suitable climatic conditions for the development of *A. glabripennis.* A complete generation could develop within 1 year in part of the southern EU, whilst development over much of central and northern EU could take place within 2 - 3 years. The occurrence of outbreaks in Austria, France, Germany and Italy confirms establishment is possible in southern and central EU. Further north in Europe populations may be transient, depending on sufficient summer temperatures. Given that there has been an ongoing out­break in Austria, perhaps since 2000, and in France, possibly since 2003, and outbreaks in Germany and Italy, and findings in live trees more recently in the Netherlands (2010), there is no doubt that climate in Europe is suitable for *A. glabripennis* development and that the beetle could establish and spread within the EU.

ALB shows a developmental threshold of 13.4°C (Yang *et al*., 2000). Under laboratory conditions, however, egg, first and second instar larvae showed a lower development temperature of 10.2°C (Zhang *et* *al*., 1995; Keena and Moore, 2010). Roden et al (2008) exposed larvae to low temperatures of -25°C to -40°C for 24 hours and observed that a high percentage of larvae (95%) survived the treatment and that, of those, 25 to 45% even completed their development and mated. The authors assume that ALB is freeze tolerant and that the northern distribution of the beetle will not be limited by winter temperatures but by host availability and summer temperatures.

CLIMEX file

A CLIMEX model for *A. glabripennis* was provided by Dominic Eyre (Fera, UK). Figure 3-3 shows the risk area and the growth potential for A*. glabripennis* according to this model.

Reproduction strategy

The majority of the area where establishment is possible in the EU suggests that develop­ment would actually take 2 years (in contrast to the situation in China). This is due to the summer temperatures of Europe generally being cooler than in China, although this will vary depending on which locations are compared. The number of generations per year will have an influence on the reproduction rate and population densities. With a two or three year development cycle, the population will not grow as fast compared to a one-year-life-cycle. However, in 2003 a shortened life cycle of 1.5 years in Germany and elsewhere in Europe was reported due to the hot summer temperatures (T. Schröder, JKI, personal communi­cation).

In the Italian infestation of Cornuda, considering a developmental threshold of 10.2° C, the Accumulated Degree Day (ADD) of 1264.2 needed for the whole ALB development (Yang *et al*., 2000) calculated in the last 19 years (1992-2011) from September first to end of August of the following year was always reached, suggesting a monovoltine cycle. Differently, considering a developmental threshold of 13.4° C, the ADD of 1264.2 was reached within the following September only by eggs laid within the end of July of the previous summer. Eggs laid in late summer show life cycle of 1.5 years (Faccoli, unpublished).

Topography or elevation limits

T. Schröder (personal communication) considers the Alps to be a natural barrier. We there­fore applied an elevation limit for *A. glabripennis* of 2000 m.


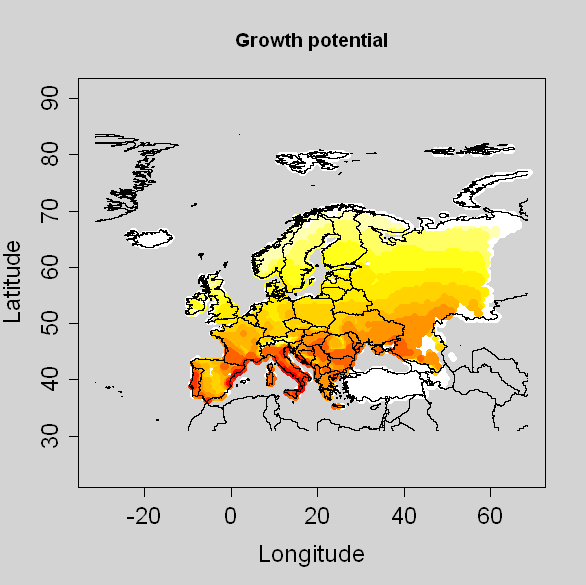

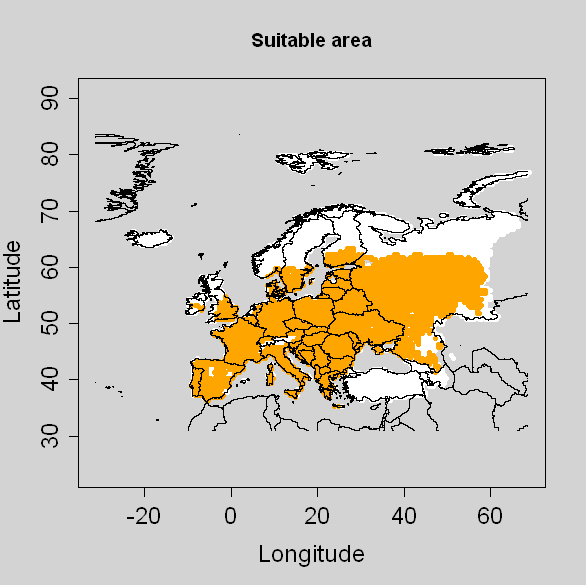


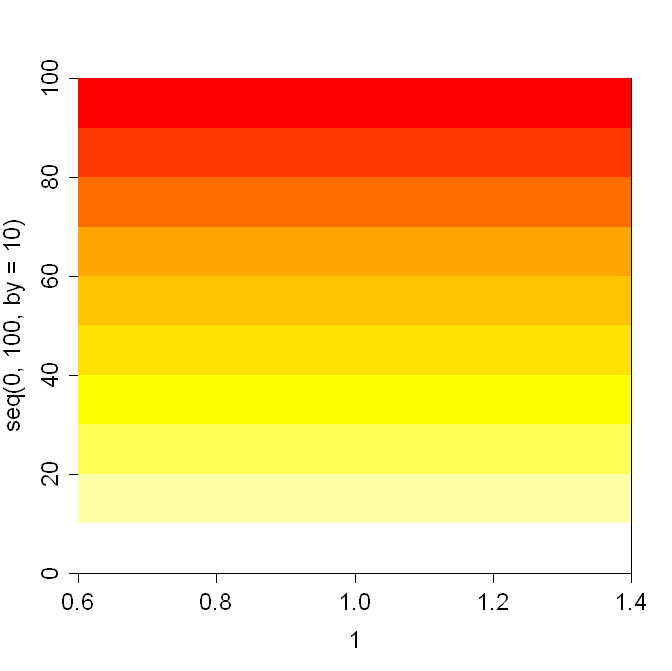


**Figure 3-3:** Suitable area (on the left; orange dots: EI>0, white dots: EI=0, grey: no data) and growth potential (GI; on the right) for *A. glabripennis* in Europe according to a CLIMEX model provided by Dominic Eyre. (EI = Ecoclimatic Index indicates, how favourable the climate is for the long term sur­vival of the species; GI=Growth Index indicates the overall potential for the population growth).

**Model C:** Population dynamics model

*For this model we assume that an initial population abundance p_0_ (%) is introduced in each suitable cell (cells where EI > 0) and then we simulate the “spread” or more precisely the growth within each cell according to a logistic function. The output map shows the areas which are the most suitable for population growth assuming introduction.*

- **Starting population *p_0_***

*p_0_  is the population abundance for all suitable cells at time t = 0 expressed as a percentage of the maximum abundance (carrying capacity P_max_).*

*Calculation:*

$p_{0}=100*\frac{P_{0}}{P_{\max}}$ *with P_0_ the number of introduced individuals, in each suitable cell, and P_max_ the carrying capacity (the maximum number of individuals in a cell).*

*P_max_ = area_cell (km²) * proportion_covered_by_host * max_population_density (/km²), or
P_max_ = area_cell (km²) * host_plant_density (/km²) * max_population_density (/host plant)*

Relevant information and data

In Italy, from a rough visual assessment carried out on 129 trees infested in 2009-2010, a mean population density of about 33 exit holes per infested tree (ranging from 1 to more than 50 per tree) was found (Battisti, experiences from Italy).

However, these numbers maybe much higher in single cases: Hérard et al. (2009) reported one sycamore maple tree (8 m tall with a triple trunk) in Italy (2007, Corbetta) that permit­ted 339 individuals to develop. At the same location three birches were found with together 32 exit holes and 74 living larvae, leading to about 100 individuals on these three *Betula* trees.

In the Netherlands (in November 2010) 16 exit holes and three larvae were detected on one *Acer* tree.

We assumed the maximum population density (max_population_density) = 10-40 beetles/host tree (Faccoli and Battisti experiences from Italy).

Host trees and shrubs per km²

In Italy, on a 5 x 5 km grid there are about 12,000 suitable trees (12,494 suitable trees found over about 26 km², according to Faccoli and Battisti), which means about 480 host trees per km² (12,000 trees / 25 km² = 480 host trees / km²).

Calculation of the capacity P_max_:

P_max1_= 10 beetles/host tree x 480 hosts/km² x 1579 km² (grid cell)

P_max1_ = 7.579.200 = 7.58 x10^6^ beetles/grid cell

P_max2_ = 20 beetles/host tree x 480 hosts/km² x 1579 km² (grid cell)

P_max2_ = 15.158.400 = 1.5 x 10^7^ beetles/grid cell

Calculation of p_0_

Starting number of beetles (= P_0_) : can be very low, just 2 beetles (one female and one male) per gird cell could be sufficient

p_0_ = 100 x starting number n_0_/K = %

p_0_ = 100 x (2 beetles per grid cell / 7.6x10^6^ beetles/grid cell) -
 100 x (2 beetles per grid cell /1.5x10^7^  beetles per grid cell

p_0_ = 2.6 x 10^-5^ % to 1,3 x 10^-5^ %

- **Multiplication factor λ_max_**

*λ_max_ is the maximum year to year multiplication factor (“finite growth rate”) that a population could achieve under optimal conditions assuming unlimited space*

Relevant information and data

In Northern Italy within 5 years the number of infested trees reached 430 (Battisti, pers. comm.).

Given that two beetles (one male and one female) were the initial population, and assuming, that there have been a mean density of 10 – 20 beetles/tree, the multiplication factor over these 5 years would have been 2150 – 4300. The multiplication factor for one year (*λ_max_)* can be calculated in the following way:

*λ_max_* = 2150^1/5^ – 4300^1/5^

*λ_max_* = 4.6 – 5.3

*λ_max_* = 5

- **Time frame *t***

t = 20 - 30 years

**Results for Model C:**

Figure 3-4 shows the results of the Population dynamics model for 20 and 30 years and two different values for p_0_.


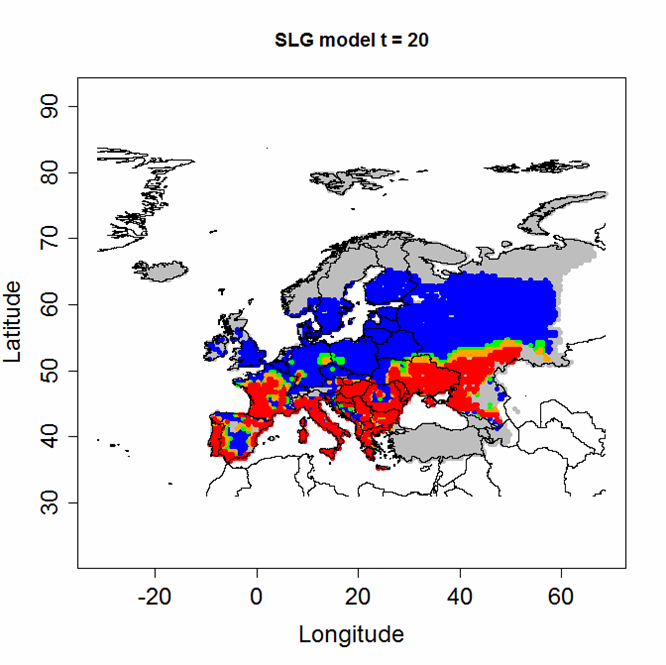

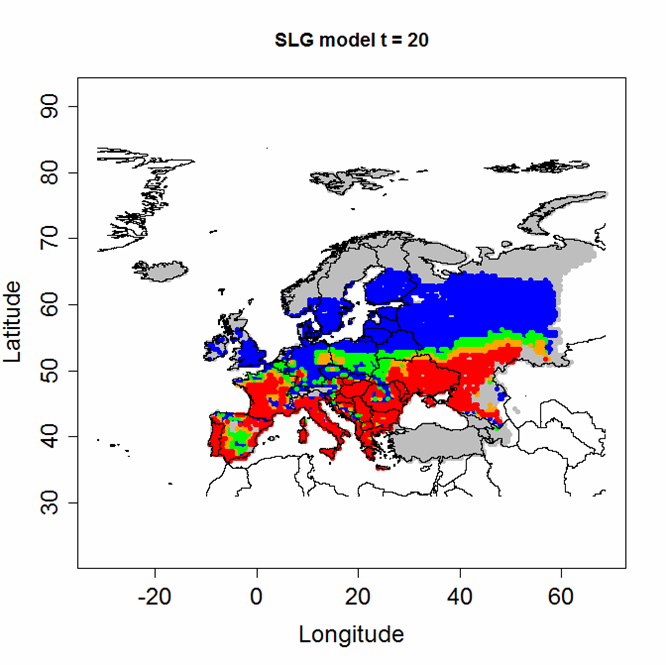


p_0_= 2.6 x10^-5^; *λ_max_*= 5

p_0_= 1.3 x10^-5^; *λ_max_*= 5


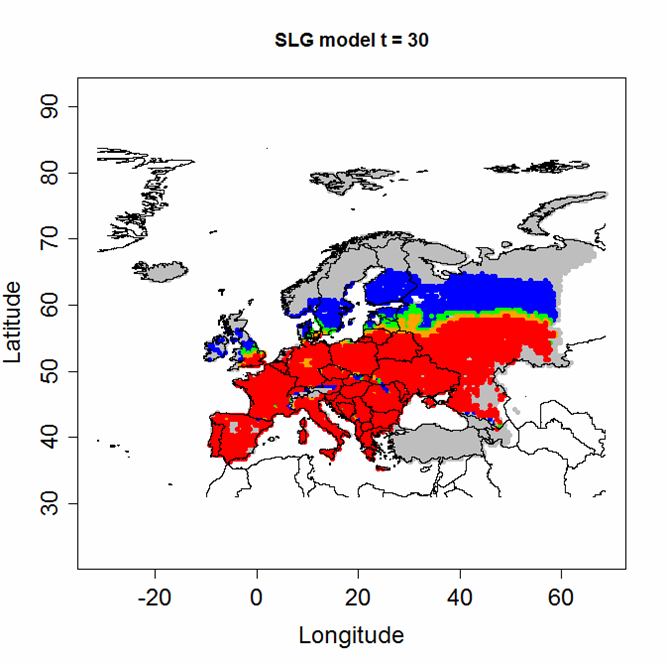

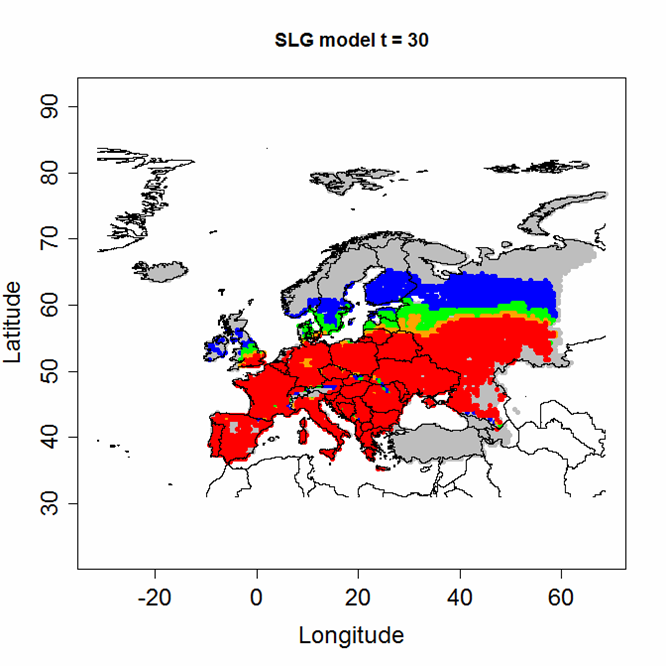


p_0_= 2.6 x10^-5^; *λ_max_*= 5

p_0_= 1.3 x10^-5^; *λ_max_*= 5

**Figure 3-4:** Output of model C for *A. glabripennis* for 20 and 30 years ( blue dots: 0 < p_t_ < = 25, green dots: 25 < p_t_ < = 50, orange dots: 50 < p_t_ < = 75, red dots: 75 < p_t_ < = 100, grey dots: p_t_=0, white: no data). Left pictures: p_0_=2.6 x10^-5^; right pic­ture: p_0_=1.3 x10^-5^.

Information provided Model C: due to the assumptions underlying this approach that every suitable grid cell is invaded at the same time the result shows the tem­poral spread within cells. It therefore clearly shows which areas are expected to have high beetle abundances after different time intervals indicating where the highest impacts / consequences are to be expected.

**Model B: Radial range expansion model**

*This model aims to determine the potential spread of a species introduced in the PRA area based on the radial rate expansion parameter. The model output is overlapped with the niche map (EI>0).*

- **Radial rate of range expansion per year**

c = 1.5 km/year

Relevant information and data

Fight distances of ALB adults have been observed in the range of up to 3 km although most remain near the tree where they emerged. Smith et al. (2004) recaptured 98% of the marked beetles within 920 m from the release point, although a part of the released females were found over 2.6 Km. In infested areas of Europe, trees with symp­toms occurred within an area of a radius of 200-500 m, in the Cornuda area in Italy 300-350 m per year have been observed.

Sawyer & Panagakos (2008) assumed dispersal distances of ALB at three infested areas in the USA of 2.25 km in 7 years, 2.25 km in 5 years and 1.6 km in 4 years leading to medium range of 0.4 km per year. If suitable host trees are abundant, a population of ALB may remain highly localized for years, reproducing on few trees in a limited area (a few hundred meters in radius). If food resources decline beetles may disperse more widely.

The chosen value of 1.5 km/year is assumed to capture the range of distances between several hundred metres and more than 2 km.

- **Time frame *t***

t = 40 – 60 years

- **Entry point(s) or simulation of an introduction at a place (*coord*)**

Northern Italy (Treviso region): coord (11,46.15)


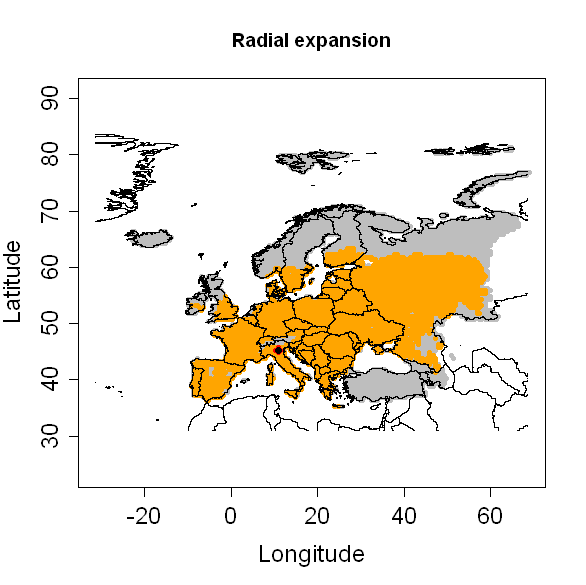
**Results for the Radial range expansion of *A. glabripennis* after 40 and 60 years**

**Figure 3-5**: Radial range expansion of *A. glabripennis* for **t=40 years**, an entry point in Northern Italy. c = 1.5 km /year (red dots: invaded cells, orange dots: non invaded but suitable cells, grey dots: EI=0 or host absent, white: no data).

*
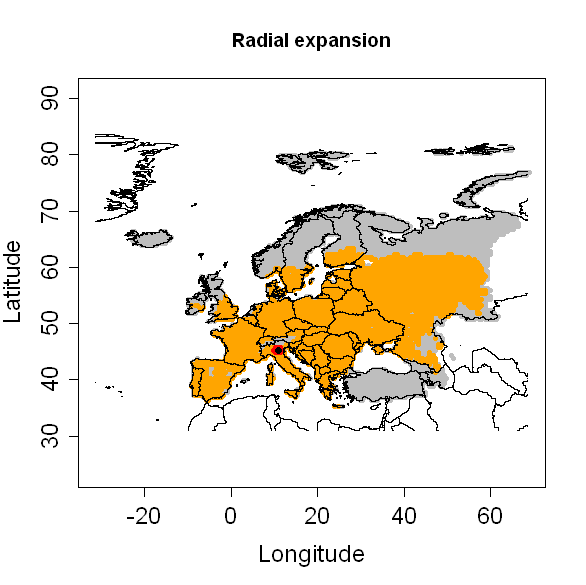
*

**Figure 3-6**: Radial range expansion of *A. glabripennis* for **t=60 years**, an entry point in Northern Italy. c = 1.5 km /year (red dots: invaded cells, orange dots: non invaded but suitable cells, grey dots: EI=0 or host absent, white: no data).

**Model D: Deterministic version of the dispersal kernel model**

*This type of model requires: a population growth model, a dispersal kernel (2Dt in this case) and the proportion of population engaged in dispersal.*

- **Starting population *p_0_***

p_0_ is the population abundance for all suitable cells at time t = 0 expressed as a percentage of the maximum abundance (carrying capacity *P_max_*).

p_0_ = 2.6 x 10^-5^ % - 1,3 x 10^-5^ %

- **Multiplication factor λ_max_**

*λ_max_ is the maximum year to year multiplication factor (“finite growth rate”) that a population could achieve under optimal conditions assuming unlimited space*

λ_max_ = 5

- **Shape parameter (p) of the 2Dt dispersal kernel**

*(number of degrees of freedom or the proportion of the population engaged in long or short distance dispersal)*

*For* υ *= 1, this kernel has a Cauchy distribution (thick tail; a large number of individuals disperses further than 3σ) and for* υ$\to+\infty$*, it has a normal distribution (thin tail; individuals disperse at short distance).*

*
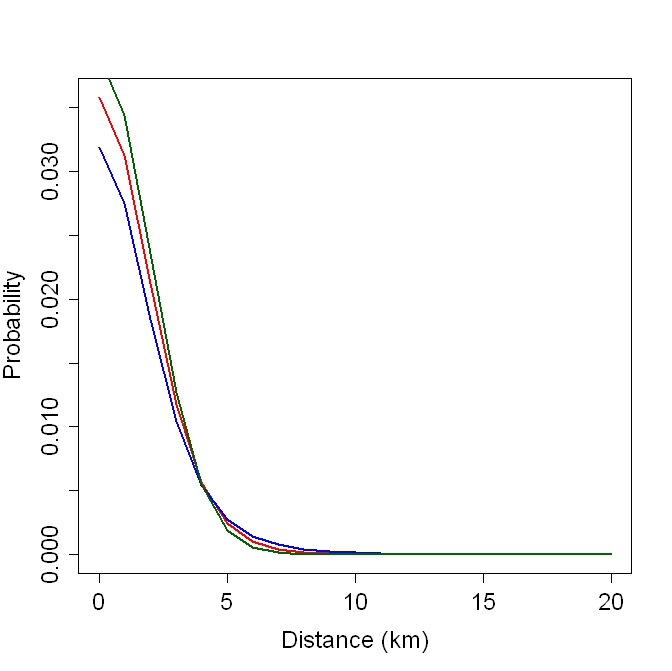
*

u = 2 km

υ = 50

υ = 10

υ = 5

**Figure 3-7**: Dispersal kernel with υ = 50 (green graph, short distance spread), υ = 10 (red graph) and υ = 5 (blue graph, long distance spread) and u = 2 km.

Based on the information on *A. glabripennis* we assume that only a small number of beetles will be engaged in long distance dispersal (see above under information on spread). We therefore do not use a “small” υ (e.g. 1 or 5) but test the Dispersal Kernel Model with a “medium” υ and compare p=30 and υ=50.

- **Scale parameter (u) for the distance in km of the 2Dt dispersal kernel**

Here we use u = 1.5 km (derived here from Model B)


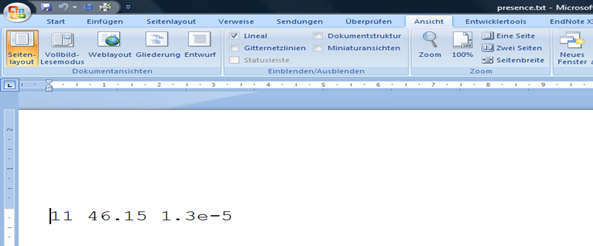


- **Presence file**

*The presence file contains the coordinates
 for the entry point and p_0_.*

**Results**

Results for υ=30

Figure 3-8 shows the results of the simulated spread of *A. glabripennis* over 20 years, starting in Northern Italy (coordinates see within the presence file) for υ =30 and a scale parameter u of 1.5 km.

Based on the estimated parameters, *A. glabripennis* would spread over Northern Italy and move further to the North including small areas of Switzerland and Southern Germany.


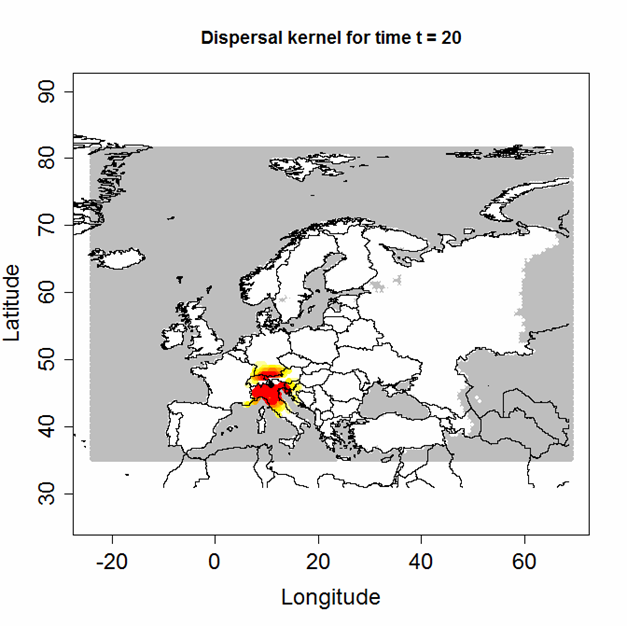

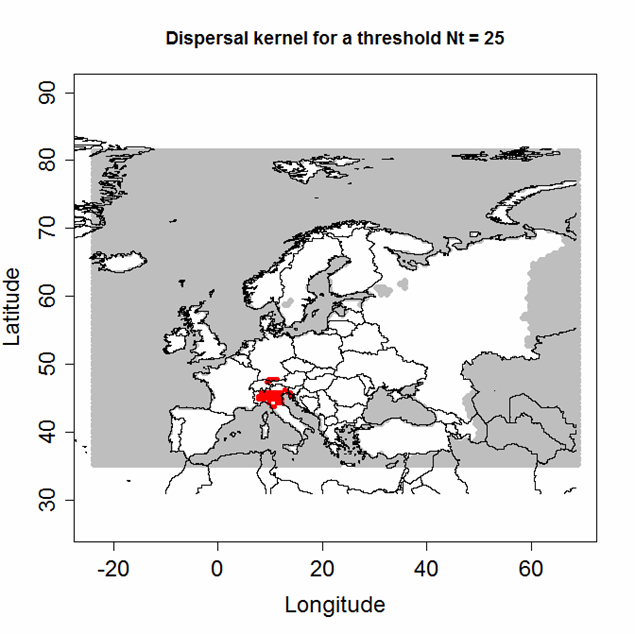


**Figure 3-8**: Left picture: Output of the dispersal kernel model for *A. glabripennis*; p_0_ = 1.3*10^-5^; **;** *λ_max_*= 5, **υ=30, u=1.5 km**, t=20 years (left picture) and 20 years (right picture) ; p_t_: population abundance (%); graduated colors from white (p_t_ < 10^(-6) %) to yellow, orange and red (p_t_ > = 10%). Grey means no data. Right picture: Output with a threshold = 25%. The population abundance (%) is above this threshold in red cells (red dots: cells where p_t_ > = threshold, white: cells where p_t_ < threshold. Grey means no data).

Results for υ=50

Figures 3-9 shows the results of the simulated spread of *A. glabripennis* over 20 years, starting in Northern Italy (for coordinates see the presence file) for υ=50 and a scale parameter u of 1.5 km. The si­mulated spread of *A. glabripennis* after 20 years only covers very small parts of Northern Italy for υ=50.


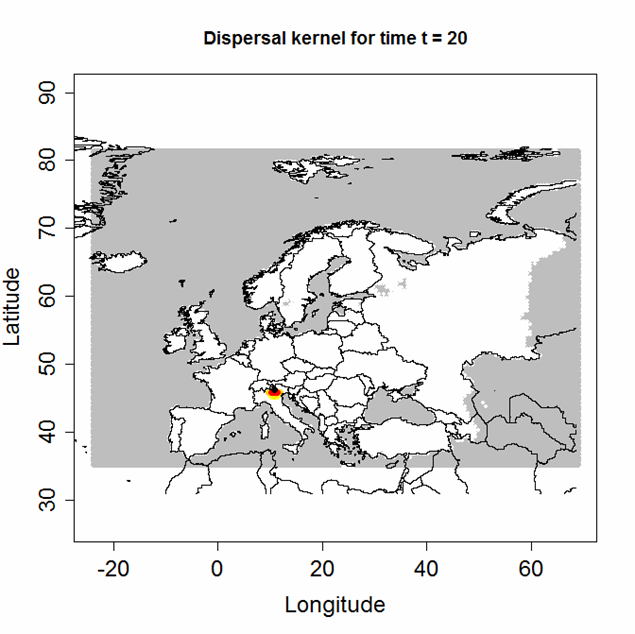

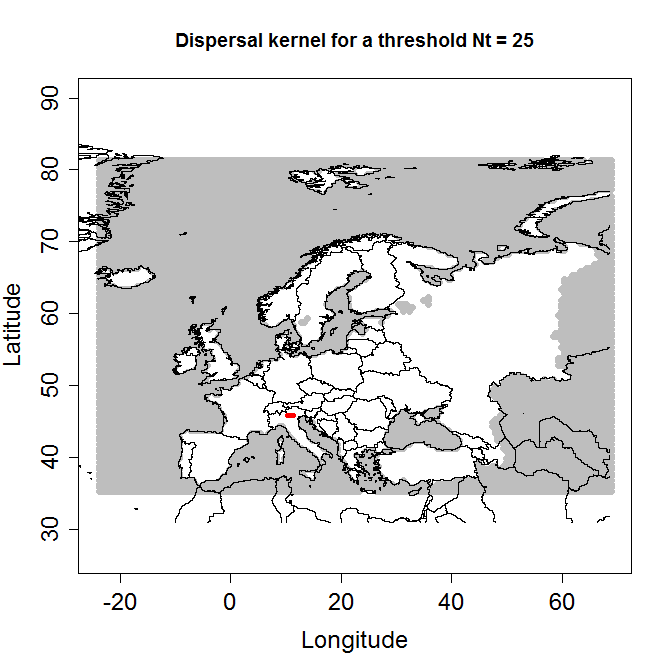


**Figure 3-9**: Left picture: Output of the dispersal kernel model for *A. glabripennis*; p_0_ = 1.3*10^-5^; **;** *λ_max_*= 5, **υ=50**, **u=1.5 km**, t=20 years (left picture) and 20 years (right picture) ; p_t_: population abundance (%); graduated colors from white (p_t_ < 10^(-6) %) to yellow, orange and red (p_t_ > = 10%). Grey means no data. Right picture: Output with a threshold = 25%. The population abundance (%) is above this threshold in red cells (red dots: cells where p_t_ > = threshold, white: cells where p_t_ < threshold. Grey means no data).

Testing a scenario with u = 3 km

Figures 3-10 shows the results of the simulated spread of *A. glabripennis* over 20 years, starting in Northern Italy with a higher scale para­meter u (u=3 km) for distance in the dispersal kernel.


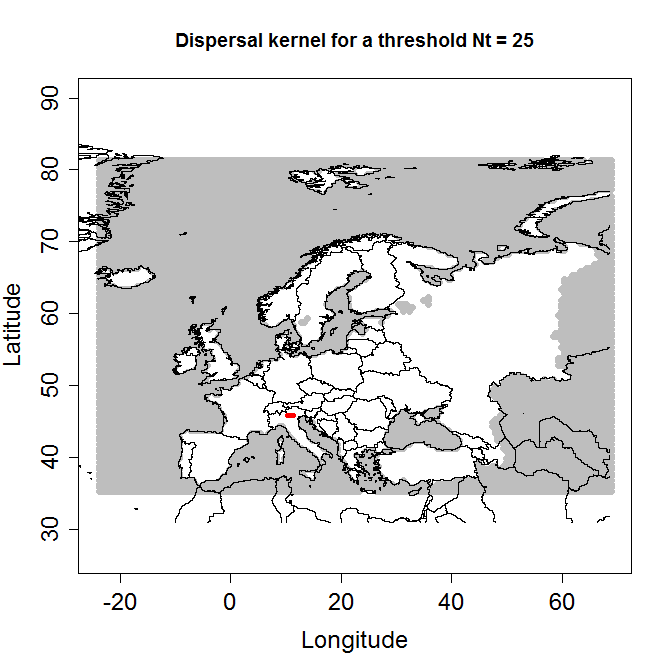

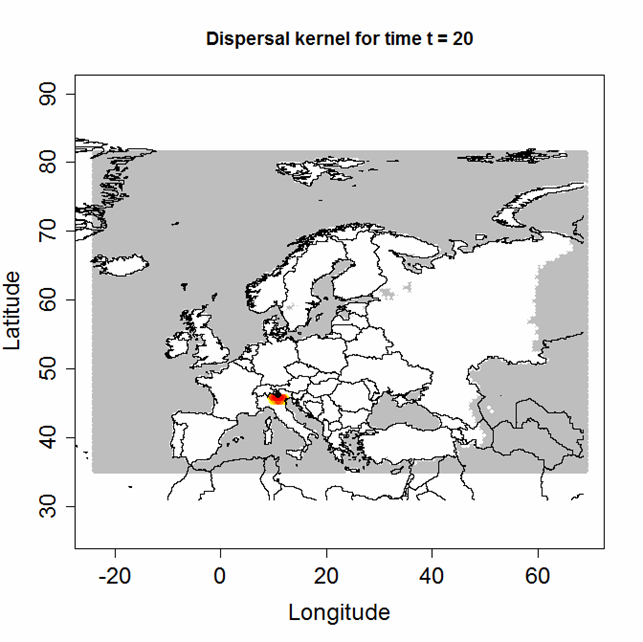


**Figure 3-10**: Left picture: Output of the dispersal kernel model for *A. glabripennis*; p_0_ = 1.3*10^-5^; **;** *λ_max_*= 5, **υ=50,** **u=3 km**, t=20 years (left picture) and 20 years (right picture) ; p_t_: population abundance (%); graduated colors from white (p_t_ < 10^(-6) %) to yellow, orange and red (p_t_ > = 10%). Grey means no data. Right picture: Output with a threshold = 25%. The population abundance (%) is above this threshold in red cells (red dots: cells where p_t_ > = threshold, white: cells where p_t_ < threshold. Grey means no data).

The beetle would have been expected to spread further with the assumption of u=3 km compared to u=1.5 km. But, the abundance of the beetle above a threshold of 25% of the carrying capacity for u=3 km after 20 years does not show a difference between the two different values for u. This means that although for u=3 km the beetle disperses further, at the places that are located farther from the starting point, the abundance of the beetle (pt) is below 25%. It would be reasonable to compare lower values (< 25%) for the carrying capacity threshold to see if more areas could be affected assuming u=3 km.

Modeling the spread of *A. glabripennis* for different starting points (Netherlands, Germany, France and Italy) over a period of 30 years

Figure 3-11 shows the results of the spread of *A. glabripennis* over a period of 30 years for different starting points in Europe for υ=50. *A. glabripennis* spreads within France, parts of Germany, Northern Italy, the Netherlands, Belgium, Austria, Czech Republic and parts of Slovenia.


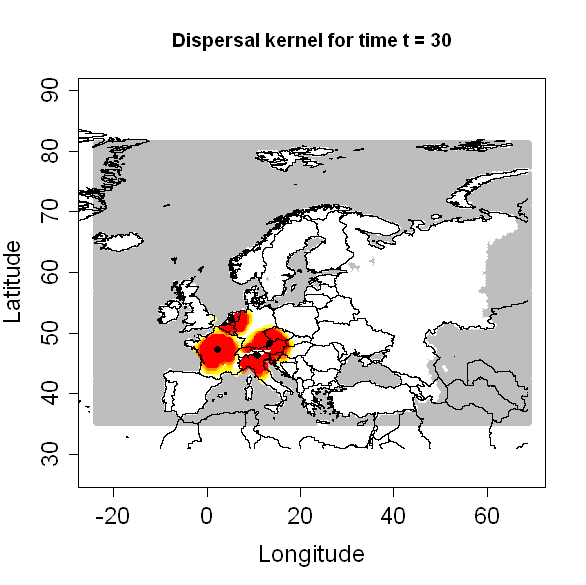

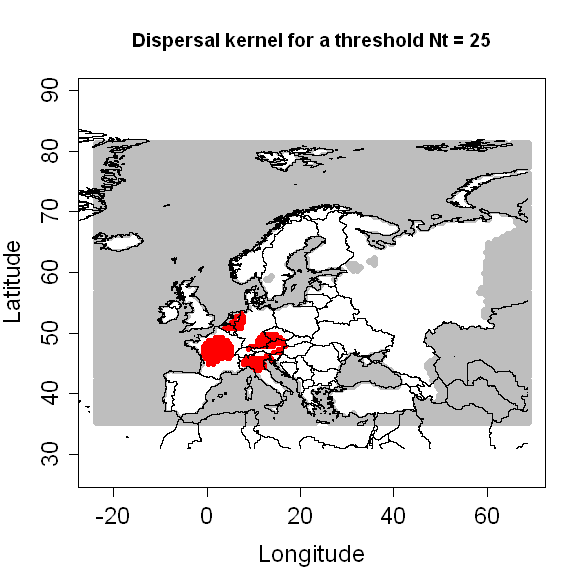


**Figure 3-11**: Left picture: Output of the dispersal kernel model for *A. glabripennis*; p_0_ = 1.3*10^-5^; **;** *λ_max_*= 5, **υ=50,** **u=1.5 km**, **t=30** years (left picture) and 20 years (right picture) ; p_t_: population abundance (%); graduated colors from white (p_t_ < 10^(-6) %) to yellow, orange and red (p_t_ > = 10%). Grey means no data. Right picture: Output with a threshold = 25%. The population abundance (%) is above this threshold in red cells (red dots: cells where p_t_ > = threshold, white: cells where p_t_ < threshold. Grey means no data).

**Summary**

The application of the spread models to the Asian longhorn beetle showed that the spread of the beetle could be mapped with reasonable results by the Radial Range Expan­sion Model and the Dispersal Kernel Model. According to the data and literature from Italy, USA and China the beetle disperses slowly by natural means and this was clearly displayed within the resulting maps, showing the areas at risk.

Additional information is provided by the Population dynamic model: due to the assumptions underlying this approach that every suitable grid cell is invaded at the same time the result shows the temporal spread within cells. It therefore highlights which areas are expected to have high beetle abundances after different time intervals and this can be used to identify the locations where the greatest consequences are to be expected.

The Radial Range Expansion Model only requires a few parameters (starting population, radial range expansion per year and starting point(s) for the infestation). For *A. glabripennis*, these were derived from outbreak data from Italy and from the literature.

The Dispersal Kernel Model also leads to reasonable results, which are best visible by mapping the area above a defined threshold (p_t_) for the abundance (p) after a specific time (t). However, in this case, the results depend on the choice of the shape parameter (υ) of the 2Dt kernel. The application therefore requires some testing of the model. For *A. glabripennis* the mapping of spread with a shape parameter υ=50 was assumed to lead to the best results in comparison to literature data but expert judgment and additional testing for p-values above υ=50 would be appropriate.

In additon models should be tested without any host plant restriction since applied broadleaf forest distribution does not really take account of the situation that the beetle mainly occurs in urban areas on ornamental trees planted in or near to towns and villages and not in natural forestry areas (this has been observed in Italy and as well in Asia). Therefore the absence of broadleaf forests should not be considered as a limiting factor for the ALB spreading in the models.

Summary of the commands for modelling the spread of *Anoplophora glabripennis*

library(sp)

library(raster)

library(rgdal)

elevmax = 2000

hostfile = T

*# load the R code*

plothost()

plotRA()

plotGI()

res = slg(N0=1.3*10^(-5),lmax=5,movie=F, t=20)

res = radial(RR=1.5,t=60,coord=c(11.5,45.6),figkm=T, figdd=F)

res = dispk(N0=NULL, t=1,lmax=5, p=50,u=1.5, presencefile=T, nen­try=NULL, figkm=F,figdd=T)

plotkernel (res$dispk,threshold=25, figkm=F, figdd=T)

**References**

Bancroft, J.S. & M.T. Smith (2005) Dispersal and influences on movement for *Anoplophora glabripennis* calculated from individual mark-recapture. *Entomologia Experimentalis et Applicata,* 116, 83–92.

EPPO (2001) First report of Anoplophora glabripennis in Austria. EPPO Reporting Service 2001/08/135. Available online at: <http://archives.eppo.org/EPPOReporting/2001/Rse-0108.pdf>

EPPO (2002) 13th USDA Interagency Research Forum on gypsy moth and other invasive species: Anoplophora glabripennis (Coleoptera: Cerambycidae – EPPO A1 quarantine pest): Situation in Austria. EPPO Reporting Service, 2002/02/019. Available online at: <http://archives.eppo.org/EPPOReporting/2002/Rse-0202.pdf>

EPPO (2004a) New finding of *Anoplophora glabripennis* in France. *EPPO Reporting Service*, 2004/11/163. Available online at: <http://archives.eppo.org/EPPOReporting/2004/Rse-0411.pdf>

EPPO (2004b) First report of *Anoplophora glabripennis* in Germany. *EPPO Reporting Service*, 2004/05/072. Available online at: http://archives.eppo.org/EPPOReporting/2004/Rse-0405.pdf

EPPO (2008) Situation of *Anoplophora glabripennis* in Germany. *EPPO Reporting Service*, 2008/05/095. Available online at: <http://archives.eppo.org/EPPOReporting/2008/Rse-0805.pdf>

EPPO (2009a) Situation of *Anoplophora glabripennis* in Austria in 2008. *EPPO Reporting Service*, 2009/03/044. Available online at: <http://archives.eppo.org/EPPOReporting/2009/Rse-0903.pdf>

EPPO (2009b) Situation of *Anoplophora glabripennis* in France in 2008. *EPPO Reporting Service*, 2009/03/045. Available online at: <http://archives.eppo.org/EPPOReporting/2009/Rse-0903.pdf>

EPPO (2009c) Eradication measures against *Anoplophora glabripennis* in Italy. *EPPO Reporting Ser­vice*, 2009/03/046. Available online at: <http://archives.eppo.org/EPPOReporting/2009/Rse-0903.pdf>

EPPO (2009d) *Anoplophora glabripennis* detected in the Veneto region, Italy. *EPPO Reporting Ser­vice*, 2009/08/157. Available online at: <http://archives.eppo.org/EPPOReporting/2009/Rse-0908.pdf>

EPPO (2010a) *Anoplophora glabripennis* found again in Germany. *EPPO Reporting Service*, 2010/01/006. Available online at: <http://archives.eppo.org/EPPOReporting/2010/Rse-1001.pdf>

EPPO (2010b) First record of *Anoplophora glabripennis* in the Netherlands. *EPPO Reporting Ser­vice,*2010/11/200. Available online at: <http://archives.eppo.org/EPPOReporting/2010/Rse-1011.pdf>

Faccoli M., Vettorazzo M., Zampini M., Zanini G., Coppe M. & Battisti A. (2011) An outbreak of *Anoplophora glabripennis* (Coleoptera: Cerambycidae) in NE Italy: first results of pest management and eradication attempt. Proceedings of the IUFRO Working Party 7.03.05 “Ecology and Management of Bark and Wood Boring Insects” meeting: “Novel Risks with Bark and Wood Boring Insects in Broadleaved and Conifer Forests”, 7 - 9 September 2011, Sopron (Hungary): 15.

Haack RA. (2006). Exotic bark and wood-boring Coleoptera in the United States: recent establish­ments and interceptions. *Canadian Journal of Forest Research* 36: 269 – 288

Hérard F, Maspero M, Ramualde N, Jucker C & Colombo M. (2009). *Anoplophora glabripennis* infes­tations (Col.: Cerambycidae) in Italy. *EPPO Bulletin* 39: 146 – 152

Herard, F., Cocquempot, C., Lopez, J., Covi, J., Maspero, M. & Colombo, M. (2005) Field study to evaluate the egg parasitoid *Aprostocetus anoplophorae* sp. n. (Hymenoptera: Eulophidae) on two *Anoplophora* hosts. *Proceedings, XV U.S. Department of Agriculture* *Interagency Re­search Forum on Gypsy Moth and Other Invasive* *Species 2004; 2004 January 13–16; Anna­polis, MD* (ed. by K. W. Gottschalk), pp. 40–42. Gen. Tech. Rep. NE-332. USDA-FS, Newtown Square, Pennsylvania.

Herard, F., M. Ciampitti, M. Maspero, H. Krehan, U. Benker, C. Boegel, R. Schrage, L. Bouhot-Delduc & P. Bialooki (2006) Anoplophora species in Europe: infestations and management processes. *Bulletin OEPP/EPPO Bulletin* 36: 470-474.

Huang J-F. et al. (1991). Current status and problems in the control of poplar wood-boring insect pests [Chinese]. *Journal Of Forest Disease And Insect Pests*, 1, 52-56.

Keena, M. A. and P. M. Moore. 2010. Effects of temperature on *Anoplophora glabripennis* (Coleoptera: Cerambycidae) larvae and pupae. Environ. Entomol. 34(4):1323-1335.

Lingafelter, S. W. & Hoebeke, E. R. (2002). Revision of the genus *Anoplophora* (Cerambycidae), *The Entomological Society of Washington, Washington, D.C*. 2002. 238 pp.

MacLeod A. 1997. Pest risk Analysis for *Anoplophora glabripennis* (Motschulsky). Proposal for addi­tion to the EPPO A1 list of quarantine pests. Available online at: <http://www.eppo.org/QUARANTINE/Pest_Risk_Analysis/PRA_documents.htm>

MacLeod, A, H. F. Evans & R. H. A. Baker (2002) An analysis of pest risk from an Asian longhorn beetle (*Anoplophora glabripennis*) to hardwood trees in the European community. *Crop Pro­tection* 21: 635-645.

Päivinen, R., M. Lehikoinen, A. Schuck, T. Häme, S. Väätäinen, P. Kennedy, P. & S. Folving (2001) Combining Earth Observation Data and Forest Statistics. EFI Research Report 14. European Forest Institute, Joint Research Centre - European Commission. EUR 19911 EN. 101p.

[*EFI Research Report 14*](http://www.efi.int/portal/virtual_library/publications/research_reports/14/). Available online at: <http://www.efi.int/portal/virtual_library/publications/research_reports/14/> , accessed on 21^st^ Janu­ary 2011.

Roden, D.B., R.A. Haack, M.A. Keena, D.W. McKenney, F.D. Beall & P.M. Roden, P.M. (2008) [Potential northern distribution of Asian longhorned beetle in North America](http://nrs.fs.fed.us/pubs/9350). In: McManus, Kathe­rine A; Gottschalk, Kurt W., eds. *Proceedings. 19th U.S. Department of Agriculture inte­ragency research forum on invasive species* *2008*; 2008 January 8-11; Annapolis, MD. Gen. Tech. Rep. NRS-P-36. Newtown Square, PA: U.S. Department of Agriculture, Forest Service, Northern Research Station: 65-67.

Sawyer, A.J. (2007) Spatial and temporal dynamics of Asian longhorned beetle infestations in Carteret and Linden, New Jersey. *Emerald Ash Borer and Asian Longhorned Beetle Research and Technology* *Development Meeting* (compiled by V. Mastro, D. Lance, R. Reardon and G. Parra), pp. 128–129. USDA, Forest Service Forest Health Technology Enterprise Team FHTET-2007-04, Cincinnati, Ohio.

Sawyer, A. J. (2008) Asian Longhorned Beetle: Annotated Host List. USDA-APHIS-PPQ, Otis pest Survey Detection and Exclusion Laboratory. Last revised 22/2/2008. Available online at:<http://www.aphis.usda.gov/plant_health/plant_pest_info/asian_lhb/downloads/hostlist.pdf>

Sawyer A.J. & W.S. Panagakos (2008) Spatial dynamics of the Asian longhorned beetle: Carteret, NJ, to Staten Island, NY, in nine years? In: McManus, Katherine A; Gottschalk, Kurt W., eds. *Pro­ceedings. 19th U.S. Department of Agriculture interagency research forum on invasive spe­cies 2008*; 2008 January 8-11; Annapolis, MD. Gen. Tech. Rep. NRS-P-36. Newtown Square, PA: U.S. Department of Agriculture, Forest Service, Northern Research Station: 68.

Schröder, T., U. Hoyer-Tomiczek, C. Bogel & R. Schrage (2006) Asian longhorn beetle in Germany. AFZ/Der Wald, *Allgemeine Forst Zeitschrift für Waldwirtschaft und Umweltvorsorge* 61 (16): 888-890.

Smith, M.T., Bancroft, J., Li, G., Gao, R. & Teale, S. (2001) Dispersal of Anoplophora glabripennis, *Environmental Entomology* 30 (6), 1036-1040.

Smith, M.T., Tobin, P.C., Bancroft, J., Li, G. & Gao, R. (2004) Dispersal and spatiotemporal dynamics of Asian longhorned beetle (Coleoptera: Cerambycidae) in China. *Environmental Entomology,* 33, 435–442.

Wen, J., Y. Li, N. Xia, & Y. Luo. 1998. Study on dispersal pattern of *Anoplophora glabripennis* adultsin poplars. *ACTA Ecol. Sin.* 18:269- 277.

**Williams D.W., Lee, H-P. & Kim, I-K. (2004).** Distribution and abundance of *Anoplophora glabripennis* (Coleoptera: Cerambycidae) in natural *Acer* stands in South Korea. *Environmental Entomol­***ogy, 33(3), 540-545.**

**Yang, Z.M., Wang, X.N., Yao, W.S., Chu, X.M. & Li, P. (2000) Generation differentiation and effective accumulated temperature of Anoplophora glabripennis (Motsch). Forest pest and Disease, 19, 12-14 [in Chinese].**

**Zhang, S.H., Xia, X. & Shu, H. (1995) Minimum temperature requirement for development and effective accumulated temperature of *Anoplophora glabripennis*. Journal of Inner Mongolia Institute of Agriculture and Animal Husbandry, 16, 45-49.**

**4. Root-Knot Nematode*, Meloidogyne* *enterolobii*** Yang et Eisenback, 1983

Authors: Hella Kehlenbeck (JKI, Germany) and Zhenya Ilieva (PPI, Bulgaria)

**Information on *M. enterolobii***

*M. enterolobii* is a tropical root-knot nematode that induces root galls and can cause significant damage to a large number of vegetable and ornamental crops in protected and field cultivation. Species was reported to overcome Mi gene of resistance in several main crops (Fargette, 1987; Cetintas *et al.,* 2008; Brito *et al.*, 2007a; Brito *et al.,* 2007b; Carneiro *et al.*, 2006; Berthou *et al.,* 2003). Further information can be derived from the EPPO A2 List of pests: (<http://www.eppo.org/QUARANTINE/listA2.htm> ).

Natural spread potential

The capacity of *M. enterolobii* for natural movement is very low and is comparable to other *Meloidogyne* species. According to Tiilikkala *et al*.(1995), free-living second-stage juveniles can move 1-2 m at maximum per year.

Human-assisted spread potential

*M. enterolobii* can easily be spread throughout the EU with infested rooted plants or soil. It can also be spread by farm machinery moving between different fields. Human assisted spread within the EU is very likely to occur through the trade of infested rooted host plants and soil.

The nematode has been found several times in imported plant material (ornamentals such as *Rosa sp., Ficus sp., Cactus sp*. from Africa and Asia). The most relevant pathways are traded host plants or cuttings with roots (with or without soil). Recent findings of *M. enterolobii* in imported ornamentals in the Netherlands show that the concentration of the pest on the pathway at origin can be high: imported *Rosa sp*. (from South Africa and China) and *Brachychiton bidwilli* Hook, 1859 (from Israel) were heavily infested. In a root sample of *Brachychiton bidwilli*, 12,360 eggs, 4,380 juveniles and 200 females were found (source: NPPO of the Netherlands).

Host range and host distribution

The host range of *M. enterolobii* includes a large number of horticultural and agricultural crops (Britto et al., 2004a,b,c; 2007; Cetintas et al., 2007). Amongst these are tomatoes, cucumbers, bell peppers, eggplants, beans, onion, potatoes, roses and a number of other ornamentals.

Findings of *M. enterolobii* in Europe

*(to be used as starting points for the modelling)*

- France

*M. enterolobii* has been reported from a cold greenhouse in France (Concarneau, Britanny) (Blok *et al*., 2002).

- Switzerland

*M. enterolobii* has been reported from two greenhouses in Switzerland, where it is still present (Kiewnick, 2008).

- Netherlands

In the Netherlands, *M. enterolobii* has been intercepted approximately 10 times (from 1991 to 2007) in imported plant material from Asia, South America and Africa.

Climatic suitability in Europe

Based on the current distribution, *M. enterolobii* needs a relatively high temperature to develop, i.e. within the tropical-Mediterranean temperature range. These conditions are present outdoors in the southern part of Europe and in greenhouses in the northern part. Although the precise temperature requirements of *M. enterolobii* have not yet been studied, it is likely that the northern limit to its range in Europe is comparable to *M. incognita* (Kofoid et White, 1949) Chitwood, 1949. The northern limit to the current area of distribution for *M. incognita* outdoors is likely to be just south of Paris (Karssen, 2002; Ritter, 1972). In Northern European countries, *M. enterolobii* will probably not be able to establish or cause major problems in field grown crops. It will probably only be able to establish in greenhouses.

The species has often been found in mixed populations with *Meloidogyne incognita*, *M. arenaria* (Neal, 1889) Chitwood, 1949 and *M., javanica* (Treub, 1885) Chitwood, 1949 (Diop, 1994, Carneiaro et al., 2001, Brito et al., 2004(a); Brito et al., 2004(c); Brito et al., 2007; Brito et Inserra, 2008; Centinas et al., 2008; etc.). All of these root-knot nematodes are widespread in the EU (CABI, 2007). We therefore decided to use the available data on the temperature limits of the following parthenogenetic nematode species: 1) *M. javanica* - a nematode with a more opportunistic life strategy and 2) *M. incognita* - a nematode with more of a K strategy (Evans & Perry, 2009) for *M. enterolobii*.

CLIMEX file

According to the assumptions made above, two different CLIMEX models were constructed by Zhenya Ilieva: one for *M. javanica* and another for *M. incognita.* Figures 4-1 and 4-2 show the area of potential establishment (EI, suitable area) and the growth potential (GI) for the two *Meloidogyne* species according to these models.


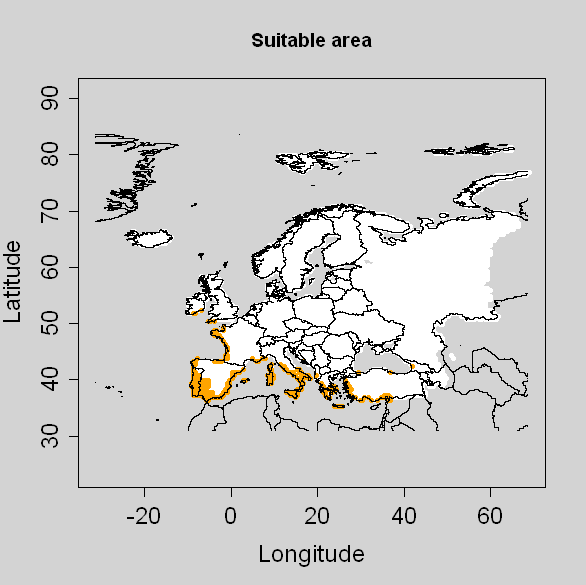

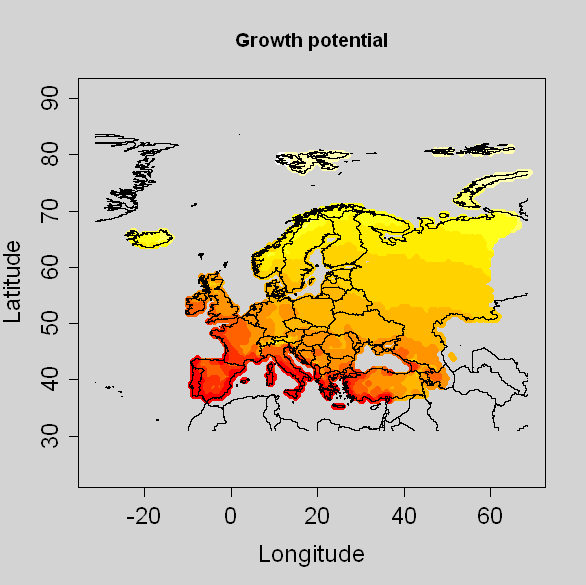

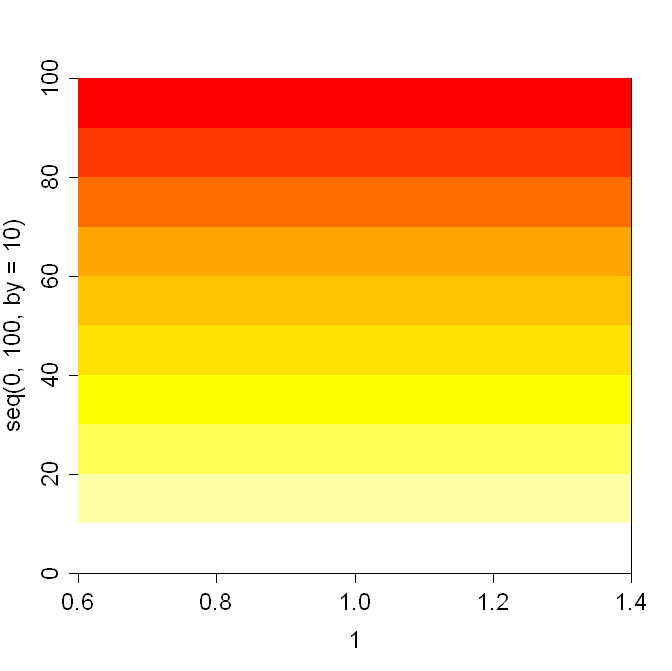


**Figure 4-1:** Suitable area (area of potential establishment, on the left; orange dots: EI>0, white dots: EI=0, grey: no data) and growth potential (GI; on the right) for *M. javanica* in Europe.


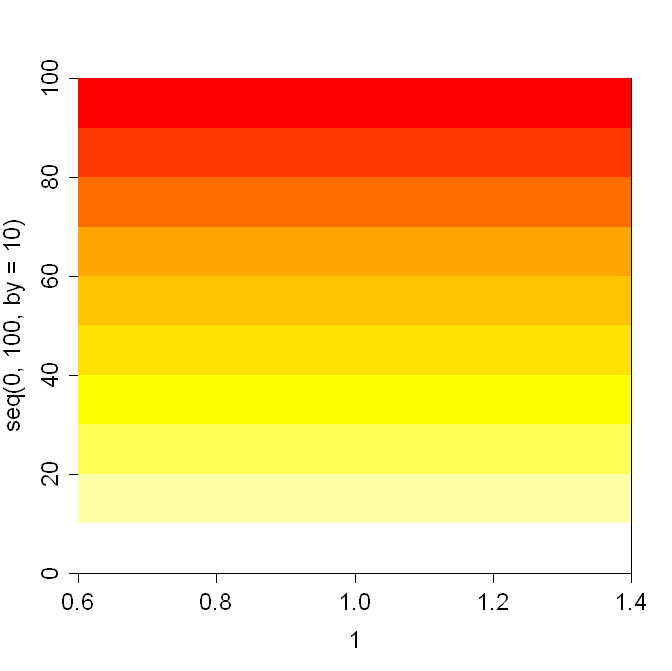

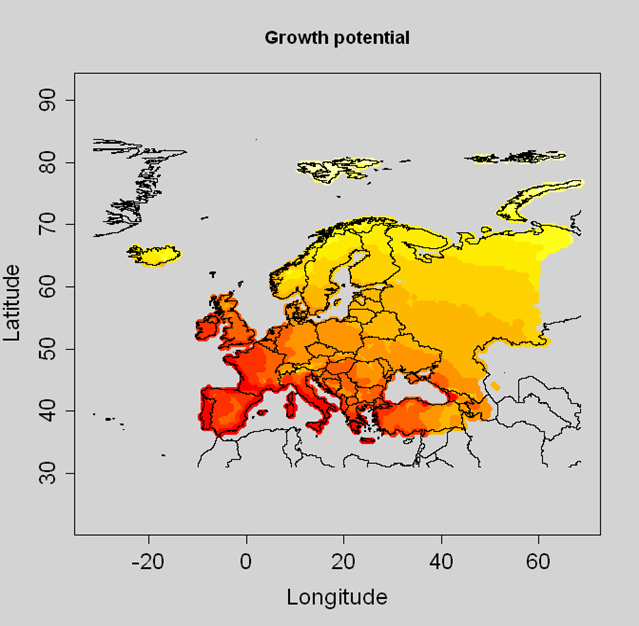

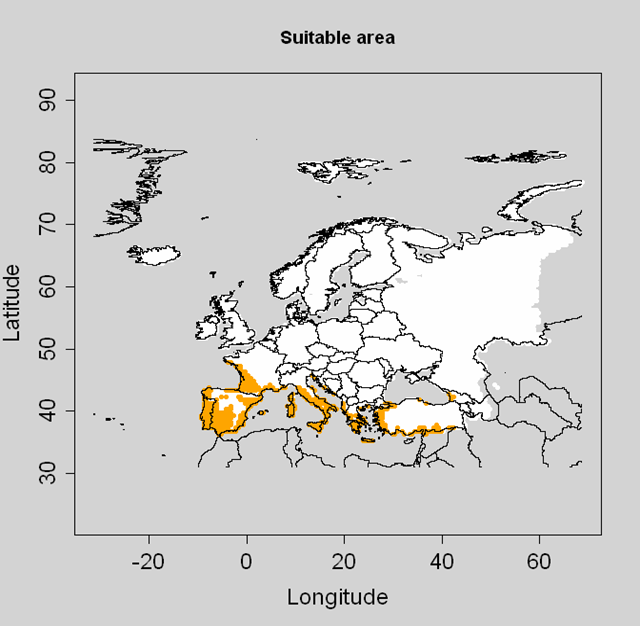


**Figure 4-2**: Suitable area (area of potential establishment, on the left; orange dots: EI>0, white dots: EI=0, grey: no data) and growth potential (GI; on the right) for *M. incognita* in Europe.

Based on the two CLIMEX models the number of suitable cells is given below:

*Meloidogyne javanica:*

[1] "The number of suitable cells is: 246"

[2] "The total number of cells in SLG, LG-Econ, radial, rand-radial is: 6416"

[3] "The total number of cells in the grid used by the dispersal kernel model is: 17298"

*Meloidogyne incognita:*

[1] "The number of suitable cells is: 402"

[2] "The total number of cells in SLG, LG-Econ, radial, rand-radial is: 6416"

[3] "The total number of cells in the grid used by the dispersal kernel model is: 17298"

Soil conditions in Europe

Soil is a very important factor that influences the potential distribution and spread of *Meloidogyne* species and needs to be taken into account when mapping the area of potential establishment. According to Braasch et al. (1996), *Meloidogyne* spp. can occur on a wide range of soil types, but their association with crop damage is mainly observed in sandy soils. Clay content above 30% and sand content below 30 % result in a denser soil structure that is generally considered unfavourable for root-knot nematodes (Greco and Di Vito, 2009). Both observations indicate that areas with such types of soils in the EU are at high risk from *M. enterolobii*, assuming the climate is suitable and hosts are available. These sandy soils are present throughout the EU (see figure 4-3 with Dominant Surface Textural map throughout Europe, Maxime Dupin). It is possible to create a 0/1 raster file to define the area where the soil is favourable (1) or not (0), and use it as the habitat file. However, here we use another approach. The models presented below only consider the CLIMEX dataset, and then, it is recommended to combine the soil maps to the outputs of the spread models in MCAS to define more precisely the potential distribution of *Meloidogyne enterolobii* (see procedures described by Dupin et al., 2011).


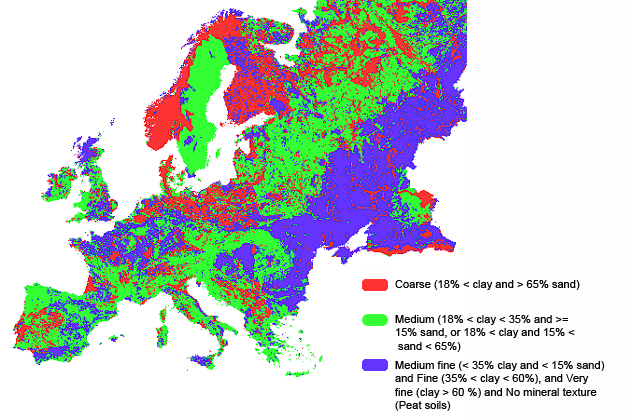


**Figure 4-3:** Dominant Surface Textural map throughout Europe (source: European Soil Database version 2 (JRC, 2010)

<http://eusoils.jrc.ec.europa.eu/ESDB_Archive/raster_archive/ESDBv2_ETRS_LAEA_raster_archive.html>)

Reproduction strategy

*M. enterolobii* reproduces by obligate mitotic parthenogenesis and is a polyploid organism (2n-44-46). Therefore, one second-stage juvenile can start a new population as it reproduces without sex (Yang & Eisenback, 1983). Within a greenhouse, it completes one generation every 4-6 weeks. Under field conditions in southern Europe, the maximum number of generations is estimated (at 20ºC with a 6 week generation time) at about 4-6 per year (Karssen & Moens, 2006). *Meloidogyne* spp. females are able to lay 100 – 500 eggs (Enneli & Toros, 1995, CABI, 2007).

**Model C: Population dynamics model**

*For the population dynamics model it is assumed that an initial population abundance p_0_ (%) is introduced in each suitable cell (cells where EI > 0) and then we simulate the “spread” or more precisely the growth within each cell according to a logistic function. On the output map, we will see areas which are the most suitable for the population growth if some individuals were introduced there.*

**Estimates of parameters**

- **The starting population p_0_**

*p_0_ is the population abundance for all suitable cells at time t = 0 expressed as a percentage of the maximum abundance (carrying capacity P_max_).*

*Calculation:*

$p_{0}=100*\frac{P_{0}}{P_{\max}}$*with n_0_ the number of introduced individuals, in each suitable cell, and P_max_ the carrying capacity (the maximum number of individuals in a cell). P_max_ = area_cell (km²) * proportion_covered_by_host * max_population_density (/km²),*

*or*

*P_max_ = area_cell (km²) * host_plant_density (/km²) * max_population_density (/host plant)*

Relevant information and data

There is a difference between the carrying capacity on different hosts but here we will take the maximum abundance on tomato plants (Britto et al. 2007b) 395 559 eggs and juveniles per plant (respectively per 1 m^2^).

We therefore assumed the maximum population density (*max_population_density/host plant) =* 396 000 eggs and juveniles per plant.

Calculation of the carrying capacity P_max_:

After Britto et al. 2007b

| P_max_ per m^2^  Pf per tomato plant at the end of the season | P_max_ per km^2^ | P_max_ per grid cell 1579 km^2^ |
| --- | --- | --- |
| 3.96x10^5^ | 3.96x10^11^ | 6.24x10^14^ |

P_max_ = 396,000 nematodes/plant; 1 tomato plant / m² = 396,000 nematodes/m²

P_max_= 396,000 nematodes/m² x 10^6^m² (transfer m² to km²) x 1579 km² (grid cell)

P_max_= 6.24 x 10^14^ nematodes/ grid cell

Calculation of p_0_:

The starting number of nematodes (=n_0_) for a new population can just be one second-stage juvenile. *M. enterolobii* females are able to lay 100 – 800 eggs (Karssen, pers. comm., 2009).

Here we take the number of eggs/juvenile found in samples (Starting number of nematodes n_0_ = 56,740 nematodes (see above, this is based on the number of eggs and juveniles found and approximately 200 eggs per female.

p_0_ = 100 x starting number P_0_ / P_max_ = %

p_0_ = 100 x 56740 / 6.24 x 10^14^ = 9.09 x 10^-9^

p_0_ = 9.1 x 10^-9^ %

- **The multiplication factor λ_max_**

*λ_max_ is the maximum year to year multiplication factor (“finite growth rate”) that a population could achieve under optimal conditions assuming unlimited space*

Relevant information and data:

Starr et Jeger (1985) investigated the overwintering of several populations of tropical root-knot nematodes and found that the average survival was 8.9% with a maximum of 33% in some of the populations. In our case if we used max Rf = 109.2 (Kleinwick et al., 2009) at the end of the season and 8.9% winter survival, then λmax = 9.7

- **Time frame:** 10 and 20 years

**Results:**

Results for the population dynamics model on *M. javanica* for 10 and 20 years


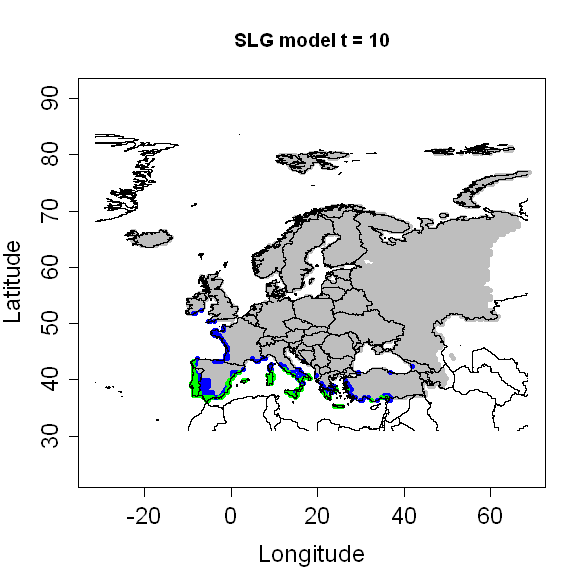

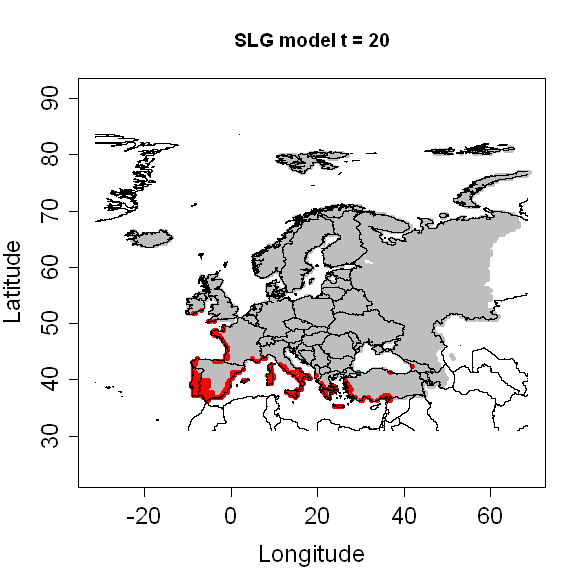


p_0_ = 9.1*10^-9^; λ_max_=9.7

p_0_ = 9.1*10^-9^; λ_max_=9.7

**Figure 4-4**: Output of the population dynamics model for *M. javanica for* 10 and 20 years; blue dots: 0 < pt < = 25, green dots: 25 < pt < = 50, orange dots: 50 < pt < = 75, red dots: 75 < pt < = 100, grey dots: pt=0, white: no data).
*Left Picture: 10 year ; R:> res = slg(N0=9.1*10^(-9),lmax=9.7, t=10)
Right Picture: 20 years; R:> res = slg(N0=9.1*10^(-9),lmax=9.7, t=20)*

Results for the population dynamics model on *M. incognita* for 10 and 20 years


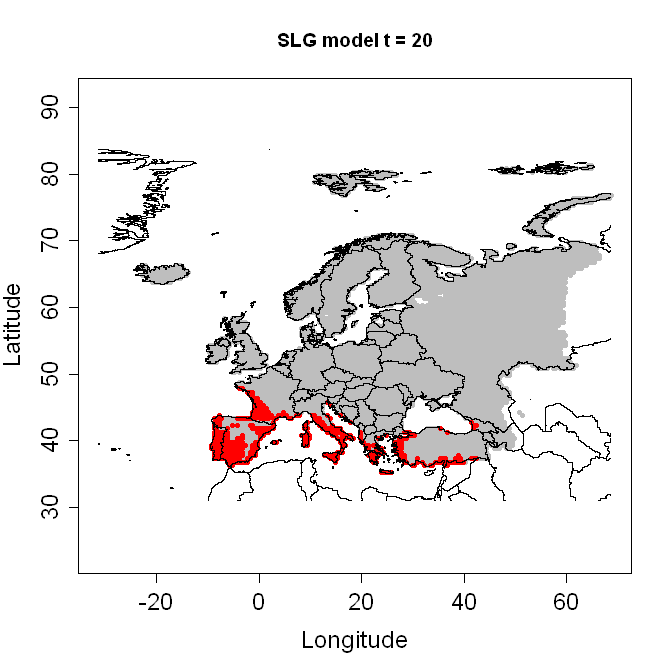

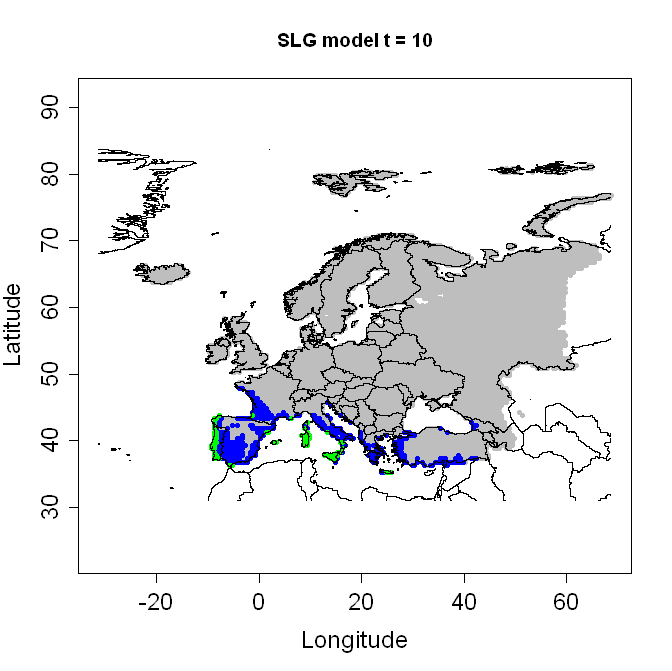


p_0_ = 9.1*10^-9^; λ_max_=9.7

p_0_ = 9.1*10^-9^; λ_max_=9.7

**Figure 4-5:** Output of population dynamics model for *M. incognita for* 10 and 20 years ( blue dots:
0 < pt < = 25, green dots: 25 < pt < = 50, orange dots: 50 < pt < = 75, red dots: 75 < pt < = 100, grey dots: pt=0, white: no data).
*Left Picture: 10 year ; R:> res = slg(N0=9.1*10^(-9),lmax=9.7, t=10)
Right Picture: 20 years; R:> res = slg(N0=9.1*10^(-9),lmax=9.7, t=20)*

The model gives an indication of the population densities that might develop assuming the pest has been introduced to all grid cells in the area of potential establishment with equal initial densities and without any control measures.

The model outputs are principally dependent on the CLIMEX Ecoclimatic Index and, because of the small amount of information about development rate on other suitable hosts for *M. enterolobii* and its presence in the soil and thus subject to a microclimate that is very different to that measured at weather stations, the accuracy is likely to be low. When selecting appropriate initial densities, it is important to take into account the likelihood that at very high initial densities the plant could die and in so doing have a major effect on population density.

The method is not very suitable for sedentary parasitic nematodes.

**Model B: Radial range expansion model**

*This model aims to determine the potential spread of a species introduced in the PRA area based on the radial rate expansion parameter. The model output is overlapped with the niche map (EI>0).*

**Estimates of parameters**

- **Radial rate of range expansion per year:**

c = 20 km/year

This value reflects both natural and human assisted spread.

- **Time frame**

t = 10 – 20 years

for the period of 10-20

- **Entry point(s)** or simulation of an introduction at a place
- France in Britanny (most western point) with coord = (-3.917, 47.867)
- Switzerland (see above, findings in Europe): tropical *Meloidogyne* spp. are unlikely to survive outdoors
- Southern Spain (-5.0, 37.0)

**Results of the radial range expansion based on the CLIMEX model for *M. javanica***


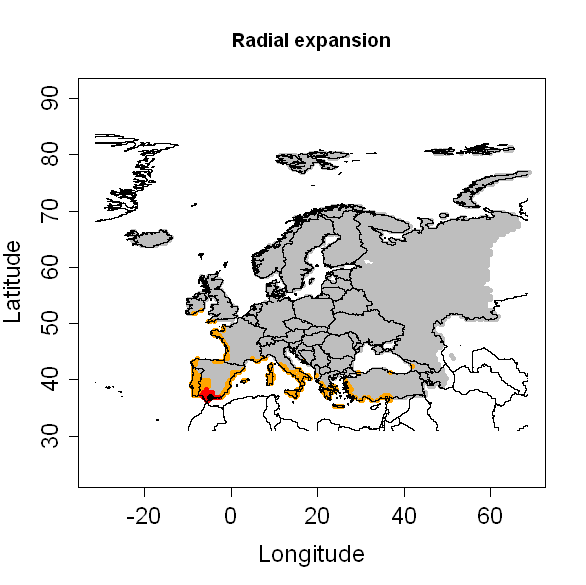

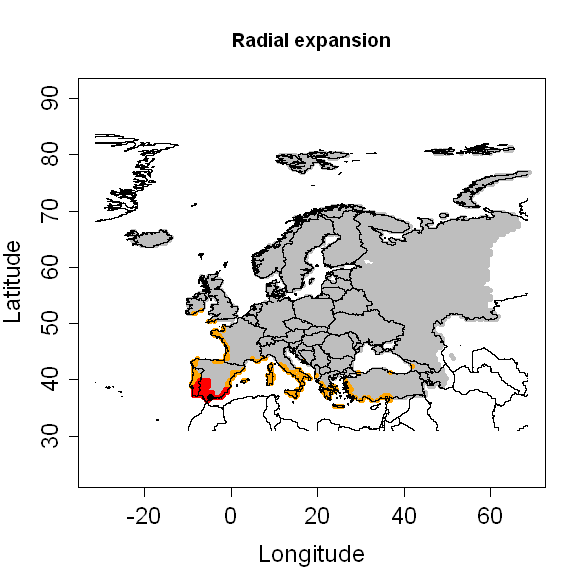


c = 20 km/year, t = 10 years

c = 20 km/year, t = 20 years

**Figure 4-6**: Radial range expansion of *M. enterolobii* for t=10 and 20 years, RR=20km/year and an entry point in Southern Spain based on the CLIMEX model for *M. javanica* (red dots: invaded cells, orange dots: non invaded but suitable cells, grey dots: EI=0, white: no data)

*Left picture: 10 years, R:> res = radial(c=10,t=20,coord=c(-5,37))
Right picture: 20 years, R:> res = radial(c=20,t=20,coord=c(-5,37))*

**Results of the radial range expansion based on the CLIMEX model for *M. incognita***


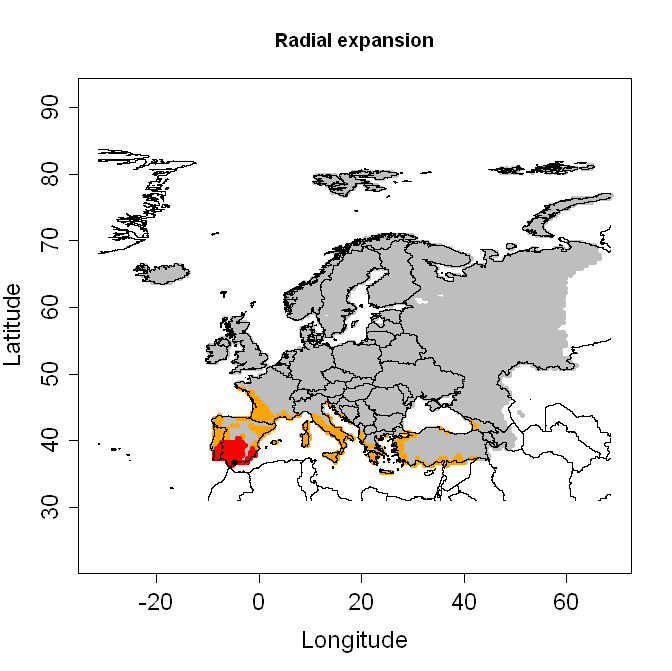

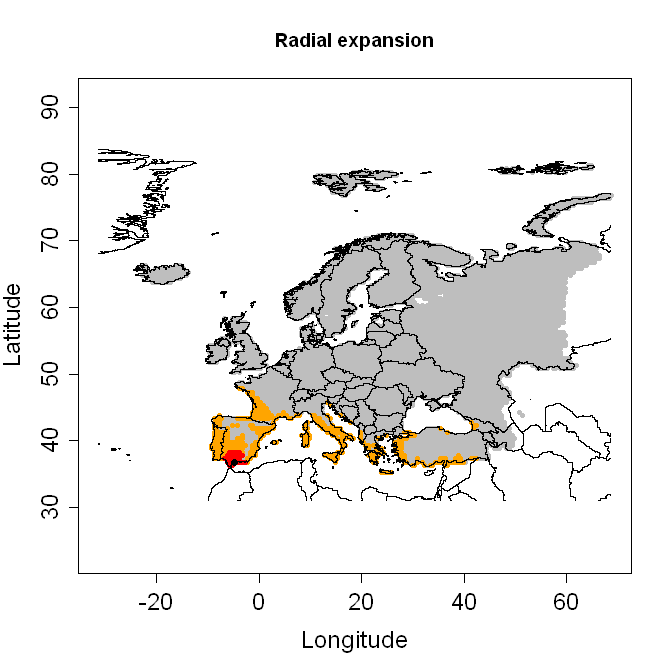


c = 20 km/year, t = 10 years

c = 20 km/year, t = 10 years

**Figure 4-7**: Radial range expansion of *M. enterolobii* for t=10 and 20 years, RR=20km/year and an entry point in Southern Spain based on the CLIMEX model for *M. incognita* (red dots: invaded cells, orange dots: non invaded but suitable cells, grey dots: EI=0, white: no data)

*Left picture: 10 years, R:> res = radial(c=20,t=10,coord=c(-5,37))
Right picture: 20 years, R:> res = radial(c=20,t=20,coord=c(-5,37))*

The scenario of Model B gives more realistic information about the potential spread of *Meloidogyne enterolobii*. Root-knot nematodes do not have a great dispersal potential and the range of 20 km per year is mostly due to human assisted spread (by shoes, machinery and devices). Intensity of occupation of potential territory in all variants is slower than in the SLG model type and more realistic if we consider only short distance spread of the species.

Again this model applies the worst scenario with continuous suitable hosts all over the climatically suitable area without any control measures.

**Model D: Deterministic version oft he dispersal kernel model**

*This type of model requires: a population growth model, a dispersal kernel (2Dt in this case) and the proportion of population engaged in dispersal.*

**Estimates of parameters**

- **The starting population p_0_**

*p_0_ is the population abundance for all suitable cells at time t = 0 expressed as a percentage of the maximum abundance (carrying capacity P_max_).*

p_0_ = 9.1 x 10^-9^ %

- The multiplication factor λ_max_

*λ_max_ is the maximum year to year multiplication factor (“finite growth rate”) that a population could achieve under optimal conditions assuming unlimited space*

λ_max_ = 9

- **Shape parameter of the 2Dt dispersal kernel υ**

*(number of degrees of freedom)*

*For* υ *= 1, this kernel has a Cauchy distribution (thick tail; a large number of individuals disperses further than 3σ) and for* υ$\to+\infty$*, it has a normal distribution (thin tail; individuals disperse at short distance).*

- short distance dispersal if only natural spread: υ=50

- long distance dispersal if we also take transport of plant material into account: υ=10

Apply first the default value υ=50, then also υ=10

- **Scale parameter for the distance of the 2Dt dispersal kernel (u)**

Here we use u = 20 km (see Radial Range Expansion Model , as well 10 km and 30 km)

- **presence file**

*The presence file contains the
coordinates for the entry point
and p_0_*


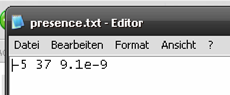


**Results of Model D based on the CLIMEX model for *M. javanica***

a) short distance spread (υ=50)


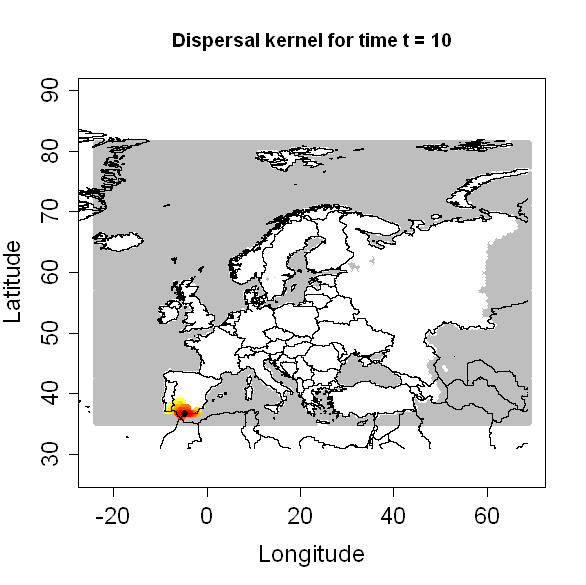

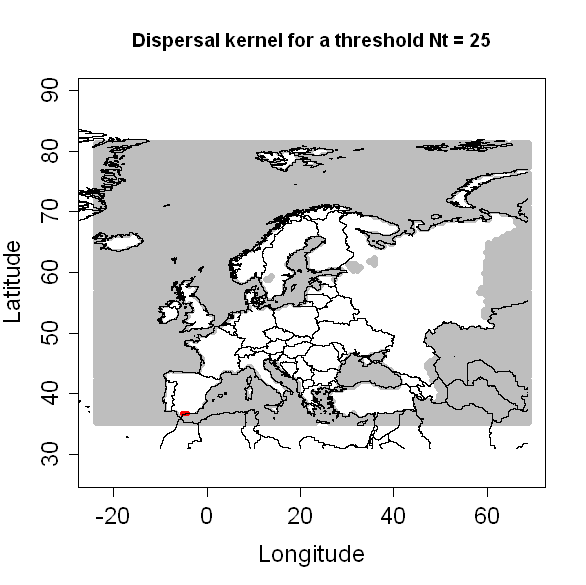


υ = 50; u = 20 km

υ = 50; u = 20 km

**Figure 4-8**: Output of the dispersal kernel model *M. enterolobii* based on the CLIMEX model for *M. javanica*; p_0_ = 9.1*10^-9^ and λmax = 9.7 for 10 years *(R:>* *res = dispk(t=10,lmax=9.7,* υ*=50,u=20)).* Left Picture: (pt: population abundance (%); graduated colors from white (pt < 10^(-6) %) to yellow, orange and red (pt > = 10%). Grey means no data). Right picture: with a threshold = 25 %. The population abundance (%) is above this threshold in red cells. (red dots: cells where pt > = threshold, white: cells where pt < threshold. Grey means no data)


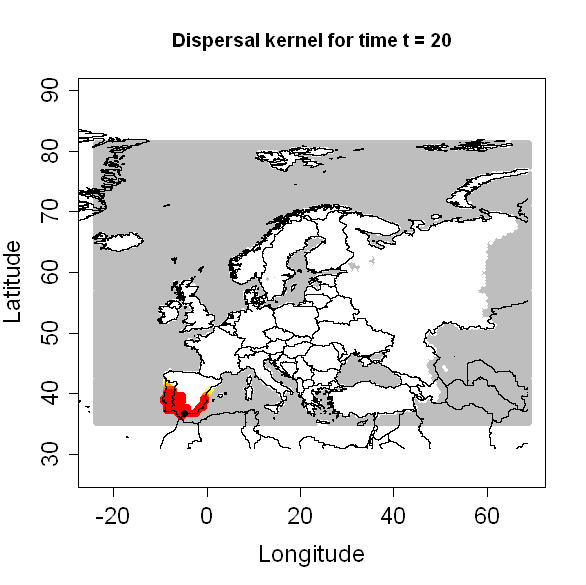

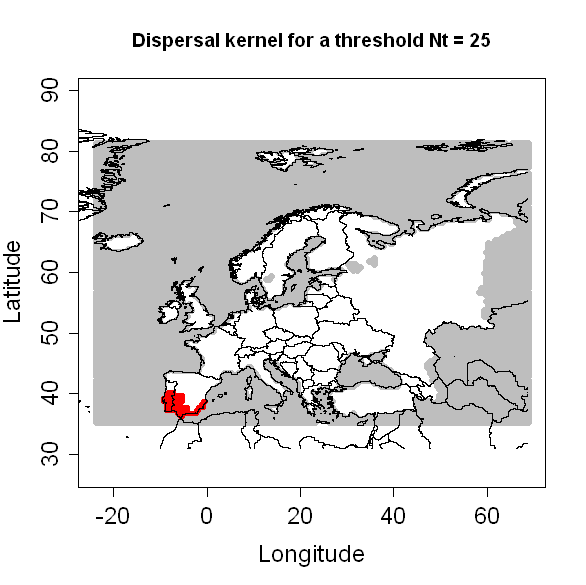


υ = 50; u = 20 km

υ = 50; u = 20 km

**Figure 4-9**: Output of the dispersal kernel model *M. enterolobii* based on the CLIMEX model for *M. javanica*; p_0_ = 9.1*10^-9^ and λmax = 9.7 for 20 years (*R:>* *res = dispk(t=10,lmax=9.7,*υ*=50,u=20)).* Left Picture: (pt: population abundance (%); graduated colors from white (pt < 10^(-6) %) to yellow, orange and red (pt > = 10%). Grey means no data); Right picture: with a threshold = 25 %. The population abundance (%) is above this threshold in red cells. (red dots: cells where pt > = threshold, white: cells where pt < threshold. Grey means no data)

b) long distance spread (υ=10)


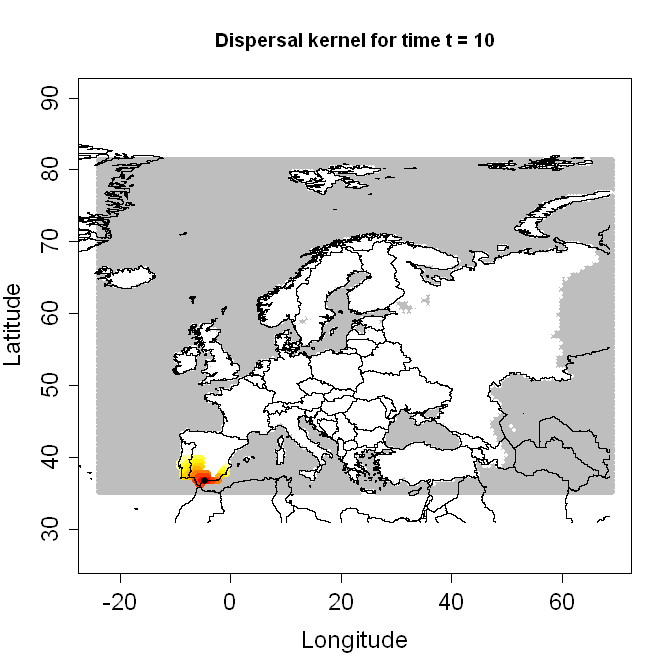

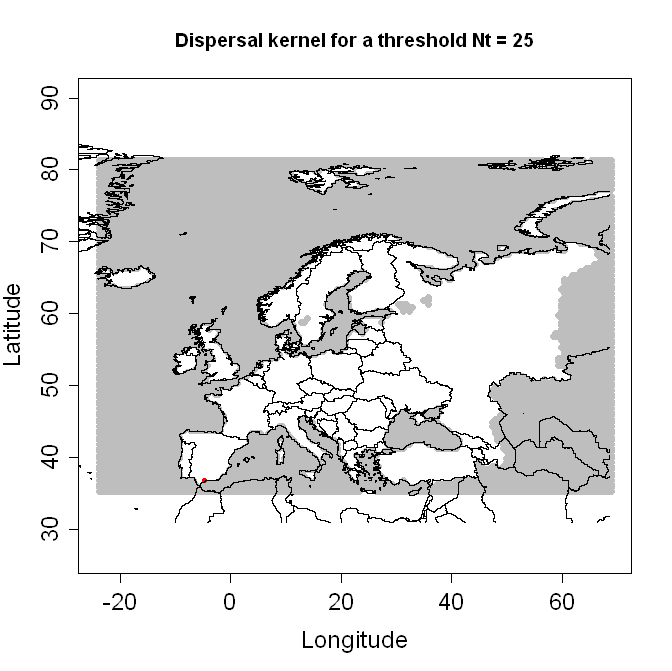


υ = 10; u = 20 km

υ = 10; u = 20 km

**Figure 4-10**: Output of the dispersal kernel model *M. enterolobii* based on the CLIMEX model for *M. javanica*; p_0_ = 9.1*10^-9^ , λmax = 9.7, u = 20 km and p=10 for 10 years; *R:> res = dispk(N0=NULL, t=10,lmax=9.7, υ=10,u=20, presencefile=T, nentry=NULL, figkm=F,figdd=T)*

Left Picture: (pt: population abundance (%); graduated colors from white (pt < 10^(-6) %) to yellow, orange and red (pt > = 10%). Grey means no data)
Right picture: with a threshold = 25 %. The population abundance (%) is above this threshold in red cells. (red dots: cells where pt > = threshold, white: cells where pt < threshold. Grey means no data)


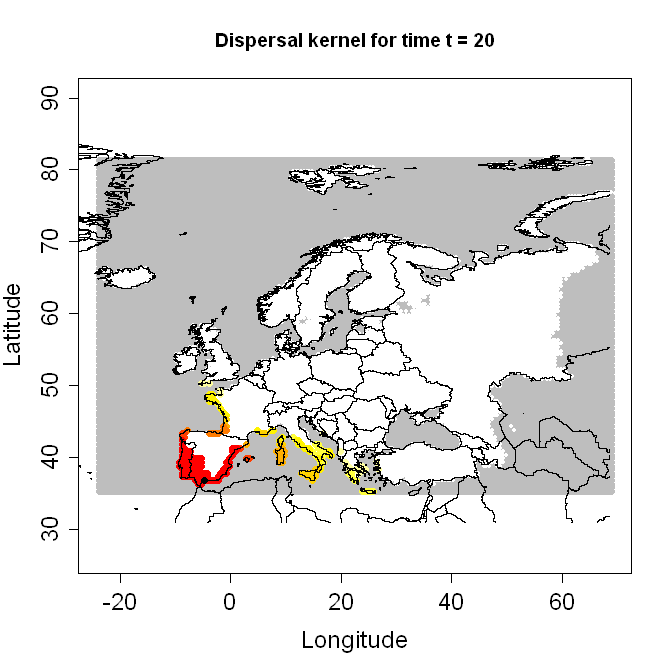

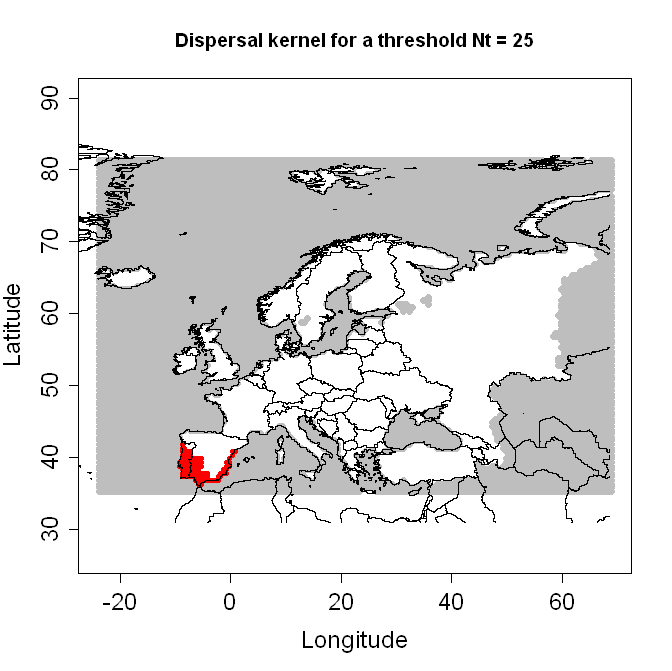


υ = 10; u = 20 km

υ = 10; u = 20 km

**Figure 4-11**: Output of the dispersal kernel model *M. enterolobii* based on the CLIMEX model for *M. javanica*;
p_0_ = 9.1*10^-9^ , λmax = 9.7, u = 20 km and p=10 for 20 years; *R:> res = dispk(p0=NULL, t=20,lmax=9.7, υ=10, u=20, presencefile=T, nentry=NULL, figkm=F,figdd=T)*

Left Picture: (pt: population abundance (%); graduated colors from white (pt < 10^(-6) %) to yellow, orange and red (pt > = 10%). Grey means no data)
Right picture: with a threshold = 25 %. The population abundance (%) is above this threshold in red cells. (red dots: cells where pt > = threshold, white: cells where pt < threshold. Grey means no data)

**Results of Model D based on the CLIMEX model for *M. incognita***

a) short distance spread (υ=50)


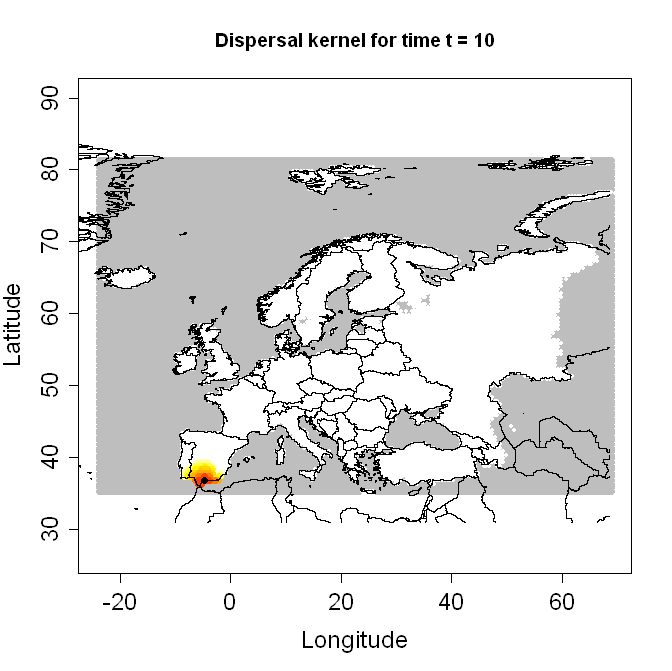

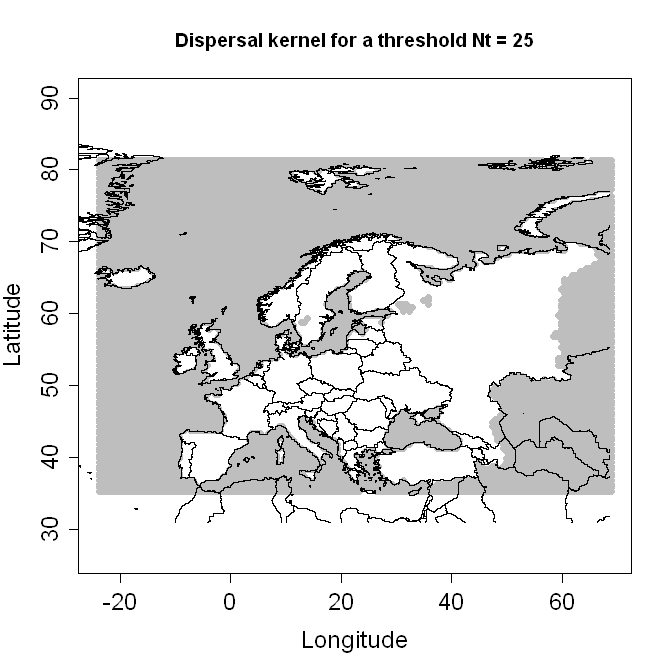


υ = 50; u = 20 km

υ = 50; u = 20 km

**Figure 4-12**: Output of the dispersal kernel model *M. enterolobii* based on the CLIMEX model for *M. incognita*; p_0_ = 9.1*10^-9^ and λmax = 9.7 for 10 years *(R:>* *res = dispk(t=10,lmax=9.7,* υ*=50,u=20)).*
Left Picture: (pt: population abundance (%); graduated colors from white (pt < 10^(-6) %) to yellow, orange and red (pt > = 10%). Grey means no data)
Right picture: with a threshold = 25 %. The population abundance (%) is above this threshold in red cells. (red dots: cells where pt > = threshold, white: cells where pt < threshold. Grey means no data)

υ = 50, u = 20 km

υ = 50, u = 20 km


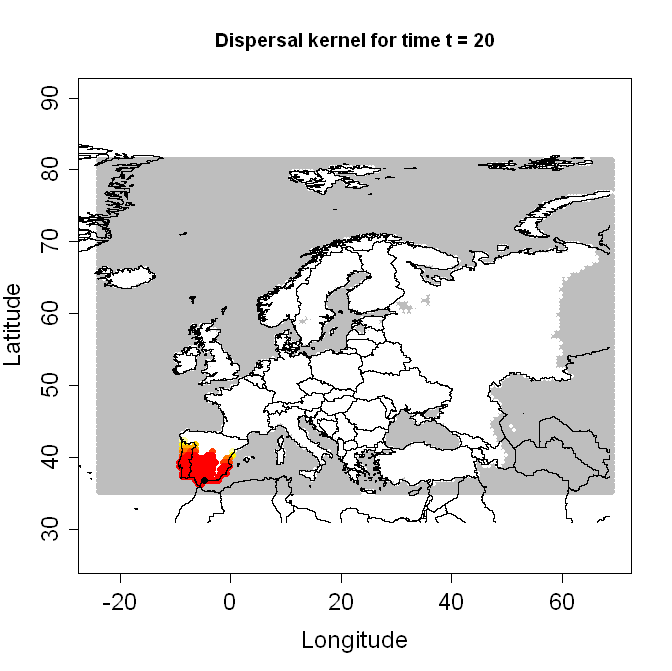

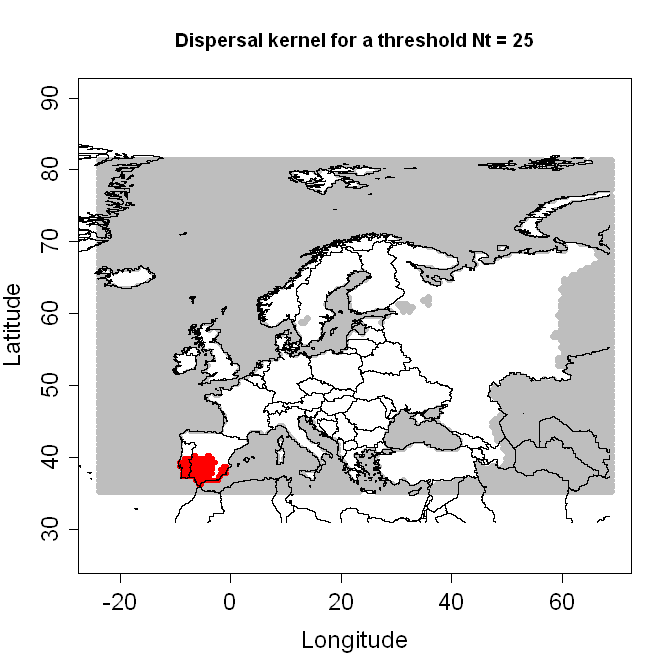


**27.86% of the risk area invaded**

**112 cells invaded**

**Figure 4-13**: Output of the dispersal kernel model *M. enterolobii* based on the CLIMEX model for *M. incognita*; p_0_ = 9.1*10^-9^ and λmax = 9.7 for 20 years *(R:>* *res = dispk(t=20,lmax=9.7,* υ*=50,u=20)).*Left Picture: (pt: population abundance (%); graduated colors from white (pt < 10^(-6) %) to yellow, orange and red (pt > = 10%). Grey means no data). Right picture: with a threshold = 25 %. The population abundance (%) is above this threshold in red cells. (red dots: cells where pt > = threshold, white: cells where pt < threshold. Grey means no data)

b) long distance spread (υ=10)


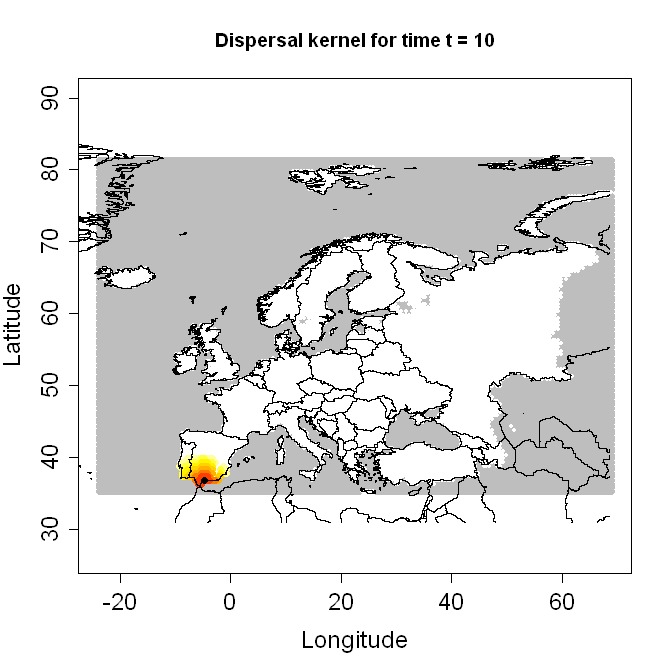

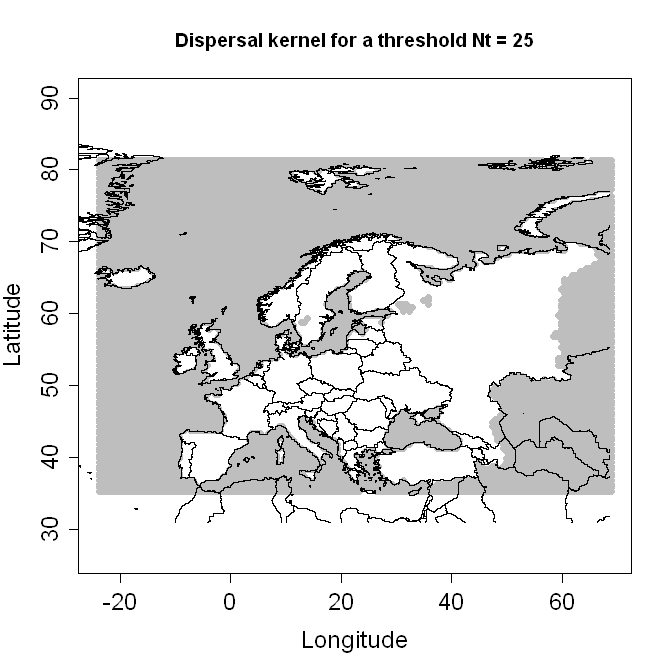


υ = 10, u = 20 km

υ = 10, u = 20 km

**Figure 4-14**: Output of the dispersal kernel model *M. enterolobii* based on the CLIMEX model for *M. incognita*; p_0_ = 9.1*10^-9^ , λmax = 9.7, u = 20 km and p=10 for 10 years; *R:> res = dispk(N0=NULL, t=10,lmax=9.7, υ=10,u=20, presencefile=T, nentry=NULL, figkm=F,figdd=T).* Left Picture: (pt: population abundance (%); graduated colors from white (pt < 10^(-6) %) to yellow, orange and red (pt > = 10%). Grey means no data). Right picture: with a threshold = 25 %. The population abundance (%) is above this threshold in red cells. (red dots: cells where pt > = threshold, white: cells where pt < threshold. Grey means no data)


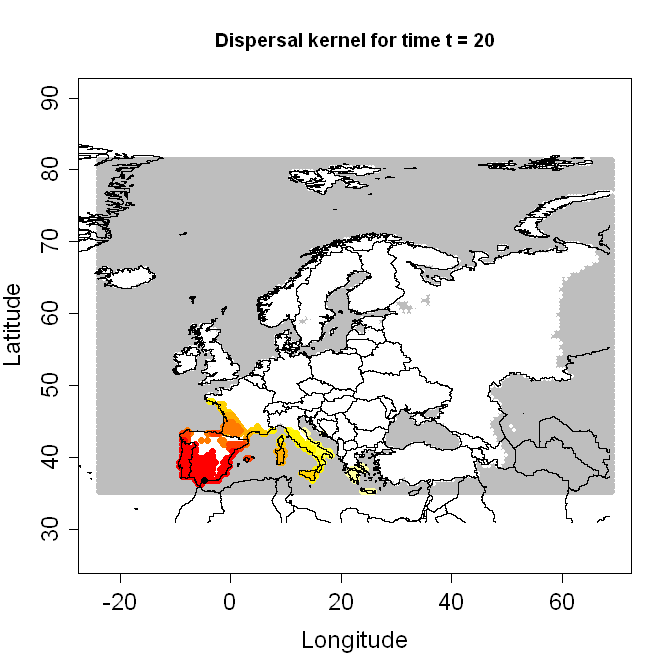

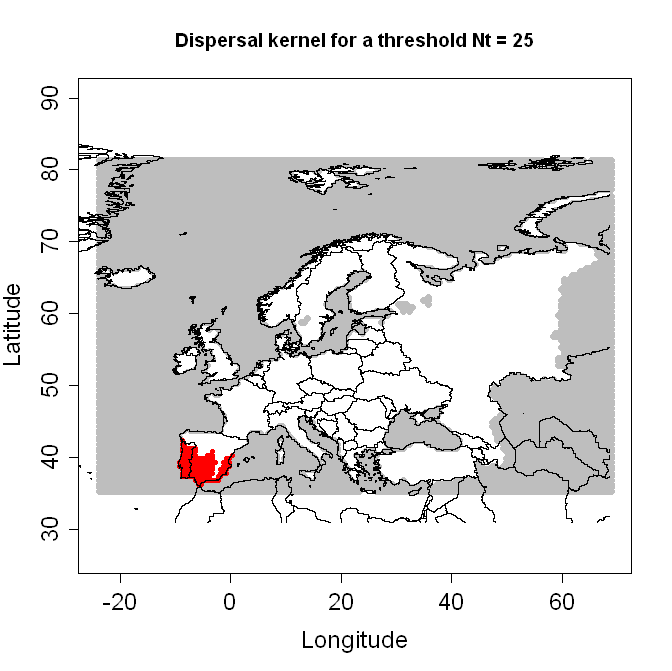


υ = 10, u = 20 km

υ= 10, u = 20 km

**38.3% of the risk area invaded**

**154 cells invaded**

**Figure 4-15**: Output of the dispersal kernel model *M. enterolobii* based on the CLIMEX model for *M. incognita*; p_0_ = 9.1*10^-9^ , λmax = 9.7, u = 20 km and p=10 for 12 years; *R:> res = dispk(p0=NULL, t=20,lmax=9.7, υ=10,u=20, presencefile=T, nentry=NULL, figkm=F,figdd=T).*
Left Picture: (pt: population abundance (%); graduated colors from white (pt < 10^(-6) %) to yellow, orange and red (pt > = 10%). Grey means no data). Right picture: with a threshold = 25 %. The population abundance (%) is above this threshold in red cells. (red dots: cells where pt > = threshold, white: cells where pt < threshold. Grey means no data)

The Dispersal Kernel Model gives the most realistic scenario of all the three ones tested. You can obtain a clearer impression of the intensity of invasion and which part of the endangered area could be invaded for a certain period from a certain entry point. It is obvious that in the case of low initial pest densities damage will appear later and will not be detected for a longer period of time. In such situations, restriction measures on the movement of host plants/host plant products could be effective.

The Dispersal Kernel Model shows a higher sensitivity to changes in the parameters compared to the other tested models concerning not only different p_0_ but also more precise levels of invasion in different parts of endangered area. The method clearly differentiates if only short distance dispersal exists (p=50) or long distance spread is also included (p=10).

**Summary**

Model C, the population dynamics model, reflects the Ecoclimatic Index obtained from CLIMEX and gives a rough indication of the population densities of the pest over time without the application of control measures.

Model B, the Radial Range Expansion Model, provides more realistic maps about the potential spread of *Meloidogyne enterolobii* and the longer distances travelled mostly occur by human assisted spread. The invasion of potential areas is slower than for the population dynamics model.

The results of Model D, the Dispersal Kernel Model, are the most realistic. The method can be applied to differentiate between short distance dispersal (e.g. p=50) and long distance spread (e.g. p=10)

Summary of the commands for modelling the spread of *M.enterolobii*

library(sp)

library(raster)

library(rgdal)

elevmax = F

hostfile = F

*# load the R code*

res = slg(N0=9.1*10^(-9),lmax=9.7,t=10)

res = radial(RR=20,t=10,coord=c(-5,37))

res = dispk(N0=NULL, t=10,lmax=9.7, p=50,u=20, presencefile=T, nentry=NULL, figkm=F,figdd=T)

**References**

Berthou,F, Kouassi A, Bossis M, Dantec JP, Eddaoudi M, Ferji Z, Pellé R, Taghzouti M, Ellisséche D & Mugniéry D (2003) Enhancing the resistance of the potato to Southern Root-knot Nematodes by using *Solanum sparsipilum* germplasm. *Euphytica* 132, 57 – 65.

Blok VC, Wishart J, Fargette M, Berthier K, Philips MS (2002) Mitochondrial DNA differences distinguishing *Meloidogyne mayaguensis* from the major species of tropical root-knot nematodes. *Nematology* 4, 773-781.

Braasch H, Wittchen U & Unger JG (1996) Establishment potential and damage probability of *Meloidogyne chitwoodi* in Germany. *Bulletin OEPP/EPPO Bulletin*, 26, 495 – 509.

Brito JA & Inserra NI (2008) Nematology Section. *Tri-ology* 47,18-19

Brito JA, Stanley J, Cetintas R, Powers T, Inserra R, McAvoy G, Crow B & Dickson D (2004 a) *Meloidogyne mayaguensis* a new plant nematode species, poses threat for vegetable production in Florida. 2004 Annual international research conference on methyl bromide alternatives and emissions reductions. *Conference* *proceedings*. *On-line* available at [www.mbao.org](http://www.mbao.org/).

Brito JA, Stanley J, Cetintas R, Powers T, Inserra R, McAvoy G, Crow B & Dickson D (2004 b) *Meloidogyne mayaguensis* a reproduction on resistant tomato and pepper. 2004 Annual international research conference on methyl bromide alternatives and emissions reductions. Conference proceedings. *On-line* available at [www.mbao.org](http://www.mbao.org/).

Brito JA, Stanley J, Cetintas R, Powers T, Inserra R, McAvoy G, Mendes ML, Crow B, Dickson D (2004 c) Identification and host preference of *Mleoidogyne mayaguensis* and other root-knot nematodes from Florida, and their susceptibility to *Pasteuria penetrans. Journal of Nematology* 36(3), 308-309.

Brito JA, Stanley JD, Mendes ML, Cetintas R & Dickson DW (2007) Host status of selected cultivated plants to *Meloidogyne mayaguensis* in Florida. *Nematropica*. 37, 65-71.

Brito JA, Stanley JD, Kaur R, Cetintas R, Vito M. di, Thies JA & Dickson DW (2007a) Effects of the *Mi-1*, *N* and *Tabasco* genes on infection and reproduction of *Meloidogyne mayaguensis* on tomato and pepper genotypes. *Journal of Nematology*. 39, 327-332.

Brito JA, Stanley JD, Mendes ML, Cetintas R & Dickson DW (2007b) Host status of selected cultivated plants to *Meloidogyne mayaguensis* in Florida. *Nematropica*. 37, 65-71.

CABI (2007). Crop protection compendium, CAB International, Wallingford, UK, 2007.

Carneiro RMDG, Almeida MRA & Braga RS (2006) First record of *Meloidogyne mayaguensis* parasitizing resistant root-knot nematode pepper and tomato plants in São Paulo State, Brazil. *Nematologia Brasileira*, 30,:81-86.

Carneiro RMDG, Moreira WA, Almeida MRA & Gomes ACMM (2001) First record of *Meloidogyne mayaguensis* on guave in Brazil. *Nematologia Brasileira,* 25, 223-228.

Cetintas R, Brito JA & Dickson DW (2008) Virulence of four Florida isolates of *Meloidogyne mayaguensis* to selected soybean genotypes. *Nematropica*. 38, 127-136.

Cetintas R, Brito JA & Dickson DW (2008) Virulence of four Florida isolates of *Meloidogyne mayaguensis* to selected soybean genotypes. *Nematropica*. 38, 127-136.

Cetintas R., Kaur R, Brito JA, Mendes ML, Nyczepir & AP, Dickson DW (2007) Pathogenicity and reproductive potential of *Meloidogyne mayaguensis* and *M. floridensis* compared with three common *Meloidogyne* spp. *Nematropica* 37, 21-31.

Diop MT (1994) Les nématodes parisites des cultures maraîcheres au Sénégal. Distribution de Pasteuria penetrans, actinomycète parasite des nématodes du genre *Meloidogyne*. *Mémoire de D.E.A. de Biologie Animale, Faculté des Sciences Techniques, Université Cheikh Anta Biop de Dakar.*

Dupin M, Brunel S, Baker R, Eyre D & Makowski D (2011) A comparison of methods for combining maps in pest risk assessment: application to Diabrotica virgifera virgifera. EPPO Bulletin/Bulletin OEPP, 41 (2), 217-225.

Enneli S & Toros S (1995) Investigation on Biology of Root-knot Nematode [*Meloidogyne incognita* (Kofoid at White) Chitwood] Harmful on Tomatoes. *Journal of Turkish Phytopatology,* 25 (3): 109-116.

Evans AAF and Perry RN (2009) Survival Mechanisms. In: “*Root-knot nematodes” edited by R.N. Perry, M. Moens, & James L. Starr, CABI 2009*, p.: 201-222.

Fargette M (1987) Use of the esterase phenotype in the taxonomy of the genus *Meloidogyne*. 2. Esterase phenotypes observed in Western African populations and their characterisation. *Revue de Nematologie* 10, 45-56.

Greco N and Di Vito M (2009). Population Dynamics and Damage Levels. In: “*Root-knot nematodes” edited by R.N. Perry, M. Moens, & James L. Starr, CABI 2009*, p.: 246-274.

Karssen G (2002) The plant-parasitic nematode genus *Meloidogyne* Göldi, 1892 (Tylenchida) in Europe. Brill, Leiden. P. 157

Karssen G and Moens M (2006) Root-knot nematodes. In: *Plant Nematology. Ed. R.N. Perry & M. Moens. CABI, Wallingford*.. 59-90.

Kiewnick S, Dessimoz M, Frank L, (2009) Effects of the Mi-1 and the N root-knot nematode-resistance gene on infection and reproduction of *Meloidogyne enterolobii* on tomato and pepper cultivars. *Journal of Nematology* 41(2):134–139. 2009.

Kiewnick S, Oggenfuss M, Frey B, Roth I, Eder R, Frey JE (2008) Nouvelle espèce de nematode dans les serres suisses. *Der Gemüsebau/Le Maraîcher* no. 2, 7-9.

Ritter M. (1972) R61e economique et importance des *Meloidogyne* en Europe et dans le bassin Mediterraneen. *EPPO Bull*. no 6 : 17-22Robinet et al. 2010

Starr JL et Jeger MJ (1985) Dynamics of Winter Survival of Eggs and Juveniles of Meloidogyne incognita and M. arenaria. Journal of Nematology 17(3):252-256.of

Tiilikkala, K, Cartert T, Heikinheimot M, Venalainent A, (1995) Pest risk analysis of *Meloidogyne chitwoodi* for Finland. *Bulletin EPPO Bulletin* 25, 419-435

Yang B and JD Eisenback (1983) Meloidogyne enterolobii n. sp. (Meloidogynidae), a Root-knot Nematode Parasitizing Pacara Earpod tree in China. Journal of Nematology, 15, 318-391.

**5. Pine wood nematode and pine wilt disease**

Author: Christelle Robinet (INRA, France)

**Information on the pine wood nematode**The pine wood nematode (PWN), *Bursaphelenchus xylophylus*, is transmitted by longhorn beetles of the genus *Monochamus*. In Europe, many species are potentially able to carry the pine wood nematode. They are widely distributed although not present in the United Kingdom and Ireland. The nematode was first detected in 1999 in Portugal on maritime pines where it is transported by *M. galloprovinciallis* (Mota et al. 1999).

Pine wilt disease (PWD) develops in susceptible species of *Pinus* under suitable climatic conditions (high summer temperature and drought), and the infected tree can wilt and die within weeks of being infested. This disease is caused by the pine wood nematode, but the presence of the nematode in a tree does not necessarily produce the symptoms of pine wilt disease.

Pine wilt disease distribution

Although the disease has been found in the presumed native area of the pine wood nematode, North America, the worst symptoms are observed in invaded countries in Asia (Japan, Korea, China) and more recently (since 1999) in Portugal. Drought and high summer temperature causing water stress in the host trees probably plays an important role in disease development but until now only thresholds defined by a correlation between disease occurrence, summer temperatures (e.g., mean temperature in July or August above 20°C) and the annual rainfall are given in the literature (Rutherford & Webster 1987).

Host range

PWN prefers *Pinus* species, but is also able to attack other Coniferae: *Abies*, *Picea*, *Larix*, *Cedrus* and *Pseudotsuga* (Evans et al. 1996). These genera are considered to be the PWN host plants. In this study, we used the distribution of *Pinus* species kindly provided by the European Forest Institute (EFI, Tröltzsch et al. 2009). All *Pinus* species are not equally susceptible, but this map provides a good indication of the host distribution for the PWN and the beetle vector.

CLIMEX file

The main problem in running the spread models is the absence of a CLIMEX model for pine wilt disease, the pine wood nematode or even for the beetle vector.

There were several options:

1. use the Mediterranean template of CLIMEX. However, the area where EI>0 is too restrictive.


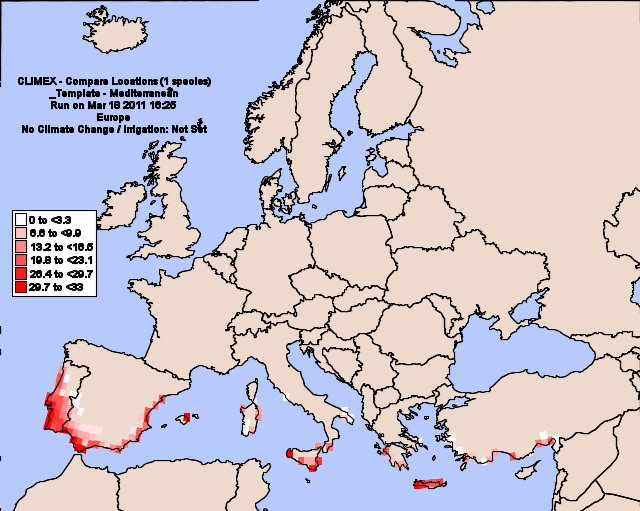


**Fig. 5-1:** CLIMEX Mediterranean template (EI)

1. use the temperate template of CLIMEX, but the area where EI>0 is probably too large and not appropriate


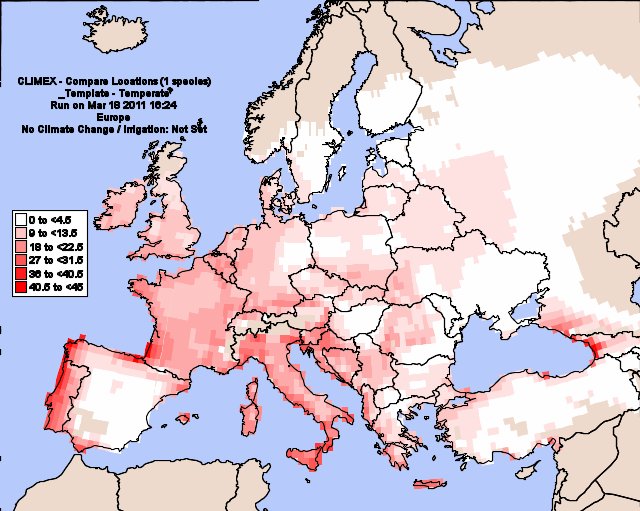


**Fig. 5-2:** CLIMEX Temperate template (EI)

The use of CLIMEX templates is however not advisable because it can only poorly predict the climatic suitability for a given species. Unless one template seems appropriate, it is generally recommended to stop and not apply the spread module if a specific CLIMEX model is not available.

1. adapt the Mediterranean template of CLIMEX for a better fit to Europe

In this case, we removed all the stress indexes and the moisture index. Parameters for the temperature were: DV0 = 13, DV1 = 20, DV2 = 30, DV3 = 35. Parameters for day-degree accumulation: MTS=7, DVCS=10, *DV4=100, DVHS=28, and degree days per generation (PDD=600) were unchanged from the Mediterranean template.

**
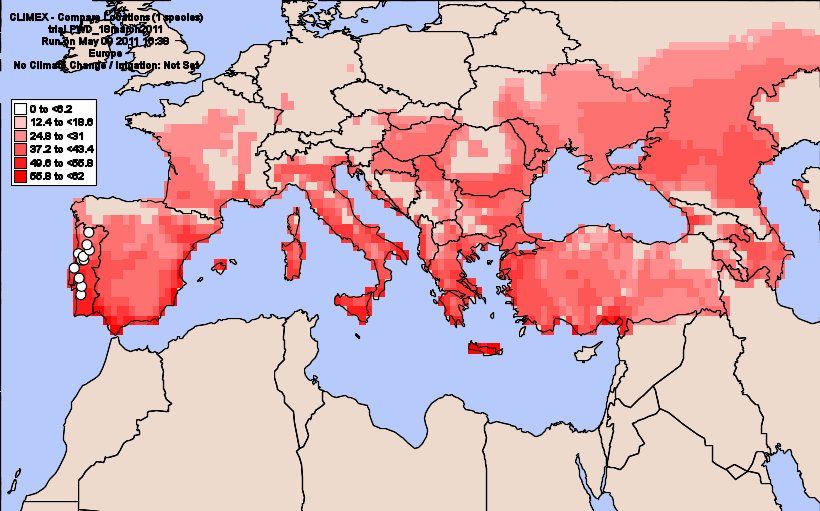

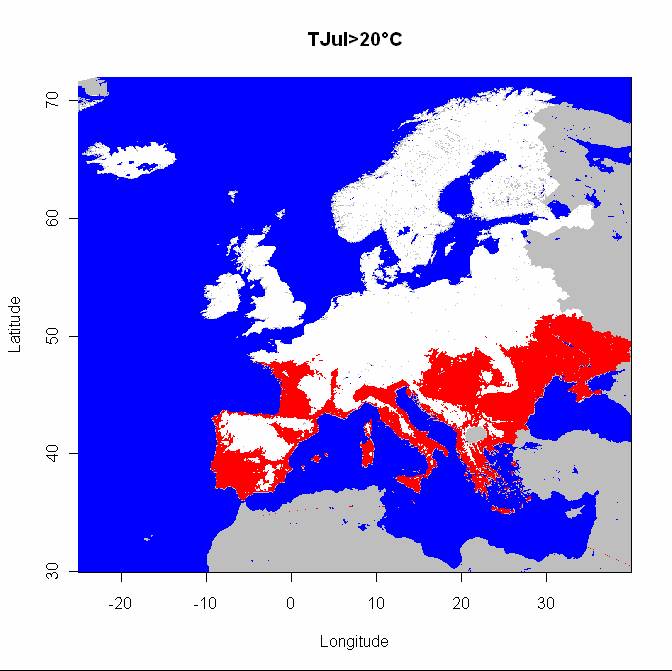
**

**Fig. 5-3:** Adjusted CLIMEX Mediterranean template (EI) (on the left) with presence of PWD (white dots) and the area where the mean July temperature is above 20°C (in red, on the right).

This choice for the CLIMEX model is a compromise between using simple but unrealistic global climate templates, and an adjusted template that is broadly in agreement with the northerly limits of PWD in eastern Asia (Japan, Korea and China). This simple adjusted model is globally in agreement with the map representing the areas where the mean temperature in July is above 20°C (interpolated with latitude, longitude and elevation, based on an average 1979-2008 climate, calculated from daily temperatures given by the European Climate Assessment & Dataset at <http://eca.knmi.nl/> , Fig. 5-3). Furthermore, it is also consistent with the occurrence of PWD in Asia and North America. **Although this model is used hereafter to apply the spread model, we do not pretend to have developed a CLIMEX model for the pine wilt disease.** We have just created a more appropriate map for PWD than the default climatic templates provided by CLIMEX to illustrate how the spread models can be applied to pine wilt disease. Any new CLIMEX model can be used in the future to enhance the predictions of the potential spread.

The following figures represent the maps used in R for the area of potential establishment (Fig. 5-4 left), the adjusted growth index (Fig. 5-4 right) and the habitat distribution density (Fig. 5-5). Information about the spread module is provided by the function printinfo() (Fig. 5-6)

**
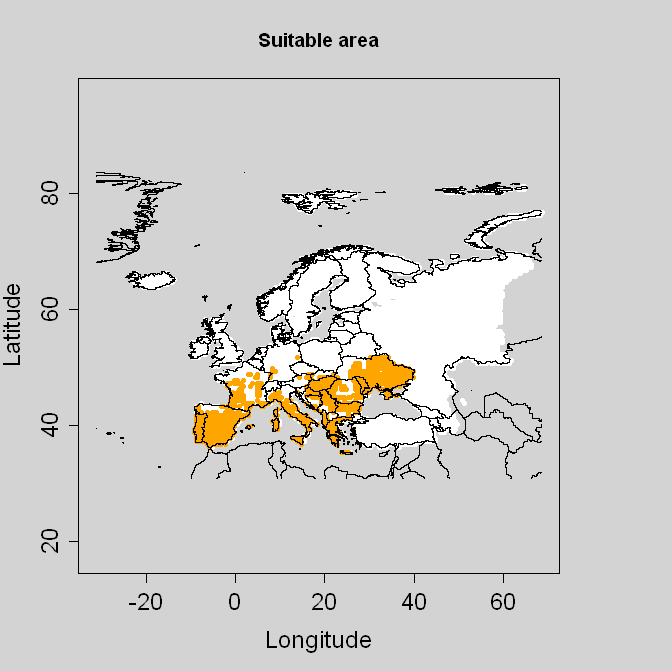

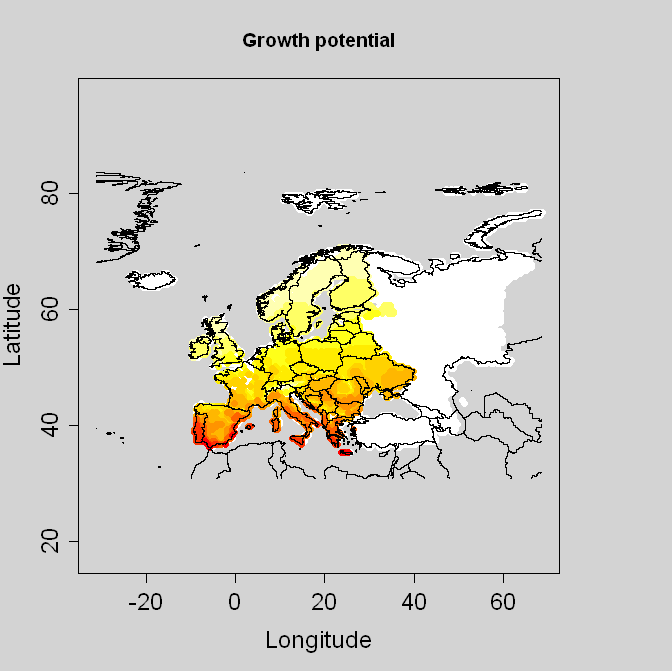
**

**Fig. 5-4**: Risk area on the left. The orange colour represents areas where EI>0 and the hosts are present. Growth potential on the right (adjusted GI) from 0 (light yellow) to 100 (red).

**
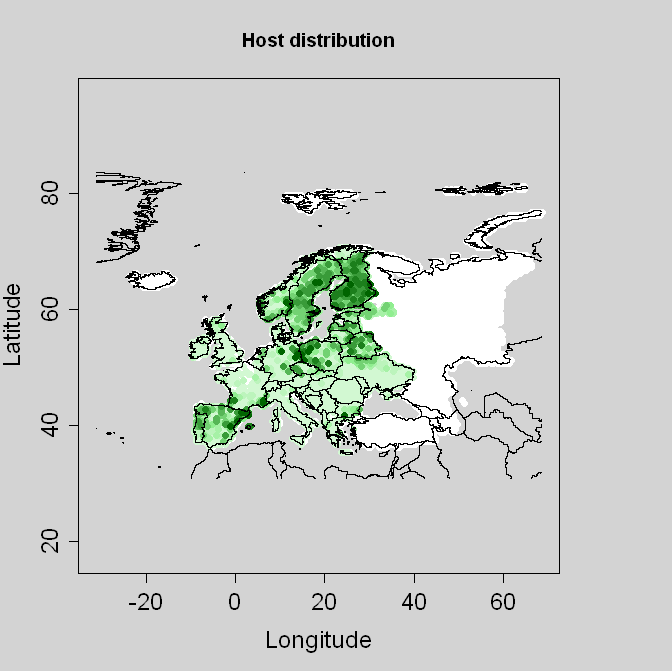
**

**Fig. 5-5:** Habitat distribution represented by the host tree density, or more precisely the proportion of land covered by *Pinus* species from 0 (light green) to 1 (dark green).

**
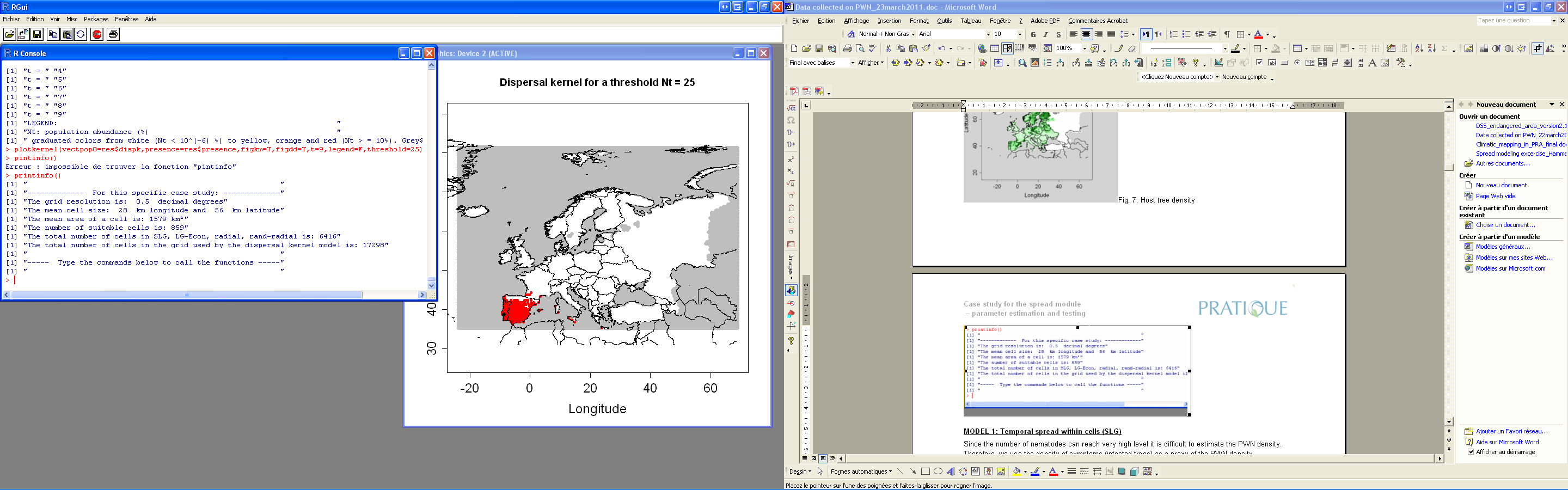
**

**Fig. 5-6:** Information provided by the spread module code.

**Model C: Population dynamics model**

Since the number of nematodes can reach a very high level it is difficult to estimate the PWN density. Therefore, we use the density of symptomatic trees as a proxy for the PWN density. This density probably underestimates the PWN density since symptoms are expressed only in susceptible trees.

- **Yearly multiplication factor λ_max_ :**

In Robinet et al. (2009), the following reaction-diffusion equation was used to simulate the spread and growth of the symptoms in China:

**
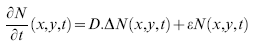
**

with ε = 2.17 ; exp(ε)=8.76

Here we assume that exp(ε) is the maximum growth of the population in China, and we also assume that this value is similar in China and in Europe. Therefore, we can take λ_max_ = 8.76 as the maximum yearly multiplication factor.

- **Initial population abundance p_0_ :**

This parameter is defined by:

$$p_{0}=100*\frac{P_{0}}{P_{\max}}$$

with *P*_0_ the number of introduced individuals, e.g. 100, in each suitable cell, and *P_max_* the carrying capacity (the maximum number of individuals in a cell).

From Rodrigues 2008, we have the following table.

**
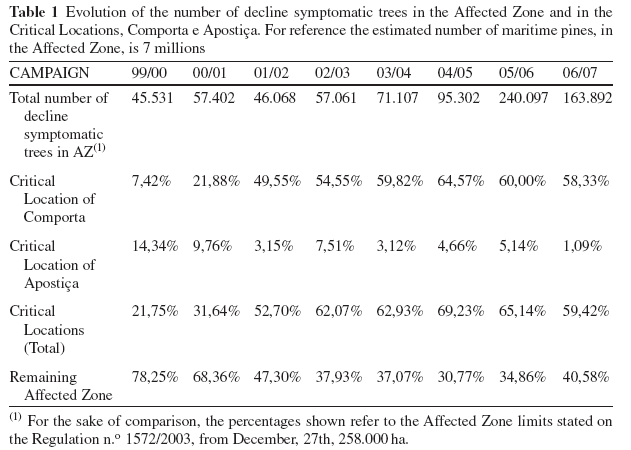
**

Since 2005-2006 was the most severe PWN infestation between 1999-2000 and 2006-2007 in terms of number of decline symptomatic trees, we assume that the pine wood nematode reached its maximum density.

In summary, 240 097 trees were infested out of 7 000 000 maritime pines in the 258 000 ha region in Portugal.

Therefore:

P_max_ = area of the cell (km²) * host plant density (/km²) * max pop density (/ host plant)

= 1579 * (7000000/258000*10^(-2) )* (240097/7000000)

P_max_ = 14.69

In 1999, 2 sites were initially infested in Portugal but the outbreak covered only one cell. We have assumed that two trees with symptoms was the initial infestation level. Therefore, P_0_ = 2 and p_0_ = 100 * 2 / 14.69. So p_0_ =13.61 %.

- **Time t :**

PWN was discovered in Portugal in 1999 and it spread considerably in 2008 (source: EPPO; Mota et al. 2009), therefore we can take t = 9 years to simulate spread in 2008.

**Results for Model C**


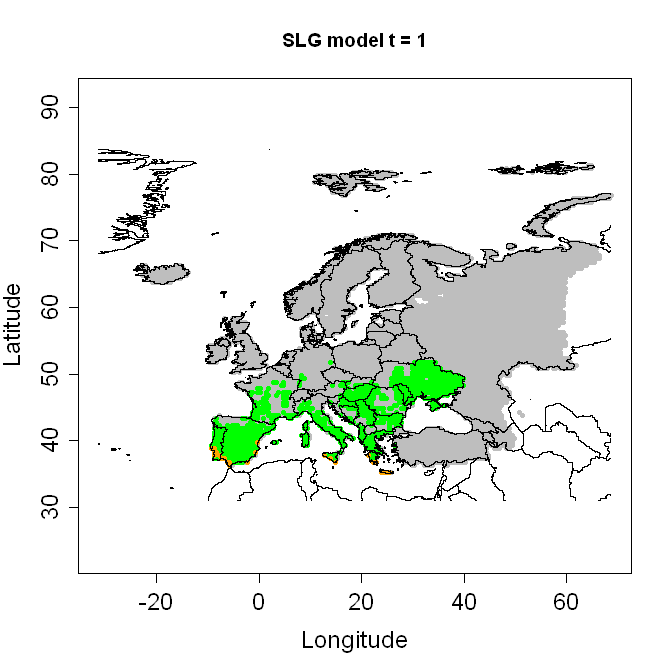

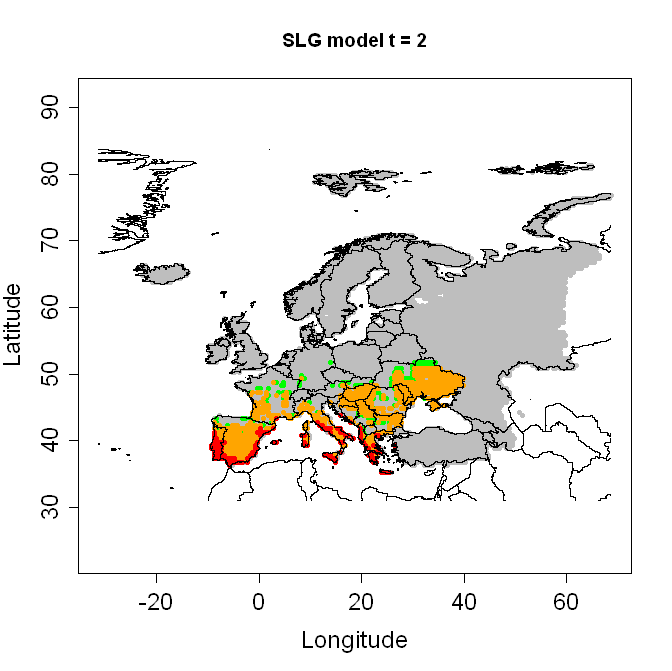


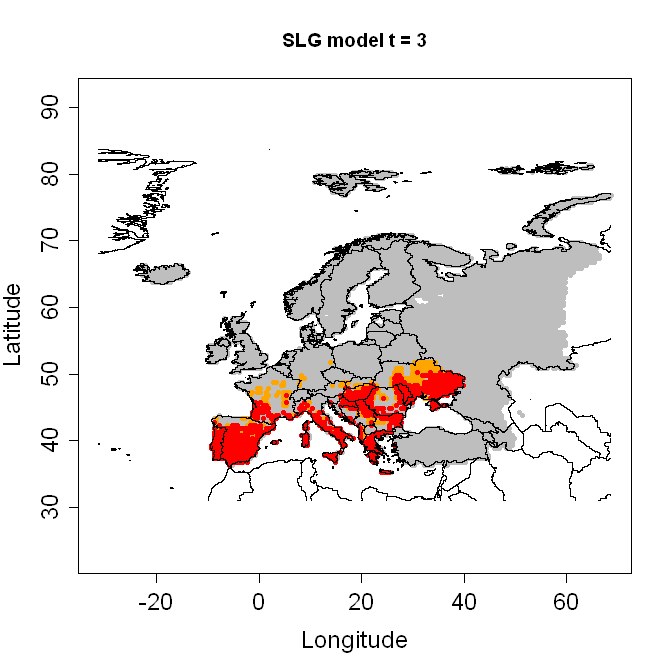

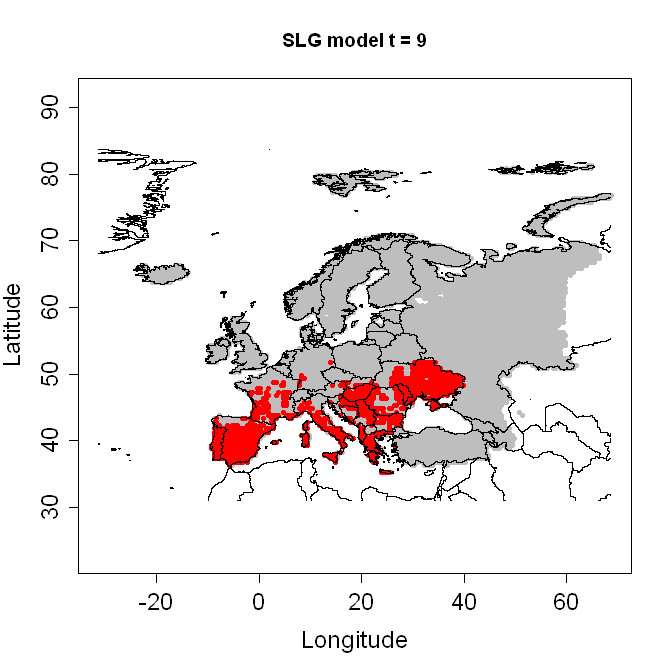


**Fig. 5-7:** Outputs of model C for t = 1, 2, 3 and 9 (blue dots: 0 < p_t_ < = 25, green dots: 25 < p_t_ < = 50, orange dots: 50 < p_t_ < = 75, red dots: 75 < p_t_ < = 100, grey dots: p_t_=0, white: no data).
*res = slg(N0=13.61,lmax=8.76,movie=F, t=9)*

This model shows that the most favourable place is southern Portugal and the PWD can grow very rapidly over all the area of potential establishment.

**Model A: Temporal spread over cells integrated with impact**

- **Initial number of infested cells N_0_:**

This parameter is defined by: *n_0_* = 100 * *N*_0_ / *N_max_*

*N*_0_ is the number of invaded cells at *t* = 0, and *N_max_,* the number of suitable cells.

The PWN was discovered at two sites separated by about 3 km (Mota et al 1999), covering only one cell. So N_0_ = 1 cell.

Besides, the number of suitable cells, *N_max_* = 859 (see Fig. 4.2.2-7)

- n_0_=1*100/859
- n_0_ = 0.12 %
- **Relative rate of spatial increase r :**

In 1999, 2 sites were initially infested within one cell. In 2008, 65 locations were infested in Portugal, covering 11 cells. To determine this number of cells, the function “pointtocell” was used.

Besides, the number of suitable cells is 859 (see Fig. 5-7).

Then we use the formula below to calculate r, with t = 9 (corresponding to the year 2008).

$$\begin{matrix} \ln\left(\frac{N_{t}}{N_{\max}-N_{t}}\right)=\ln\left(\frac{N_{0}}{N_{\max}-N_{0}}\right)+r\cdot t \\ \ln\left(\frac{11}{859-11}\right)=\ln\left(\frac{1}{859-1}\right)+9r \\ r=\frac{1}{9}\left(\ln\left(\frac{11}{848}\right)-\ln\left(\frac{1}{858}\right)\right)=0.27\text{ yr}^{\text{-1}} \end{matrix}$$

r = 0.27 yr^-1^

- **Time t :**

We chose t = 9 years (corresponding to the year 2008, since we take t = 0 to be 1999).

- **Economic dataset (valperhost):**

We have no precise GIS maps for economic value. From the value at a country scale, we tried to derive the value per km². If H(i) is the percentage of land covered by host trees (pines) and val is the value for each % of land covered by host tree per km², then we should sum{ H(i)} * val and make it equal to the country value to obtain val (euros per % of cells covered by pines). Finally, we should divide this number by the mean area of a cell (1579 km²). We obtain valperhost = 8 €.


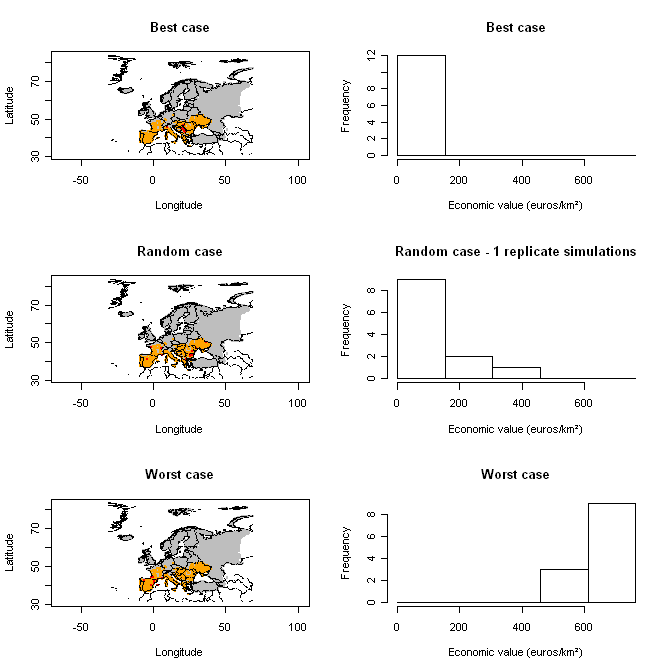


**Fig. 5-8** : Outputs of model A. On the left column is shown the invaded area (in red), non invaded area within the area of potential establishment (in orange), and area outside the area of potential establishment (in grey) for each of the three scenarios (best case, random case and worst case). White area is outside our study area (no data). On the right column, the number of invaded cells is given according to their economic value class for each of the three scenarios.
*res = lgecon(N0=0.12,r=0.27,t=9,econraster=F, hostraster=T,valperhost=8)*

Number of invaded cells = 12

Percentage of the niche invaded = 1.3 %

Economic impact = 25 € (best)– 2,265,000 € (random) – 18,306,372 € (worst)

This model shows that the worst case in terms of economic impact is the presence of the PWN in the Iberian Peninsula and southern France. Absolute economic values are not reliable since the economic value is based on a rough estimate of the economic value based on the proportion of cells covered by pines, and an assumption of a constant value over the PRA area and the economic value is set to 0 where data on hosts are missing. The main advantage of this model is to give an idea of the area where the economic impact would be higher and the magnitude of potential spread in terms of the number of invaded cells in this area. It also provides a range of possible economic impacts, showing whether the PRA area is relatively homogeneous or not concerning the economic impact.

**Model B: Radial range expansion model**

- **Radial rate of range expansion c:**

Short distance spread mediated by longhorn beetles was estimated at 7.5 km per year in China (Robinet et al 2009). In Japan it is estimated from 2 to 15 km/yr, with an average of 6 km/yr (Togashi & Shigesada 2006). c = 6 - 7.5 km/yr.

- **Time t :**

We can take t = 9 years to obtain simulations in 2008 that can be compared to the real situation (t = 0 in 1999, year of first detection and map available, and the model simulates the spread 9 years later, in 2008 and thus it is possible to compare the simulated spread and the real spread in Portugal).

- **Geographical coordinates coord:**

There were two introduction points in the Setubal Peninsula, approximately at:

Pegoes (38°41’00”N 008°36’43”W)

Landeira (38°35’46”N 008°38’58”W)

Therefore, coord = c(-8.61, 38.70, -8.65, 38.60)

**
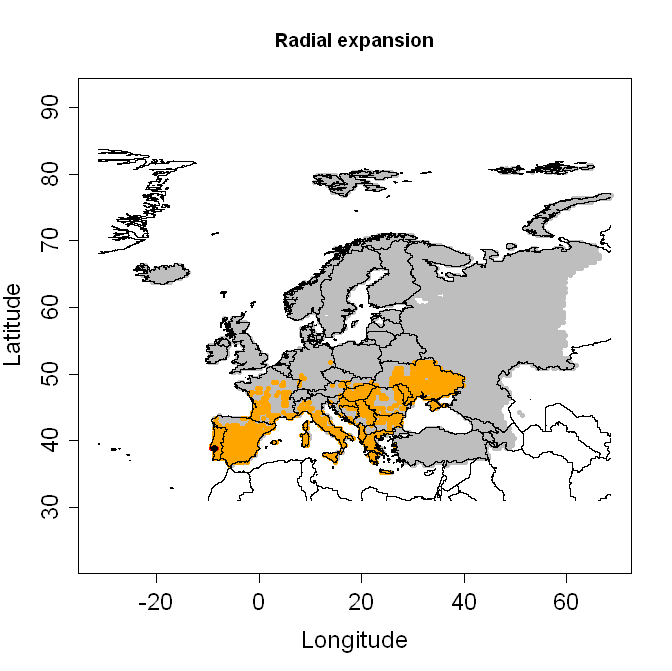

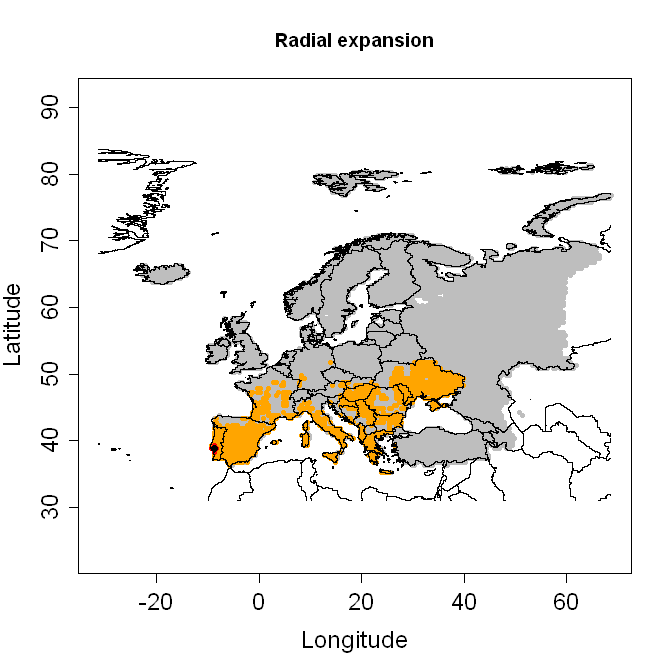
**

**Fig. 5-9a:** Outputs of model B for c = 6 km/yr on the left and c = 7.5 km/yr on the right (red dots: invaded cells, orange dots: non invaded but suitable cells, grey dots: non suitable cells, white: no data).
*res = radial(RR=6,t=9,coord=c(-8.61, 38.70, -8.65, 38.60),figkm=F, figdd=T)*

This model shows that the PWN cannot spread very fast if only short distance dispersal (e.g. the carrier beetle only disperses on its own) is taken into account. It shows that human mediated dispersal plays the major role in PWN spread.

If we increase the radial spread rate to obtain a satisfactory result for 2008 (i.e with the infestation covering nearly all Portugal with possible incursions in Spain), we have c = 35 km/yr approximately.

**
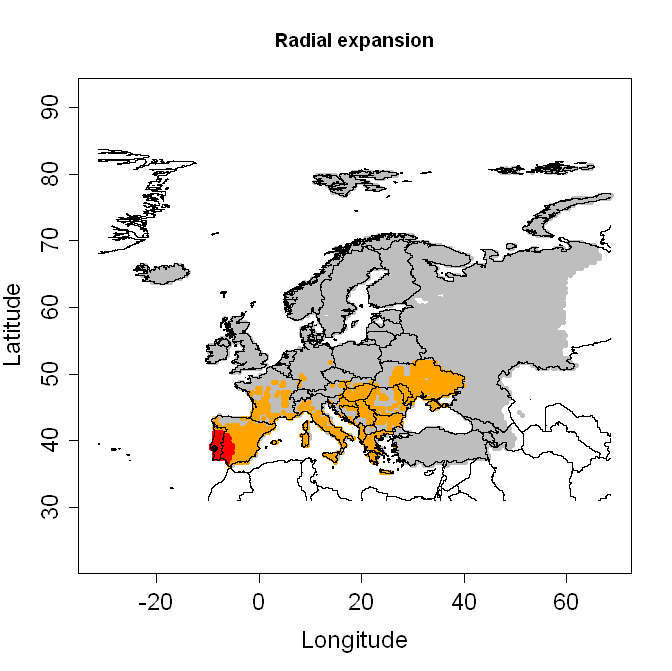
**

**Fig. 5-9b**. Outputs of model B for c = 35 km/yr (red dots: invaded cells, orange dots: non invaded but suitable cells, grey dots: non suitable cells, white: no data).

Spain is currently less affected than predicted by this map but this is not surprising given the early stage of the invasion process and the eradication strategy employed to contain the pest.

**Model D: Deterministic version of the dispersal kernel**

Most of the parameters have already been estimated in model C (λ_max_, p_0_, t) and B (u ≈ c).

We created a presence file with the entry points mentioned previously (in model B), and put p_0_ as the initial population density.


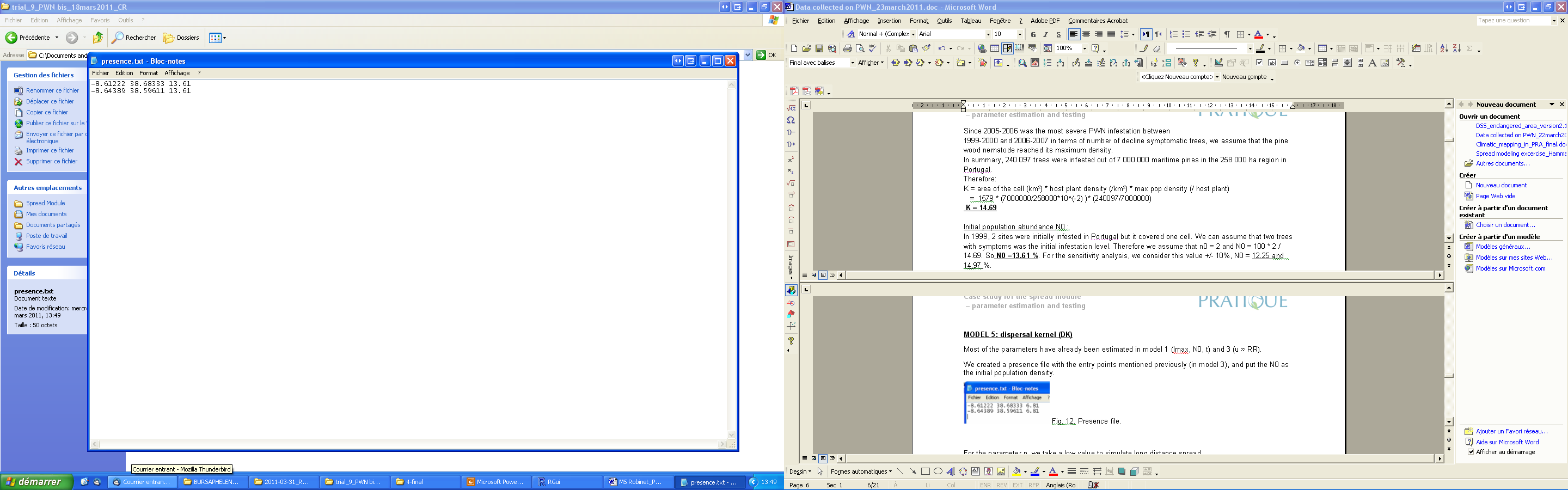
 **Fig. 5-10**. Presence file.

For the parameter υ, we take a low value to simulate long distance spread.

u = 35 (c estimated above)

υ = 5 (long distance spread)


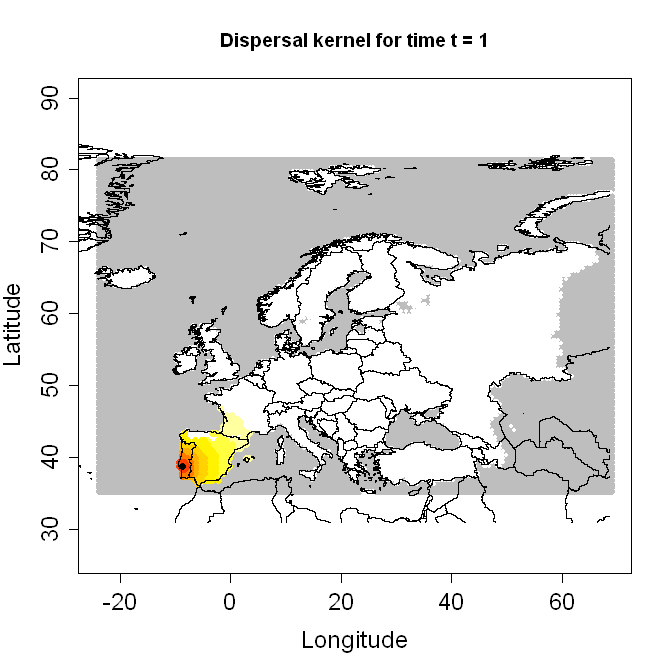

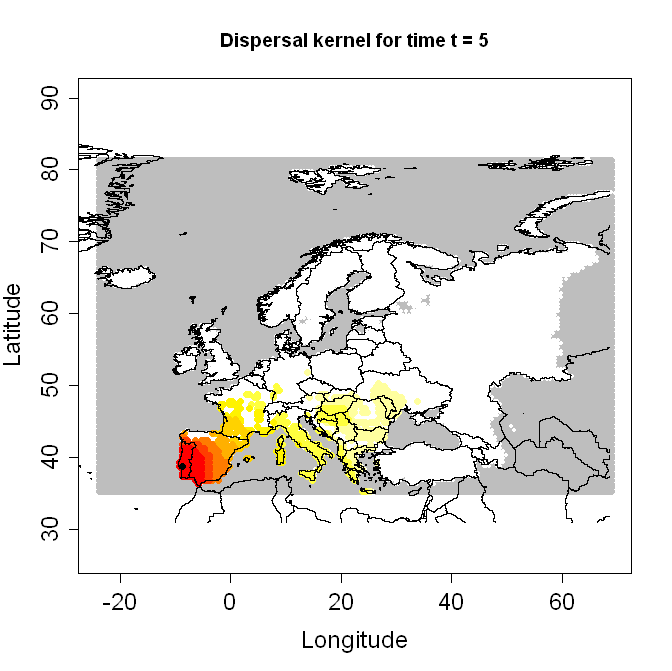


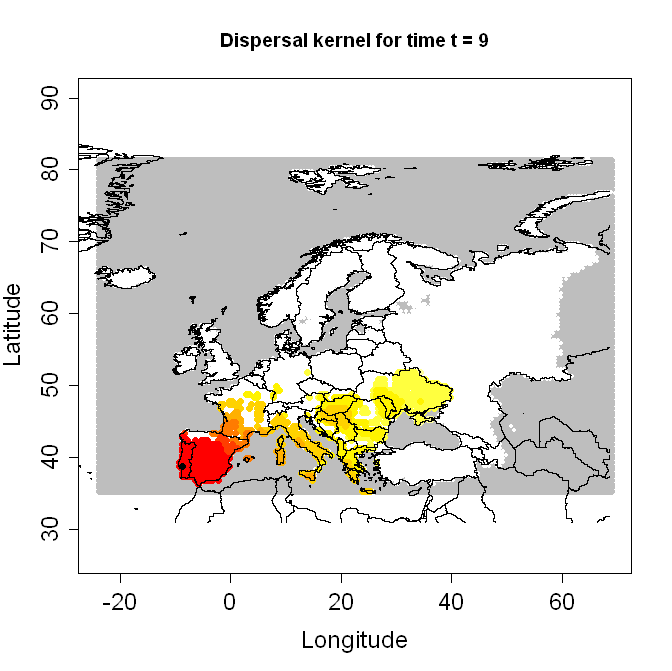


**Fig. 5-11a.** Outputs of model D for u = 35 and t = 1, 5, 9 (p_t_: population abundance (%); graduated colors from white (p_t_ < 10^(-6) %) to yellow, orange and red (p_t_ > = 10%). Grey means no data).
*res = dispk(N0=NULL, t=9,lmax=8.76, p=5,u=35, presencefile=T, nentry=NULL, figkm=F,figdd=T)*

**Fig. 5-11b**. Outputs of model D for u = 35 and t = 9 (p_t_ > 25 %). *plotkernel(vectpop0=res$dispk,presence=res$presence,figkm=T,figdd=T,t=9,legend=F,threshold=25)*

For comparison, we made simulations for a lower u, based on the initial spread distance found in China:

u = 7.5 (c for short distance spread in China)

υ = 5 (long distance spread)

**Fig. 5-12a**. Outputs of model D for u = 7.5 and t = 1, 5, 9 (p_t_: population abundance (%); graduated colors from white (p_t_ < 10^(-6) %) to yellow, orange and red (p_t_ > = 10%). Grey means no data).
*res = dispk(N0=NULL, t=9,lmax=8.76, p=5,u=7.5, presencefile=T, nentry=NULL, figkm=F,figdd=T)*

**Fig. 5-12b**. Outputs of model D for u = 7.5 and t = 9 (p_t_ > 25 %). *plotkernel(vectpop0=res$dispk,presence=res$presence,figkm=T,figdd=T,t=9,legend=F,threshold=25)*

When u is decreased, the population does not disperse as far and the population density increases. Therefore, the population density can build up more rapidly and the pest can then spread more rapidly instead of being diluted in the PRA area. Therefore decreasing u makes the spread rate increase.

This dispersal kernel model assumes that the population can spread continuously from short to long distances whereas, in reality, some places will remain uninfested because man will move some individuals from one place to another and not in-between. Thus, there will be some gaps in the distribution and this pattern cannot be simulated by model D (nor the previous ones).

**Summary**

In Europe, (a) it is too early in the invasion process to estimate and adjust the parameters based on the observations of spread, and (b) the rate of spread is likely to have been affected by the strategy undertaken to contain and eradicate the pest.

Model C is useful to show how fast PWD can grow and which regions are the most favourable. Model A is useful to provide a range of possible economic impacts and indicate the area where the highest impact is expected (although the economic impact predictions are unreliable because economic value is not available at the grid’s spatial resolution). Model B shows that long distance spread should be taken into account and we can estimate the overall radial spread. Model D is not appropriate to describe long distance dispersal due to human transportation.

**Commands for modelling the spread of pine wood nematode** (or more precisely pine wilt disease symptoms)

library(sp)

library(raster)

library(rgdal)

elevmax = F

habitatfile = T

*# load the R code*

plothabitat()

plotRA()

plotGI()

res = slg(N0=13.61,lmax=8.76,movie=F, t=9)

res = lgecon(N0=0.12,r=0.27,t=9,econraster=F, hostraster=T,valperhost=8)

res = radial(RR=35,t=9,coord=c(-8.61, 38.70, -8.65, 38.60),figkm=T, figdd=F)

res = dispk(N0=NULL, t=9,lmax=8.76, p=5,u=35, presencefile=T, nentry=NULL, figkm=F,figdd=T)

plotkernel(vectpop0=res$dispk,presence=res$presence,figkm=T,figdd=T,t=10,legend=F,threshold=25)

**References**

Evans HF, McNamara DG, Braasch H et al (1996) Pest risk analysis (PRA) for the territories of the European Union (as PRA area) on Bursaphelenchus xylophilus and its vectors in the genus Monochamus. EPPO Bull 26:199-249.

Mota MM, Braasch H, Bravo MA et al (1999) First report of Bursaphelenchus xylophilus in Portugal and in Europe. Nematology 1:727-734.

Mota MM, Futai K, Vieira P (2009) Pine wilt disease and the pinewood nematode, Bursaphelenchus xylophilus. In: Ciancio A, Mukerji KG (eds). Integrated management of fruit crops and forest nematodes. Springer science, pp 253-274. DOI: 10.1007/978-1-4020-9858-1_11.

Robinet C, Roques A, Pan HY, Fang GF, Ye JR, Zhang YZ & Sun JH (2009) Role of Human-Mediated Dispersal in the Spread of the Pinewood Nematode in China. Plos One 4, e4646. doi:10.1371/journal.pone.0004646

Robinet C, Van Opstal N, Baker R, Roques A (2011) Applying a spread model to identify the entry points from which the pine wood nematode, the vector of pine wilt disease, would spread most rapidly across Europe. Biological Invasions, 13:2981-2995.

Rodrigues JM (2008) National eradication programme for the pinewood nematode. In: Mota MM, Vieira PR (Eds) Pine wilt disease: a worldwide threat to forest. Springer Science + Business Media B.V., ISBN: 978-1-4020-8454-6, pp 5-14.

Rutherford TA, Webster JM (1987) Distribution of pine wilt disease with respect to temperature in North America, Japan, and Europe. Can J For Res 17:1050-1059.

Togashi K, Shigesada N (2006) Spread of the pinewood nematode vectored by the Japanese pine sawyer: modeling and analytical approaches. Popul Ecol 48: 271-283.

Tröltzsch K, Van Brusselen J, Schuck A (2009) Spatial occurrence of major tree species groups in Europe derived from multiple data sources. For Ecol & Manag 257: 294-302. doi:10.1016/j.foreco.2008.09.012.

**6. Water hyacinth, Eichhornia crassipes**

Authors: Hella Kehlenbeck (JKI, Germany) and Sarah Brunel (EPPO, Paris)

**Information on *E. crassipes***

*E. crassipes* is considered to be one of the worst aquatic invasive plants worldwide. In Europe it already poses a threat in Spain and Portugal, but its distribution is still limited in the EPPO region. Data were derived from the EPPO PRA on *E. crassipes* of 2008.

Natural spread potential

The natural spread of up to hundreds of kilometres has been documented in many of the large river systems in Africa and in Spain. Studies in Spain showed that propagule dispersal was highest in September with an average of 4.5 propagules dispersed in 15 minutes. However, such dispersal is confined within to the same catchment.

Human-assisted spread potential

*E. crassipes* can be accidentally spread by human activities during the maintenance of swimming areas and drainage ditches and attached to fishing gear or to hulls, anchor lines, engines, or other parts of boats both within the same catchment and between catchments. Moreover, existing practices of mechanical waterway maintenance tend to cut off plants and to spread the fragments. These fragments thrive and form new plants, enabling new populations to develop.

Long distance spread is enhanced because Water Hyacinth is intentionally imported as an ornamental aquatic plant for use outdoors.

Host range and habitat distribution, here

The habitats at risk are freshwater bodies and ecosystems which are very common throughout the EPPO region. These have not been included in the spread models. Ideal habitats for *E. crassipes* are slow moving or still freshwater bodies and ecosystems.

According to the CORINE Land Cover nomenclature, the suitable habitats are:

- Continental waters (water courses, water bodies)

- Banks of continental water, Riverbanks / canal sides (dry river beds)

The land cover by country can be explored at <http://www.eea.europa.eu/data-and-maps/figures/corine-land-cover-2006-by-country> . See also PRATIQUE Deliverable 3.3.

Findings of *E. crassipes* in Europe *(to be used as potential starting points for the modelling)*

The plant is established in Italy, Portugal, and Spain. It is casual (= transient) in France, the Netherlands, Belgium and the UK. *E. crassipes* also occurs in botanical gardens, e.g the Botanic Gardens of Amsterdam (The Netherlands), Cologne (Germany) and in Brno (Czech Republic). It has been reported from Slovakia where it is cultivated during the summer in gardens. It does not thrive in these countries and is replanted every year. It might be more widely spread as it is freely available in shops, markets and on the internet. *E. crassipes* is widely grown as an ornamental in UK glasshouses.

Climatic suitability in Europe

The climatic conditions in southern Europe are very similar to those in its native range but are totally dissimilar to those in northern Europe. Optimal growth occurs at temperatures of 28-30°C (air temperatures) while growth ceases when water temperatures drop below 10ºC. The whole Mediterranean area would be suitable for *E. crassipes*. In the more temperate regions of Europe it is more likely that transient populations of the weed may occur. Populations can expand during the summer months, but die back during winter, as in canals in the Netherlands*.*

CLIMEX file

A CLIMEX model for this species was provided by Sarah Brunel and Darren Kriticos. Figure 6-1 shows the area at risk and the growth potential for *E. crassipes* according to CLIMEX.

**Figure 6-1**: Suitable area (on the left; orange dots: EI>0, white dots: EI=0, grey: no data) and growth potential (GI; on the right) for *E. crassipes* in Europe.

Based on the CLIMEX model the number of suitable cells for *E. crassipes*

is given below:

[1] "The number of suitable cells is: 252"

[2] "The total number of cells in SLG, LG-Econ, radial, rand-radial is: 6416"

[3] "The total number of cells in the grid used by the dispersal kernel model is: 17298"

Reproduction strategy of *E. crassipes*

*E. crassipes* grows very fast. Another species is not needed to complete the life cycle of the plant and the plant is able to reproduce vegetatively. In Spain (River Guadiana), *E. crassipes* reproduces both vegetatively and sexually and has floral cycles of about 1-2 days; 1-2 months rae required to produce mature dehiscent fruits and seeds. Its doubling time can be as little as one week, and depends on water nutrient content and temperature. In the Guadiana river in Spain, doubling time varied between 10 and 60 days.

Each flower of *E. crassipes* produces about 250 long-lived seeds (up to 20 years) that are resistant to the drying up of the water body. Germination occurs once the water body is re-inundated and the plants are then capable of rapid growth through asexual production of daughter plants

It is thought that *E. crassipes* was introduced to a number of countries as a single plant from which infestations have arisen.

**Model C: Population dynamics model**

*For the population dynamics model it is assumed that an initial population abundance p_0_ (%) is introduced in each suitable cell (cells where EI > 0) and then we simulate the “spread” or more precisely the growth within each cell according to a logistic function. On the output map, we will see areas which are the most suitable for the population growth if some individuals were introduced there.*

**Estimates of parameters**

- **The starting population p_0_**

*p_0_ is the population abundance for all suitable cells at time t = 0 expressed as a percentage of the maximum abundance (carrying capacity K).*

*Calculation:*

$p_{0}=100*\frac{P_{0}}{P_{\max}}$ *with P_0_ the number of introduced individuals, in each suitable cell, and P_max_ the carrying capacity (the maximum number of individuals in a cell).*

*P_max_ = area_cell (km²) * proportion_covered_by_host * max_population_density (/km²), or
P_max_ = area_cell (km²) * host_plant_density (/km²) * max_population_density (/host plant)*

Relevant information and data:

On a length of 75 km in a river in Africa, 200,000 tonnes of *E. crassipes* has been observed.

Considering an average width of 60 m of the river, the surface area of these 75 km of river was calculated to be 4.5 km². The carrying capacity of this river is therefore calculated to be *P_max_* =200000 ton/4.5 km².

It is assumed that about 5% of the surface is covered with water.

Calculation of the carrying capacity *P_max_* :

*P_max_* = 44444 tons / km².
*P_max_* = 44444 tons / km² x 1579 km² (size of the grid cell)

*P_max_* = 7.02*10^7^ tons / grid cell

Calculation of the starting population p_0_:

Initial density P_0_ = 500 g of *E. crassipes* (or 0,0005 tons)

5% of the surface is covered with water

p_0_ = 100 x 0,0005 t / 7.02*10^7^ x 0.05

p_0_ = 3,6 *10^-11^ %

- **The multiplication factor λ_max_**

*λ_max_ is the maximum year to year multiplication factor (“finite growth rate”) that a population could achieve under optimal conditions assuming unlimited space*

Relevant information and data

According to the literature provided by Sarah Brunel, Water Hyacinth develops during the growing season from 1 kg to about 30 kg within one year.

λ_max_ = 30

- **Time frame:** 10 and 20 years

**Results**

p_0_ = 3.6*10^-11^; λ_max_=30

p_0_ = 3.6*10^-11^; λ_max_=30

**Figure 6-2**: Output of Model C for *E. crassipes* *for* 10 and 20 years ( blue dots: 0 < pt < = 25, green dots: 25 < pt < = 50, orange dots: 50 < pt < = 75, red dots: 75 < pt < = 100, grey dots: pt=0, white: no data).

*Left Picture: 10 years ; R:> res = slg(N0=3.6*10^(-11),lmax=30, t=10)**Right Picture: 20 years; R:> res = slg(N0=3.6*10^(-11),lmax=30, t=20)*

|  |  |
| --- | --- |

The results of the population dynamics model reflect what is expected according to the CLIMEX model.

**Model B: Radial Range expansion model**

*This model aims to determine the potential spread of a species introduced in the PRA area based on the radial rate expansion parameter. The model output is overlapped with the niche map (EI>0).*

**Estimates of parameters**

- **Radial rate of range expansion per year**

c = 50 km/year

To consider as well human assisted spread, a value of c= 100 km / year was used.

- **Time frame**: t = 10 – 20 years
- **Entry point(s)** or simulation of an introduction at a place

- Southern Spain (-6.2,38.5), where the species is present

**Results**

**a) short distance spread (c=50 km/year)**

**c=50 km/yr: 35.7% of the risk area invaded**

**t=10 90 cells invaded**

**c=50 km/yr: 49.2% of the risk area invaded**

**t=20 124 cells invaded**

**Figure 6-3**: Radial range expansion of *E. crassipes* for t=10 and 20 years, c=50 km/year and an entry point in Southern Spain (red dots: invaded cells, orange dots: non invaded but suitable cells, grey dots: EI=0, white: no data)

*Left picture: 10 years, R:> res = radial(RR=50,t=10,coord=c(-6.2,38.5))
right picture: 20 years, R:> res = radial(RR=50,t=20,coord=c(-6.2,38.5))*

**b) long distance, human assisted spread (c=100 km/year)**

**c=100 km/yr: 49.2% of the risk area invaded**

**t=10 124 cells invaded**

**c=100 km/yr: 76.8% of the risk area invaded**

**t=10 193 cells invaded**

**Figure 6-4**: Radial range expansion of *E. crassipes* for t=10 and 20 years, c=100 km/year and an entry point in Southern Spain (red dots: invaded cells, orange dots: non invaded but suitable cells, grey dots: EI=0, white: no data)

*Left picture: 10 years, R:> res = radial(RR=100,t=10,coord=c(-6.2,38.5))
right picture: 20 years, R:> res = radial(RR=100,t=20,coord=c(-6.2,38.5))*

The Radial Range Expansion Model maps the spread of *E. crassipes* with acceptable results.

**Model D: Deterministic version of the dispersal kernel**

*This type of model requires: a population growth model, a dispersal kernel (2Dt in this case) and the proportion of population engaged in dispersal.*

Estimates of parameters

- **The starting population p_0_**

*N_0_ is the population abundance for all suitable cells at time t = 0 expressed as a percentage of the maximum abundance (carrying capacity P_max_).*

p_0_ = 3.6 x 10^-11^ %

- **The multiplication factor λ_max_**

*λ_max_ is the maximum year to year multiplication factor (“finite growth rate”) that a population could achieve under optimal conditions assuming unlimited space*

λ_max_ = 30

- **Shape parameter of the 2Dt dispersal kernel υ**

*(number of degrees of freedom)*

*For* υ *= 1, this kernel has a Cauchy distribution (thick tail; a large number of individuals disperses further than 3σ) and for* υ$\to+\infty$*, it has a normal distribution (thin tail; individuals disperse at short distance).*

- if we take into account more natural spread we have short distance dispersal if only and apply υ=50

- if we also take long distance dispersal into account (e.g. with transport of plant material) we apply υ=10

- **Scale parameter for the distance of the 2Dt dispersal kernel (u)**

Here we use u = 50 km

- **presence file**

*The presence file contains the coordinates for*

*the entry point and p_0_*

p_0_ = 3.6 x 10^-11^ %

**Results**

**a) short distance spread (**υ**= 50)**

**p_0_ = 3.6 x 10^-11^ % , λ_max_ = 30, υ= 50, u= 50, t=10 years, 0% of the risk area invaded**

**t=10 0 cells invaded**

**Results**

**a)**

**Figure 6-5**: Output of the dispersal kernel model; p_0_ = 3.6*10^-11^and λmax = 30 for 10 years *(R:>* *res = dispk(t=10,lmax=30,p=50,u=50)).*

Left Picture: (pt: population abundance (%); graduated colors from white (pt < 10^(-6) %) to yellow, orange and red (pt > = 10%). Grey means no data)

Right picture: with a threshold = 25 %. The population abundance (%) is above this threshold in red cells. (red dots: cells where pt > = threshold, white: cells where pt < threshold. Grey means no data)

Right picture: with a threshold = 25 %. The population abundance (%) is above this threshold in red cells. (red dots: cells where Nt > = threshold, white: cells where Nt < threshold. Grey means no data)

**p_0_ = 3.6 x 10^-11^ % , λ_max_ = 30, υ= 50, u= 50, t=20 years, 41.2% of the risk area invaded**

**t=20 104 cells invaded**

**Figure 6-6:** Output of the dispersal kernel model; p_0_ = 3.6*10^-11^and λmax = 30 for 20 years *(R:>* *res = dispk(t=20,lmax=30,p=50,u=50)).*

Left Picture: (pt: population abundance (%); graduated colors from white (pt < 10^(-6) %) to yellow, orange and red (pt > = 10%). Grey means no data)

Right picture: with a threshold = 25 %. The population abundance (%) is above this threshold in red cells. (red dots: cells where pt > = threshold, white: cells where pt < threshold. Grey means no data)

**b) long distance spread (υ= 10)**

**p_0_ = 3.6 x 10^-11^ % , λ_max_ = 30, υ= 10, u= 50, t=20 years, 41.7 % of the risk area invaded**

**t=20 105 cells invaded**

**Figure 6-7:** Output of the dispersal kernel model; p_0_ = 3.6*10^-11^and λmax = 30 for 20 years *(R:>* *res = dispk(t=20,lmax=30,p=10,u=50).*

Left Picture: (pt: population abundance (%); graduated colors from white (pt < 10^(-6) %) to yellow, orange and red (pt > = 10%). Grey means no data)

Right picture: with a threshold = 25 %. The population abundance (%) is above this threshold in red cells. (red dots: cells where pt > = threshold, white: cells where pt < threshold. Grey means no data)

The application of the Dispersal Kernel Model is felt to be less intuitive compared to the other two tested models. The higher number of parameters and the number of the resulting maps after applying the sensitivity analysis is a little bit confusing.

Table 6-1 summarizes the results of the sensitivity analysis for the different parameters of the dispersal kernel model. While changes in the starting population p_0_ and of the shape parameter **υ** do not lead to changes in the numbers of invaded cells after 20 years, the multiplication factor λmax and the scale parameter reduce the number of invaded cells by 9% by using lower parameter values.

*E. crassipes* is a species with a rather small range of suitability in Europe (252 suitable cells within the spread module). This could be one reason that the changes in the parameters do not lead to very big changes in the output of the models.

**Table 6- 1:** Summary of results from the sensitivity analysis as reflected in the number of invaded cells and percentage of invaded area for a time frame of 20 years

| Parameter | Lower value | | | Higher value | | |
| --- | --- | --- | --- | --- | --- | --- |
|  | **Change compared to “normal”** | **Number of invaded cells** | **Relative to normal  (normal =104)** | **Change compared to “normal”** | **Number of invaded cells** | **Relative to normal** |
| Multiplication factor λ_max_ | - 50% | 95 | 91% | + 50% | 107 | 103% |
| p_0_ | - 50% | 103 | 99% | + 50% | 105 | 101% |
| Shape parameter υ | **More long distance (**υ**=10)** | 105 | 101% | **More short distance (**υ**=70)** | 104 | 100% |
| Scale parameter u | - 40% | 95 | 91% | + 40% | 105 | 101% |

**Summary**

The application of the population dynamics model C is useful to show how fast *E. crassipes* can grow without any control measures and which regions are the most favourable ones. The Radial Range expansion model B shows that if long distance spread is taken into account almost the whole suitable area will be invaded after 20 years. Model D maps the spread of *E crassipes* mainly in Spain and Portugal. *E. crassipes* is a species with a rather small range of suitability in Europe. Therefore the range of expansion for this pest is already very limited by the habitat suitability. Changes in parameters do not lead to big changes in the output of the Dispersal Kernel Model.

**Summary of the commands for modelling the spread of *E. crassipes:***

library(sp)

library(raster)

library(rgdal)

elevmax = F

hostfile = F

*# load the R code*

res = slg(N0=3.6*10^(-11),lmax=30,t=10)

res = radial(RR=50,t=10,coord=c(-6.2,38.5))

res = dispk(N0=NULL, t=10,lmax=30, p=50,u=50, presencefile=T, nentry=NULL, figkm=F,figdd=T)

**7. Pitch canker disease, *Gibberella circinata***

Authors: Christelle Robinet and Annie Yart (INRA, Orléans)

**Information on *Gibberella circinata***Fungi, Ascomycota, Hypocreales, Nectriaceae.

Teleomorph: *Gibberella circinata* Nirenberg et O'Donnell

Anamorph: *Fusarium circinatum* Nirenberg et O'Donnell

Synonyms: *Fusarium subglutinans* f. sp. *Pini* Hepting*, Fusarium moniliforme* Sheldon var. s*ubglutinans* Wollenweber*, Fusarium lateritium* f. sp. *Pini* Hepting.

*Gibberella circinata* is the causal agent of Pitch canker disease which can affect all *Pinus* species but also Douglas fir (*Pseudotsuga menziesii*) (Anonymous 2009). This disease, causes cankers that girdle branches and is a serious threat to the pine forests because tree mortality may occur after multiple branch infection. Moreover, *Gibberella circinata* may also be soil-borne, cause root rot even on mature trees (Garbelotto et al. 2007) and infect seeds externally or internally (without any symptoms before seed germination).

The anamorph, *Fusarium circinatum,* is a wound pathogen and may occur after mechanical wounds (Sakamoto & Gordon 2006) as well as after wood boring insect damage (Anonymous 2009). Tree infection occurs by aerial dispersion of conidiospores or by feeding insect vectors (Gordon et al. 2001, Schweigkofler et al. 2004).

*Gibberella circinata* is officially reported in USA, Mexico, Haiti, South Africa, Japan, Chile, Iraq, Japan, Kyushu, Ryukyu Archipelago, South Africa (EPPO 2005, EFSA 2010), and also in Europe: Spain, France, Portugal and Italy, mainly in coastal areas Anonymous 2009, EFSA 2010).

Pitch canker has been described in several regions of Spain but always as isolated outbreaks originating from nurseries (EPPO 2005 and 2006a).

It was first reported in France in 2006 on declining pines and Douglas fir (EPPO 2006b) and visual inspection combined with laboratory tests confirmed pest eradication (EPPO 2008). But new isolated outbreaks (Vosges 2008 and Vendée, Côtes d'Armor, EPPO 2009a) were reported and studies were initiated to identify the origin of the infection (EPPO 2009a and 2010).

During the same period, *Gibberella circinata* dieback symptoms in Italy were identified on the basis of morphological and cultural characteristics confirmed with PCR with specific primers (Carlucci et al. 2007) and the fungus was eradicated (EPPO 2009b) while in Portugal its presence on symptomatic plant samples was confirmed by PCR and pathogenicity tests after a first identification based on morphological and cultural characteristics (EPPO 2009c, Bragança et al. 2009).

Host range

*Pinus* species

*Pseudotsuga menziesii*

CLIMEX file

A CLIMEX model was applied in Ganley et al. (2009). Parameters’ values were used in CLIMEX and the required file was exported (with latitude, longitude, EI and GI). This model fitted well with the observed disease distribution. However, when using a more recent climate (1999-2007) instead of the past climate (1961-1990) or using an increased spatial resolution, the model predicted favourable conditions in larger areas (EFSA 2010). Therefore caution is needed when using this model and interpreting the results of this study.

**Comments on the difficulties**

It is difficult to find quantitative data to estimate P_max_ and *λ_max_*. There are perhaps several reasons for this:

- the number of spores is extremely high in an infected tree (some millions), so it is impossible to determine the “population density” to estimate P_max_ and *λ_max_*.
- as a proxy, we could take the number of trees infected by the disease, but we have no clear information on this. Moreover, there is a latent infection period.

It is also difficult to estimate the spread rate because the infection is usually transmitted from one nursery to another one, and because of control actions. There is long-distance dispersal resulting from trade, humans themselves, or machines carrying the pathogen. The disease can also spread by natural means (wind, rain, insect vector) (EFSA 2010). However, trees in nurseries are under surveillance and as soon as an infection is detected, some eradication measures are applied and the invaded area is contained. Based on the invasion history, there is few short distance (spatial extent of the disease from one introduction point) but relatively more long distance spread from one nursery to another (several introduction points). The latent period probably makes it difficult to detect the pathogen before sale and transport. Consequently, it seems that model B is not appropriate and model D could be tested with small u (few short-distance dispersal events) and small υ (thick tail – high proportion of long-distance spread). However this model will not take into account the observations that dispersal occurs mainly between nurseries and it may overestimate spread in Europe.

**Qualitative assessment of spread**

Estimating spread qualitatively is also difficult. In the available PRA (done in 2000, see: <http://www.eppo.org/QUARANTINE/Pest_Risk_Analysis/PRA_documents.htm>), it seems that the spread of G circinata could be very fast at both short and long distances but no precise information is given about the possible spread rate. The responses to the spread questions in the EPPO DSS for PRA version 2011
(<http://archives.eppo.org/EPPOStandards/PM5_PRA/PRA_scheme_2011.doc>) are given below.

| - 1. **What is the most likely rate of spread by natural means (in the PRA area)?**   ***Note:*** Natural population spread, increasing the infested area, can result from the movement of the pest by flight (of an insect), wind or water dispersal (except irrigation), transport by vectors such as insects, birds or other animals (internally through the gut or externally on the fur), natural migration, rhizomial growth.  Consider potential vectors of the pest in the PRA area, the presence of natural barriers, and the suitability of the environment. In this question the mean rate of spread should be taken into account to decide on the rating. The maximum spread capacity should be described in the justification text and the corresponding rating may also be given when the assessors considers it important to describe different scenarios.  Spread can be described as distance covered per unit time (e.g. 50 m /year) or in increasing area occupied (e.g. km^2^) over time.  very low rate of spread, low rate of spread, moderate rate of spread, high rate of spread, very high rate of spread   | Level of uncertainty: | Low | Medium | High | | --- | --- | --- | --- | |
| --- | --- | --- | --- | --- |
| **Rating guidance** |
| 1) The pest cannot spread naturally (the vector is absent or it can only spread by intervention of man (e.g. grafting or budding)) or the pest has a very low rate of spread (less than 10 m per year)?  Yes very low rate of spread  No go to 2 |
| 2) The pest has a low mobility (10 m to 1 km per year) that only allows movement within production sites or within sites of suitable habitat?  (Spreading to occupy a circular area at a linear speed of between 10 m and 1 km per year would, within 4 years, lead to up to 50 km^2^ being occupied)  Yes low rate of spread  No go to 3 |
| 3) The pest has a medium mobility (1km to 10km per year)?  (Spreading to occupy a circular area at a linear speed of between 1 and 10 km per year would, within 4 years, lead to between approximately 50 km^2^ and 5,000 km^2^ being occupied)  Yes moderate rate of spread  No go to 4 |
| 4) The pest has a high mobility (between 10 and 50 km per year)?  (Spreading to occupy a circular area at a linear speed of between 10 and 50 km per year would, within 4 years lead to approximately 5,000 and 125,000 km^2^ being occupied)  Yes high rate of spread  Are there natural barriers that would prevent the natural spread of the pest?  Yes moderate rate of spread  No go to 5 |
| 5) The pest has a very high mobility (more than 50 km/year)?  (Spreading to occupy a circular area at a linear speed of 50 km per year would, within 4 years over 125,000km^2^ would be occupied)  Yes very high rate of spread  Are there natural barriers that would prevent the natural spread of the pest?  Yes high rate of spread (or even lower, depending on e.g. the location and dimension of the natural barriers) |

| ***G. circinata***  Rating: **moderate rate of spread**  Level of uncertainty : **medium**  The pathogen can disperse with wind or in water splash (larger than 200 m from an infected pine; Blakeslee et al., 1979; Dwinell et al., 1981; Garbelotto et al., 2008) and also with bark beetles. In mark_recapture experiments, bark beetles, *Ips sexdentatus*, were caught at distance up to 4 km (Jactel 1991). However it may not necessarily establish because wounds are necessary for a successful infection (EFSA 2010). |
| --- |

| - 1. **What is the most likely rate of spread by human assistance (in the PRA area)?**   Note: consider the potential for movement with commodities, packing materials, baggage, mail or conveyances, the fact that the species is intentionally dispersed by people and the ability of the pest to be unintentionally dispersed along major transport routes. For intentionally introduced plants consider spread to the unintended habitat.  Mechanical transmission through human activities (by grafting or budding and contamination of hands, clothing and tools used for pruning, cutting, thinning and preparing the soil) commonly occurs over short distances within the place of production. However, since employees often travel long distances to work and contract workers (that visit many production sites) are commonly employed, it is considered that evidence of mechanical transmission indicates the potential for at least moderate spread.  Very low rate of spread, moderate rate of spread, high rate of spread, very high rate of spread   | Level of uncertainty: | Low | Medium | High | | --- | --- | --- | --- | |  |
| --- | --- | --- | --- | --- | --- |
| Rating guidance | |
| 1 Has a pathway that is not natural spread been identified for this pest?  If yes, the rate of increase in the infested area by human assistance is at minimum moderate go to 2  If no, the rate of increase in the infested area by human assistance is very low | |
| 2 Can the pest be transmitted by pollen, seed or (other) plants for planting (cuttings, budwood grafted plants, etc.), plant products, with packaging, conveyance machinery?  If yes, the rate of increase in the infested area by human assistance is at minimum high go to 3  If no, the rate of increase in the infested area is moderate | |
| 3 Is the pathway on which the pest is likely to be present widely distributed in the PRA area (trade or movement with persons) or is the pest likely to be moved intentionally by persons in the PRA area? (unintentional movement)  If yes, the rate of increase in the infested area by human assistance is very high  If no, the rate of increase in the infested area by human assistance is high | |

| ***G. circinata***  Rating: **very high rate of spread**  Level of uncertainty: **medium**  The pathogen can be moved unintentionally when trees, pine seeds, wood commodities, wood packages, wood bark are transported (EFSA 2010). Only few data are available about this pathway but, due to the location of infected areas, there is some evidence that the pathogen can be introduced by human assistance (e.g., isolated outbreaks found in France – Vosges, Vendée, and Côtes d'Armor, EPPO 2009a). |
| --- |

| Conclusion on the probability of spread   - 1. **Describe the overall rate of spread**  *Note*: The overall rate for spread should combine the assessments of the rate for natural spread and human spread. In most situations the overall rate of spread equals the highest rate of spread given to either question 4.01 or 4.02. **very low rate of spread, low rate of spread, moderate rate of spread, high rate of spread, very high rate of spread**   | Level of uncertainty: | Low | Medium | High | | --- | --- | --- | --- |   **The assessor should also give his/her best estimate for the following questions:**   - 1. **What is your best estimate of the time needed for the pest to reach its maximum extent in the PRA area?**   *Note*: In this question, ignore any containment measures (considered in question 4.03) that may be taken to prevent or contain the spread of the pest. The maximum extent can be considered to be the area of potential establishment defined in question 3.08.  The factors to be taken into account in deciding on the time to reach its maximum extent include:   - The rate of spread, - The survival and reproductive rate - The relationship between population density and impact thresholds - The time taken for impacts to be observed, e.g. through a lag phase - Climate and land use change   1. **Based on your responses to questions 4.01, 4.02, and 4.05 while taking into account any current presence of the pest, what proportion of the area of potential establishment do you expect to have been invaded by the organism after 5 years?**  | Level of uncertainty: | Low | Medium | High | | --- | --- | --- | --- |   Go to the next section |
| --- | --- | --- | --- | --- | --- | --- | --- | --- |

| ***G. circinata***  Rating 4.03: **very high rate of spread**  Level of uncertainty: **medium**  ***G. circinata***  Rating 4.04: **10 years**  Level of uncertainty: **high**  ***G. circinata***  Rating 4.05: **50%**  Level of uncertainty: **high**  It is also difficult to say because spread seems to occur mainly at long distance between points (nurseries), therefore the area of spread would perhaps remain relatively restricted compared to the area of potential establishment. If we ignore containment measures, the spread will mainly depends on human-mediated transportation, which is a rather random process in the number of transportations and in the geographical areas connected. For these two questions, it seems essential to obtain some simulations from the spread module to define more clearly a range of possible outcomes. |
| --- |

**Application of the spread module**

The main source of information to find the estimates of the parameters is a paper published by Cook & Matheson in 2008. They developed a generic model that combined spread and economic impact. They estimated the parameters based on Zadocks & Shein (1979), refined by expert opinion. Although these estimates may be subject to errors, we based our spread model on them because, at least until now, no other estimates were available to our knowledge.

We used the CLIMEX model developed by Ganley et al. (2009) and the *Pinus* distribution provided by EFI (Tröltzsch et al. 2009) (Fig. 7-1) to determine the area of potential establishment (Fig. 7-2) and the adjusted GI map (Fig. 7-3).

**Fig. 7-1**. Proportion of land covered by pines from 0 (light green) to 1 (dark green).

**Fig. 7-2**. Area of potential establishment (EI > 0 and presence of host trees).

**Fig. 7-3**. Adjusted GI from 0 (light yellow) to 100 (red).

**Model C: Population dynamics model**

*For this model we assume that an initial population abundance p_0_ (%) is introduced in each suitable cell (cells where EI > 0) and then we simulate the “spread” or more precisely the growth within each cell according to a logistic function. On the output map, we will see areas which are the most suitable for the population growth if some individuals were introduced there.*

- **Starting population p_0_**p_0_ is the population abundance for all suitable cells at time t = 0 expressed as a percentage of the maximum abundance (carrying capacity P_max_):

$p_{0}=100*\frac{P_{0}}{P_{\max}}$ with *P*_0_ the number of introduced individuals, in each suitable cell, and *P_max_* the carrying capacity (the maximum number of individuals in a cell).

We used the following estimates from Cook & Matheson (2008):

- for P_0_:

Nmin = Pert (1,2,3) the pest density immediately upon establishment (supposedly per ha).
We used the most likely value: P_0_ = 2 * 100

- for P_max_:

K(Nmax) = Pert (10000,55000,100000) the carrying capacity per ha

proportion_covered_by_host = mean (host)= 6%
We used the most likely value: K = 55000*100*1576 (area of cell)*0.06 (mean proportion of host)

P_max_ = 5.21 10^8^

Therefore p_0_ = 100* P_0_ / P_max_ => N_0_ = 3.8 10 ^-5^

- **Multiplication factor λ_max_**(λ_max_ is the maximum year to year multiplication factor (“finite growth rate”) that a population could achieve under optimal conditions assuming unlimited space. Try to consider how the number of infested trees may have developed from one year to the next or over several years and then calculate the multiplication factor from these data.

From Cook & Matheson (2008): r = Pert (0.5,0.75,1.0) the intrinsic rate of population growth

Since the required value is the maximum yearly multiplication factor, we considered the higher value of r and calculated:

λ_max_ = expr(r_max_)=exp(1) => λ_max_ = 2.72

**Results**

**Fig. 7-4**. Outputs of model C for t = 10, 20, 30, and 40 (blue dots: 0 < p_t_ < = 25, green dots: 25 < p_t_ < = 50, orange dots: 50 < p_t_ < = 75, red dots: 75 < p_t_ < = 100, grey dots: p_t_=0, white: no data).

From these simulations, it seems that the pathogen population needs a long time to start increasing.

**Model D: Deterministic version of the dispersal kernel model**

*This model combines a population growth model and a dispersal kernel (2Dt)*

- **Starting population p_0_**p_0_ = 3.8 10 ^-5^ (see model C)
- **Multiplication factor λ_max_**λ_max_ = 2.72 (see model C)
- **Scale parameter u (km) and shape parameter υ**Since the pathogen cannot disperse at very long distance unless transported by humans, we used an arbitrary small scale parameter (u = 1 km) and an arbitrary small shape parameter p = 2 (thick tail, long distance).

**Results**

**Fig. 7-5.** Outputs of model D for t = 3, 5, and 10 (p_t_: population abundance (%); graduated colors from white (p_t_ < 10^(-6) %) to yellow, orange and red (p_t_ > = 10%). Grey means no data)

Despite very small short-distance spread, it seems that the disease could spread very fast across Europe (based on very arbitrary values of the parameters). This model is not appropriate because it does not simulate long distance jumps but a continuous spread from short to long distance spread.

**Summary**

For this pathogen, it was relatively difficult to estimate the parameters for the spread models and also to assess the potential spread qualitatively. Although some values are chosen arbitrarily, the spread module can be used to simulate some possible outcomes and help the risk assessor to answer some of the spread questions in the EPPO DSS for PRA. Model D can provide some indications to the answers for:
- question 4.04: it seems that the pest could reach its maximum extent within 10 years.

- question 4.05: after 5 years, around a half of the area of potential establishment could be infested by the pest at relatively high densities.

Caution is however needed in this particular case because of high uncertainties and low performance of the model to simulate long distance jumps.

Model C was useful to show that the pathogen population may not build up rapidly. Model D was not applied because of the lack of economic data and maps to calculate the relative rate of spatial increase. Model B was not appropriate because the pathogen mainly makes some long distance jumps and therefore its spread cannot be simulated through a radial rate of expansion.

**Commands for modelling the spread of Pitch canker disease, *Gibberella circinata***

library(sp)

library(raster)

library(rgdal)

elevmax = F

habitatfile = T

*# load the code*

plothabitat()

plotRA()

plotGI()

res = slg(N0 = 3.8*10^(-5), lmax = 2.72, t = 10)

res = dispk(lmax= 2.72, presencefile=F, N0= 3.8*10^(-5), nentry=1, p=2, u=1, t = 10)

**References**

Anonymous 2009. Diagnostic protocol for Gibberella circinata. EPPO Bulletin 39: 298-309.

Carlucci, A.; Colatruglio, L.; Frisullo, S. 2007. First report of pitch canker caused by Fusarium circinatum on Pinus halepensis and P. pinea in Apulia (Southern Italy). Plant Disease 91: 1683.

Bragança H, Diogo E, Moniz F, Amaro P 2009. First report of pitch canker on pines caused by Fusarium circinatum in Portugal. Plant Disease 93: 1079.

Cook D.C. & Matheson A.C. 2008. An estimate of the potential economic impact of pine pitch canker in Australia. Australian Forestry, 71, 107-112.

EFSA 2010. Risk assessment of Gibberella circinata for the EU territory and identification and evaluation of risk management options. EFSA Journal 8:Article 1620.

EPPO 2005. Eppo Reporting Service 2005/097. First record of Gibberella circinata (pine pitch canker) in Spain.

EPPO 2006a. Reporting Service 2006/050. Further details on the situation of Gibberella circinata in Spain.

EPPO 2006b. Eppo Reporting Service 2006/104. First report of Gibberella circinata in France.

EPPO 2008. Eppo Reporting Service 2008/103. Gibberella circinata eradicated in France.

EPPO 2009a. Eppo Reporting Service 2009/093. Situation of Gibberella circinata in France.

EPPO 2009b. Eppo Reporting Service 2009/052. Gibberella circinata eradicated from Italy.

EPPO 2009c. Eppo Reporting Service 2009/196. First report of Gibberella circinata in Portugal.

EPPO 2010. Eppo Reporting Service 2010/034. Gibberella circinata detected again in France.

Ganley RJ, Watt MS, Manning L & Iturritxa E (2009) A global climatic risk assessment of pitch canker disease. Can J For res, 39:2246-2256.

Garbelotto, M.; Schweigkofler, W.; Shaw, D. 2007. First report of Fusarium circinatum, causal agent of pitch canker disease, from the roots of mature Aleppo pines in California. Plant Health Progress (February): 1-2.

Gordon, TR; Storer, AJ; Wood, DL 2001. The pitch canker epidemic in California . Plant Disease 85:1128-1139.

Sakamoto, J. M.; Gordon, T. R. 2006. Factors influencing infection of mechanical wounds by Fusarium circinatum on Monterey pines (Pinus radiata). Plant Pathology 55: 130-136.

Schweigkofler, W; O'Donnell, K; Garbelotto, M 2004. Detection and quantification of airborne conidia

Tröltzsch K, Van Brusselen J, Schuck A (2009) Spatial occurrence of major tree species groups in Europe derived from multiple data sources. For Ecol & Manag 257: 294-302. doi:10.1016/j.foreco.2008.09.012.

Zadoks, J., and R. Schein. 1979. Epidemiology and Plant Disease Management. Oxford

University Press, Oxford.
